# Long–Short-Arm Acridine Ru-Pincer Catalysts for Reversible Hydrogen Storage Based on Ethylene Glycol

**DOI:** 10.1021/jacs.5c07428

**Published:** 2025-08-07

**Authors:** Cai You, Lijun Lu, Jie Luo, Yael Diskin-Posner, David Milstein

**Affiliations:** † Department of Molecular Chemistry and Materials Science, 34976 Weizmann Institute of Science, Rehovot 76100, Israel; ‡ Department of Chemistry, School of Sciences, Great Bay University, Dongguan 523000, People’s Republic of China; § Department of Chemical Research Support, Weizmann Institute of Science, Rehovot 7610001, Israel

## Abstract

Liquid organic hydrogen carriers (LOHCs) offer an attractive strategy for efficient hydrogen storage and release, thereby facilitating the effective use of hydrogen as a carbon-neutral energy carrier. The advancement of LOHC technology is highly dependent on the innovation of the catalysts. Herein, based on a strategy combining rigidity and flexibility in a single molecular catalyst, a novel class of PNP-pincer ligands, called long–short-arm acridine ligands, and their Ru complexes have been developed and successfully used in the LOHC system based on ethylene glycol (EG). In comparison to previously reported catalytic systems, which suffered from low conversions or insufficient H_2_ release due to the dual challenge of catalyst stability and catalytic activity in the acceptorless dehydrogenative coupling of EG, this new catalytic system overcomes these challenges and achieves high conversion (up to >99%) with high H_2_ yield (up to 96%), achieving a hydrogen storage capacity of 6.2 wt %. Mechanistic and computational studies reveal that the special coordination mode, one 5-membered metallacycle and one 6-membered metallacycle, is essential for the high reactivity and late-stage dehydrogenative coupling. Moreover, both dehydrogenation and hydrogenation can be achieved under solvent- and additive-free conditions, highlighting the robustness and application potential of this new catalytic system. This advances the promising liquid-to-liquid paired LOHC systems based on inexpensive, widely accessible, and biobased EG toward practical application.

## Graphical abstract

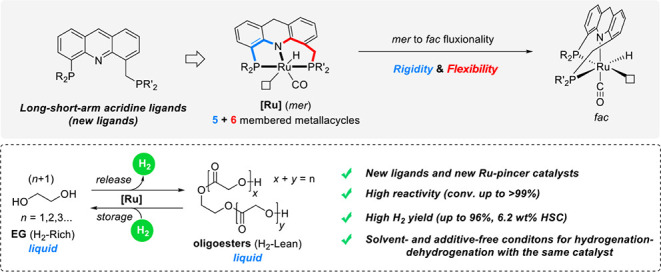

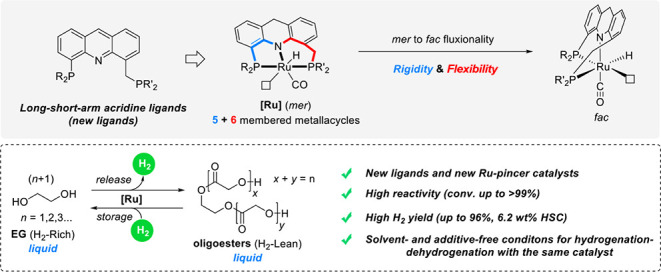

## Introduction

Our excessive consumption of fossil fuels leads to a significant increase in the generation of waste and greenhouse gas emissions, particularly CO_2_, which causes environmental pollution and climate change.^1–4^ Additionally, heavy consumption of fossil fuels is unsustainable because they take millions of years to form and are being depleted much faster than they can be naturally replenished. Thus, there is an urgent need to develop green and renewable energy sources to replace traditional fossil fuels for a sustainable future.^5–7^ In this context, hydrogen, which produces only water upon combustion and could be produced by electrochemical water splitting, is considered as a promising renewable energy carrier.^8–11^ However, although hydrogen possesses the highest gravimetric energy density (33.3 kWh kg^–1^), its low volumetric energy density at ambient conditions (0.003 kWh L^–1^), along with its flammability and broad explosion limits, impedes efficient handling, storage, and transportation, particularly for long-term/long-distance applications.^12–14^ To address these issues, various hydrogen storage methods such as compressed hydrogen,^15^ cryogenic hydrogen,^16^ metal hydrides,^17^ and hydrogen adsorption in porous materials^18^ have been established and investigated, but these methods often suffer from high costs, low capacity, or safety risks. Liquid organic hydrogen carriers (LOHCs), which store hydrogen in covalent bonds of liquid organic compounds, have a high hydrogen storage capacity (HSC) and can be easily handled and transported, representing a promising approach for chemical hydrogen storage in liquid form. Moreover, the compatibility of LOHCs with existing oil and gas transportation infrastructure is a major benefit that can further reduce the costs of storage and delivery, making them more attractive.^19–23^

Recently, simple organic compounds, including formic acid,^24–29^ formaldehyde,^30,31^ methanol,^32–35^ and methyl formate^36^ have been introduced as hydrogen carriers. However, the release of CO_2_ and the inconvenient reloading of H_2_ due to the consumption of these liquid carriers limit these approaches. To advance more efficient hydrogen storage systems, liquid-organic hydrogen carriers (LOHCs) have emerged as a unique and powerful tool, using a pair of H_2_-rich and H_2_-lean organic liquids that can reversibly discharge and load hydrogen via catalytic dehydrogenation and hydrogenation cycles.^19–23^ In this regard, aromatic compounds and their hydrogenated alicyclic compounds have been investigated, but harsh reaction conditions (usually >250 °C) are required, especially for the strongly endothermic dehydrogenation step.^23,37,38^ To lower the enthalpy of hydrogenation/dehydrogenation, LOHCs based on nitrogen-containing heterocycles, which have high HSCs ranging from 5.3 to 7.3 wt %, have been developed. However, high temperatures are still required and result in decomposition products in some cases.^23,39–43^ Recently, our group,^44–47^ Prakash group^48^ and Liu group^49^ have developed LOHCs based on amide bond formation and hydrogenation, and widely available and inexpensive amines along with alcohols have been used in these systems. Nevertheless, the solid nature of these amides (hydrogen-deficient compounds) leads to difficulties in the hydrogenation step as well as transportation complications. Consequently, the pursuit of ideal liquid-to-liquid paired LOHC systems is challenging but highly desirable. Moreover, in line with the principles of sustainable development, the advancement of LOHC systems based on biobased materials is becoming increasingly attractive.

Since 2019, three promising liquid-to-liquid paired LOHC systems based on inexpensive, widely accessible, and biobased alcohols, including ethylene glycol,^50^ 1,4-butanediol,^51^ and ethanol,^52^ have been developed by our group, the Fujita group, and the Tran group, respectively. In comparison to the 1,4-butanediol and ethanol systems, whose theoretical HSCs are 4.5 and 4.4 wt %, respectively, our ethylene glycol system possesses a theoretical HSC of 6.5 wt %, which is above the targets set for 2020 by the European Union (5.0 wt %) and the US Department of Energy (5.5 wt %).^53,54^ Therefore, the economic, environmental, and practical merits of EG^55,56^ make it a promising candidate for LOHC applications (Figure 1a).

**Figure 1:**
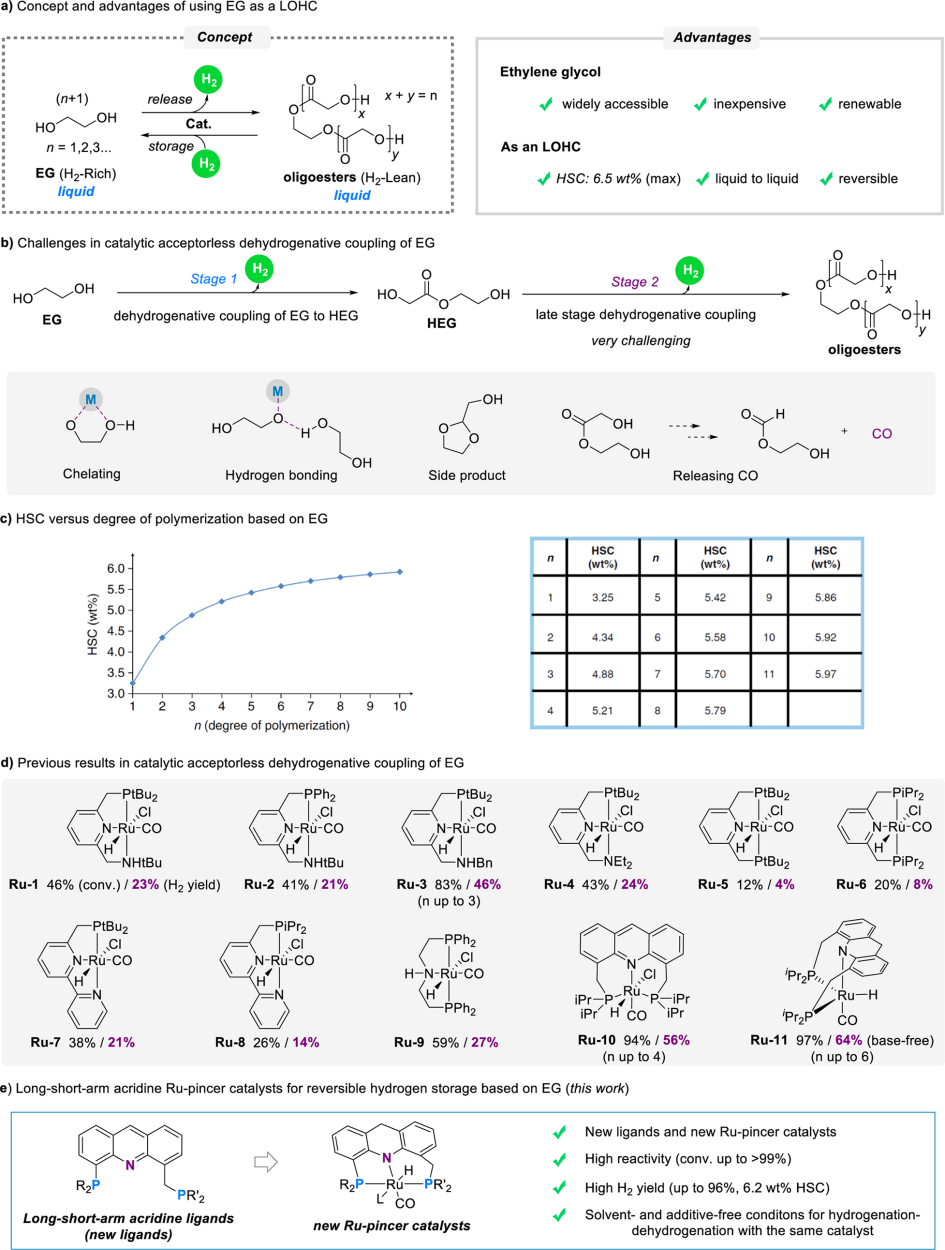
Liquid organic hydrogen carrier (LOHC) system based on ethylene glycol (EG).

Nevertheless, due to encountering a dual challenge of catalyst stability and catalytic activity in the acceptorless dehydrogenative coupling of EG, previously tested catalysts suffer from modest conversions or insufficient H_2_ release, and the development of a more efficient catalyst for this process is desirable.

The reaction pathway for the LOHC based on EG comprises two parts (Figure 1a, left): the acceptorless catalytic dehydrogenative coupling of EG (hydrogen release process) and the hydrogenation of the corresponding oligoesters back to EG (hydrogen storage process). For the hydrogen release process, the initial step involves the coupling of two molecules of EG to form 2-hydroxyethyl glycolate (HEG), catalyzed by a metal pincer complex, with the concomitant release of 2 equiv of hydrogen. Subsequently, HEG can react with additional equivalents of EG in a similar manner to produce higher oligomers (Figure 1b). However, the acceptorless catalytic dehydrogenative coupling of EG to HEG or corresponding oligoesters is highly challenging due to potential drawbacks that may explain EG’s reluctance to efficiently undergo the desired transformation, including: (1) EG chelates the metal center of the pincer complex, hampering catalyst activity; (2) hydrogen bonding between a possible alkoxy metal complex and neighboring EG may hinder the β-hydride elimination steps, preventing the generation of the aldehyde intermediate; (3) HEG can be dehydrogenated to an α-keto ester upon oxidation of the α-hydroxyl group, which could decompose to CO and aldehyde, leading to CO poisoning of the catalyst; and (4) the undesired formation of cyclic side products ((1,3-dioxolan-2-yl)­methanol) with lower hydrogen storage capacities. Crucially, as higher HSC is directly contingent upon achieving greater degrees of oligomerization (Figure 1c), the catalytic capability to facilitate high levels of oligomerization becomes a critical determinant of the success of the system. However, this poses a significant challenge for catalysts due to the increased steric hindrance and the more complex structures of these oligoesters.

Previously, a series of pincer catalysts, which catalyze hydrogenation and dehydrogenation reactions, including PNNH-Ru (**Ru-1**, **Ru-2**, and **Ru-3**), PNN-Ru (**Ru-4**, **Ru-7**, and **Ru-8**), PNP-Ru (**Ru-5** and **Ru-6**), and MACHO-Ru (**Ru-9**), were tested by us^50^ and others^57^ in the dehydrogenative coupling of EG. However, the low conversions and insufficient H_2_ yields (4–46%) demonstrated that these catalyst systems are inefficient for this transformation, underscoring the challenges of this process (Figure 1d). In contrast, our acridine-based complex **Ru-10** and its dearomatized version **Ru-11** achieved significantly better results, with **Ru-10** affording 94% conversion and 56% H_2_ yield, and **Ru-11** reaching 97% conversion and 64% H_2_ yield. Despite **Ru-11**’s improved performance, this result remains insufficient, particularly for practical applications. Therefore, the development of new catalysts that can overcome the limiting factors in the dehydrogenative coupling of EG, give higher reactivities, and give better H_2_ yields is highly desirable.

Herein, we report the design of novel PNP-Ru complexes based on innovative long–short-arm acridine-based ligands and their application in the LOHC system using ethylene glycol (Figure 1e). These complexes facilitate a highly efficient dehydrogenative coupling of EG, achieving high conversions (up to >99%) and a hydrogen yield exceeding 90% (up to 96%) for the first time, resulting in a hydrogen storage capacity of up to 6.2 wt %. Notably, the entire cycle of the EG-LOHC system, encompassing both dehydrogenation and hydrogenation, has been successfully achieved for the first time using a single catalyst under solvent- and additive-free conditions.

## Results and Discussion

### Catalyst Design and Preparation

The acridine ligand framework within **Ru-10** and **Ru-11**, distinct from other tested Ru-pincer catalysts (Figure 1d), forms two six-membered metallacycles upon coordination, which imparts structural flexibility. This unique flexibility enables access to the *fac* isomer, playing a crucial role in overcoming various mechanistic challenges (Figure 2a).^58,59^ Given the superior performance of complexes with an acridine-based backbone in the current dehydrogenative coupling of ethylene glycol, developing novel complexes based on new acridine-based ligands is a promising direction. Herein, we designed a series of novel PNP-pincer ligands termed long–short-arm acridine-based ligands. In comparison to our previous acridine-based ligands, which featured two long arms, our current design modifies one of the long arms to a short arm, forming a five-membered metallacycle.^60^ This adjustment enhances the rigidity of the catalyst, potentially improving the stability of the complexes. At the same time, to maintain flexibility in the new structure, allowing for the formation of a fac isomer, which is crucial for the current transformation, we retained one long arm to form a flexible six-membered metallacycle (Figure 2b).

**Figure 2:**
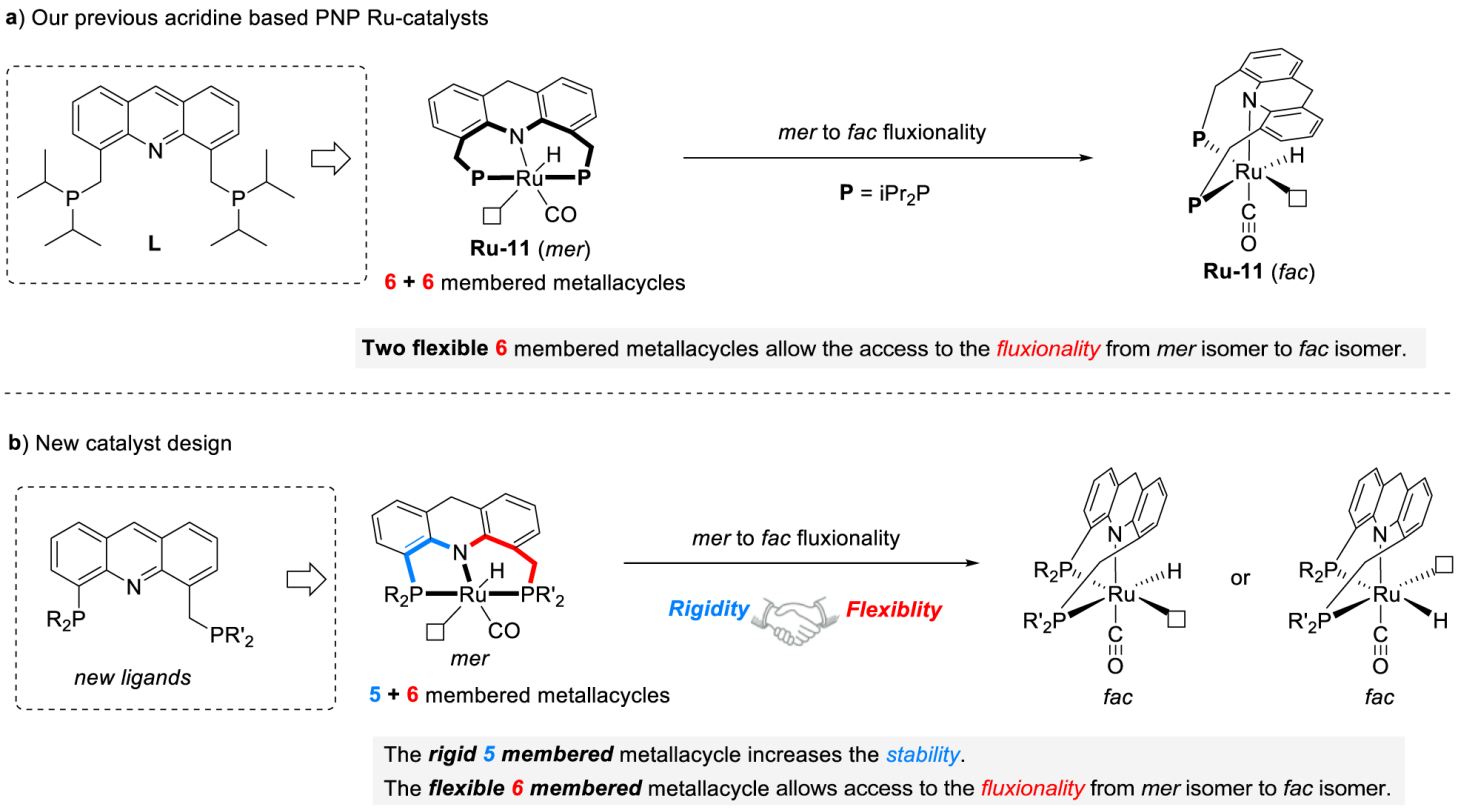
Catalyst design. a) Analysis of the fluxionality ability of **Ru-11**. b) Conceptualization and design of novel PNP-Ru-pincer catalysts utilizing innovative long–short-arm acridine ligands.

On the basis of these ideas, a new long–short-arm acridine ligand, **L1**, was synthesized. The corresponding novel pincer complex, **LS-Ru-1-Cl**, was prepared by the reaction of **L1** with [RuCl_2_(DMSO)_3_(CO)] ^61,62^ in THF at 60 °C for 7 h (Figure 3a). The NMR spectra of **LS-Ru-1-Cl** showed two doublet phosphine signals at 57.2 ppm (*J* = 22.9 Hz) and 56.4 ppm (*J* = 22.0 Hz) in the ^31^P NMR spectrum. The structure of **LS-Ru-1-Cl** was determined by single-crystal X-ray crystallography and exhibited a distorted octahedral structure with the CO and N ligands *trans* to each other and the two phosphorus “arms” in mutually *cis* positions, which performs a *fac* coordination model (Figure 3e, top). As shown in Figure 3b, the reaction of **LS-Ru-1-Cl** with 2 equiv of NaHBEt_3_ in a THF solution at room temperature for 35 min, gave the dearomatized complex, **LS-Ru-1**, which is expected to be utilized in dehydrogenation and hydrogenation reactions in a base-free manner. The NMR spectra of complex **LS-Ru-1** showed two doublet phosphine signals at 60.4 ppm (*J* = 255.0 Hz) and 50.7 ppm (*J* = 257.8 Hz) in the ^31^P NMR spectrum.

**Figure 3:**
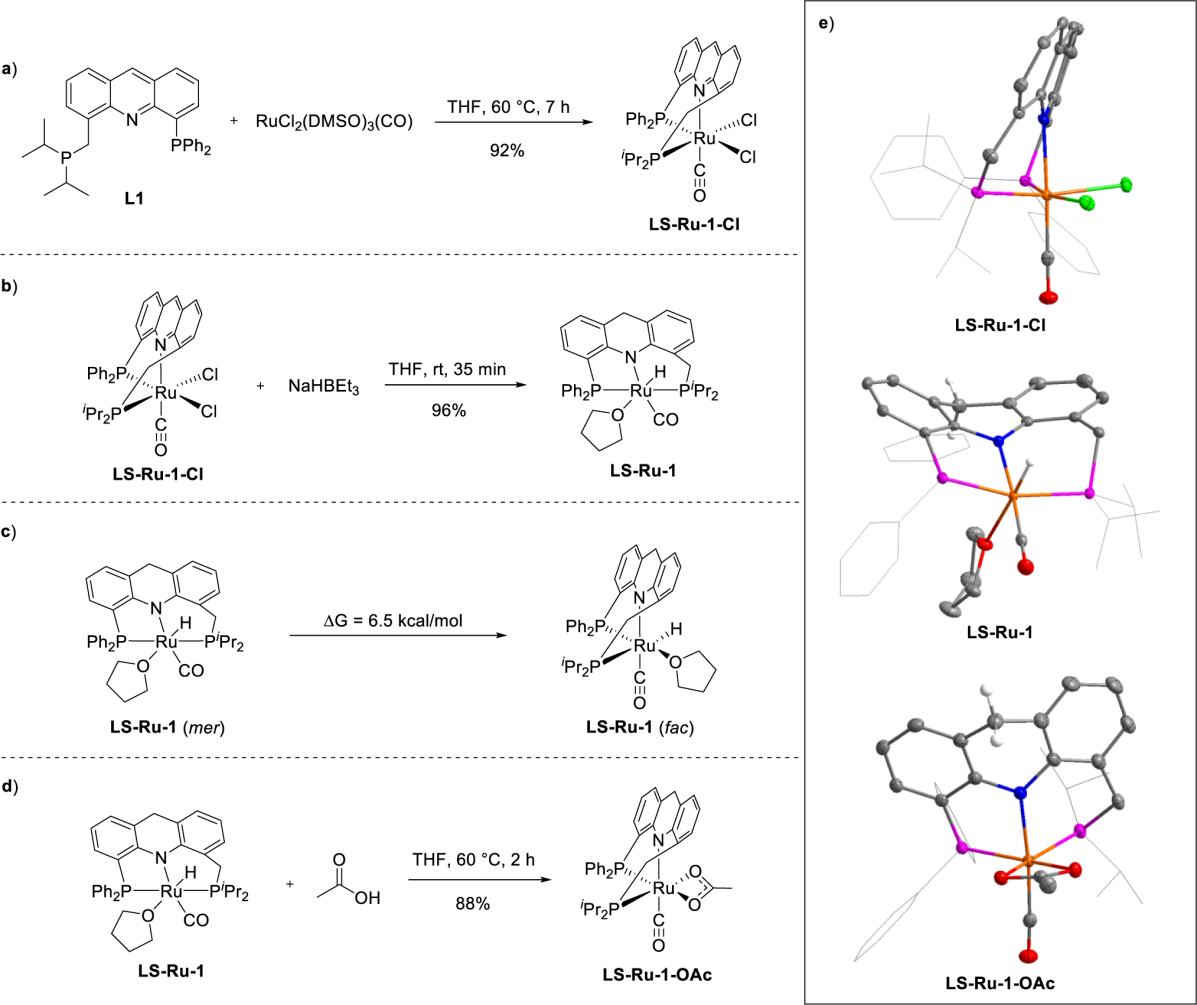
Preparation of **LS-Ru-1** and investigations of its fluxionality. a, Synthesis of **LS-Ru-1-Cl** from **L1** and RuCl_2_(DMSO)_3_(CO). b, Preparation of **LS-Ru-1** from **LS-Ru-1-Cl** with NaHBEt_3_ as the reductant. c, Density functional theory (DFT) calculations to investigate the fluxionality of **LS-Ru-1**. d, Reaction of **LS-Ru-1** with acetic acid. e, Crystal structures of **LS-Ru-1-Cl** (top), **LS-Ru-1** (middle), and **LS-Ru-1-OAc** (bottom).

The structure of the complex **LS-Ru-1** was then determined by single-crystal X-ray crystallography and exhibited a distorted octahedral structure with the CO and N ligands trans to each other and the two phosphorus “arms” in mutually *trans* positions, which performs a *mer* coordination model (Figure 3e, middle).

At the outset, it was unclear whether this new acridine-based pincer complex **LS-Ru-1** would be flexible enough to allow *mer*-*fac* fluxionality. Thus, we performed density functional theory (DFT) calculations on *mer*-**LS-Ru-1** and *fac*-**LS-Ru-1**. As shown in Figure 3c, our calculations indicate that *fac*-**LS-Ru-1** is only 6.5 kcal mol^–1^ less stable than *mer*-**LS-Ru-1**, indicating a very easy interconversion between these two isomers. From the experimental aspect, the reaction of **LS-Ru-1** with 1 equiv of acetic acid in a THF solution at 60 °C for 2 h gave a kinetically stable complex **LS-Ru-1-OAc** (Figure 3d). The ^1^H NMR spectrum of **LS-Ru-1-OAc** did not show any characteristic hydride peaks, as expected. The ^31^P NMR spectrum of **LS-Ru-1-OAc** showed two doublet phosphine signals at 65.0 ppm (*J* = 29.5 Hz) and 56.5 ppm (*J* = 29.5 Hz). The structure of complex **LS-Ru-1-OAc** was then determined by single-crystal X-ray crystallography; it exhibited a distorted octahedral structure with the CO and N ligands trans to each other and the two phosphorus “arms” in mutually *cis* positions, in *fac* coordination (Figure 3e, bottom). Therefore, both computational and experimental results suggest the *mer*/*fac* fluxional ability of this new complex, **LS-Ru-1**.

### Acceptorless Dehydrogenative Coupling of Ethylene Glycol (EG)

With 0.5 mol % **LS-Ru-1** as the catalyst, the base-free dehydrogenative coupling of EG was carried out at 150 °C in a 1:1 (v/v) mixture of toluene and 1,2-dimethoxyethane as the solvent. To our delight, >99% conversion of EG, a very high H_2_ yield (92%) with 99.00% purity, and oligoesters with high degrees of oligomerization (*n* up to 8) were achieved (Figure 5), indicating superior performance compared to our previous catalyst, **Ru-11**, which required 1.0 mol % loading, achieved 97% conversion, 64% H_2_ yield, and oligomers with *n* up to 6. To examine the temperature dependence of the catalytic performance, we conducted a control experiment using **LS-Ru-1** at a lower reaction temperature of 135 °C. Under otherwise identical conditions, the conversion of EG dropped to 85%, and the H_2_ yield decreased significantly to 58%. These results indicate that 150 °C is close to the minimum temperature required to maintain efficient catalytic turnover and effective dehydrogenation in the current system. To investigate the impact of phosphine ligands with different substituents, a series of novel long–short arm acridine-based ligands (**L2**, **L3**, and **L4**) were synthesized, followed by the preparation of their dearomatized Ru-pincer complexes (**LS-Ru-2**, **LS-Ru-3**, and **LS-Ru-4**) in a two-step process, as shown in Figure 4a. It is important to note that the presence of pyridine is essential for stabilizing these dearomatized Ru-pincer complexes. The structures of **LS-Ru-3-Cl** and **LS-Ru-3** were determined by single-crystal X-ray crystallography and exhibit similar coordination models to those of **LS-Ru-1-Cl** and **LS-Ru-1**, respectively (Figure 4b). Next, their performance in the base-free dehydrogenative coupling of EG was investigated (Figure 5). **LS-Ru-2**, featuring a diphenylphosphine group on the long arm, achieved 94% conversion but yielded only a moderate H_2_ production (63%, 98.52% purity), along with lower degrees of oligomerization, with *n* reaching only up to 3. In contrast, **LS-Ru-3**, which incorporates a dithienylphosphine group on the short arm, achieved a significantly higher H_2_ yield of 96% (98.70% purity). Analysis of the reaction mixture also revealed that higher degrees of oligomerization, with *n* reaching up to 14, were attained. However, substituting the dithienylphosphine in **LS-Ru-3** with a difurylphosphine in **LS-Ru-4** resulted in a dramatically lower H_2_ yield of 58% (98.98% purity) and much lower degrees of oligomerization, with *n* reaching only up to 3. These comparative results highlight the critical role of phosphine substitution patterns in modulating catalytic performance. In particular, the superior performance of **LS-Ru-1** over that of **LS-Ru-2** suggests that the diisopropylphosphine group on the long arm plays an essential role. While the exact origin of this effect remains unclear, our working hypothesis is that the steric and electronic properties of the diisopropylphosphine may beneficially influence the structure of the active species or the stability of key catalytic intermediates. Furthermore, the significantly improved performance of **LS-Ru-3** relative to **LS-Ru-1** is likely attributed to the reduced steric hindrance of the dithienylphosphine substituent on the short arm, which may create a less congested coordination environment, thereby facilitating chain propagation and resulting in higher degrees of oligomerization and enhanced H_2_ yield. In contrast, **LS-Ru-4**, which bears a difurylphosphine group with steric properties similar to those of **LS-Ru-3**, exhibited markedly reduced activity. This observation may be due to the lower electron density of the difurylphosphine moiety, which could compromise the stability of the active catalyst species. However, this remains a working hypothesis and further studies are required to clarify the underlying factors.

**Figure 4:**
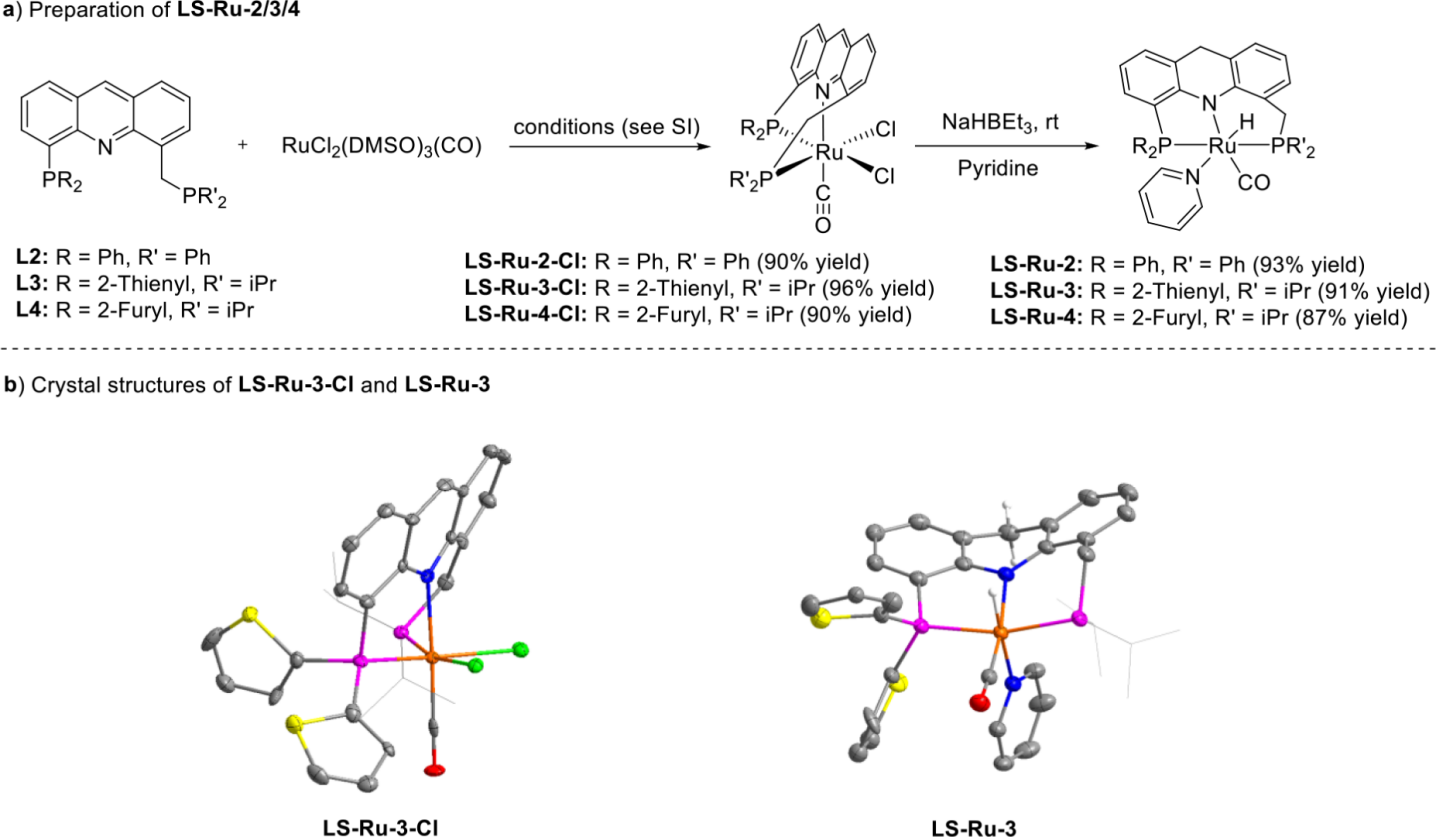
Preparation of **LS-Ru-2/3/4** from **L2-L4** and crystal structures of **LS-Ru-3-Cl** (bottom left) and **LS-Ru-3** (bottom right).

**Figure 5:**
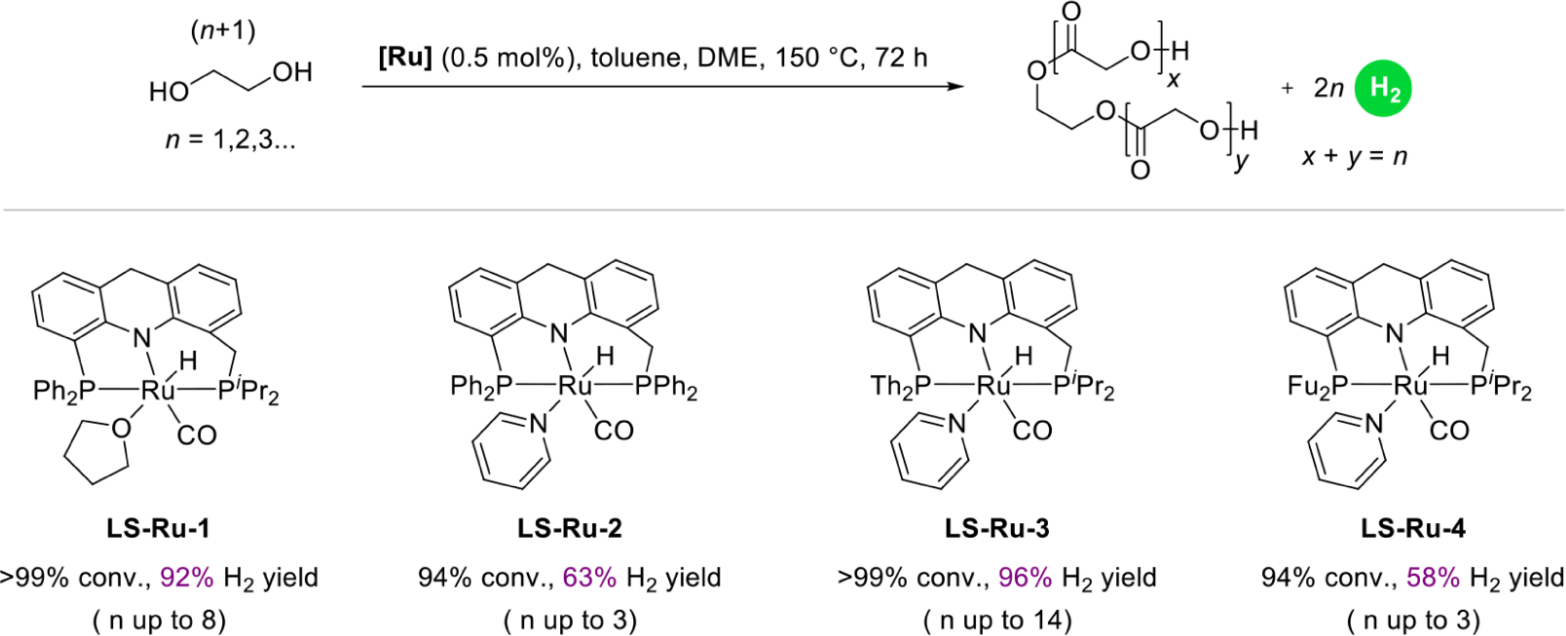
Long–short-arm acridine Ru-pincer complexes catalyzed the base-free dehydrogenative coupling of EG. Th = 2-Thienyl, Fu = 2-Furyl.

### Investigation of Differences between LS-Ru-1 and Ru-11

In order to gain a deeper understanding of the differences between **LS-Ru-1** and **Ru-11**, a series of experiments were designed and conducted, as shown in Figure 6. First, the base-free dehydrogenative coupling of EG was carried out with different reaction times. As shown in Figure 6a, both the conversion of EG and the H_2_ generation rate are significantly faster in the **LS-Ru-1**-catalyzed system compared to the **Ru-11**-catalyzed system, indicating that the reactivity of **LS-Ru-1** is considerably higher than that of **Ru-11**. There are two potential reasons for the significantly higher H_2_ yield observed in the **LS-Ru-1** system compared to that of the **Ru-11** system:

**Figure 6:**
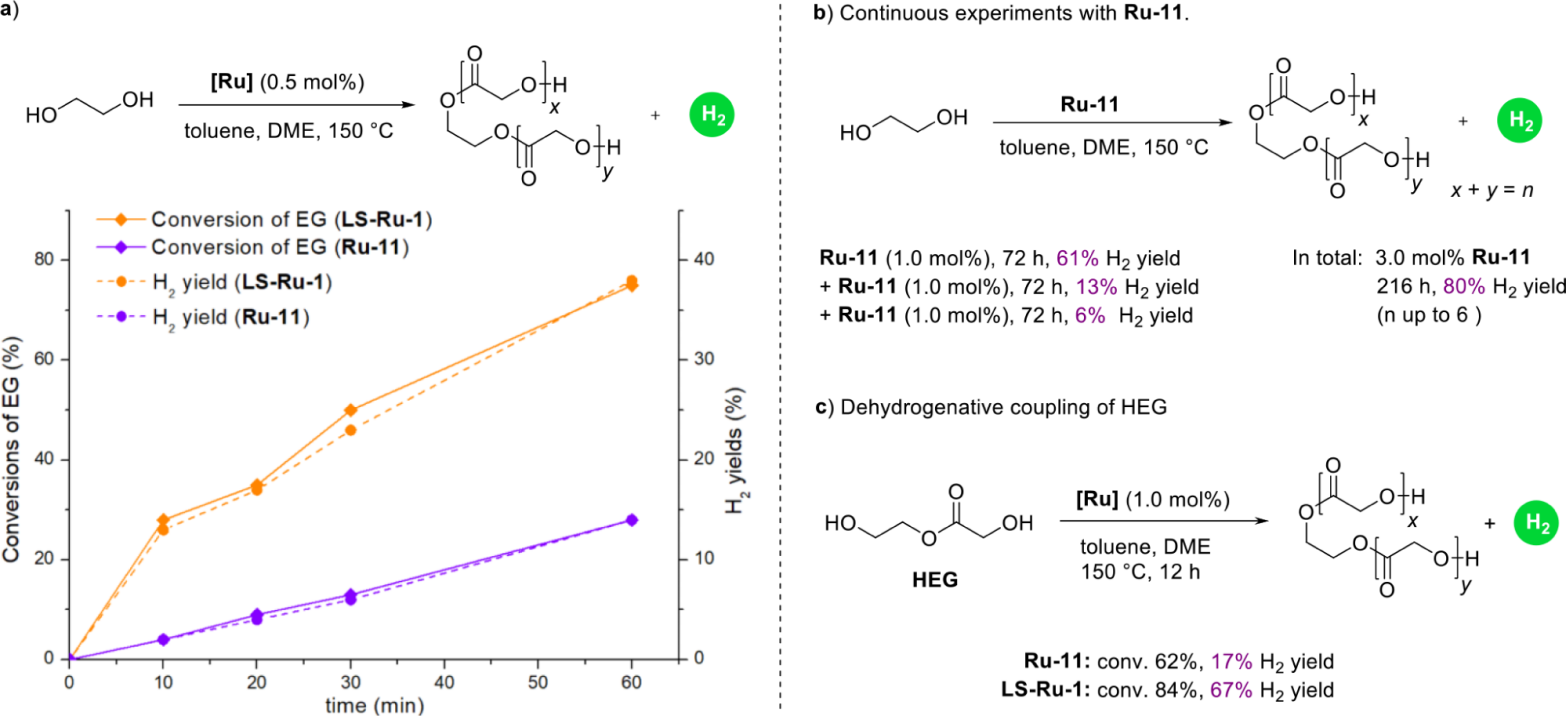
Investigation of different performances of **LS-Ru-1** and **Ru-11** in the dehydrogenative coupling of EG. a, Correlation between reaction time and conversions of EG and H_2_ yields. b, Continuous experiments with **Ru-11**. c, Dehydrogenative coupling of HEG.

1) **LS-Ru-1** exhibits better stability during the current transformation, enabling it to operate for a longer duration; 2) **LS-Ru-1** demonstrates a superior ability to achieve high degrees of oligomerization, particularly during the late-stage dehydrogenative coupling of HEG or other higher-grade oligoesters. As shown in Figure 6b, continuous experiments utilizing 3.0 mol % **Ru-11** over a duration of 216 h (please see the detailed procedure in the Supporting Information) resulted in a total H_2_ yield of 80%, which remains lower than that achieved in in the **LS-Ru-1** system (0.5 mol % **LS-Ru-1**, 92% H_2_ yield) and the **LS-Ru-3** system (0.5 mol % **LS-Ru-3**, 96% H_2_ yield). The maximum degree of oligomerization was limited to 6, which is lower than that observed with the **LS-Ru-1** system (with *n* values up to 8) and the **LS-Ru-3** system (with *n* values up to 14). This result suggests that stability is not the primary factor limiting **Ru-11**’s capacity to achieve a high H_2_ yield. Since HEG should be the primary product in the early stage of the dehydrogenative coupling of EG, we employed HEG as the substrate to further compare the performance of **LS-Ru-1** and **Ru-11**. As shown in Figure 6c, reactions were conducted with 1.0 mol % **LS-Ru-1** or **Ru-11** at 150 °C for 12 h. **LS-Ru-1** produced a significantly higher H_2_ yield (67%) compared to that of **Ru-11** (17%). Given the substantially greater steric hindrance of HEG relative to EG, these results suggest that **LS-Ru-1** is more effective than **Ru-11** in the late-stage dehydrogenative coupling of EG, which is very critical for achieving high H_2_ yields (Figure 1c).

### Mechanistic Studies

To gain mechanistic insights and understand the differences between the current **LS-Ru-1** system and our previous **Ru-11** system, density functional theory (DFT) calculations were conducted to analyze the overall dehydrogenation process of EG to HEG for both catalytic systems (Figure 7). For the *fac*-*mer* fluxionality step, the *fac* isomer of**LS-Ru-1** was found to be 6.5 kcal mol^–1^ higher in energy than the *mer* isomer, and this energy difference suggests that *mer*-**LS-Ru-1** can access *fac*-**LS-Ru-1** easily (Figure 7a). In comparison, the energy difference between the *mer* isomer and *fac* isomer of **Ru-11** is a little higher (10.6 kcal mol^–1^, Figure 7b). Next, due to the dissociation energy of THF from the Ru center, there is a very slight uphill in energy (0.1 kcal mol^–1^) from *fac*-**LS-Ru-1** to the EG-coordinated **LS-Ru-1-a**, while a slight downhill in energy (−2.6 kcal mol^–1^) is calculated in the **Ru-11** system for EG coordination to the five-coordinate complex *fac*-**Ru-11**. Compared with the dehydrogenation of EG complex **Ru-11-a**, dehydrogenation of EG complex **LS-Ru-1-a**, leading to the generation of **LS-Ru-1-b** and H_2_, is less energetically demanding (Δ*G* ^⧧^ = 20.3 kcal mol^–1^ for the reaction **LS-Ru-1-a** to **LS-Ru-1-b**, vs 25.6 kcal mol^–1^ for the reaction **Ru-11-a** to **Ru-11-b**). Decoordination of the hydroxo group allows for β-hydride elimination via TS_b,c_ (8.1 kcal mol^–1^) and reforms a Ru–H bond in **LS-Ru-1-c** (6.4 kcal mol^–1^). With another molecule of EG, **LS-Ru-1-c** undergoes dehydrogenation to **LS-Ru-1-d** (5.6 kcal mol^–1^) via a concerted Zimmerman–Traxler-like six-membered transition state (Δ*G* ^⧧^ = 23.1 kcal mol^–1^). In contrast, the reaction from **Ru-11-c** to **Ru-11-d** exhibits a higher Δ*G* ^⧧^ of 24.6 kcal mol^– 1^, indicating that this process is more energetically demanding than that in the **LS-Ru-1** system. Another β-hydride elimination from κ^2^-hemiacetalate **LS-Ru-1-d** proceeds via transition state **TS**_**d,a**_, with an energy barrier of 11.5 kcal mol^–1^, leading to the release of HEG, after which EG coordination regenerates **LS-Ru-1-a**. Importantly, the overall dehydrogenation of EG to HEG is calculated to be nearly thermoneutral in both the **LS-Ru-1** and **Ru-11** systems, with Δ*G* = 0 kcal mol^–1^, which indicates that the dehydrogenation and hydrogenation events are readily feasible and reversible. Overall, our DFT calculations show that the overall rate-determining process for dehydrogenation of EG to HEG is the dehydrogenation of the second EG molecule to form κ^2^-hemiacetalate **LS-Ru-1-d**, which exhibits an apparent activation barrier of 29.5 kcal mol^–1^. In contrast, the transition state in the **Ru-11** system, TS′_c,d_, exhibits an apparently higher activation barrier of 33.6 kcal mol^–1^. These results tentatively explain the higher reactivity of **LS-Ru-1** compared to **Ru-11** in the dehydrogenative coupling of EG.

**Figure 7:**
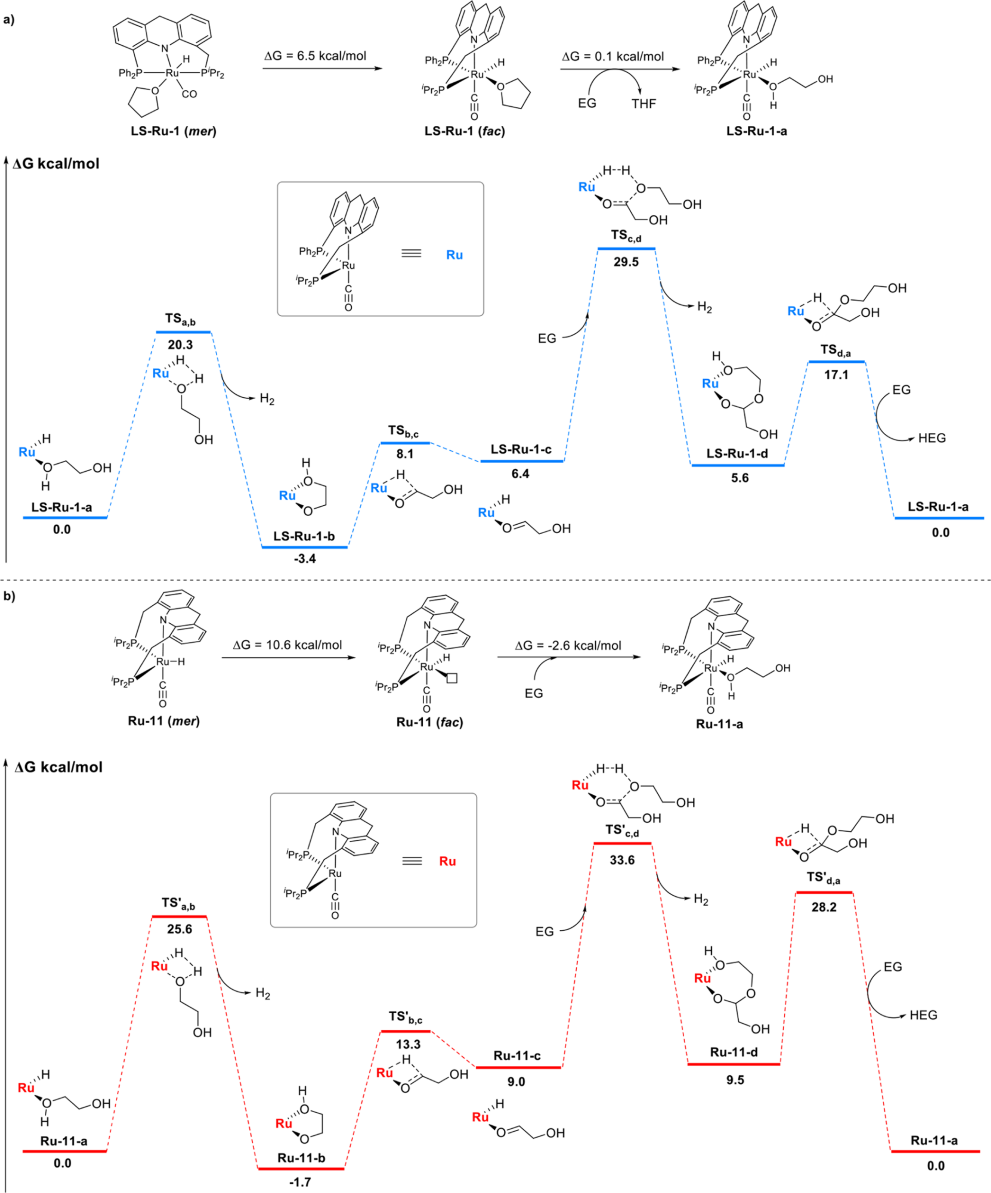
Mechanistic study for acceptorless dehydrogenative coupling of EG into HEG. a, Calculated lowest free energy pathway for acceptorless dehydrogenative coupling of EG into HEG catalyzed by **LS-Ru-1**. b, Calculated lowest free energy pathway for acceptorless dehydrogenative coupling of EG into HEG catalyzed by **Ru-11**. All calculations were performed in the gas phase.

Next, we sought a reasonable explanation for the higher reactivity and enhanced ability for the late-stage dehydrogenative coupling of **LS-Ru-1** compared to **Ru-11**. Based on the crystal structures of **LS-Ru-1-OAc** and **Ru-11-OAc**, topographic steric maps of these Ru-pincer complexes were drawn by SambVca web application^63,64^ to calculate the percentage of buried volume (%*V*_bur_) around the ruthenium center and quantify the steric hindrance of these catalytic pockets (Figure 8). The smaller %*V*_bur_ indicates a larger catalytic pocket. The results illustrated that the smaller hindrance of **LS-Ru-1** is mainly contributed by its acridine part, which is almost perpendicular to the metal phosphine part. Correspondingly, %*V*_bur_ decreases from 64.6% (**Ru-11-OAc**) to 60.4% (**LS-Ru-1-OAc**). Noteworthily, the steric hindrance is drastically reduced in the northern hemisphere of its catalytic pocket in **LS-Ru-1-OAc.** These visible changes in the pocket space and steric hindrance around the Ru center, resulting from the structural differences in the ligands, rationalize the observed variations in performance with different ligands. Therefore, compared to **Ru-11**, the larger catalytic pocket and reduced steric hindrance around the Ru center created by the current long–short arm acridine skeleton in **LS-Ru-1** are advantageous for achieving higher reactivity and a greater degree of oligomerization, resulting in an improved H_2_ yield.

**Figure 8:**
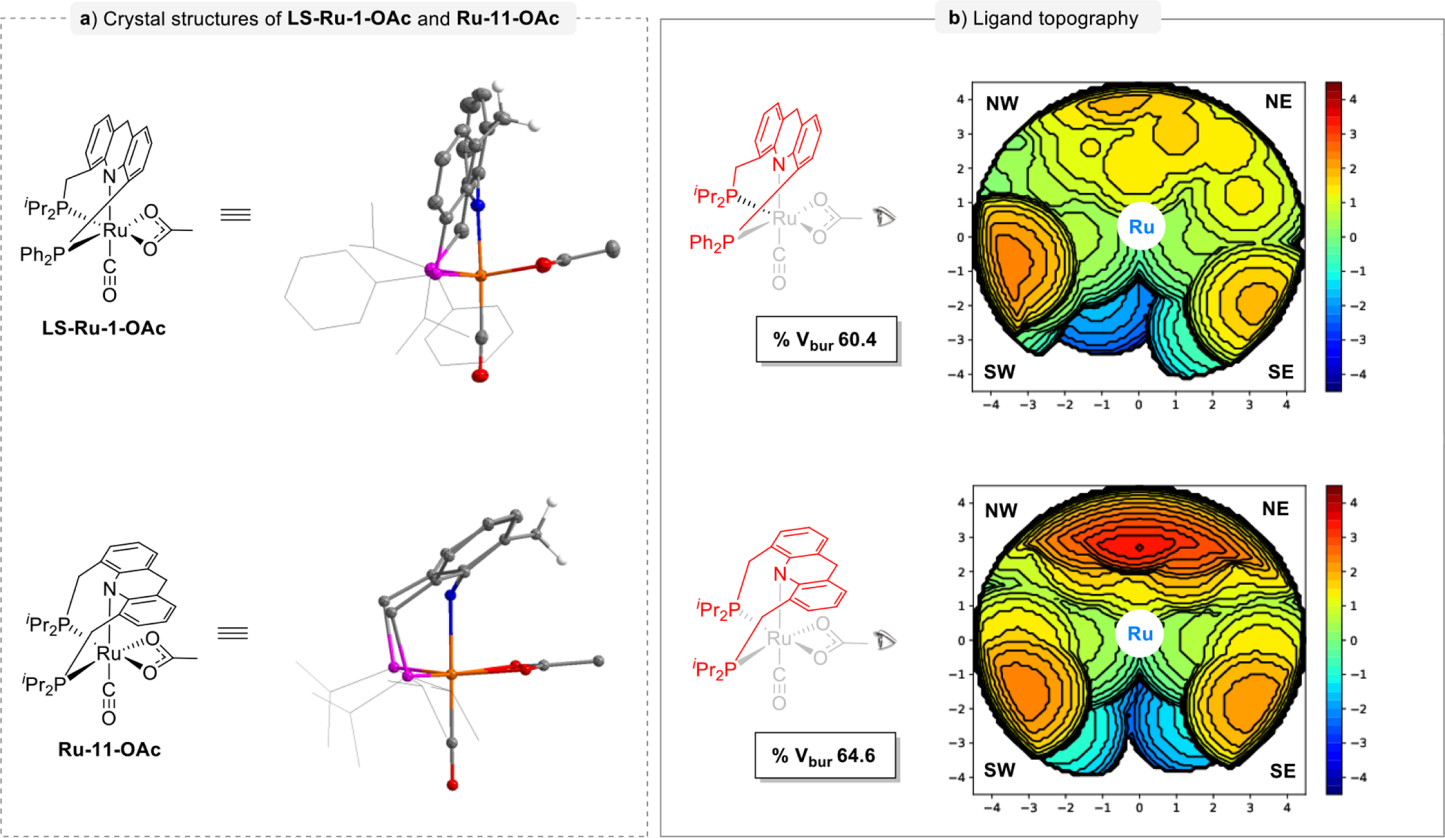
Steric maps based on the crystal structures of **LS-Ru-1-OAc** and **Ru-11-OAc**. Only the red parts of the complexes were considered in the definition of the catalytic pocket. The isocontour scheme, in Å, is shown on the right side of each map. The red and blue zones indicate the more and less-hindered zones in the catalytic pocket, respectively. %*V*_bur_ = percentage of buried volume. A sphere radius of 4.5 Å centered on the Ru atom was used for the %*V*_bur_ calculations.

### Solvent- and Additive-Free Dehydrogenation/Hydrogenation Cycle

Solvent- and additive-free reaction conditions offer several advantages, including optimal gravimetric and volumetric H_2_ densities, potentially decreased reaction times, reduced energy consumption, and lower capital investment. These benefits make such conditions more environmentally benign and cost-effective, thereby making them more attractive for industrial applications. Furthermore, in the current EG system, solvent-free conditions may facilitate polymerization reactions and reduce CO formation due to the higher EG concentration compared with systems with solvents. Consequently, we investigated the performance of these new complexes under neat conditions. First, **LS-Ru-1** and **LS-Ru-3** were tested, but their limited solubility in EG inhibited their performance, resulting in low conversions and low H_2_ yields. To address this solubility issue, we rationally designed the new ligand (**L5**) bearing a poly­(ethylene glycol) (PEG) chain as an EG-solubilizing group and synthesized the corresponding complex **LS-Ru-5** (Figure 9a). To minimize the impact of the appended PEG on the coordination model, steric hindrance, stability, and activity compared to those of the original complex **LS-Ru-1**, we chose to introduce PEG at the *para* position of the phenyl moieties. Encouragingly, **LS-Ru-5** exhibited markedly improved solubility in EG and enabled efficient catalysis under solvent- and additive-free conditions, as summarized in Figure 9b and further elaborated below. The novel pincer complex **LS-Ru-5-Cl** was prepared by reaction of new ligand **L5** with [RuCl_2_(DMSO)_3_(CO)] in toluene at 80 °C for 10 h. The reaction of **LS-Ru-5-Cl** with 2 equiv of NaHBEt_3_ in a THF solution at room temperature for 7 h, followed by the addition of pyridine, gave the novel complex **LS-Ru-5** (Figure 9a). Using 0.5 mol % **LS-Ru-5** as the catalyst, the base-free dehydrogenative coupling of EG was carried out at 150 °C in a 1:1 (v/v) mixture of toluene and 1,2-dimethoxyethane as solvent. Notably, > 99% conversion of EG, 92% H_2_ yield with 98.99% purity, and oligoesters with high degrees of oligomerization (*n* up to 9) were achieved (for details, see Supporting Information), demonstrating performance very similar to that of **LS-Ru-1**. Next, we performed the dehydrogenation reaction of EG on a larger scale (17.8 mmol, 1 mL) under neat conditions at 150 °C and a partial vacuum of 95 mbar. The reduced pressure was employed to maintain reflux in the reaction system, facilitating the efficient removal of generated hydrogen and driving the reaction forward. Under these conditions, a 95% conversion was obtained after 7 days using 0.2 mol % of **LS-Ru-5** (Figure 9b). The remaining EG condensed in the reflux condenser, out of reach of the catalyst. Based on ^1^H NMR spectroscopy of the crude reaction mixture, the hydrogen yield was estimated at 82% (referenced to the maximum HSC of EG, 6.5 wt %), with an average degree of oligomerization of approximately 6, and the realized HSC was 5.6 wt %. To complete the entire cycle, the hydrogenation of the reaction mixture described above back to EG was investigated. Encouragingly, without the need for further addition of catalyst, the above crude reaction mixture could be fully hydrogenated back to EG within 24 h under solvent- and additive-free conditions and 50 bar of hydrogen. Thus, the entire cycle of the current LOHC system has been successfully achieved under solvent- and additive-free conditions for the first time. Notably, this achievement was accomplished by using the same catalyst throughout the process.

**Figure 9:**
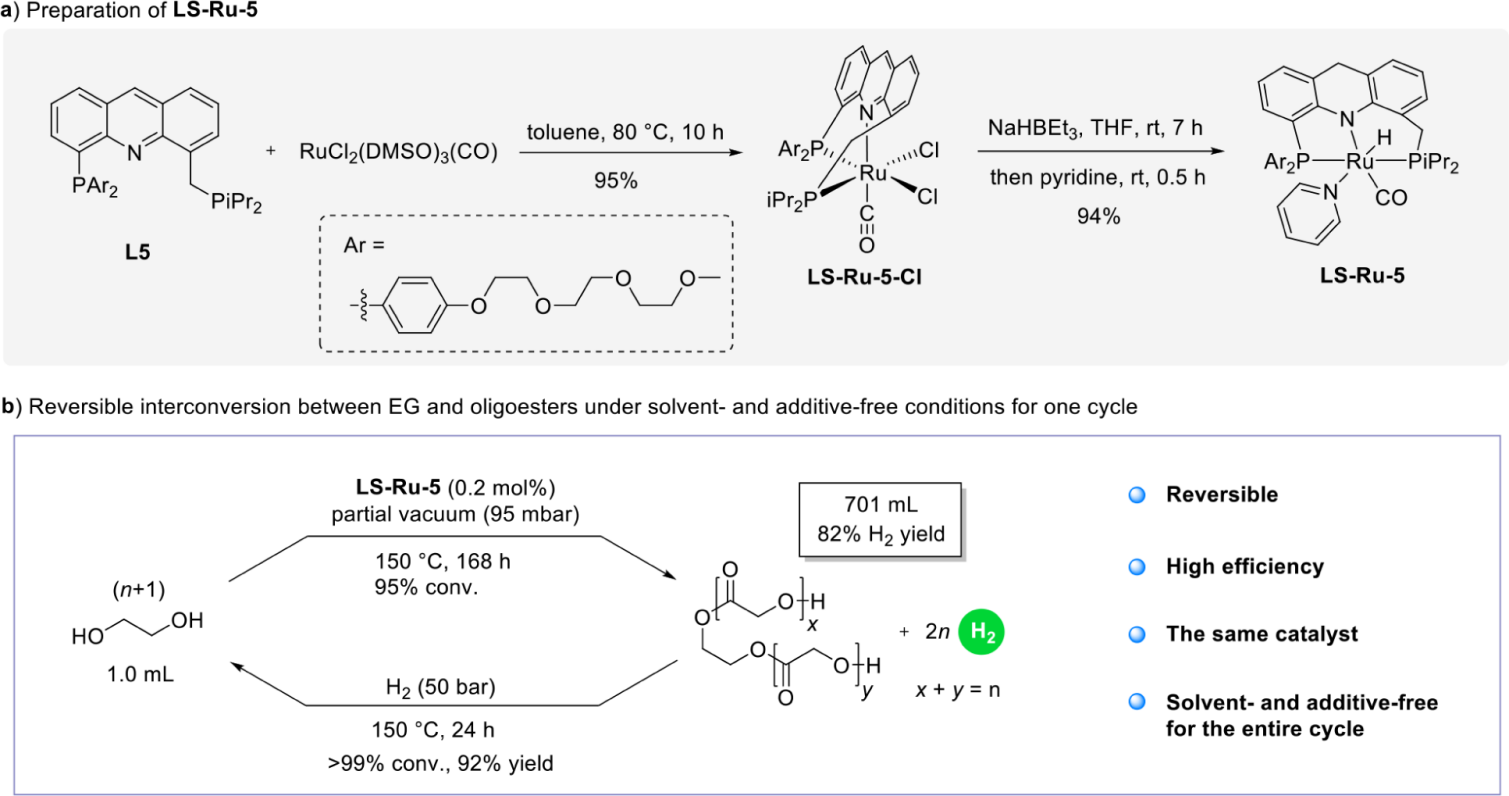
Solvent- and additive-free dehydrogenation/hydrogenation cycle.

## Conclusions

In conclusion, a new class of PNP-pincer ligands, called long–short-arm acridine ligands, and their Ru-complexes have been developed. These new catalysts, **LS-Ru-1** and **LS-Ru-3**, facilitate a highly efficient dehydrogenative coupling of EG, achieving an H_2_ yield of over 90% for the first time. Compared to our previous **Ru-11**, **LS-Ru-1** shows not only higher reactivity in the early-stage dehydrogenative coupling of EG, but also better ability in the late-stage dehydrogenative coupling process, which is very critical for achieving a high H_2_ yield. Mechanistic and computational studies reveal that the unique coordination model based on the current long–short-arm acridine skeleton, featuring one 5-membered and one 6-membered metallacycle, which enables *mer*-*fac* fluxionality, a large catalytic pocket, and reduced steric hindrance around the Ru center, is the key factor in achieving high reactivity, a high degree of oligomerization, and subsequently, a high H_2_ yield. Moreover, by using **LS-Ru-5** as the catalyst, the entire cycle of the current LOHC system, including both dehydrogenation and hydrogenation, has been successfully achieved under solvent- and additive-free conditions for the first time, which further highlights the robustness and application potential of this new catalytic system. The development of novel pincer catalysts and LOHC systems is ongoing in our lab.

## Supplementary Material



## Notes

Experimental details, characterization data, NMR spectra (PDF)
