# Supplementary material for: Long–Short-Arm Acridine Ru-Pincer Catalysts for Reversible Hydrogen Storage Based on Ethylene Glycol
Source: J Am Chem Soc. 2025 Aug 7;147(33):30060–71. doi: 10.1021/jacs.5c07428 (PMC12371887; doi:10.1021/jacs.5c07428)
Supplement: Supplementary file 1 [file ja5c07428_si_001.pdf]

Supporting Information

Long-Short-Arm Acridine Ru-Pincer Catalysts for Reversible Hydrogen Storage Based on Ethylene Glycol

Cai You,[1] Lijun Lu,[1] Jie Luo,[1] Yael Diskin-Posner,[2] David Milstein[1*]

[1]*Department of Molecular Chemistry and Materials Science, The Weizmann Institute of Science, Rehovot 7610001, Israel;*

[2]*Department of Chemical Research Support, The Weizmann Institute of Science, Rehovot 7610001, Israel;*

**e-mail: david.milstein@weizmann.ac.il.*

Table of Contents

1. General information..S2

2. Preparation of **L1-L5**…………………………………………………………………………S3

3. Preparation of Ru-complexes………..S39

4. Base-free dehydrogenative coupling of EG using a mixed solvent…….....................................S93

5. Continuous experiments with **Ru-11**..S106

6. Base-free dehydrogenative coupling of HEG using a mixed solvent......................................S109

7. Solvent- and additive-free dehydrogenation/hydrogenation cycle..S114

8. GC data…..S120

9. Computational studies…...S127

10. References………………………………………………………………………..........S191

1. General information

All reactions were performed under an atmosphere of purified nitrogen in an MBraun glovebox, or by using standard Schlenk techniques unless otherwise noted. All commercially available reagents were used as received unless otherwise mentioned. Ethylene glycol (Acros Organics) was further dried over 4 Å molecular sieves (MS) before using. All solvents were purified according to standard procedures under an argon atmosphere, and stored over 4 Å MS. Deuterated benzene, chloroform, dichloromethane and THF were degassed with nitrogen and stored in the glovebox over 4Å MS. The pincer complex **Ru-11** was prepared according to the literature procedure.[1] Elevated temperatures were maintained using Thermostat-controlled silicone oil baths. Gas chromatography (GC) analysis was performed on an HP 6890 chromatograph, equipped with a thermal conductivity detector (TCD), using helium as the carrier gas. High resolution electrospray ionization mass spectrometry (HR-ESI-MS) was carried out on a Waters Xevo G2-XS QT of mass spectrometer at the Department of Chemical Research Support, Weizmann Institute of Science. NMR spectra were recorded using Bruker Avance NEO 300 MHz, Avance NEO 400 MHz, or Avance III HD 500 MHz spectrometers at 293 K. ^{1}H NMR chemical shifts are referenced to the residual hydrogen signal of the deuterated solvent, and the ^{13}C NMR chemical shifts are referenced to the ^{13}C signal of the deuterated solvent. ^{31}P NMR chemical shifts are referenced with respect to an external solution of 85% phosphoric acid in D_2O. Abbreviations used in the description of NMR data are as follows: br, broad; s, singlet; d, doublet; t, triplet; q, quartet; m, multiplet. Infrared (IR) spectra were recorded on a Thermo Nicolet 6700 FT-IR spectrometer. Analytical TLC was performed on Merck silica gel 60 F254 plates. Flash chromatography columns were packed with 200-300 mesh silica gel.

2. Preparation of L1-L5

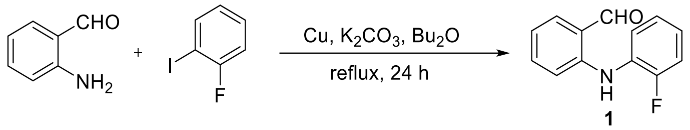

1

Synthesis of 2-((2-fluorophenyl)amino)benzaldehyde (1):

The aldehyde **1** was prepared according to a literature procedure.[2] Under an N_2 atmosphere, 2-aminobenzaldehyde (146.6 mmol), 1-fluoro-2-iodobenzene (146.6 mmol), K_2CO_3 (99.7 mmol), and Cu (23.5 mmol) were dissolved in Bu_2O (16.0 mL) and heated to reflux for 24 h. The reaction was then filtered and concentrated in vacuo. The crude product was purified via flash chromatography (hexane/ethyl acetate = 100:1 to 30:1) to give **1** as a yellow solid (33.5 g, 77% yield).

^{1}H NMR (300 MHz, $CDCl_3$) δ 9.93 (s, 1H), 9.90 (s, 1H), 7.59 (dd, J = 7.7, 1.3 Hz, 1H), 7.48 – 7.34 (m, 2H), 7.21 – 7.10 (m, 3H), 7.07 (d, J = 8.6 Hz, 1H), 6.88 (t, J = 7.4 Hz, 1H).

^{13}C NMR (75 MHz, $CDCl_3$) δ 194.47, 156.22 (d, J = 247.2 Hz), 147.29, 136.58, 135.63, 125.57 (d, J = 7.6 Hz), 124.85 (d, J = 1.4 Hz), 124.51 (d, J = 3.9 Hz), 119.96, 117.82, 116.53 (d, J = 19.8 Hz), 113.17 (d, J = 1.2 Hz).

^{19}F NMR (282 MHz, $CDCl_3$) δ -124.64 (s).

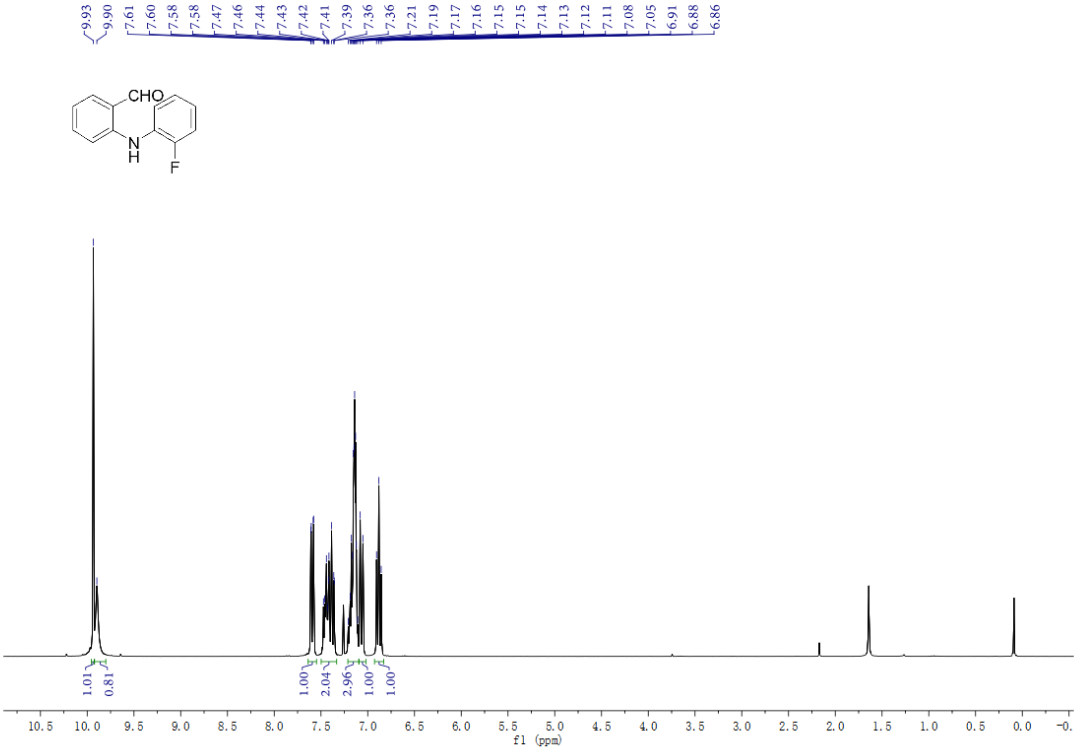

Figure S1. ^{1}H NMR (300 MHz, $CDCl_3$) spectrum of **1**

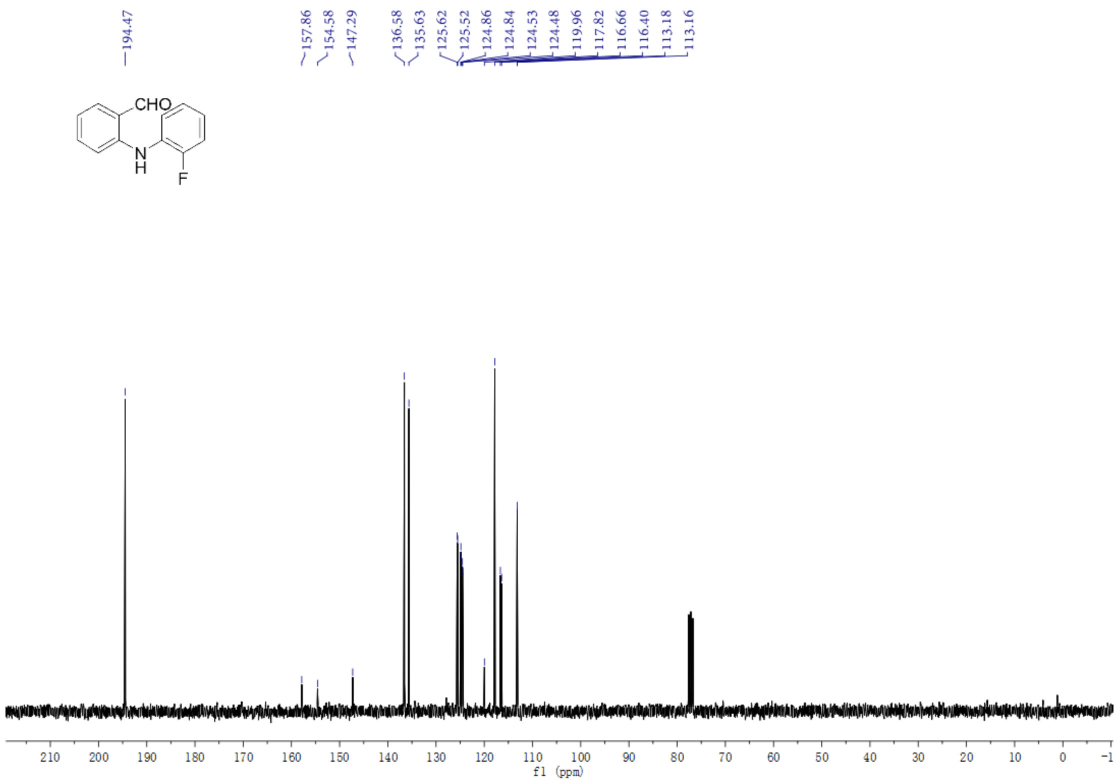

Figure S2. ^{13}C NMR (75 MHz, $CDCl_3$) spectrum of **1**

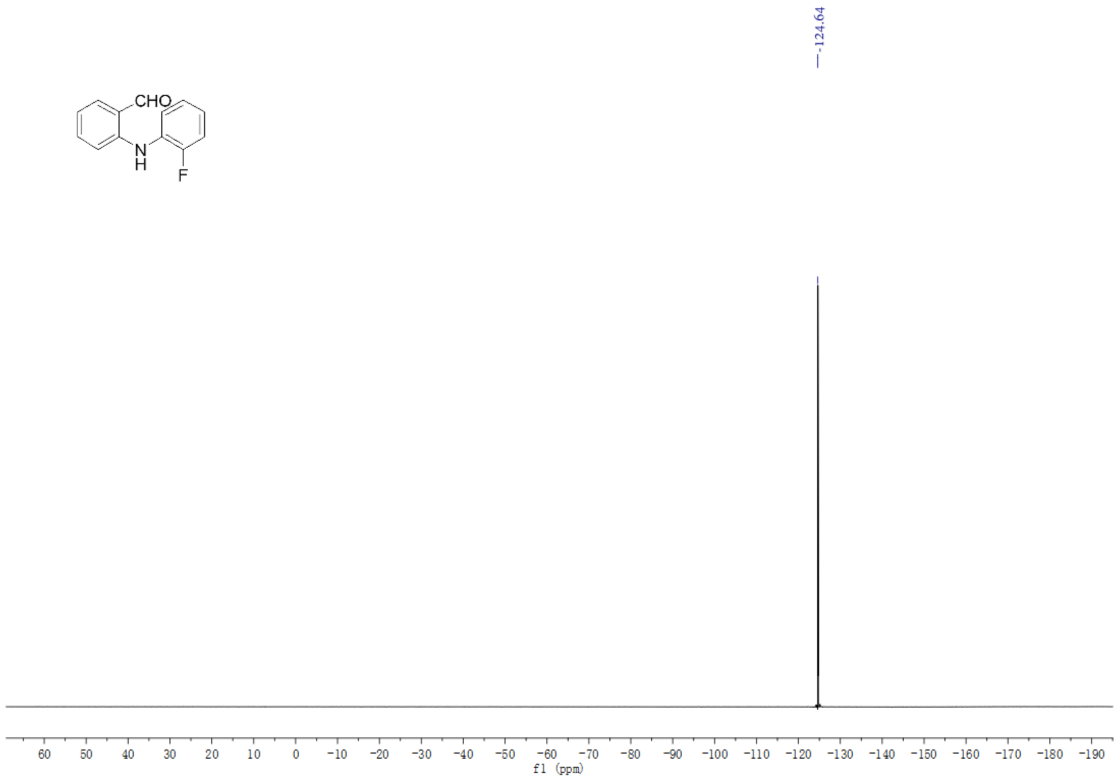

Figure S3. ^{19}F NMR (282 MHz, $CDCl_3$) spectrum of **1**

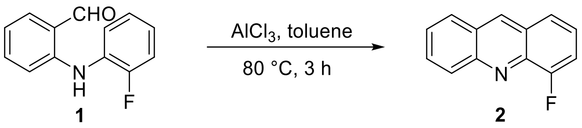

Synthesis of 4-fluoroacridine (2):

4-Fluoroacridine **2** was prepared according to a literature procedure.[3] $AlCl_3$ (104.0 mmol) was added into a solution of 2-((2-fluorophenyl)amino)benzaldehyde **1** (52.0 mmol) in toluene (208.0 mL) and the mixture was heated at 80 °C for 3 h. After the reaction was cooled to room temperature, it was poured onto crushed ice and stirred slowly for 1 h. The mixture was extracted with dichloromethane and the combined organic phase was dried over Na_2SO_4, filtered and concentrated. The crude product was purified by flash chromatography (hexane/ethyl acetate = 20:1 to 5:1) to give **2** as a yellow solid (9.2 g, 90% yield).

^{1}H NMR (400 MHz, $CDCl_3$) δ 8.73 (s, 1H), 8.33 (d, J = 8.9 Hz, 1H), 7.95 (d, J = 8.5 Hz, 1H), 7.81 – 7.70 (m, 2H), 7.56 – 7.50 (m, 1H), 7.45 – 7.36 (m, 2H).

^{13}C NMR (101 MHz, $CDCl_3$) δ 157.88 (d, J = 258.0 Hz), 148.93, 140.19 (d, J = 12.8 Hz), 136.07 (d, J = 3.4 Hz), 130.91, 129.97, 128.20, 127.96 (d, J = 2.3 Hz), 127.02, 126.54, 124.95 (d, J = 7.7 Hz), 124.08 (d, J = 5.1 Hz), 113.01 (d, J = 19.1 Hz).

^{19}F NMR (282 MHz, $CDCl_3$) δ -125.80.

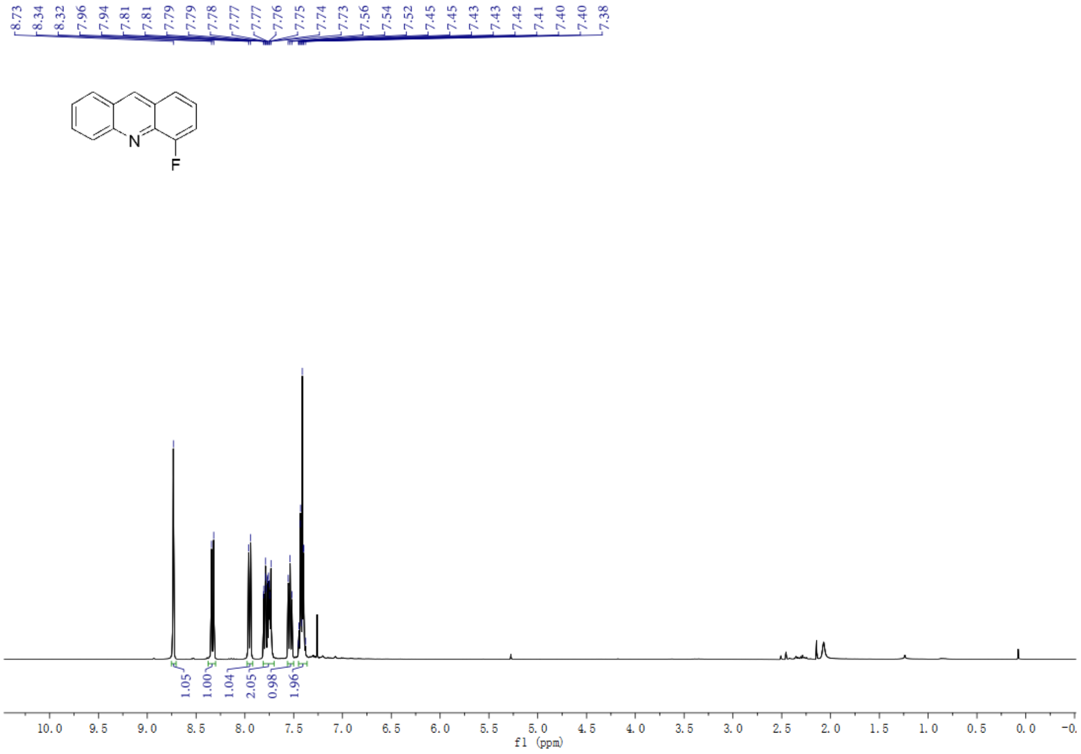

Figure S4. ^{1}H NMR (400 MHz, $CDCl_3$) spectrum of **2**

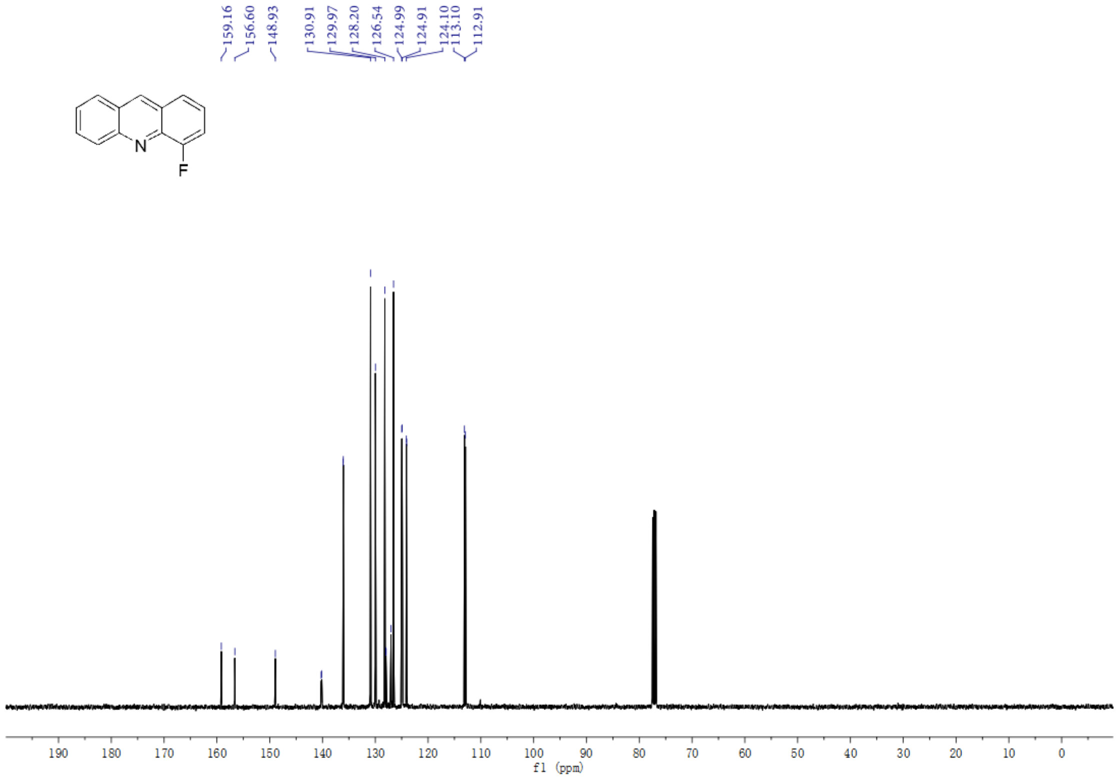

Figure S5. ^{13}C NMR (101 MHz, $CDCl_3$) spectrum of **2**

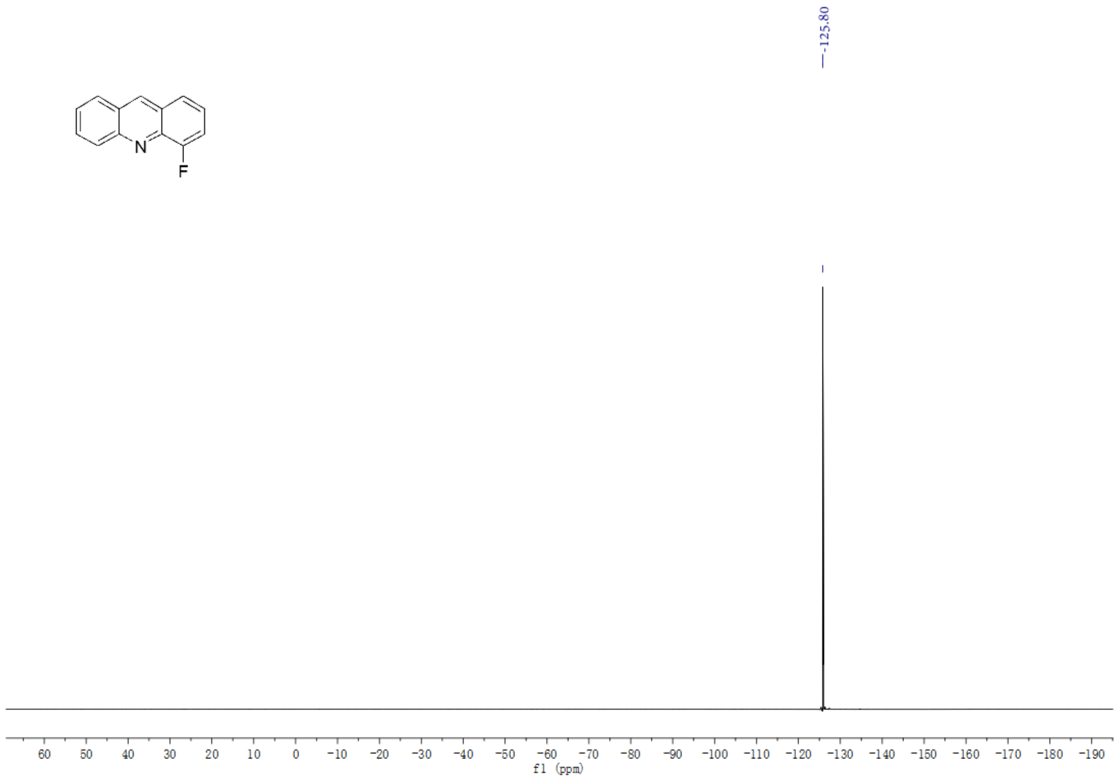

Figure S6. ^{19}F NMR (282 MHz, $CDCl_3$) spectrum of **2**

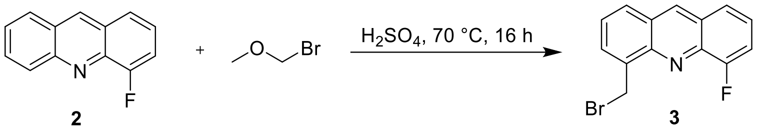

Synthesis of 4-(bromomethyl)-5-fluoroacridine (3):

A mixture of 4-fluoroacridine **2** (10.0 mmol) and concentrated H_2SO_4 (98%, 20.0 mL) was stirred under argon at 70 °C and bromomethyl methyl ether (20.0 mmol) was added to it in one portion. The mixture was maintained under nitrogen for 16 h at 70 °C and cooled to room temperature before slowly transferred to a beaker containing crushed ice and dichloromethane. The mixture was extracted with dichloromethane and the combined organic phase was dried over Na_2SO_4, filtered and concentrated. The crude product was purified by flash chromatography (hexane/ dichloromethane = 4:1) to give **3** as a yellow solid (1.14 g, 39% yield).

^{1}H NMR (500 MHz, $CDCl_3$) δ 8.80 (s, 1H), 8.01 – 7.95 (m, 2H), 7.83 – 7.77 (m, 1H), 7.55 (t, J = 7.7 Hz, 1H), 7.51 – 7.44 (m, 2H), 5.44 (s, 2H). (7.26 ppm)

^{13}C NMR (126 MHz, $CDCl_3$) δ 158.32 (d, J = 259.4 Hz), 146.33, 139.84 (d, J = 12.9 Hz), 136.83, 136.22 (d, J = 3.3 Hz), 131.76, 129.12, 128.23 (d, J = 1.7 Hz), 127.09, 126.33, 125.52 (d, J = 7.6 Hz), 123.86 (d, J = 5.2 Hz), 113.31 (d, J = 18.8 Hz), 29.83. (77.16 ppm)

^{19}F NMR (471 MHz, $CDCl_3$) δ -124.99.

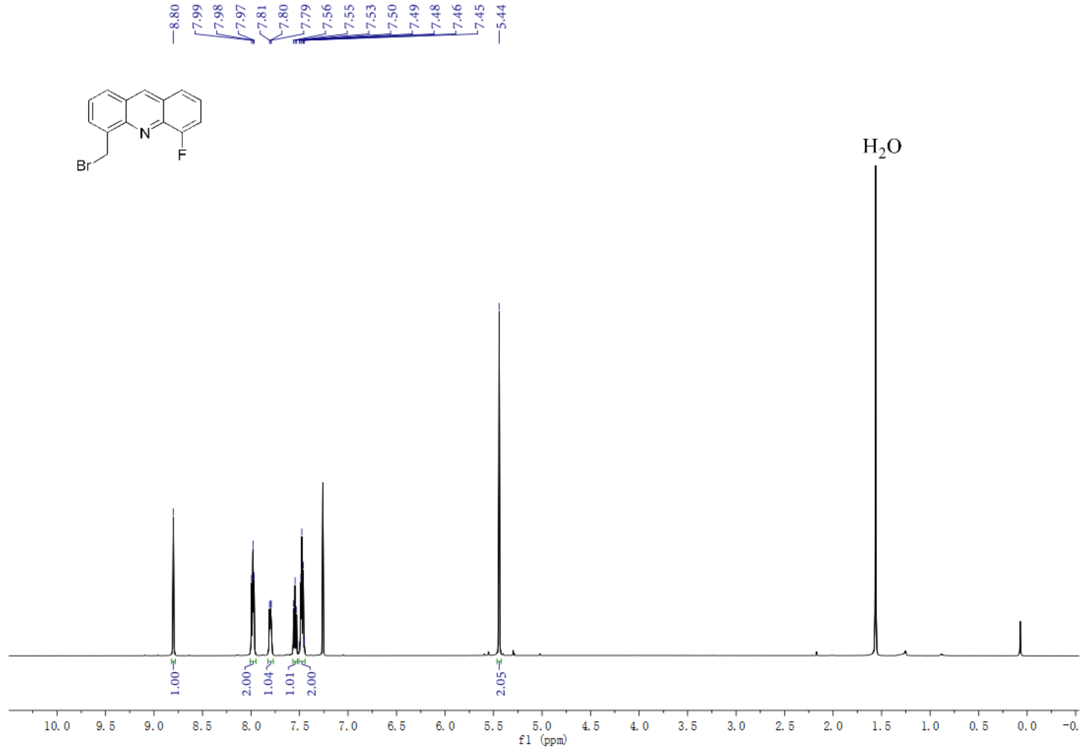

Figure S7. ^{1}H NMR (500 MHz, $CDCl_3$) spectrum of **3**

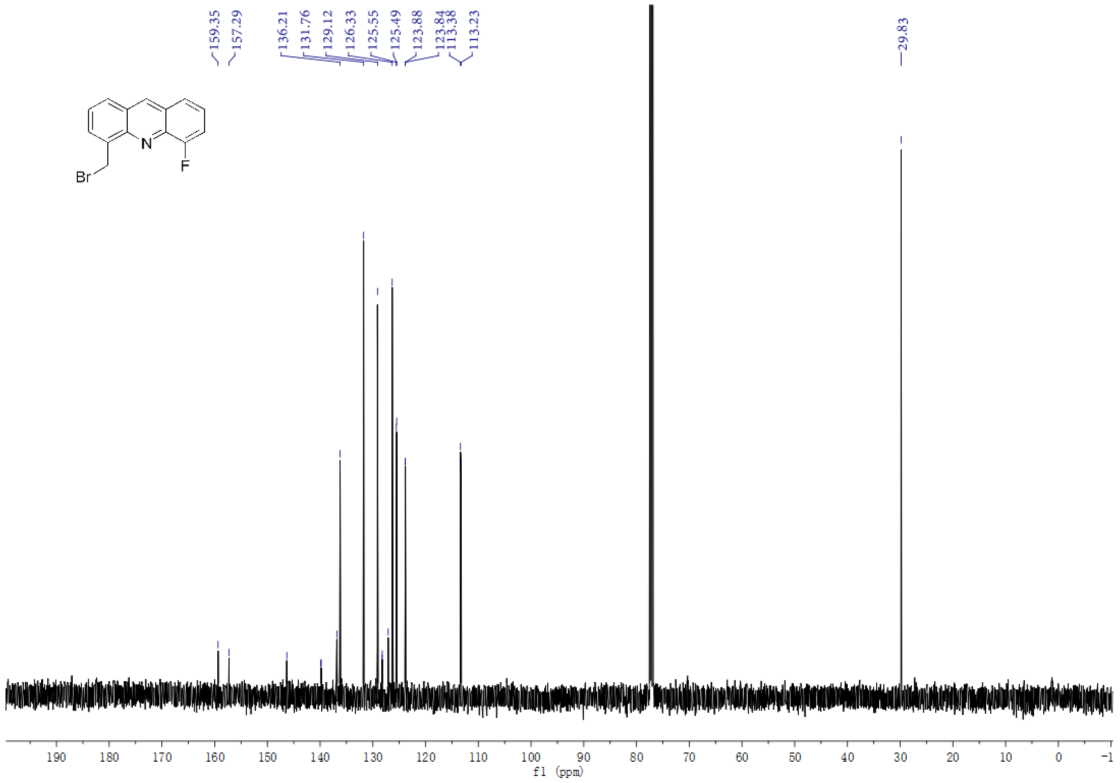

Figure S8. ^{13}C NMR (126 MHz, $CDCl_3$) spectrum of **3**

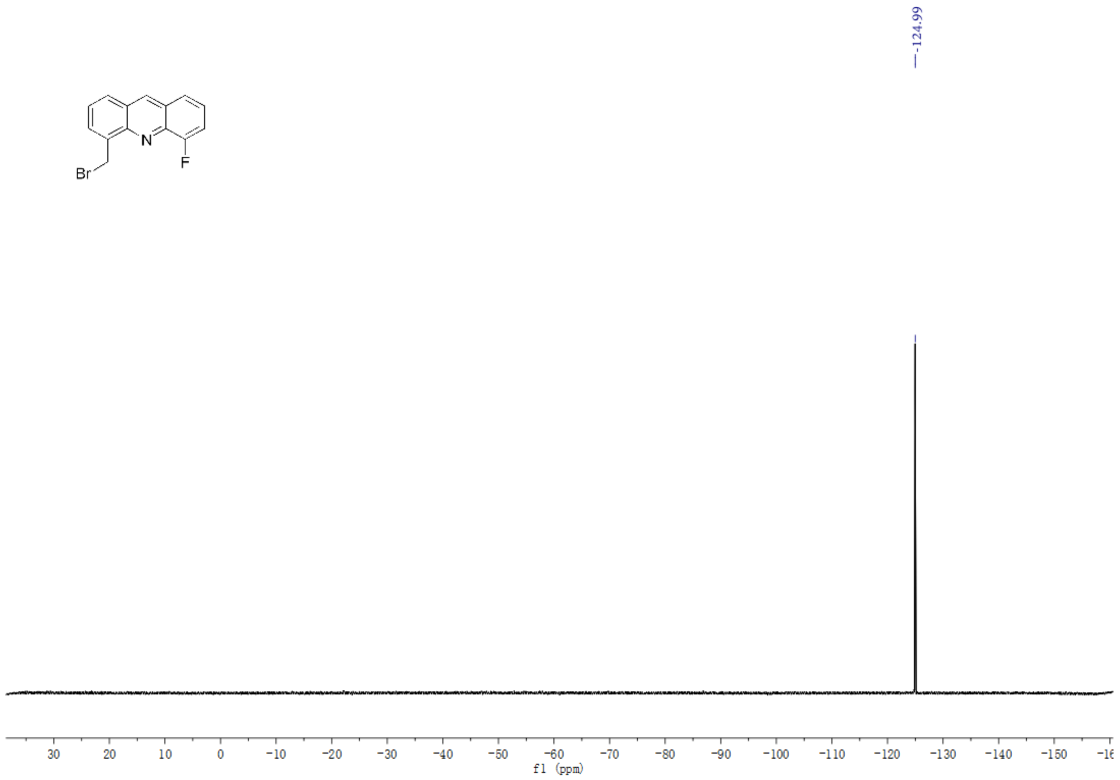

Figure S9. ^{19}F NMR (471 MHz, $CDCl_3$) spectrum of **3**

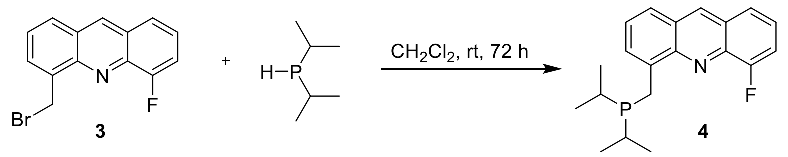

Synthesis of 4-((diisopropylphosphanyl)methyl)-5-fluoroacridine (4):

In a glovebox, 4-(bromomethyl)-5-fluoroacridine **3** (748.0 mg, 2.58 mmol) was dissolved in dichloromethane (50 mL) in an oven-dried 100 mL round-bottom flask equipped with a magnetic stirring bar. Then diisopropylphosphane (457.4 mg, 3.87 mmol) was added and stirred at room temperature for 72 h. Triethylamine (1.31 g, 12.9 mmol) was added, and the resulting solution was stirred at room temperature for 1 hour. The solvent was removed under vacuum and the residue was extracted with diethyl ether. Subsequently, the diethyl ether solution was filtered through Celite and then removed under vacuum to yield **4** as a yellow solid (770.0 mg, 91% yield).

^{1}H NMR (400 MHz, $CDCl_3$) δ 8.74 (s, 1H), 7.82 (d, J = 7.7 Hz, 2H), 7.79 – 7.74 (m, 1H), 7.49 (t, J = 7.7 Hz, 1H), 7.41 (pd, J = 7.4, 3.3 Hz, 2H), 3.68 (d, J = 3.5 Hz, 2H), 1.93 (dtd, J = 14.2, 7.1, 2.0 Hz, 2H), 1.17 (ddd, J = 19.9, 12.3, 7.1 Hz, 12H).

^{13}C NMR (126 MHz, $CDCl_3$) δ 158.56 (d, J = 259.3 Hz), 147.66 (s), 140.60 (d, J = 8.2 Hz), 139.37 (d, J = 12.9 Hz), 135.99 (d, J = 3.3 Hz), 130.41 (d, J = 9.3 Hz), 128.01 (d, J = 2.1 Hz), 127.37 (s), 126.42 (s), 125.90 (d, J = 2.2 Hz), 124.95 (d, J = 7.6 Hz), 123.75 (d, J = 5.1 Hz), 112.58 (d, J = 18.8 Hz), 24.42 (d, J = 18.9 Hz), 24.08 (d, J = 14.6 Hz), 19.80 (d, J = 13.0 Hz), 19.63 (d, J = 12.0 Hz).

^{31}P NMR (121 MHz, $CDCl_3$) δ 18.12.

^{19}F NMR (377 MHz, $CDCl_3$) δ -143.31.

HRMS (ESI): Exact mass calculated for $C_{20}H_{24}FNP^+$ ($[M+H]^+$): 328.1625, mass found: 328.1626.

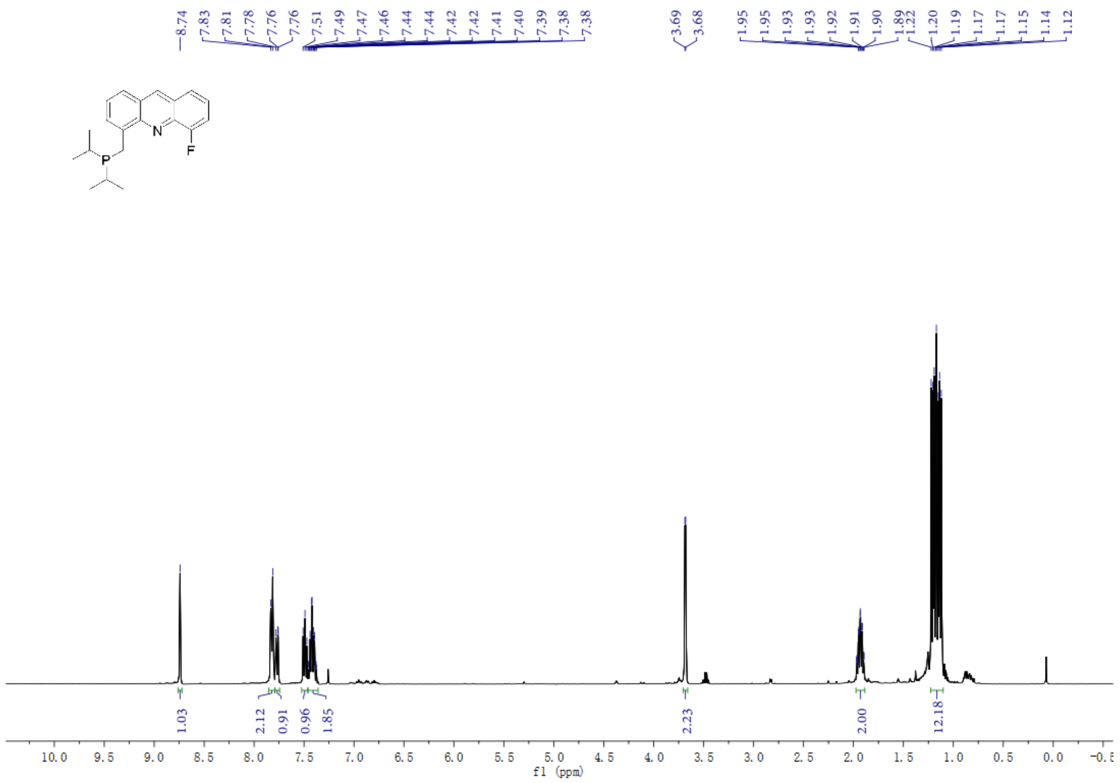

Figure S10. ^{1}H NMR (400 MHz, $CDCl_3$) spectrum of **4**

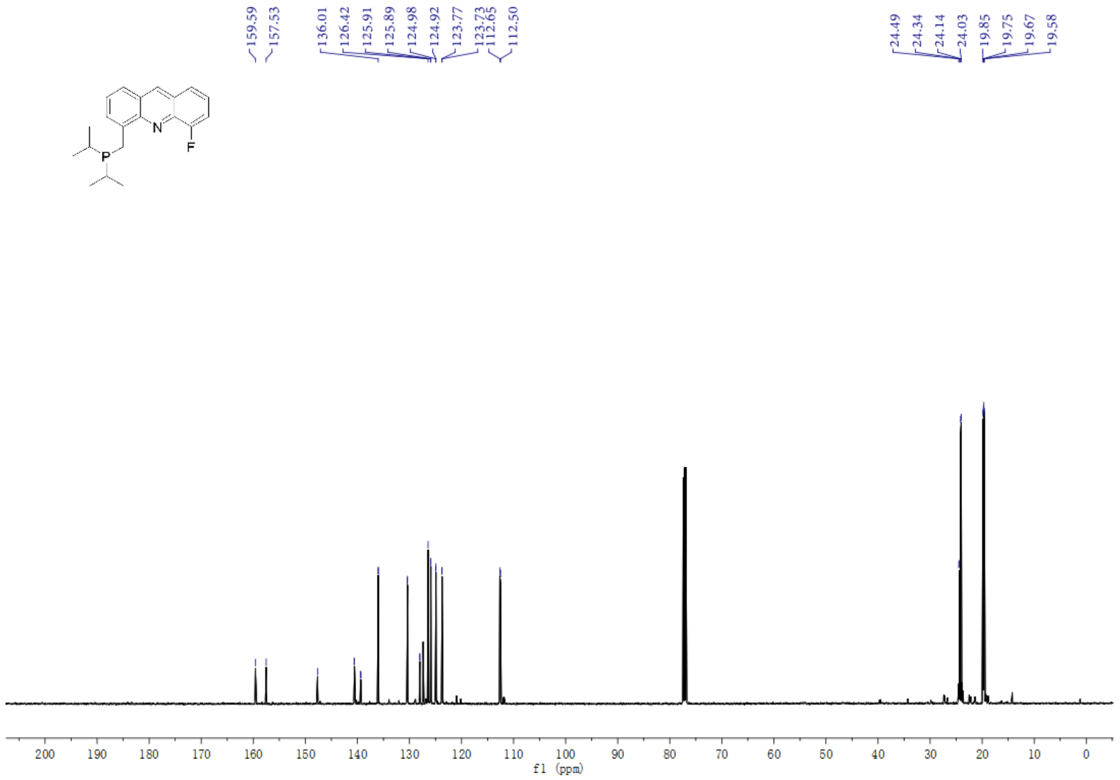

Figure S11. ^{13}C NMR (126 MHz, $CDCl_3$) spectrum of **4**

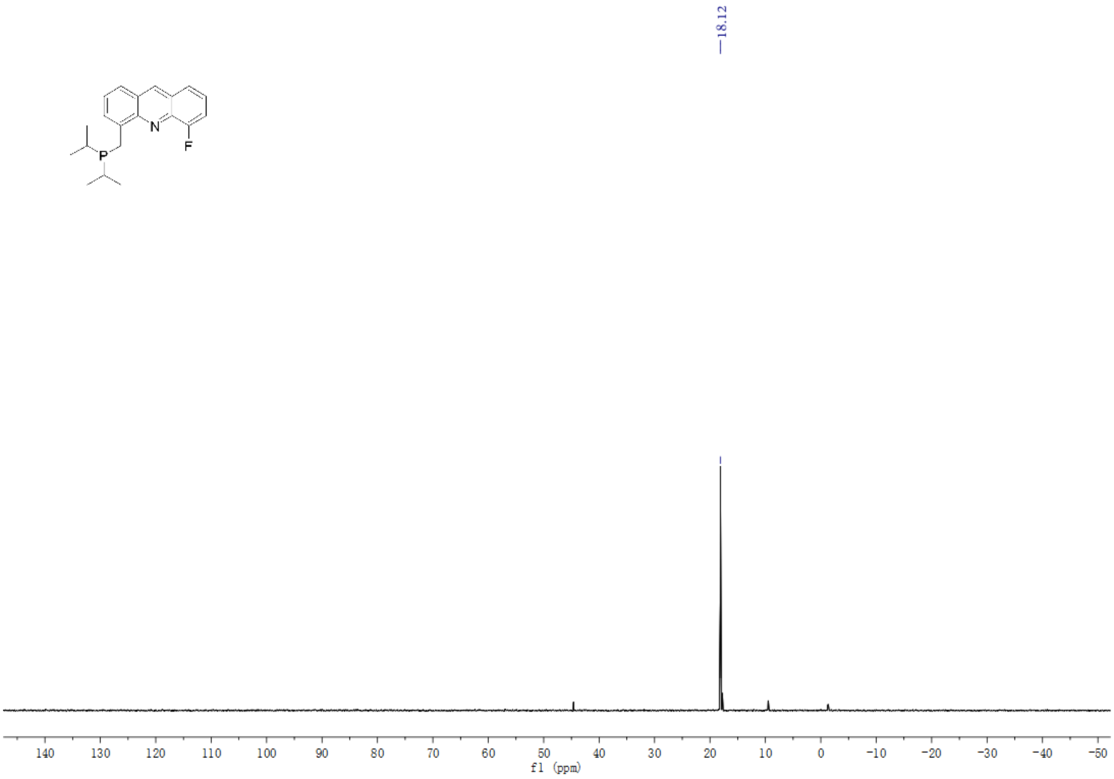

Figure S12. ^{31}P NMR (121 MHz, $CDCl_3$) spectrum of **4**

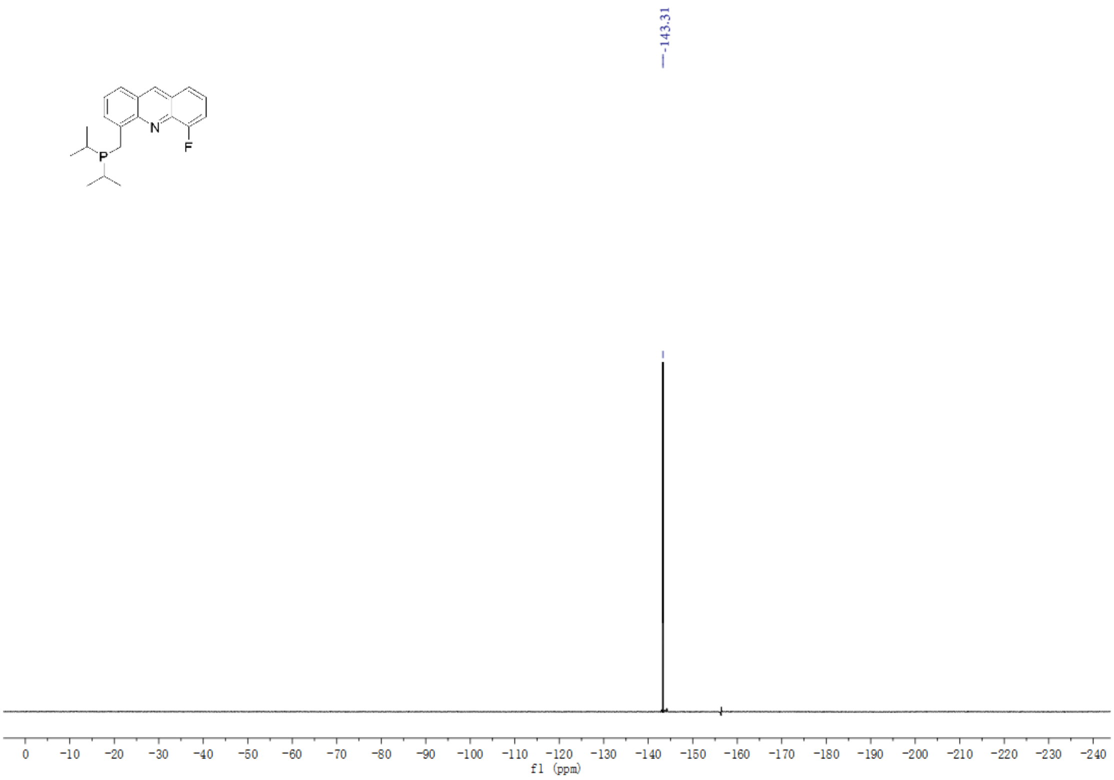

Figure S13. ^{19}F NMR (377 MHz, $CDCl_3$) spectrum of **4**

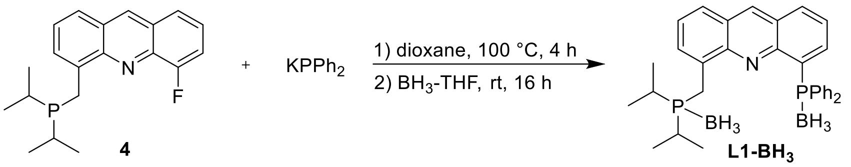

Synthesis of L1-BH$_3$:

In a glovebox, to a solution of 4-((diisopropylphosphanyl)methyl)-5-fluoroacridine **4** (1.42 g, 4.34 mmol) in dioxane (86 mL) was added dropwise 8.7 mL (4.34 mmol) of 0.5 M KPPh$_2$ in THF. The reaction tube was taken out of the glovebox and heated at 100 °C for 4 h, and cooled to room temperature. Then, BH$_3$-THF complex (13.0 mL, 13.0 mmol, 1M) was added and the mixture was stirred for 16 h at room temperature under nitrogen protected conditions. The resulting solution was concentrated in *vacuo*. The crude product was purified by flash chromatography (hexane/dichloromethane = 4:1 to 2:1) to give **L1-BH$_3$** as a yellow solid (1.31 g, 58% yield).

^{1}H NMR (500 MHz, CDCl$_3$) δ 8.79 (s, 1H), 8.28 (d, *J* = 5.8 Hz, 1H), 8.15 (d, *J* = 7.8 Hz, 1H), 7.84 (d, *J* = 7.9 Hz, 1H), 7.63 (t, *J* = 8.6 Hz, 4H), 7.51 – 7.45 (m, 3H), 7.44 – 7.34 (m, 6H), 3.66 (d, *J* = 12.5 Hz, 2H), 1.85 – 1.75 (m, 2H), 1.96 – 1.46 (broad, 3H, BH$_3$), 1.00 (dd, *J* = 14.0, 6.7 Hz, 6H), 0.86 (dd, *J* = 13.3, 6.8 Hz, 6H), 0.82 – 0.25 (broad, 3H, BH$_3$).

^{13}C NMR (126 MHz, CDCl$_3$) δ 147.72 (d, *J* = 6.8 Hz), 147.10 (d, *J* = 5.4 Hz), 138.89 (d, *J* = 5.5 Hz), 137.45, 133.60 (d, *J* = 2.3 Hz), 133.48, 133.40, 132.70, 132.68, 132.36 (d, *J* = 4.4 Hz), 130.95, 130.94, 130.76, 130.29, 129.44, 128.98, 128.78, 128.70, 126.96, 126.67, 126.40 (d, *J* = 5.4 Hz), 126.24, 124.99 (d, *J* = 10.3 Hz), 22.81 (d, *J* = 33.3 Hz), 19.82 (d, *J* = 31.1 Hz), 17.38.

^{31}P NMR (202 MHz, CDCl$_3$) δ 37.23 – 35.91 (broad), 23.08 – 21.62 (broad).

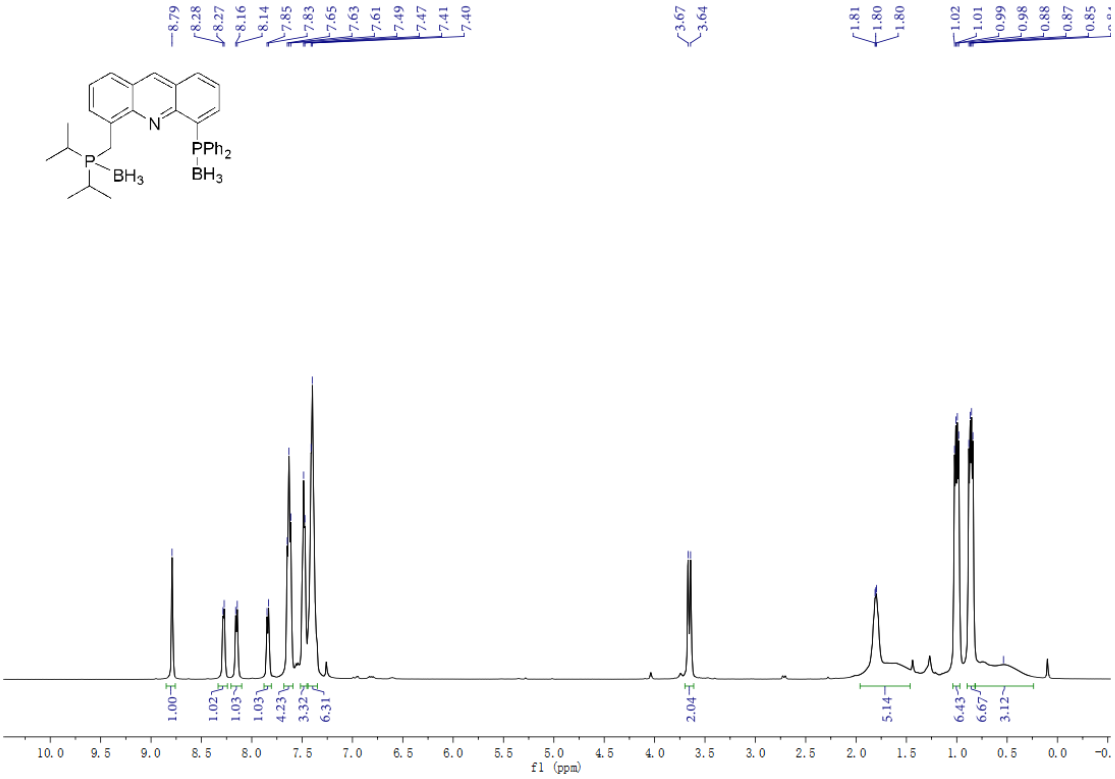

Figure S14. ^{1}H NMR (500 MHz, $CDCl_3$) spectrum of **L1-BH₃**

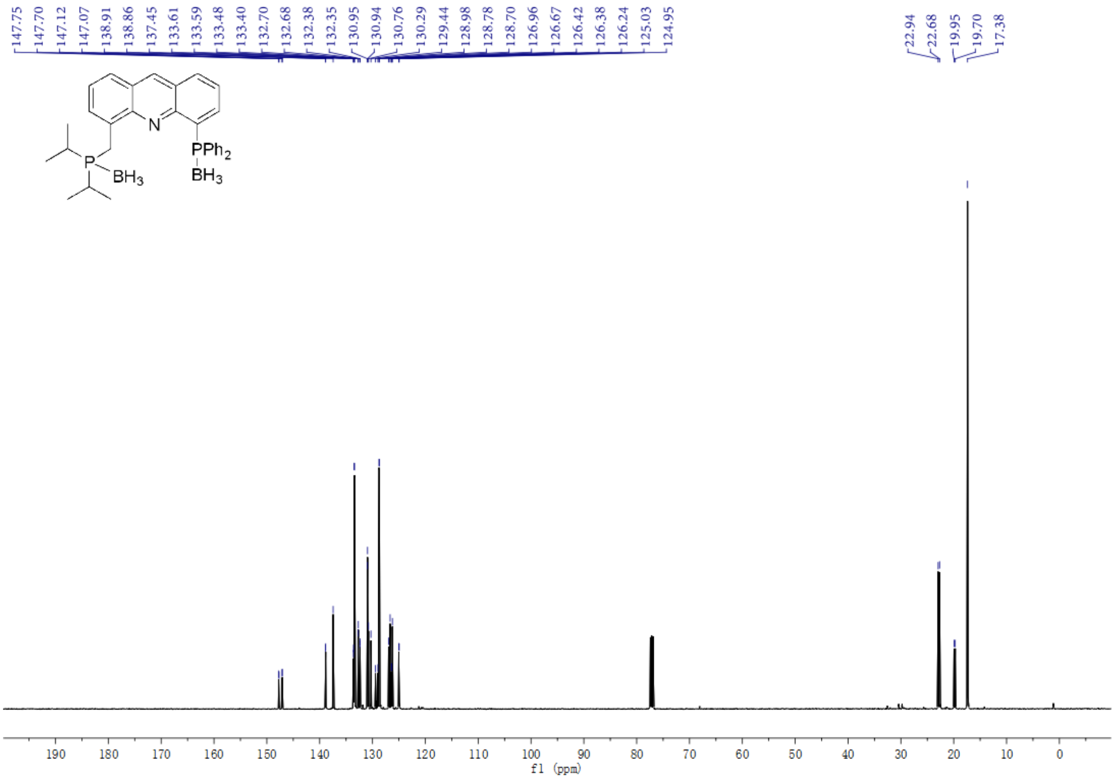

Figure S15. ^{13}C NMR (126 MHz, $CDCl_3$) spectrum of **L1-BH₃**

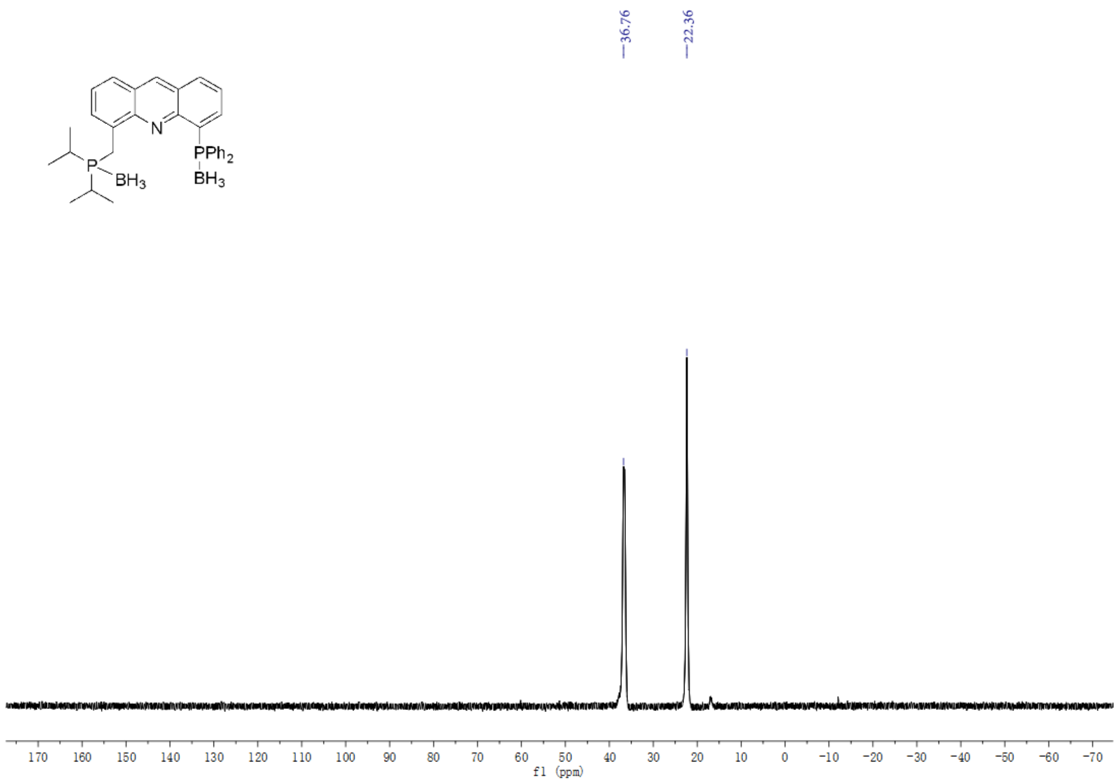

Figure S16. ^{31}P NMR (202 MHz, $CDCl_3$) spectrum of **L1-BH$_3$**

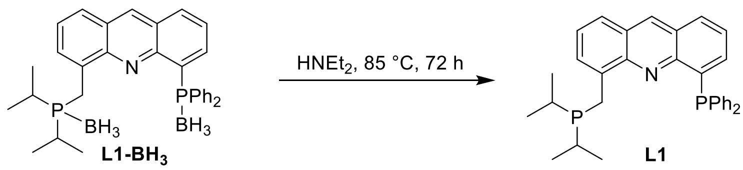

Synthesis of L1:

In a glovebox, **L1-BH$_3$** (1.54 g, 3.04 mmol) and $HNEt_2$ (40 mL) were added into a 100 mL Schlenk tube equipped with a magnetic stirring bar. The reaction tube was taken out of the glovebox and heated at 85 °C for 72 h, cooled to room temperature and taken back into the glovebox. The solvent was removed under vacuum and the residue was extracted with diethyl ether. The diethyl ether solution was filtered through Celite and then removed under vacuum. The resulting yellow solid was washed with a small amount of methanol to obtain the pure ligand **L1** (1.39 g, 93% yield).

^{1}H NMR (300 MHz, $CDCl_3$) δ 8.73 (s, 1H), 7.96 (dd, J = 10.7, 4.8 Hz, 2H), 7.79 (d, J = 8.3 Hz, 1H), 7.46 (dd, J = 8.2, 7.2 Hz, 1H), 7.42 – 7.27 (m, 11H), 7.12 (ddd, J = 6.8, 3.7, 1.2 Hz, 1H), 3.38 (d, J = 3.4 Hz, 2H), 1.73 – 1.57 (m, 2H), 1.02 (dd, J = 13.6, 7.1 Hz, 6H), 0.83 (dd, J = 11.2, 7.0 Hz,

6H).

^{13}C NMR (126 MHz, $CDCl_3$) δ 148.90 (d, *J* = 16.2 Hz), 147.23, 140.20, 140.13, 139.49 (d, *J* = 12.1 Hz), 138.11 (d, *J* = 11.2 Hz), 136.45, 136.44, 134.94, 134.54, 134.38, 130.46, 130.33, 128.85, 128.52, 128.48, 128.42, 126.97, 126.06, 125.97, 125.82, 125.40, 125.38, 24.10 (d, *J* = 12.6 Hz), 22.21 (d, *J* = 16.5 Hz), 20.09 (d, *J* = 14.6 Hz), 19.39 (d, *J* = 9.9 Hz).

^{31}P NMR (121 MHz, $CDCl_3$) δ 13.01, -12.32.

HRMS (ESI): Exact mass calculated for $C_{32}H_{34}NP_2^+$ ([M+H]$^+$): 494.2161, mass found: 494.2162.

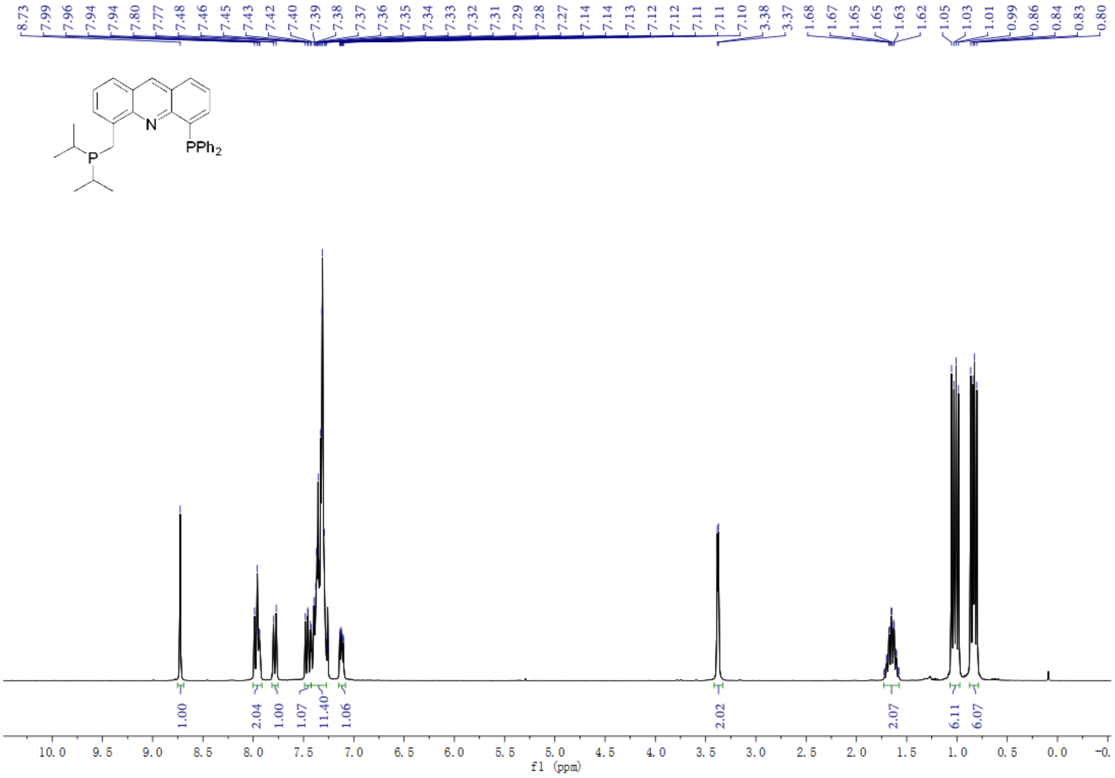

Figure S17. ^{1}H NMR (300 MHz, $CDCl_3$) spectrum of **L1**

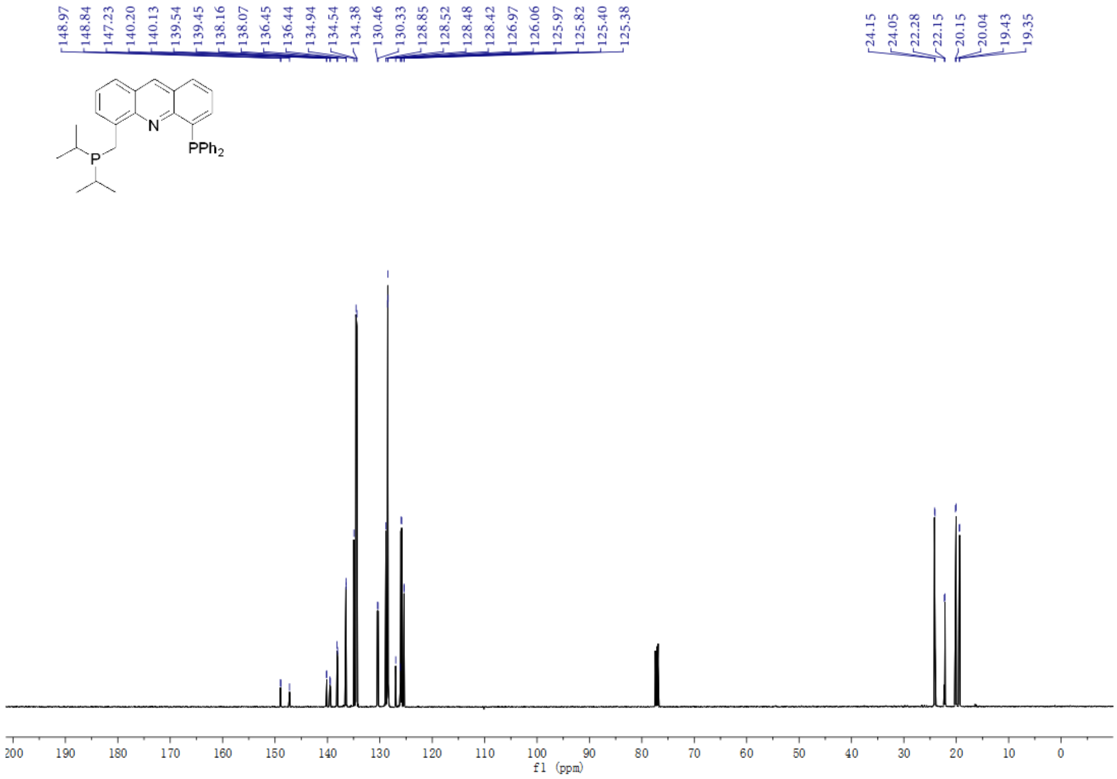

Figure S18. ^{13}C NMR (126 MHz, $CDCl_3$) spectrum of **L1**

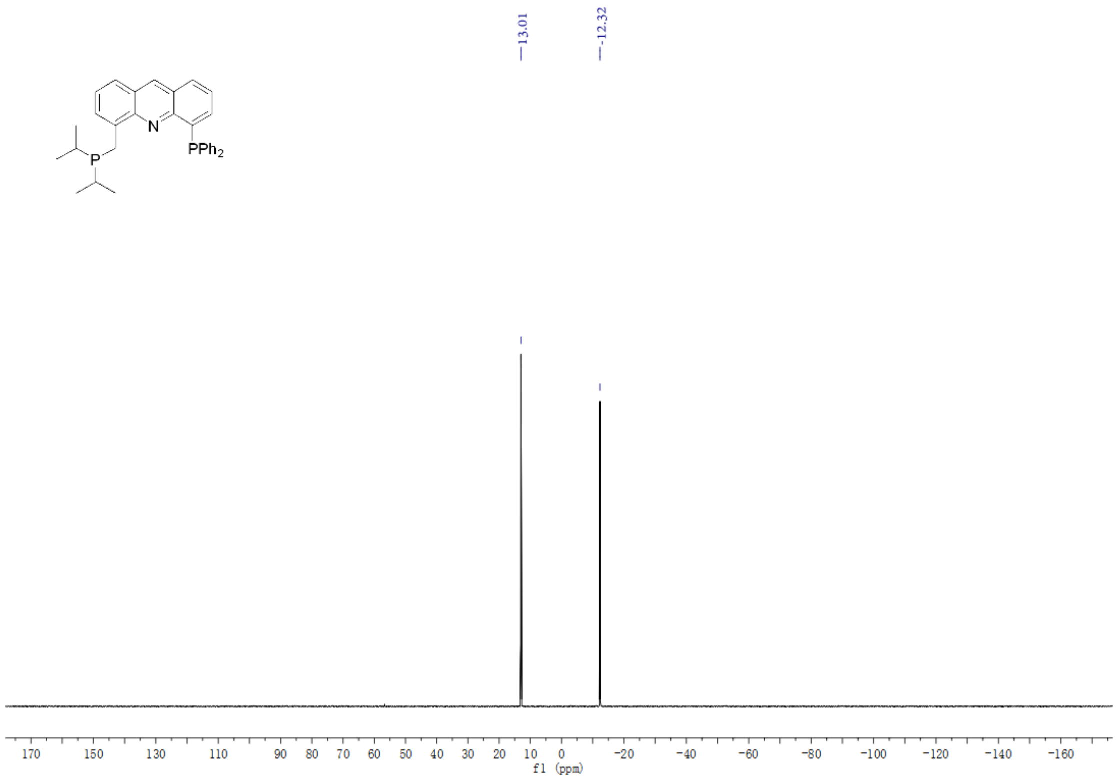

Figure S19. ^{31}P NMR (121 MHz, $CDCl_3$) spectrum of **L1**

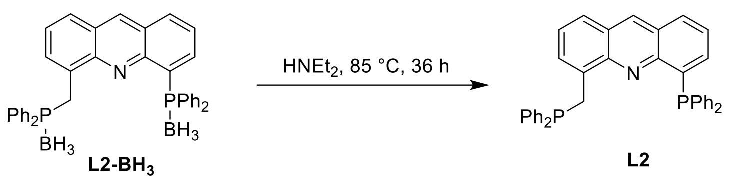

Synthesis of L2:

In a glovebox, **L2-BH$_3$** (140.0 mg, 0.24 mmol) and $HNEt_2$ (10 mL) were added into a 100 mL Schlenk tube equipped with a magnetic stirring bar. The reaction tube was taken out of the glovebox and heated at 85 °C for 36 h, cooled to room temperature and taken back into the glovebox. The solvent was removed under vacuum and the residue was extracted with toluene. The toluene solution was filtered through Celite and then removed under vacuum. The resulting yellow solid was washed with a small amount of methanol to obtain the pure ligand **L2** (121.2 mg, 90% yield).

^{1}H NMR (300 MHz, $CDCl_3$) δ 8.72 (s, 1H), 7.99 (d, *J* = 8.4 Hz, 1H), 7.81 – 7.75 (m, 1H), 7.52 – 7.46 (m, 4H), 7.45 – 7.39 (m, 1H), 7.36 – 7.31 (m, 9H), 7.30 – 7.27 (m, 4H), 7.25 – 7.15 (m, 6H), 3.90 (d, *J* = 1.3 Hz, 2H).

^{13}C NMR (75 MHz, $CDCl_3$) δ 148.79 (d, *J* = 16.0 Hz), 146.73 (dd, *J* = 3.3, 1.8 Hz), 140.04, 139.87, 139.61, 139.40, 137.92, 137.78, 137.09, 136.99, 136.31 (d, *J* = 1.5 Hz), 134.66, 134.39, 133.59, 133.34, 129.79, 129.63, 128.69, 128.49, 128.47, 128.37, 128.30, 128.21, 126.89, 126.22 (d, *J* = 1.4 Hz), 126.16 (d, *J* = 2.5 Hz), 125.90, 125.64 (d, *J* = 1.3 Hz), 29.98 (d, *J* = 14.0 Hz).

^{31}P NMR (121 MHz, $CDCl_3$) δ -11.34 (d, *J* = 6.5 Hz), -12.66 (d, *J* = 6.5 Hz).

HRMS (ESI): Exact mass calculated for $C_{38}H_{30}NP_2^+$ ([M+H]$^+$): 562.1848, mass found: 562.1854.

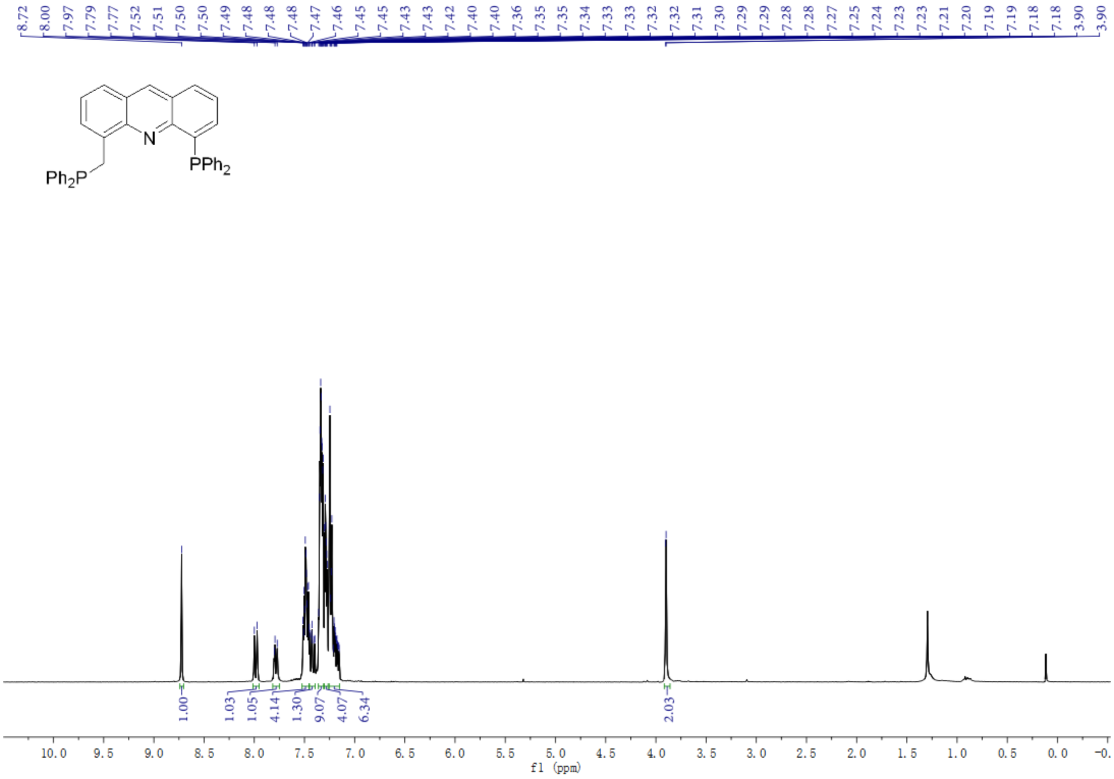

Figure S20. ^{1}H NMR (300 MHz, $CDCl_3$) spectrum of **L2**

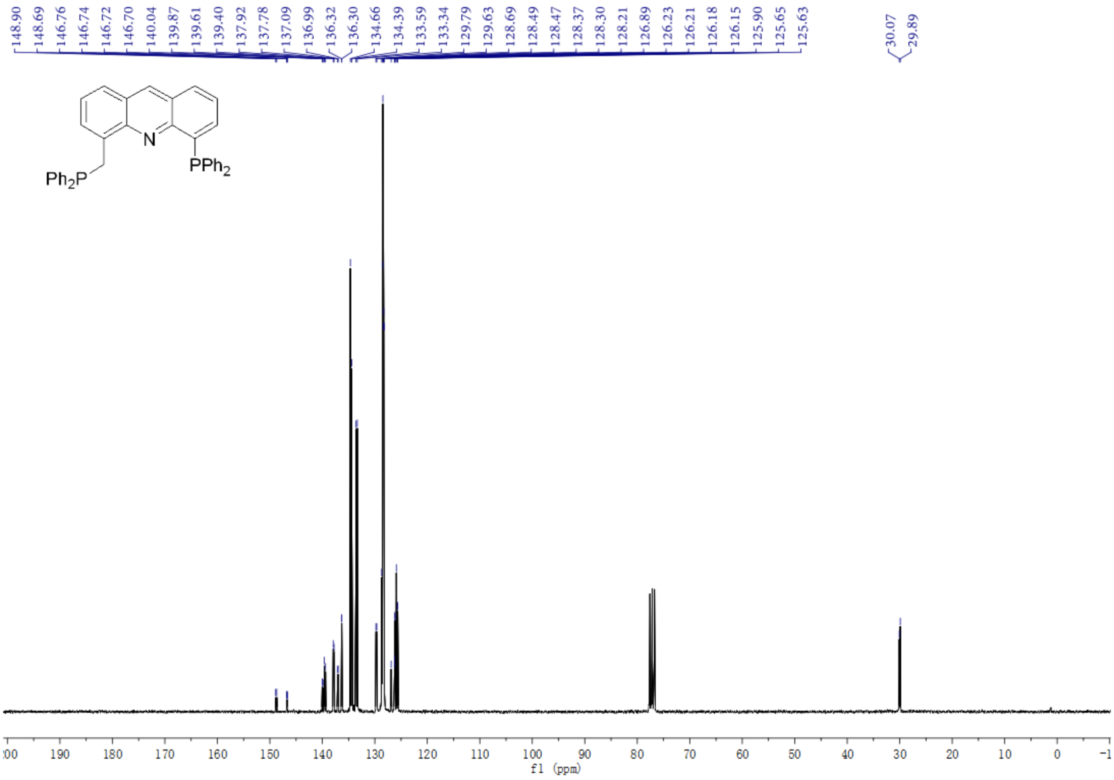

Figure S21. ^{13}C NMR (75 MHz, $CDCl_3$) spectrum of **L2**

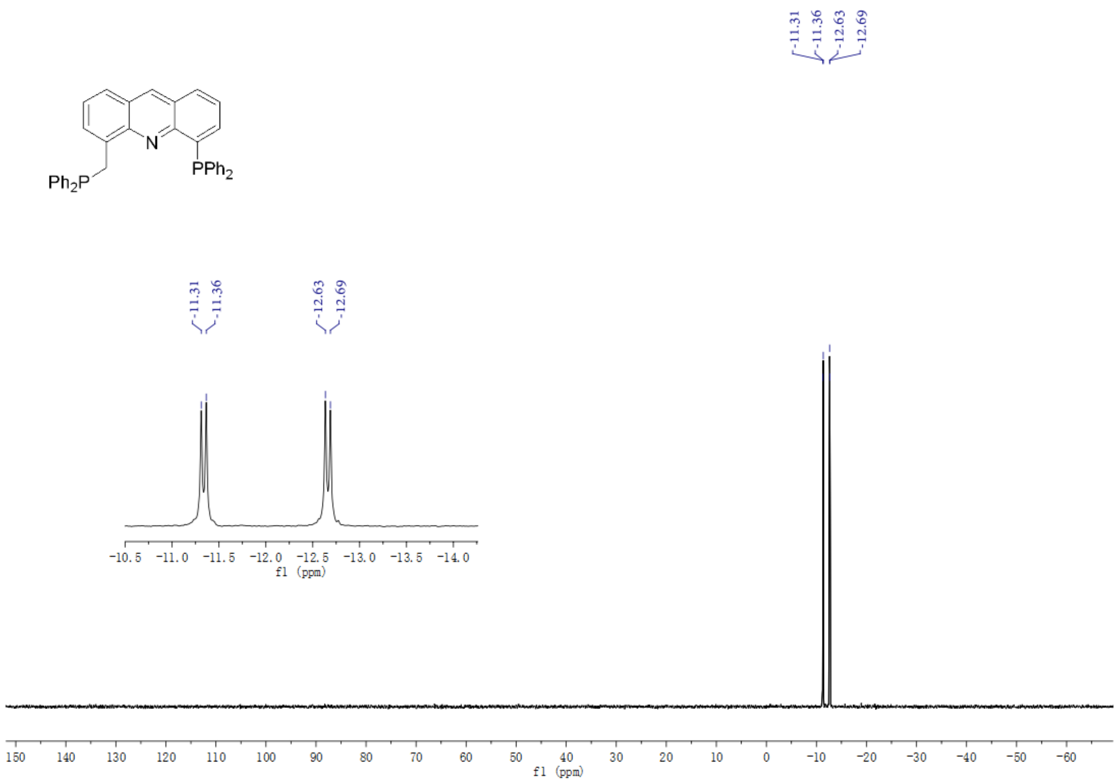

Figure S22. ^{31}P NMR (121 MHz, $CDCl_3$) spectrum of **L2**

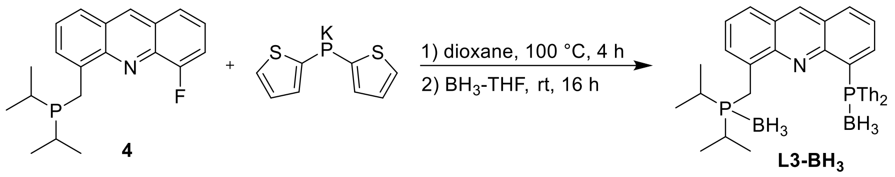

Synthesis of L3-BH_3:

In a glovebox, to a suspension of KH (1.1 equiv.) in anhydrous THF (0.5 M) was added dropwise di(thiophen-2-yl)phosphane[4] (1.0 equiv.) at room temperature. Then the reaction mixture was stirred at room temperature for 12 hours, affording a THF solution of potassium di(thiophen-2-yl)phosphanide (~ 0.5 M), which was used directly in the next step.

In a glovebox, to a solution of 4-((diisopropylphosphanyl)methyl)-5-fluoroacridine **4** (654.8 mg, 2.00 mmol) in dioxane (40 mL) was added dropwise 4.4 mL (~2.20 mmol) of potassium di(thiophen-2-yl)phosphanide in THF. The reaction tube was taken out of the glovebox and heated at 100 °C for 4 h, and cooled to room temperature. Then, BH_3-THF complex (6.0 mL, 6.0 mmol, 1M) was added and the mixture was stirred for 16 h at room temperature under nitrogen protected

condition. The resulting solution was concentrated in *vacuo*. The crude product was purified by flash chromatography (hexane/dichloromethane = 4:1 to 2:1) to give **L3-BH$_3$** as a yellow solid (554.6 mg, 52% yield).

^{1}H NMR (500 MHz, CDCl$_3$) δ 8.80 (s, 1H), 8.32 (d, *J* = 6.9 Hz, 1H), 8.18 (d, *J* = 8.2 Hz, 1H), 7.86 (d, *J* = 8.3 Hz, 1H), 7.71 – 7.65 (m, 2H), 7.61 – 7.45 (m, 5H), 7.22 – 7.15 (m, 2H), 3.77 (d, *J* = 12.6 Hz, 2H), 1.98 (qd, *J* = 14.1, 7.1 Hz, 2H), 1.90 – 1.45 (broad, 3H, BH$_3$), 1.05 (dd, *J* = 14.4, 7.1 Hz, 6H), 0.95 (dd, *J* = 13.8, 7.1 Hz, 6H), 0.79 – 0.30 (broad, 3H, BH$_3$).

^{13}C NMR (126 MHz, CDCl$_3$) δ 147.47 (d, *J* = 7.8 Hz), 147.14 (d, *J* = 5.4 Hz), 138.12 (d, *J* = 6.1 Hz), 137.50, 137.13 (d, *J* = 10.2 Hz), 133.74 (d, *J* = 3.1 Hz), 133.59 (d, *J* = 2.6 Hz), 133.14 (d, *J* = 2.3 Hz), 132.51 (d, *J* = 4.5 Hz), 132.01, 131.50, 130.13, 129.64, 128.55, 128.46, 126.97, 126.78 (d, *J* = 1.6 Hz), 126.45, 126.38 (d, *J* = 1.5 Hz), 125.02, 124.94, 22.94 (d, *J* = 33.3 Hz), 20.20 (d, *J* = 31.2 Hz), 17.50, 17.47.

^{31}P NMR (202 MHz, CDCl$_3$) δ 37.55 – 35.98 (broad), 6.82 – 5.55 (broad).

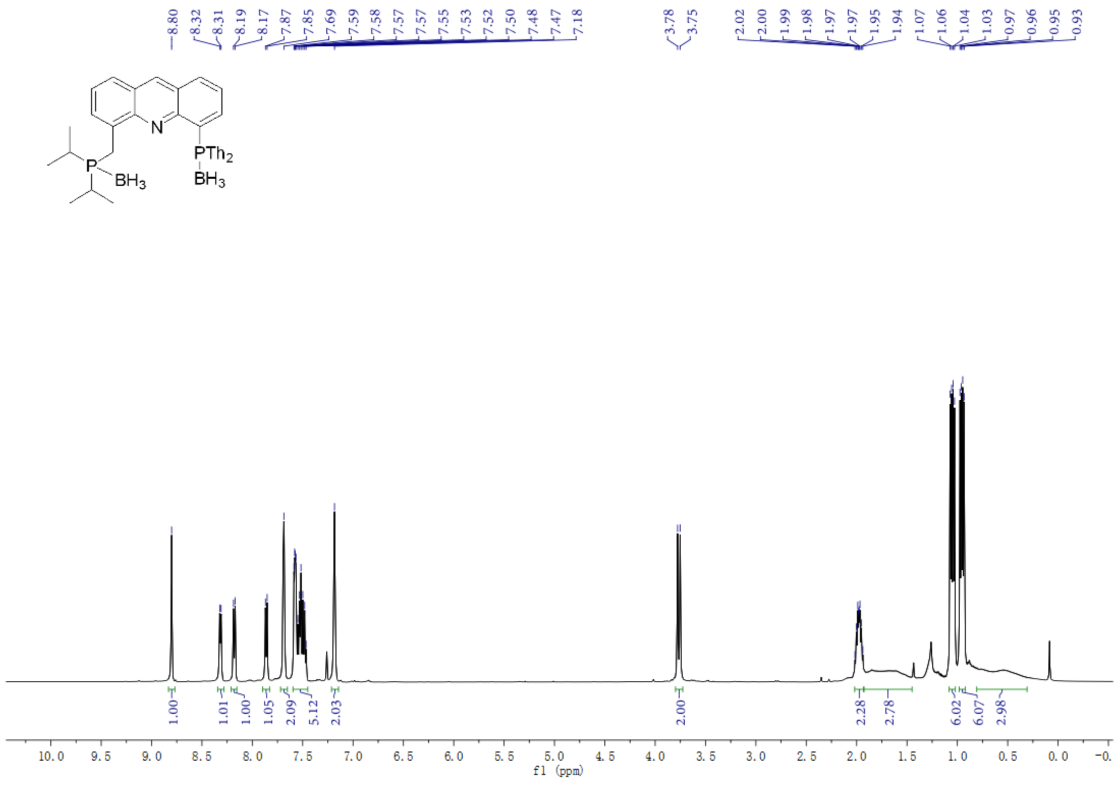

Figure S23. ^{1}H NMR (500 MHz, CDCl$_3$) spectrum of **L3-BH$_3$**

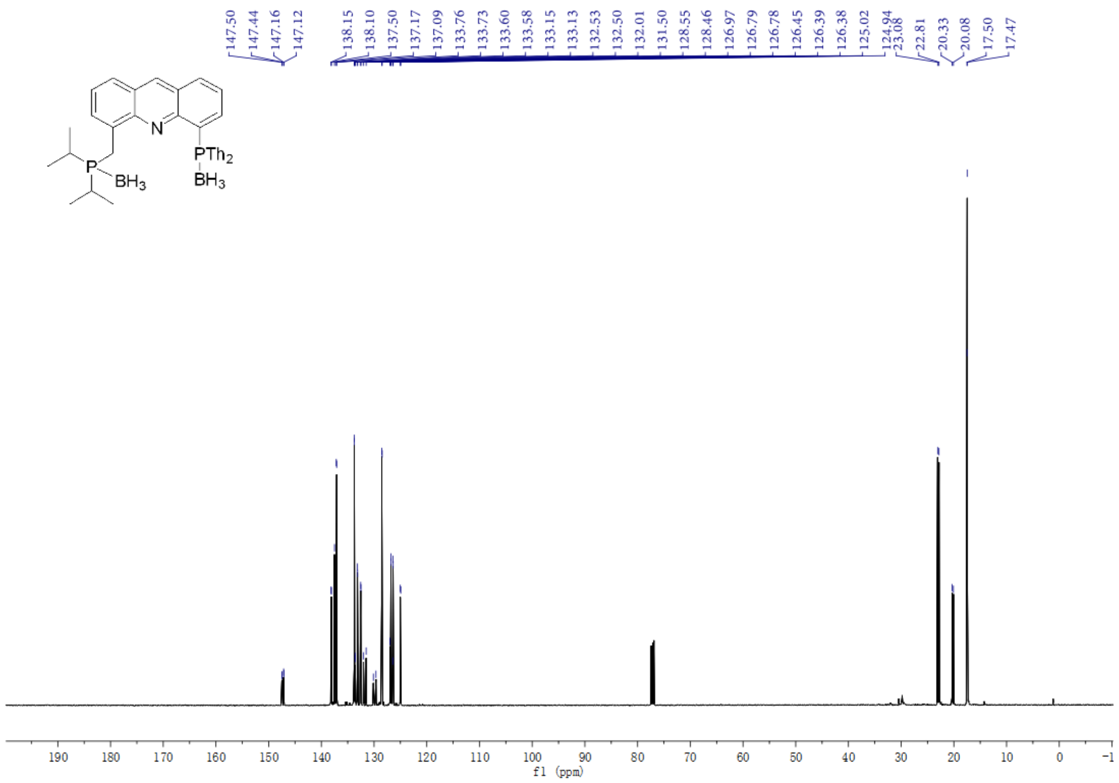

Figure S24. ^{13}C NMR (126 MHz, $CDCl_3$) spectrum of **L3-BH$_3$**

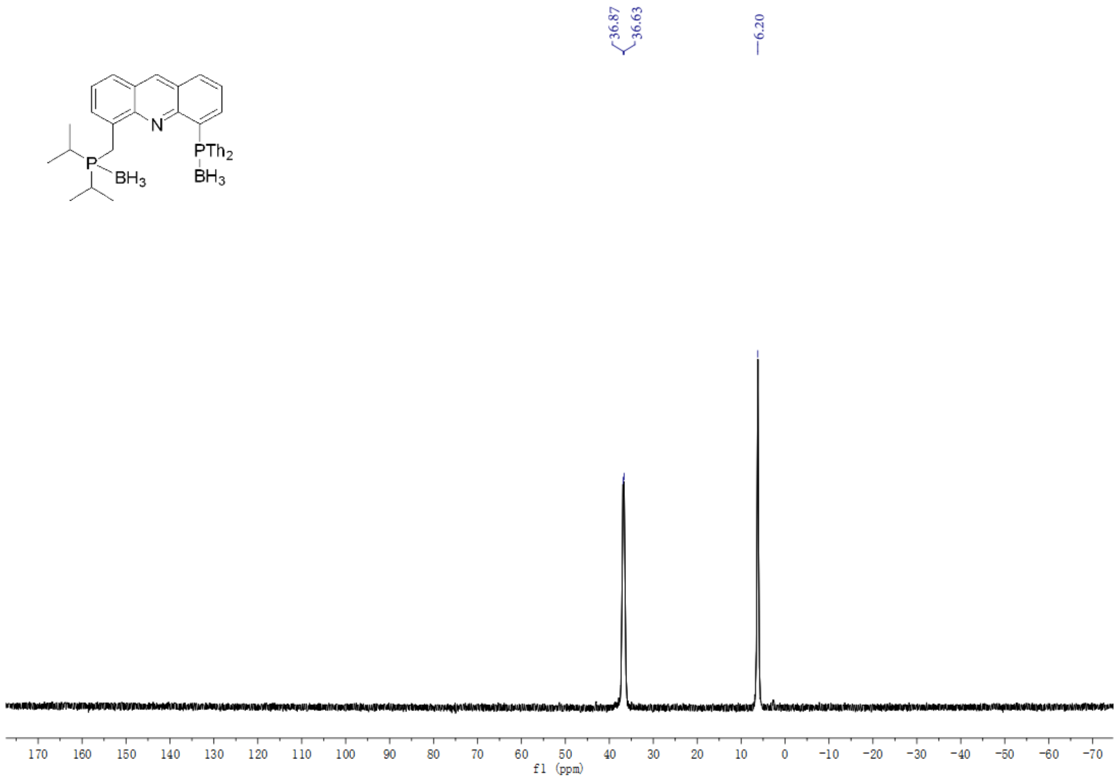

Figure S25. ^{31}P NMR (202 MHz, $CDCl_3$) spectrum of **L3-BH$_3$**

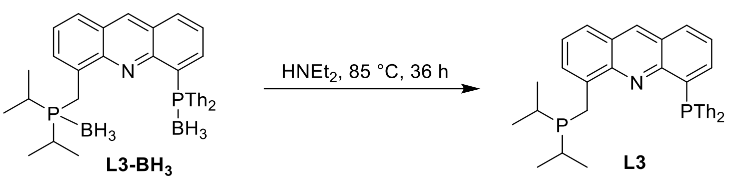

Synthesis of L3:

In a glovebox, **L3-BH₃** (427.0 mg, 0.80 mmol) and $HNEt_2$ (18 mL) were added into a 100 mL Schlenk tube equipped with a magnetic stirring bar. The reaction tube was taken out of the glovebox and heated at 85 °C for 36 h, cooled to room temperature and taken back into the glovebox. The solvent was removed under vacuum and the residue was extracted with diethyl ether. The diethyl ether solution was filtered through Celite and then removed under vacuum. The resulting yellow solid was washed with a small amount of methanol to obtain the pure ligand **L3** (347.8 mg, 86% yield).

^{1}H NMR (500 MHz, $CDCl_3$) δ 8.72 (s, 1H), 7.99 (d, *J* = 8.3 Hz, 1H), 7.94 (d, *J* = 6.4 Hz, 1H), 7.78 (d, *J* = 8.4 Hz, 1H), 7.52 (d, *J* = 4.9 Hz, 2H), 7.48 – 7.42 (m, 2H), 7.41 – 7.35 (m, 3H), 7.11 (t, *J* = 4.0 Hz, 2H), 3.49 (d, *J* = 3.1 Hz, 2H), 1.88 – 1.78 (m, 2H), 1.09 (dd, *J* = 13.4, 7.1 Hz, 6H), 0.97 (dd, *J* = 11.4, 7.0 Hz, 6H).

^{13}C NMR (126 MHz, $CDCl_3$) δ 148.41 (d, *J* = 16.9 Hz), 147.19 (dd, *J* = 2.7, 2.0 Hz), 140.09, 140.02 (d, *J* = 2.5 Hz), 139.97, 139.80, 139.60, 136.49 (d, *J* = 1.8 Hz), 135.43, 135.22, 133.97, 131.40, 130.56, 130.43, 129.30, 128.05 (d, *J* = 7.6 Hz), 126.96, 126.06 (d, *J* = 1.6 Hz), 126.00, 125.70, 125.53 (d, *J* = 2.0 Hz), 24.18 (d, *J* = 13.0 Hz), 22.61 (d, *J* = 16.9 Hz), 20.13 (d, *J* = 14.2 Hz), 19.54 (d, *J* = 10.3 Hz).

^{31}P NMR (202 MHz, $CDCl_3$) δ 14.15, -41.16.

HRMS (ESI): Exact mass calculated for $C_{28}H_{30}NP_2S_2^+$ ($[M+H]^+$): 506.1289, mass found: 506.1295.

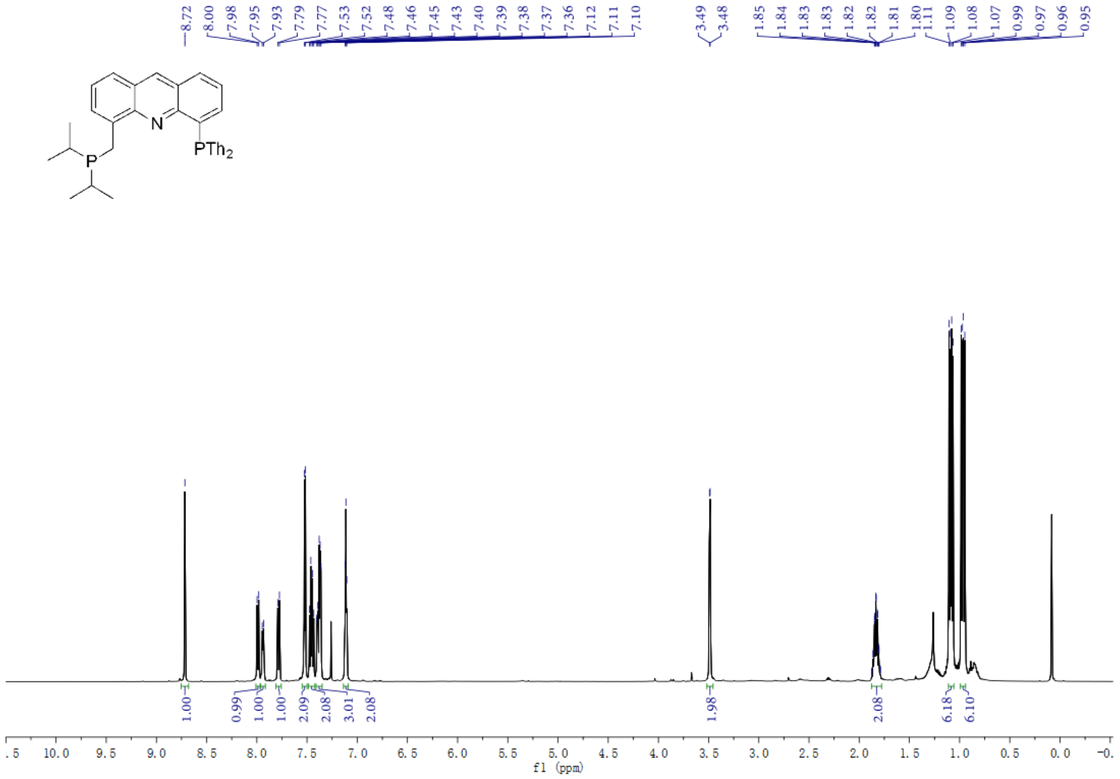

Figure S26. ^{1}H NMR (500 MHz, $CDCl_3$) spectrum of **L3**

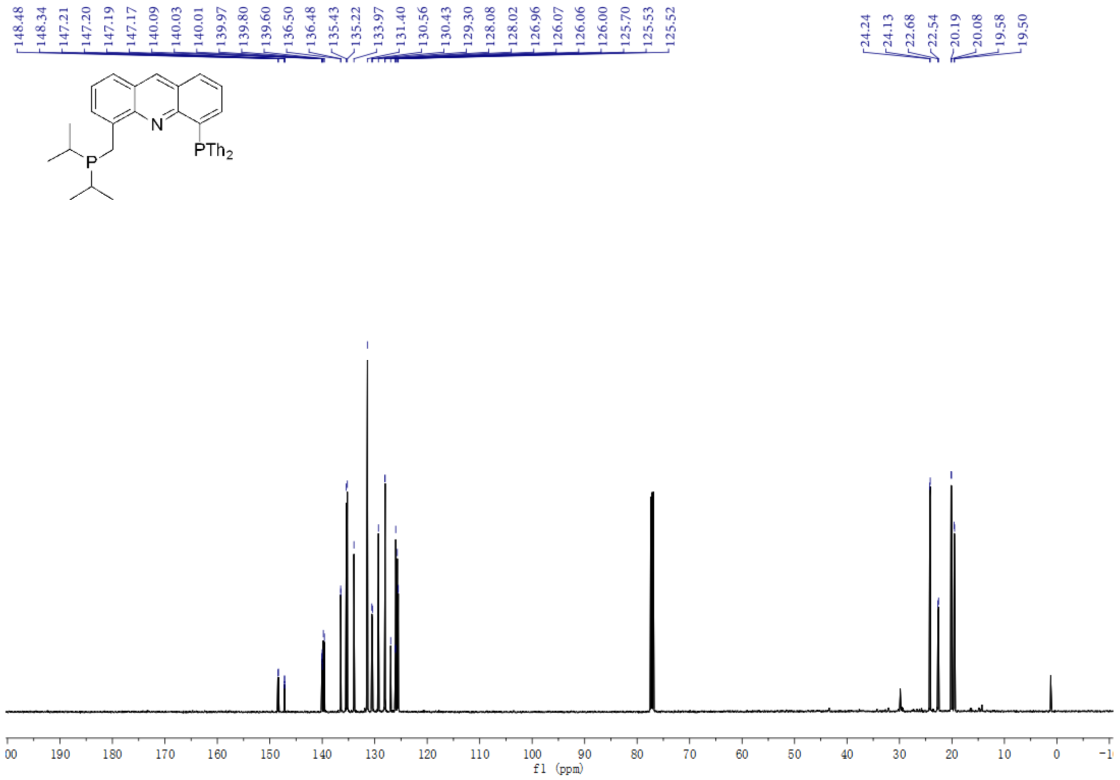

Figure S27. ^{13}C NMR (126 MHz, $CDCl_3$) spectrum of **L3**

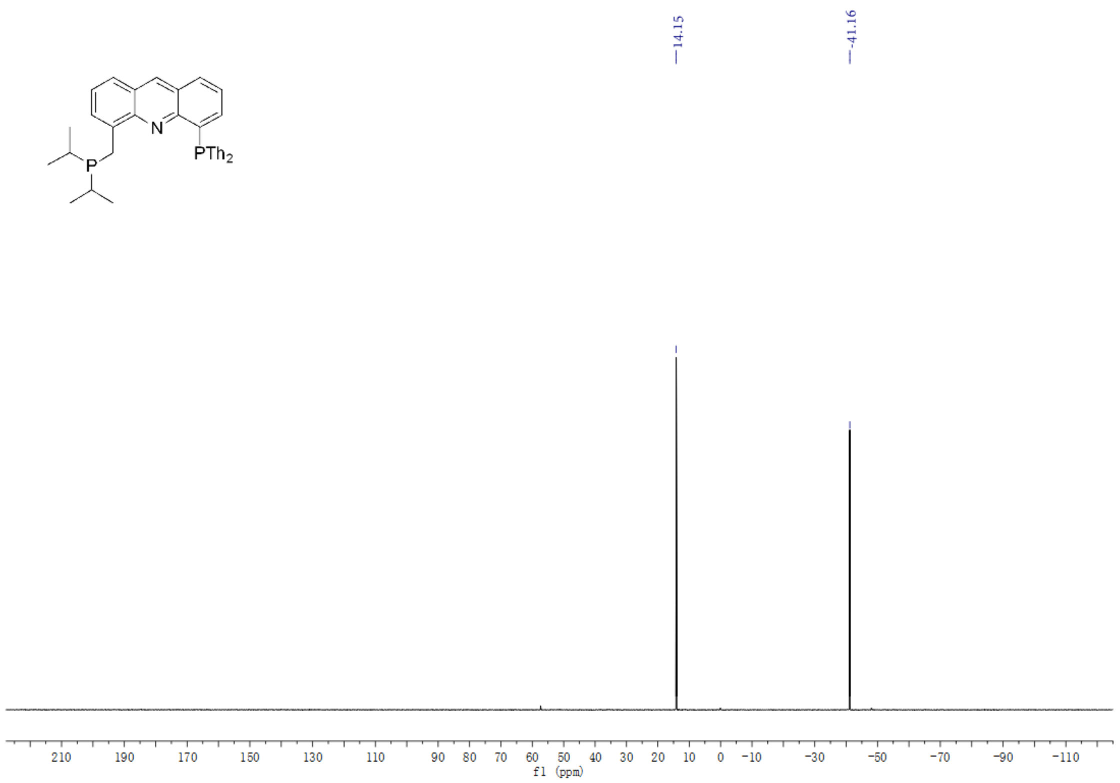

Figure S28. ^{31}P NMR (202 MHz, $CDCl_3$) spectrum of **L3**

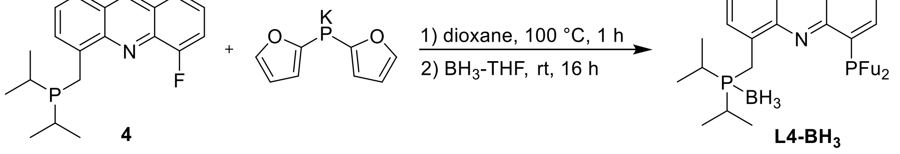

Synthesis of L4-BH_3:

In a glovebox, to a suspension of KH (1.1 equiv.) in anhydrous 1,4-dioxane (0.5 M) was added dropwise di(furan-2-yl)phosphane[4] (1.0 equiv.) at room temperature. Then the reaction mixture was stirred at room temperature for 10 minutes, affording a 1,4-dioxane solution of potassium di(furan-2-yl)phosphanide (~ 0.5 M), which was used directly in the next step.

In a glovebox, to a solution of 4-((diisopropylphosphanyl)methyl)-5-fluoroacridine **4** (114.6 mg, 0.35 mmol) in dioxane (3.5 mL) was added dropwise 3.5 mL (~1.75 mmol) of potassium di(furan-2-yl)phosphanide in 1,4-dioxane. The reaction tube was taken out of the glovebox and heated at 100 °C for 1 h, and cooled to room temperature. Then, BH_3-THF complex (1.1 mL, 1.1 mmol, 1M) was added and the mixture was stirred for 16 h at room temperature under nitrogen protected

condition. The resulting solution was concentrated in *vacuo*. The crude product was purified by flash chromatography (hexane/dichloromethane = 4:1 to 2:1) to give **L4-BH$_3$** as a yellow solid (68.2 mg, 40% yield).

^{1}H NMR (500 MHz, $CDCl_3$) δ 8.74 (s, 1H), 8.04 (d, *J* = 6.6 Hz, 1H), 8.01 (d, *J* = 8.0 Hz, 1H), 7.87 (d, *J* = 8.3 Hz, 1H), 7.66 – 7.62 (m, 2H), 7.54 – 7.43 (m, 3H), 6.68 (s, 2H), 6.43 (s, 2H), 3.89 (d, *J* = 12.6 Hz, 2H), 2.22 – 2.10 (m, 2H), 1.11 (dd, *J* = 14.2, 7.0 Hz, 7H), 1.03 (dd, *J* = 13.7, 6.9 Hz, 7H), 0.73 – 0.15 (broad, 3H, BH_3).

^{13}C NMR (126 MHz, $CDCl_3$) δ 151.64 (d, *J* = 8.4 Hz), 148.65, 148.52, 147.21, 147.19, 146.82 (d, *J* = 2.7 Hz), 136.88, 136.32, 134.66, 133.08 (d, *J* = 4.6 Hz), 132.07 (d, *J* = 4.5 Hz), 129.36, 126.86, 126.84, 126.16 (d, *J* = 1.3 Hz), 126.06, 125.96 (d, *J* = 2.2 Hz), 121.01, 120.85, 111.08, 111.04, 22.49 (d, *J* = 32.3 Hz), 20.54 (d, *J* = 29.8 Hz), 17.48, 17.35.

^{31}P NMR (202 MHz, $CDCl_3$) δ 38.82 – 37.70 (broad), -55.48.

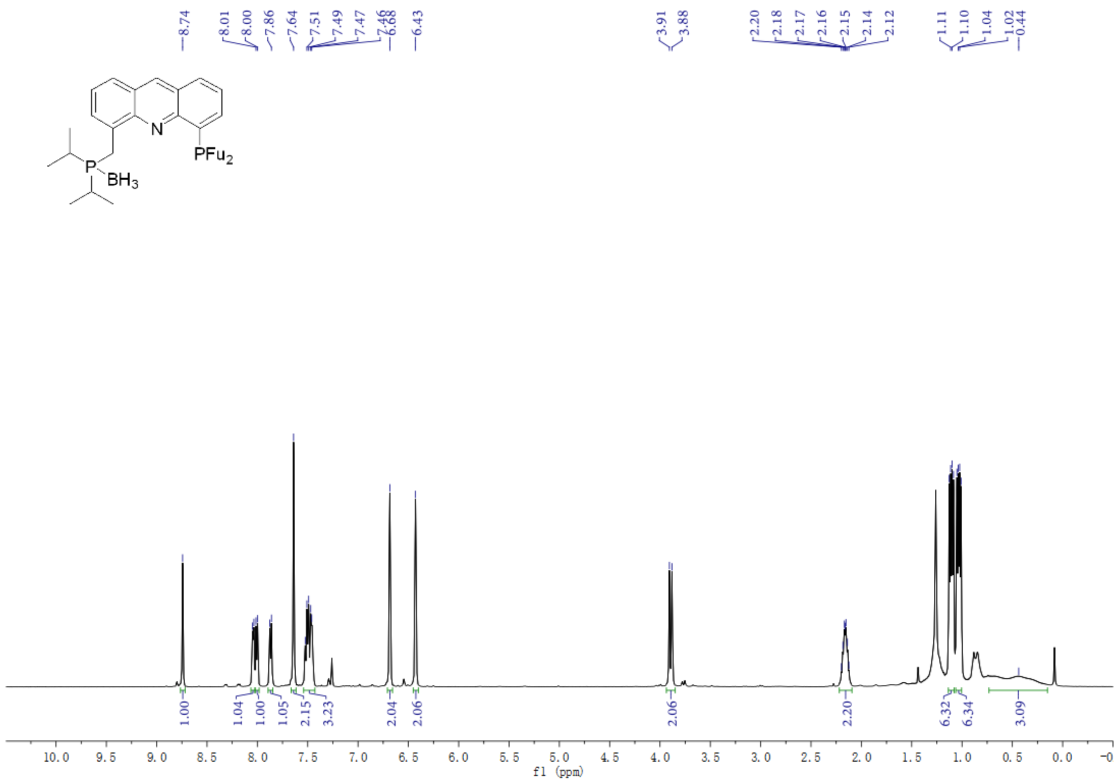

Figure S29. ^{1}H NMR (500 MHz, $CDCl_3$) spectrum of **L4-BH$_3$**

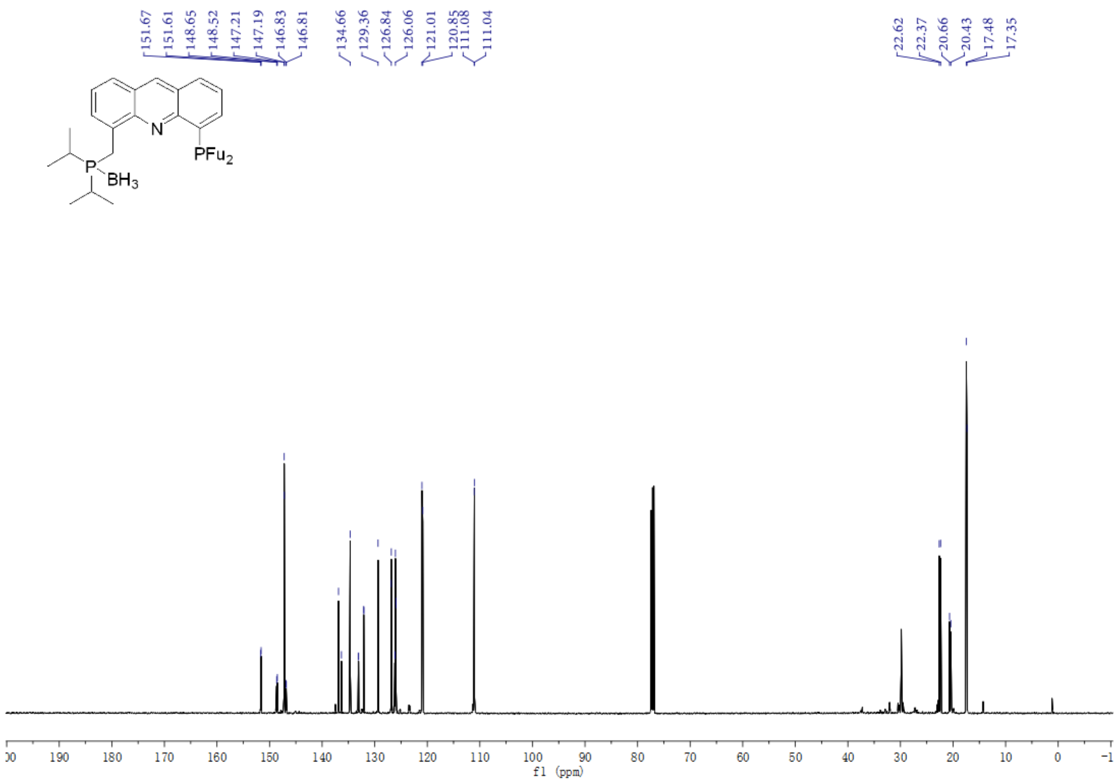

Figure S30. ^{13}C NMR (126 MHz, $CDCl_3$) spectrum of **L4-BH$_3$**

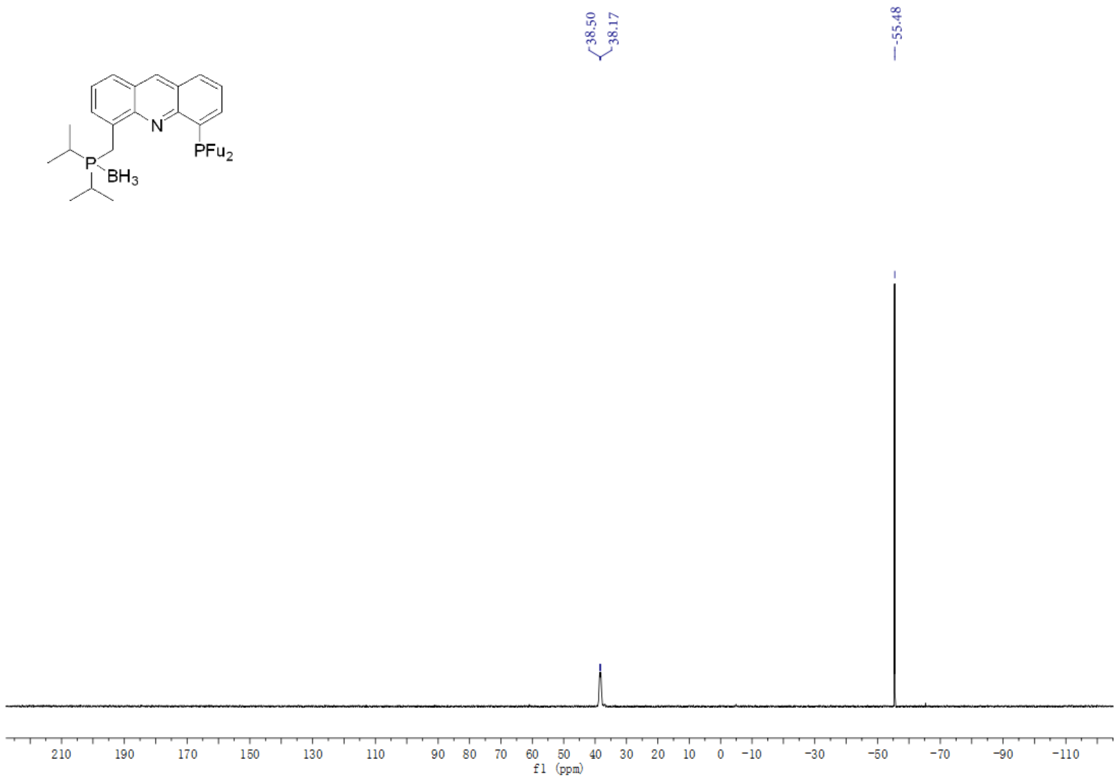

Figure S31. ^{31}P NMR (202 MHz, $CDCl_3$) spectrum of **L4-BH$_3$**

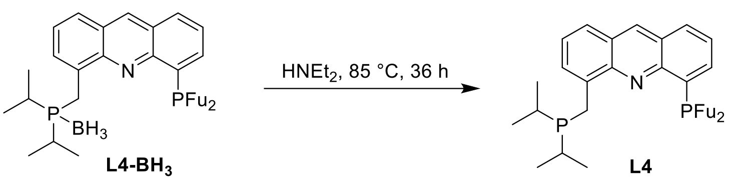

Synthesis of L4:

In a glovebox, **L4-BH$_3$** (58.0 mg, 0.12 mmol) and $HNEt_2$ (4.0 mL) were added into a 50 mL Schlenk tube equipped with a magnetic stirring bar. The reaction tube was taken out of the glovebox and heated at 85 °C for 36 h, cooled to room temperature and taken back into the glovebox. The solvent was removed under vacuum and the residue was extracted with diethyl ether. The diethyl ether solution was filtered through Celite and then removed under vacuum. The resulting yellow solid was washed with a small amount of methanol to obtain the pure ligand **L4** (52.2 mg, 92% yield).

^{1}H NMR (500 MHz, C_6D_6) δ 8.09 (d, *J* = 6.5 Hz, 1H), 8.02 (s, 1H), 7.66 – 7.61 (m, 1H), 7.50 (d, *J* = 8.3 Hz, 1H), 7.40 (d, *J* = 8.3 Hz, 1H), 7.24 (s, 2H), 7.21 (t, *J* = 7.7 Hz, 1H), 7.08 (t, *J* = 7.5 Hz, 1H), 6.76 (s, 2H), 6.07 (s, 2H), 3.78 (s, 2H), 1.97 (dt, *J* = 13.8, 6.9 Hz, 2H), 1.16 (d, *J* = 7.1 Hz, 6H), 1.14 (d, *J* = 7.4 Hz, 6H).

^{13}C NMR (126 MHz, C_6D_6) δ 152.66 (d, *J* = 11.1 Hz), 148.91 (d, *J* = 17.0 Hz), 147.65, 147.09 (d, *J* = 1.8 Hz), 140.72 (d, *J* = 9.4 Hz), 137.56, 136.57 (d, *J* = 1.5 Hz), 134.43, 130.91, 130.78, 129.20, 128.35, 127.22, 126.30, 126.04, 125.76, 125.64, 121.15, 120.98, 111.09, 111.05, 24.54 (d, *J* = 15.1 Hz), 23.07 (d, *J* = 18.8 Hz), 20.32 (d, *J* = 14.9 Hz), 19.67 (d, *J* = 10.9 Hz).

^{31}P NMR (202 MHz, C_6D_6) δ 13.66, -55.32.

HRMS (ESI): Exact mass calculated for $C_{28}H_{30}NO_2P_2^+$ ($[M+H]^+$): 474.1746, mass found: 474.1745.

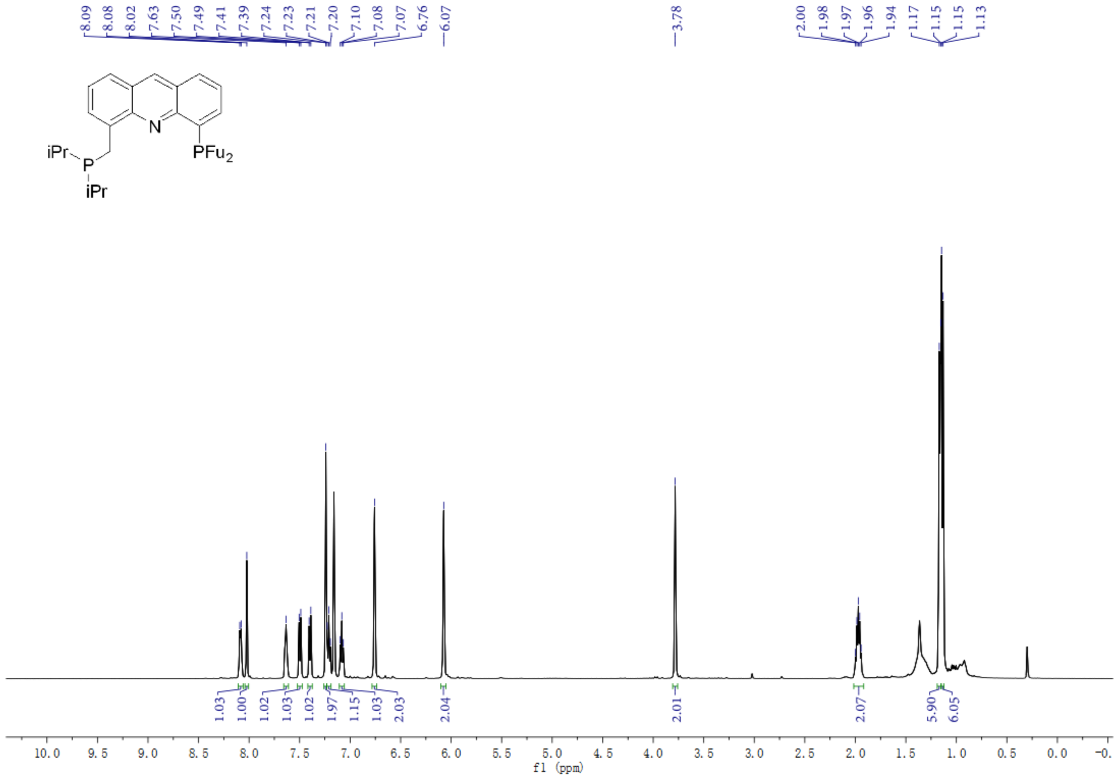

Figure S32. ^{1}H NMR (500 MHz, C_6D_6) spectrum of **L4**

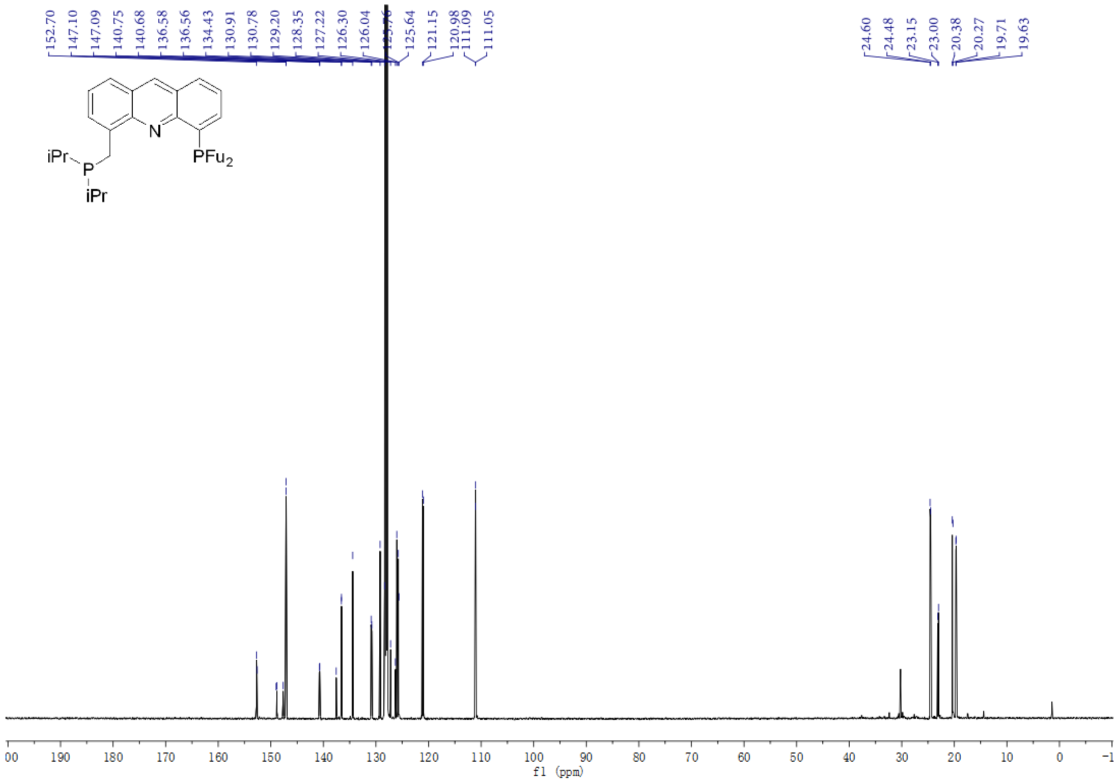

Figure S33. ^{13}C NMR (126 MHz, C_6D_6) spectrum of **L4**

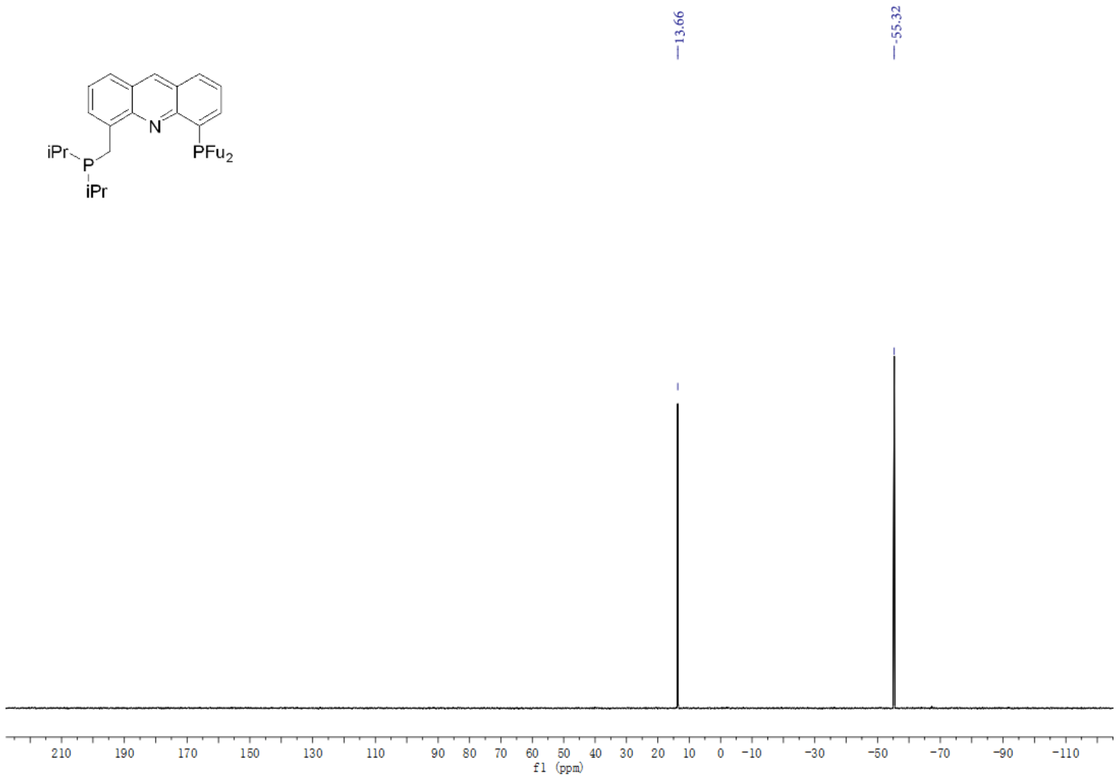

Figure S34. ^{31}P NMR (202 MHz, C_6D_6) spectrum of **L4**

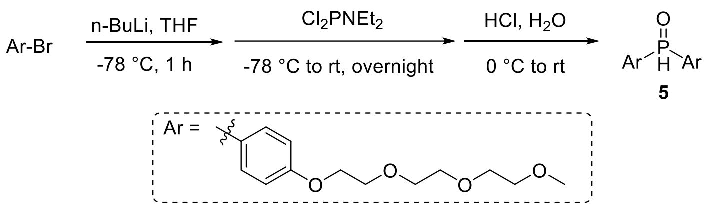

Synthesis of bis(4-(2-(2-(2-methoxyethoxy)ethoxy)ethoxy)phenyl)phosphine oxide (5):

1-Bromo-4-(2-(2-(2-methoxyethoxy)ethoxy)ethoxy)benzene[5] (3.19 g, 10.0 mmol) was added to THF (25 ml) in a 100 ml Schlenk round-bottom flask and cooled to -78 °C. n-BuLi (6.25 mL, 10.0 mmol, 1.6 M in hexanes) was added dropwise to the solution over 10 min and the mixture was allowed to stir for 1 hour, before a solution of diethylphosphoramidous dichloride (870.5 mg, 5.0 mmol) in THF (5.0 mL) was added dropwise. The resulting solution was allowed to warm to room temperature overnight. Hydrochloric acid (10 ml, 40.0 mmol, 4 M) was then added slowly at 0 °C, the resulting solution was warmed to room temperature and stirred for a further 1 hour. The mixture was extracted with dichloromethane and the combined organic phase was dried over Na_2SO_4, filtered and concentrated. The crude product was purified by flash chromatography

(dichloromethane/MeOH = 50:1) to give **5** as a light-yellow oil (1.85 g, 70% yield).

^{1}H NMR (500 MHz, $CDCl_3$) δ 7.96 (d, *J* = 477.4 Hz, 1H). 7.53 (dd, *J* = 12.4, 8.4 Hz, 4H), 6.95 (d, *J* = 8.2 Hz, 4H), 4.16 – 4.08 (m, 4H), 3.85 – 3.77 (m, 4H), 3.69 – 3.65 (m, 4H), 3.60 (m, 8H), 3.50 – 3.46 (m, 4H), 3.31 (s, 6H).

^{13}C NMR (126 MHz, $CDCl_3$) δ 162.13 (d, *J* = 2.6 Hz), 132.59 (d, *J* = 12.9 Hz), 123.12 (d, *J* = 107.8 Hz), 115.01 (d, *J* = 13.9 Hz), 71.89, 70.84, 70.62, 70.54, 69.47, 67.54, 59.00.

^{31}P NMR (202 MHz, $CDCl_3$) δ 21.79.

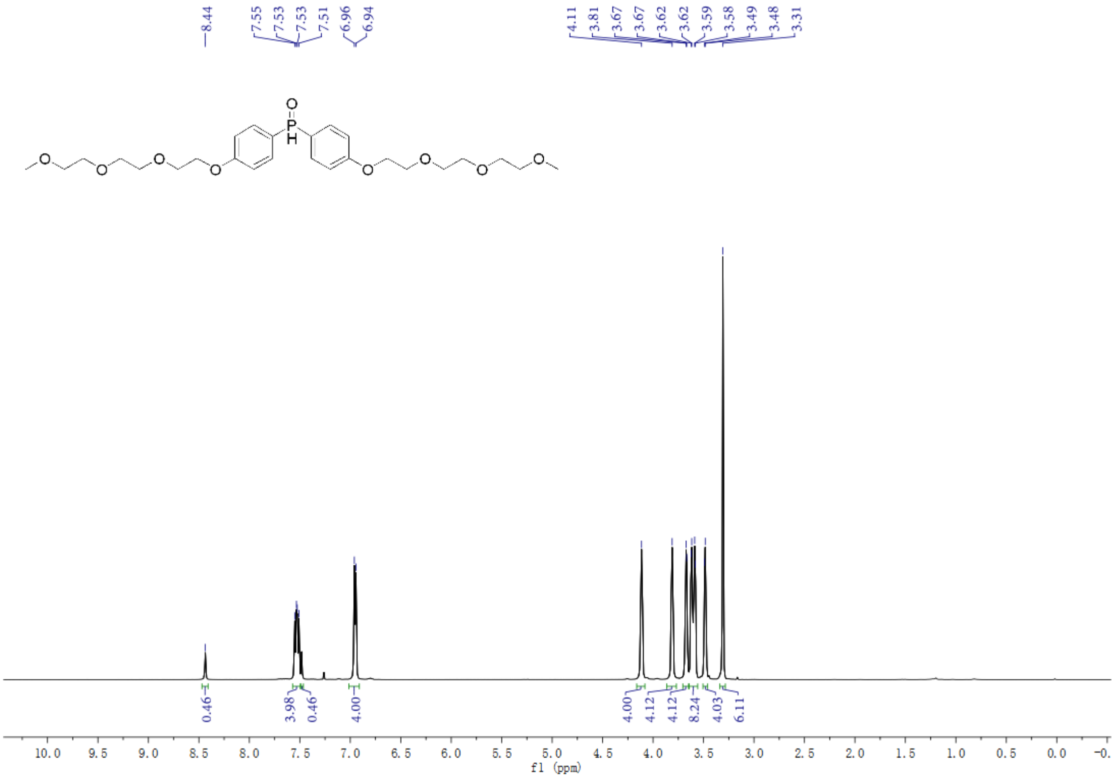

Figure S35. ^{1}H NMR (500 MHz, $CDCl_3$) spectrum of **5**

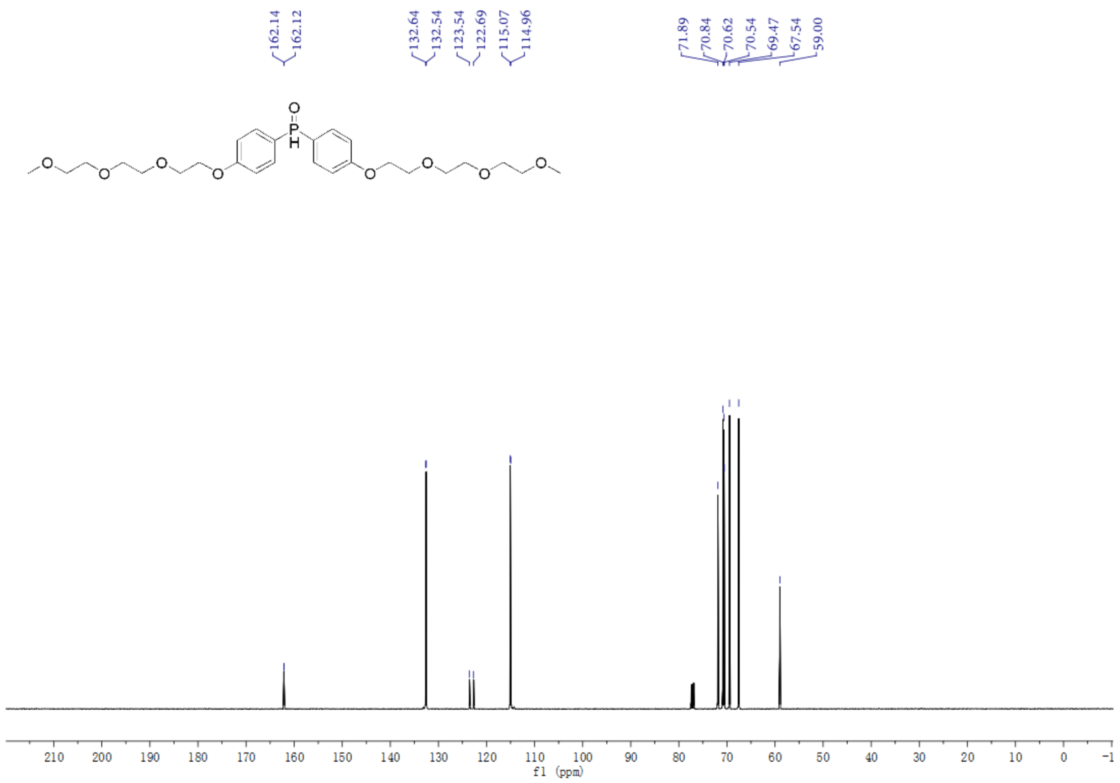

Figure S36. ^{13}C NMR (126 MHz, $CDCl_3$) spectrum of **5**

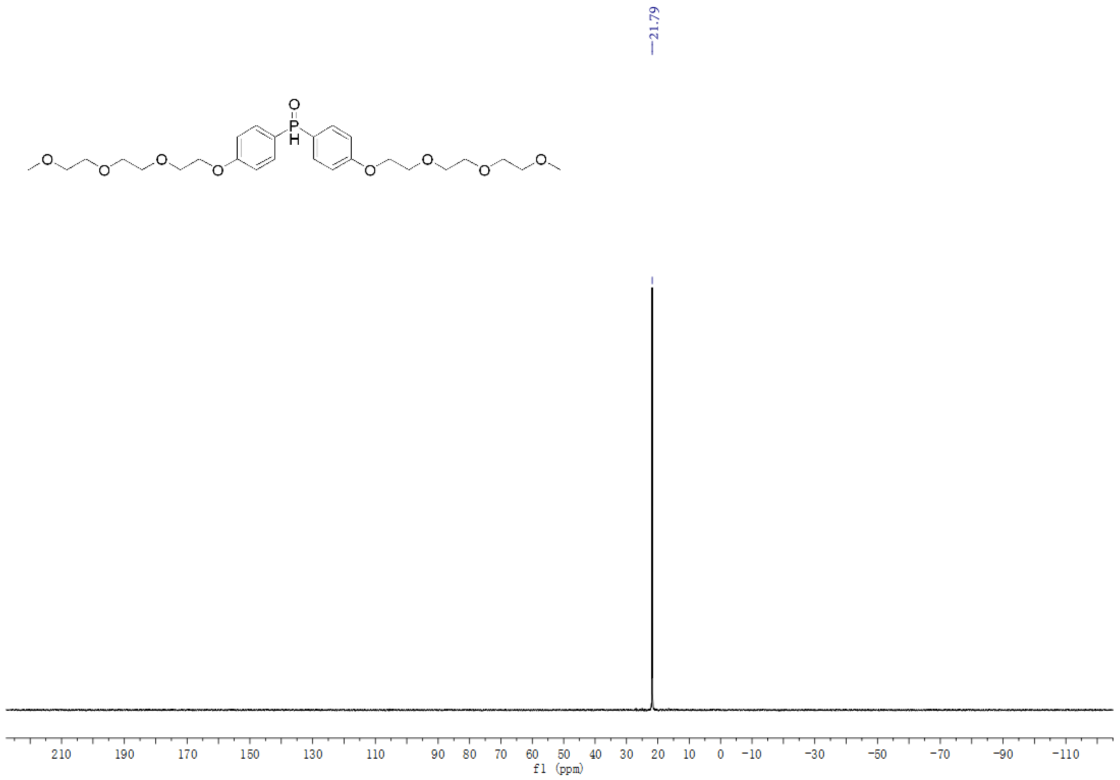

Figure S37. ^{31}P NMR (202 MHz, $CDCl_3$) spectrum of **5**

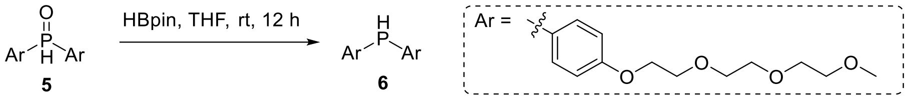

Synthesis of bis(4-(2-(2-(2-methoxyethoxy)ethoxy)ethoxy)phenyl)phosphane (6):

This compound was prepared according to a literature procedure.[6] In a glovebox, **5** (1.85 g, 3.50 mmol) was added to THF (12 ml) a 100 ml round-bottom flask. Pinacolborane (678.3 mg, 5.30 mmol) was then added to the solution and the resulting solution was stirred for 12 hours at room temperature. 4.5 ml of degassed isopropyl alcohol was then added to quench the residual HBPin, after which all the solvent was removed under vacuum. The residue was re-dissolved in MeCN and passed through a plug of alumina. The solvent was then removed under vacuum to afford **6** as a colorless oil (1.63 g, 91%).

^{1}H NMR (500 MHz, $CDCl_3$) δ 7.36 (t, J = 7.7 Hz, 4H), 6.85 (d, J = 8.3 Hz, 4H), 5.16 (d, J = 218.6 Hz, 1H), 4.11 – 4.08 (m, 4H), 3.85 – 3.81 (m, 4H), 3.73 – 3.69 (m, 4H), 3.68 – 3.62 (m, 8H), 3.54 – 3.51 (m, 4H), 3.36 (s, 6H).

^{13}C NMR (126 MHz, $CDCl_3$) δ 159.38, 135.49 (d, J = 18.3 Hz), 126.04 (d, J = 7.4 Hz), 114.99 (d, J = 7.1 Hz), 72.03, 70.93, 70.76, 70.67, 69.75, 67.43, 59.13.

^{31}P NMR (202 MHz, $CDCl_3$) δ -43.48.

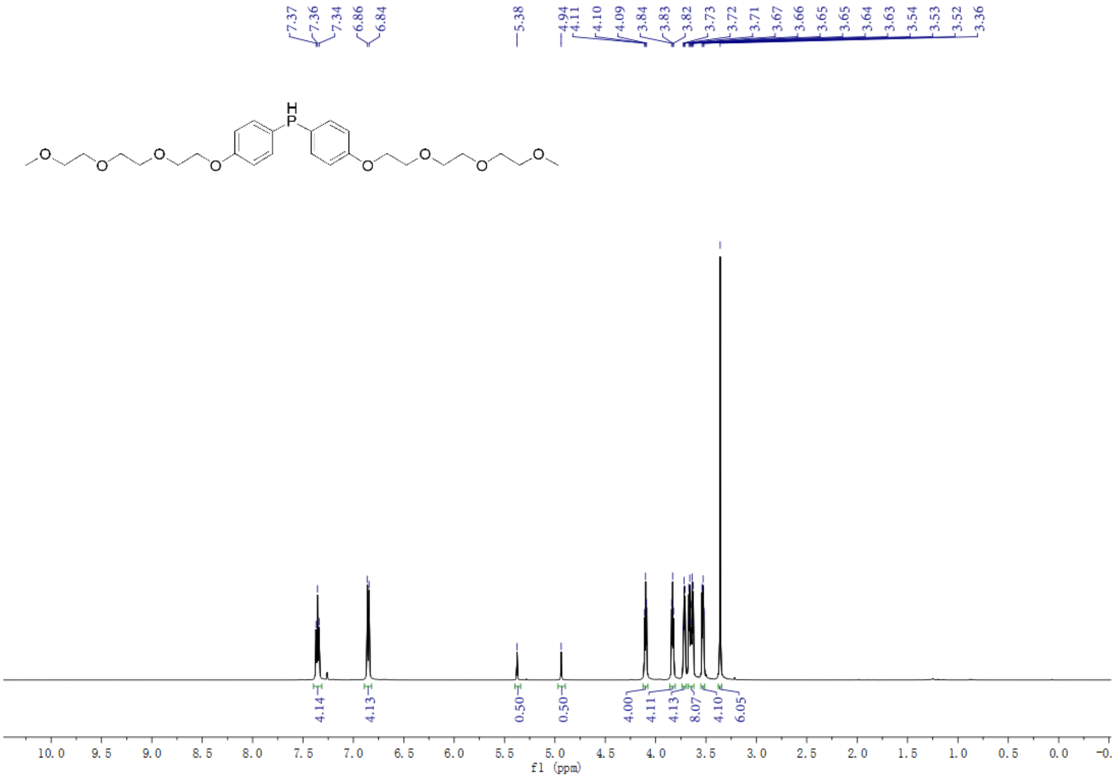

Figure S38. ^{1}H NMR (500 MHz, $CDCl_3$) spectrum of **6**

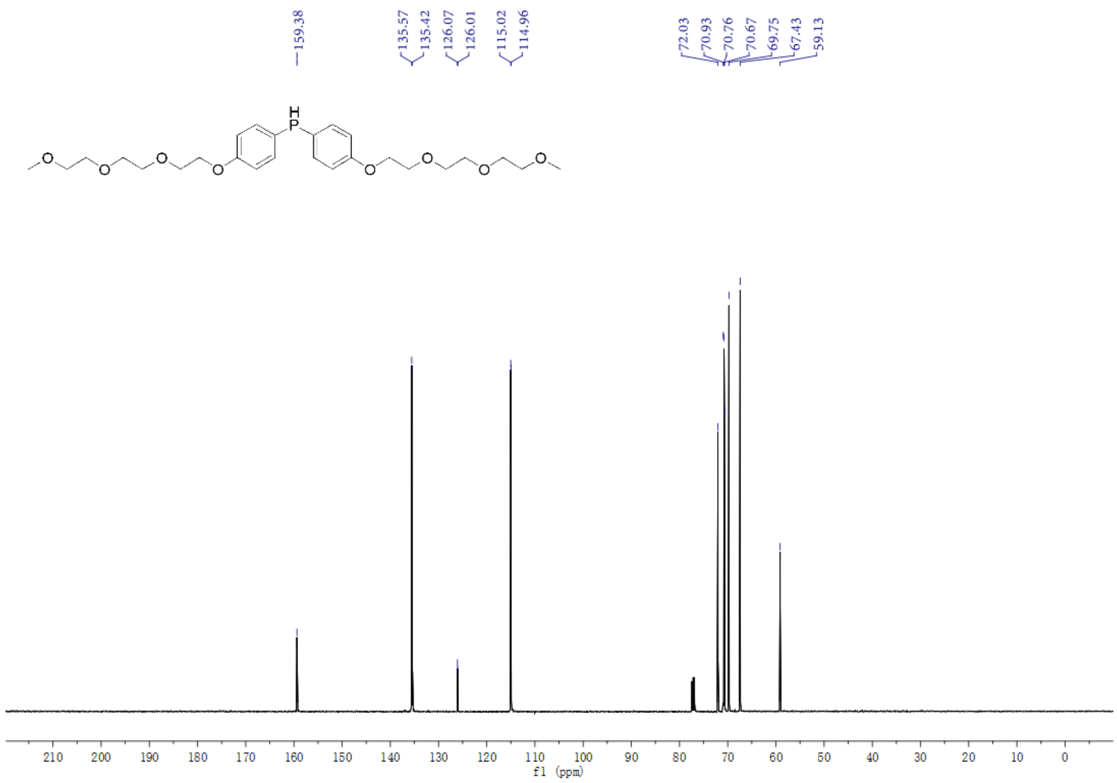

Figure S39. ^{13}C NMR (126 MHz, $CDCl_3$) spectrum of **6**

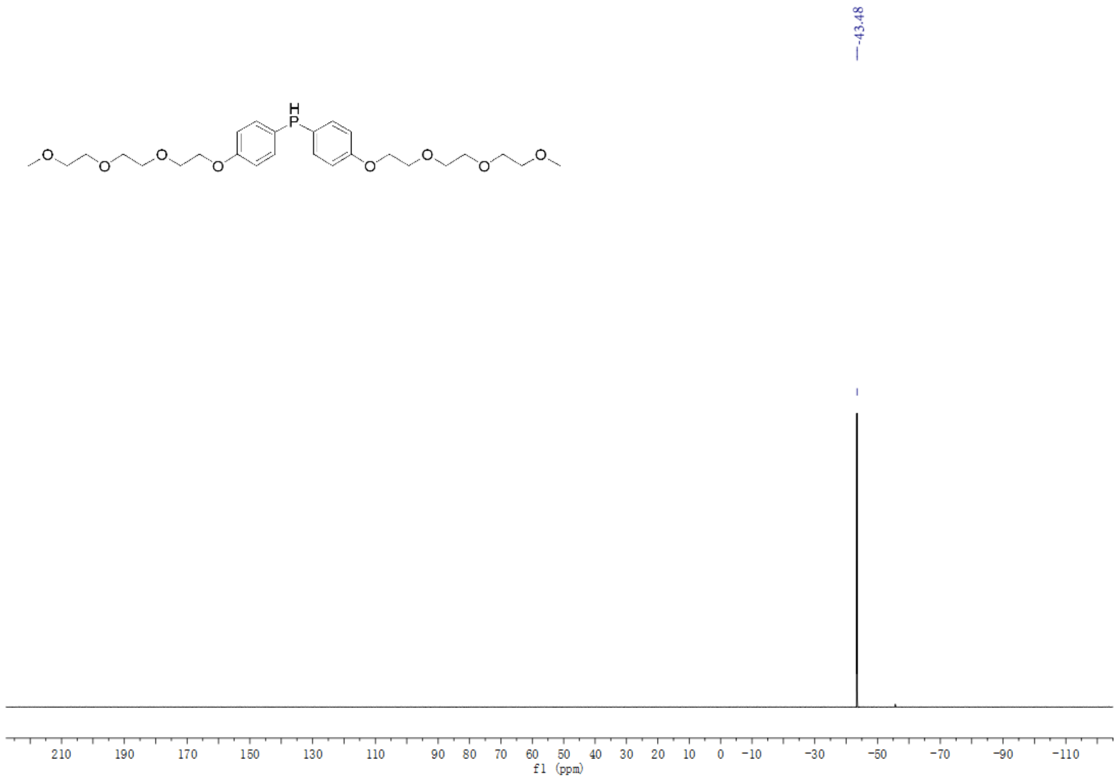

Figure S40. ^{31}P NMR (202 MHz, $CDCl_3$) spectrum of **6**

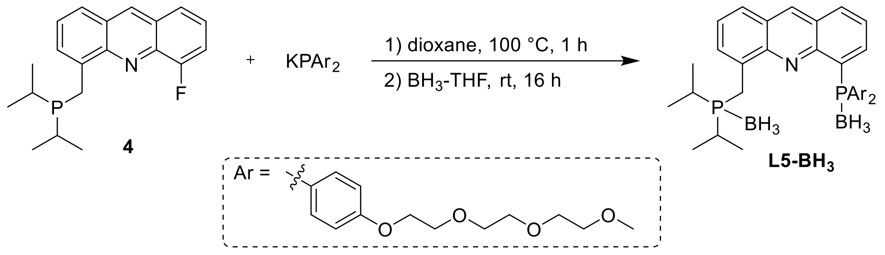

Synthesis of L5-BH$_3$:

In a glovebox, to a suspension of KH (1.1 equiv.) in anhydrous THF (0.2 M) was added dropwise **6** (1.0 equiv.) at room temperature. Then the reaction mixture was stirred at room temperature for 12 hours, affording a THF solution of potassium bis(4-(2-(2-(2-methoxyethoxy)ethoxy)ethoxy)phenyl)phosphanide (~ 0.2 M), which was used directly in the next step.

In a glovebox, to a solution of 4-((diisopropylphosphanyl)methyl)-5-fluoroacridine **4** (163.7 mg, 0.50 mmol) in dioxane (5.0 mL) was added dropwise 3.0 mL (~ 0.60 mmol) of potassium di(furan-2-yl)phosphanide in THF. The reaction tube was taken out of the glovebox and heated at 100 °C for 1 h, and cooled to room temperature. Then, BH_3-THF complex (1.8 mL, 1.8 mmol, 1M) was added and the mixture was stirred for 16 h at room temperature under nitrogen protected condition. The resulting solution was concentrated in *vacuo*. The crude product was purified by flash chromatography (hexane/acetone = 4:1 to 3:1) to give **L5-BH$_3$** as a yellow solid (267.0 mg, 63% yield).

^{1}H NMR (500 MHz, $CDCl_3$) δ 8.79 (s, 1H), 8.27 (d, *J* = 6.9 Hz, 1H), 8.14 (d, *J* = 8.3 Hz, 1H), 7.85 (d, *J* = 8.4 Hz, 1H), 7.53 – 7.46 (m, 5H), 7.43 (t, *J* = 7.6 Hz, 1H), 7.32 (dd, *J* = 12.8, 6.9 Hz, 1H), 6.92 (d, *J* = 8.3 Hz, 4H), 4.12 (t, *J* = 4.5 Hz, 4H), 3.85 (t, *J* = 4.5 Hz, 4H), 3.75 – 3.70 (m, 6H), 3.68 – 3.65 (m, 4H), 3.65 – 3.62 (m, 4H), 3.55 – 3.52 (m, 4H), 3.36 (s, 6H), 1.83 – 1.74 (m, 2H), 1.72 – 1.36 (broad, 3H, BH_3), 1.00 (dd, *J* = 14.4, 7.0 Hz, 6H), 0.84 (dd, *J* = 13.7, 7.0 Hz, 6H), 0.76 – 0.28 (broad, 3H, BH_3).

^{13}C NMR (126 MHz, $CDCl_3$) δ 161.00 (d, *J* = 1.5 Hz), 147.84 (d, *J* = 6.9 Hz), 147.19 (d, *J* = 5.2 Hz), 138.71 (d, *J* = 5.3 Hz), 137.34 (s), 133.77 (d, *J* = 1.7 Hz), 132.47 (s), 130.48 (s), 130.02 (s), 126.96 (s), 126.62 (s), 126.30 (s), 122.07 (s), 121.56 (s), 72.04 (s), 70.98 (s), 70.78 (s), 70.71 (s), 69.64 (s), 67.54 (s), 59.17 (s), 22.89 (d, *J* = 33.4 Hz), 19.85 (d, *J* = 31.1 Hz), 17.46 (s), 17.37 (s).

^{31}P NMR (202 MHz, $CDCl_3$) δ 37.62 – 36.40 (broad), 20.10 – 19.28 (broad).

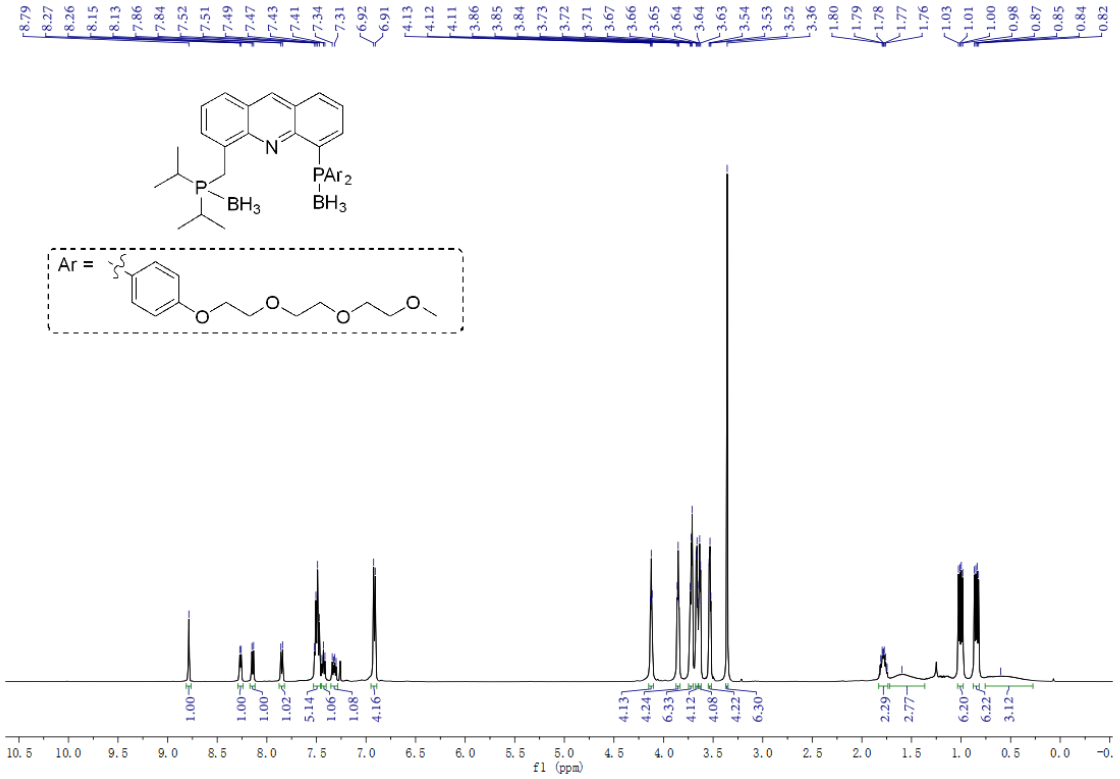

Figure S41. ^{1}H NMR (500 MHz, $CDCl_3$) spectrum of **L5-BH$_3$**

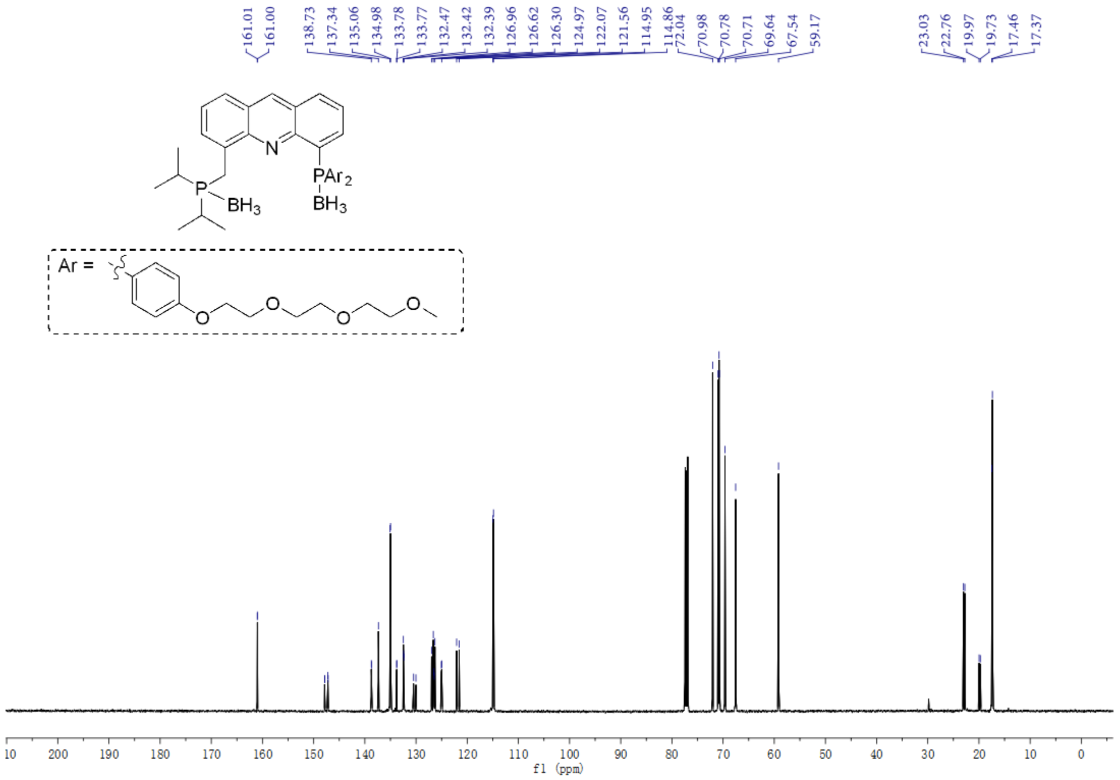

Figure S42. ^{13}C NMR (126 MHz, $CDCl_3$) spectrum of **L5-BH$_3$**

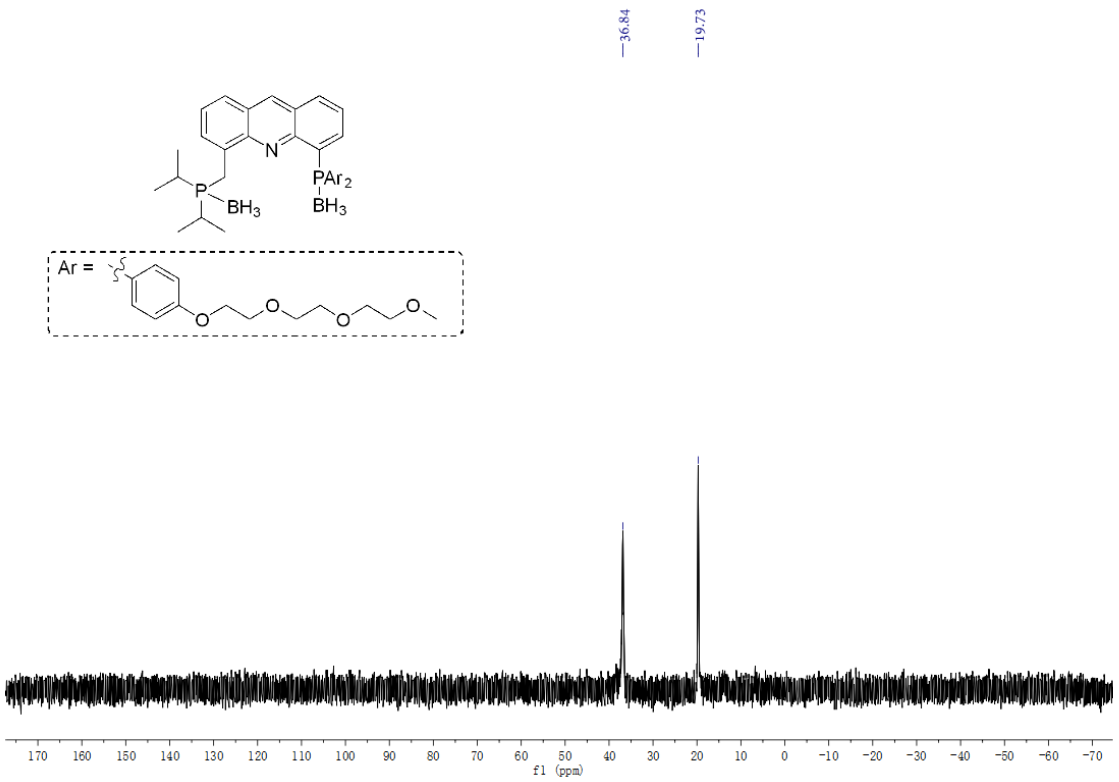

Figure S43. ^{31}P NMR (202 MHz, $CDCl_3$) spectrum of **L5-BH₃**

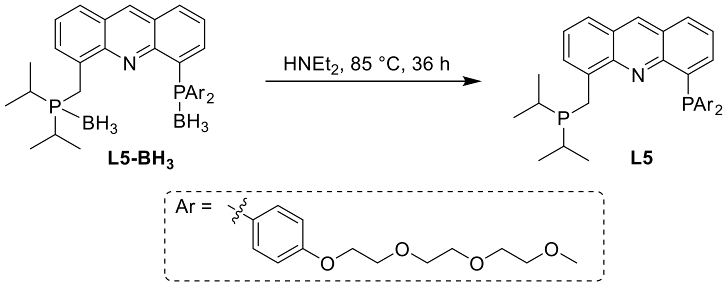

Synthesis of L5

In a glovebox, **L5-BH$_3$** (253.6 mg, 0.30 mmol) and $HNEt_2$ (6.0 mL) were added into a 100 mL Schlenk tube equipped with a magnetic stirring bar. The reaction tube was taken out of the glovebox and heated at 85 °C for 36 h, cooled to room temperature and taken back into the glovebox. The solvent was removed under vacuum and the residue was extracted with toluene. The toluene solution was filtered through Celite and then removed under vacuum. The resulting yellow solid was washed with a small amount of methanol to obtain the pure ligand **L5** (230.8 mg, 94% yield).

^{1}H NMR (300 MHz, $CDCl_3$) δ 8.70 (s, 1H), 7.95 (d, *J* = 8.4 Hz, 2H), 7.77 (d, *J* = 8.2 Hz, 1H), 7.48 – 7.35 (m, 2H), 7.26 (dd, *J* = 8.6, 7.3 Hz, 4H), 7.11 (ddd, *J* = 6.8, 3.8, 1.2 Hz, 1H), 6.86 (d, *J* = 8.1 Hz, 4H), 4.14 – 4.08 (m, 4H), 3.88 – 3.82 (m, 4H), 3.76 – 3.71 (m, 4H), 3.70 – 3.63 (m, 8H), 3.57 – 3.51 (m, 4H), 3.40 (d, *J* = 3.4 Hz, 2H), 3.37 (s, 6H), 1.72 – 1.58 (m, 2H), 1.03 (dd, *J* = 13.6, 7.1 Hz, 6H), 0.84 (dd, *J* = 11.2, 7.0 Hz, 6H).

^{13}C NMR (75 MHz, $CDCl_3$) δ 159.22, 148.73 (d, *J* = 16.0 Hz), 147.07 (dd, *J* = 3.1, 1.8 Hz), 140.31 (d, *J* = 12.3 Hz), 140.04 (d, *J* = 9.5 Hz), 136.27 (d, *J* = 1.3 Hz), 135.74, 135.45, 134.65, 130.32 , 130.10, 129.43, 129.32, 128.52, 126.77, 125.96 (d, *J* = 1.3 Hz), 125.79, 125.70, 125.27 (d, *J* = 1.8 Hz), 114.70, 114.59, 71.96, 70.86, 70.69, 70.61, 69.71, 67.26, 59.06, 24.05 (d, *J* = 12.6 Hz), 22.13 (d, *J* = 16.4 Hz), 20.00 (d, *J* = 14.6 Hz), 19.35 (d, *J* = 9.9 Hz).

^{31}P NMR (121 MHz, $CDCl_3$) δ 13.82, -14.75.

HRMS (ESI): Exact mass calculated for $C_{46}H_{62}NO_8P_2^+$ ($[M+H]^+$): 818.3945, mass found: 818.3976.

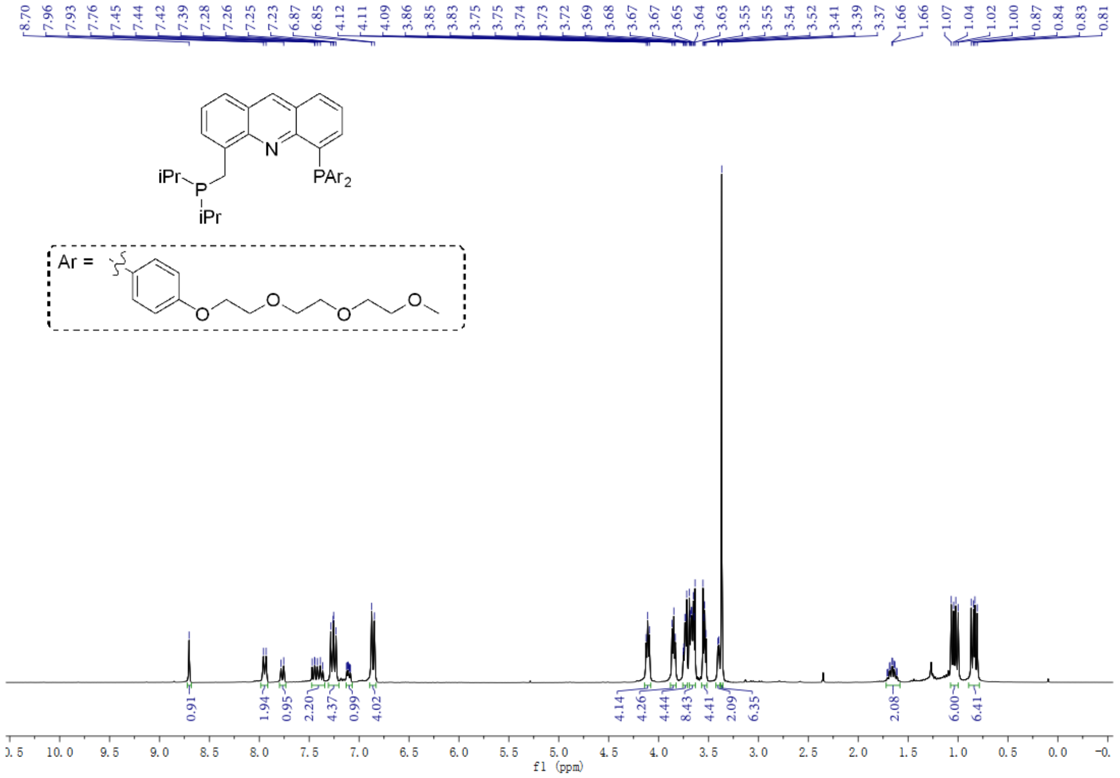

Figure S44. ^{1}H NMR (300 MHz, $CDCl_3$) spectrum of **L5**

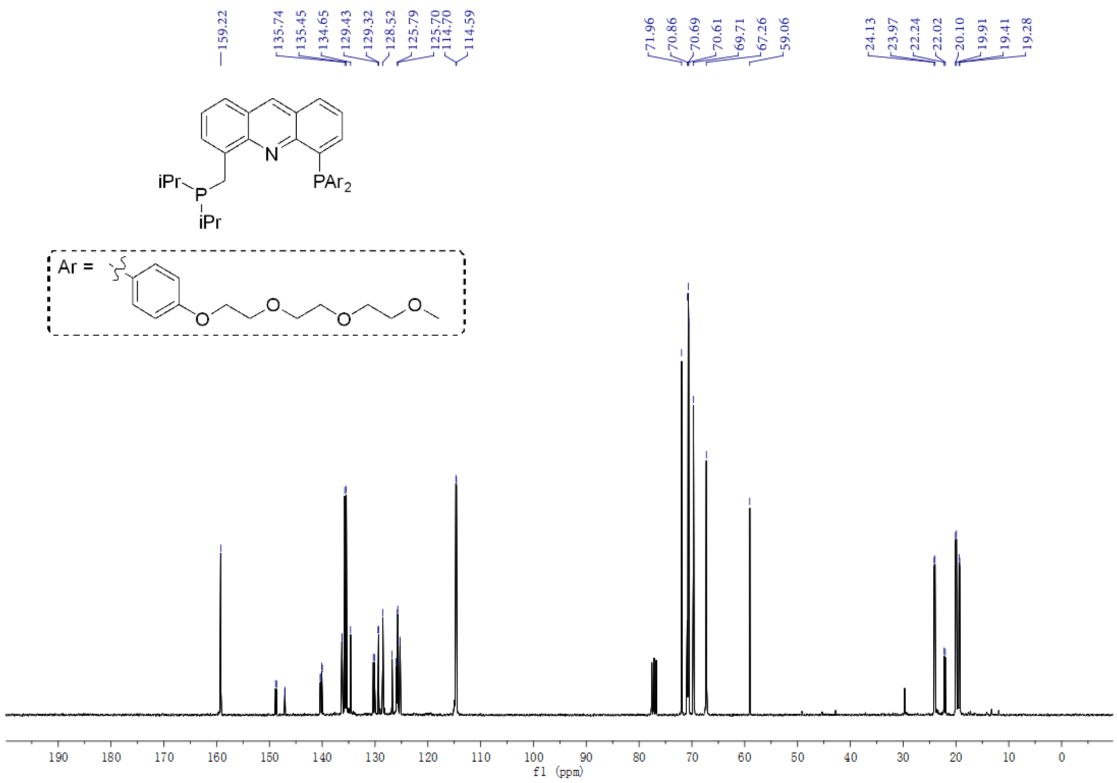

Figure S45. ^{13}C NMR (75 MHz, $CDCl_3$) spectrum of **L5**

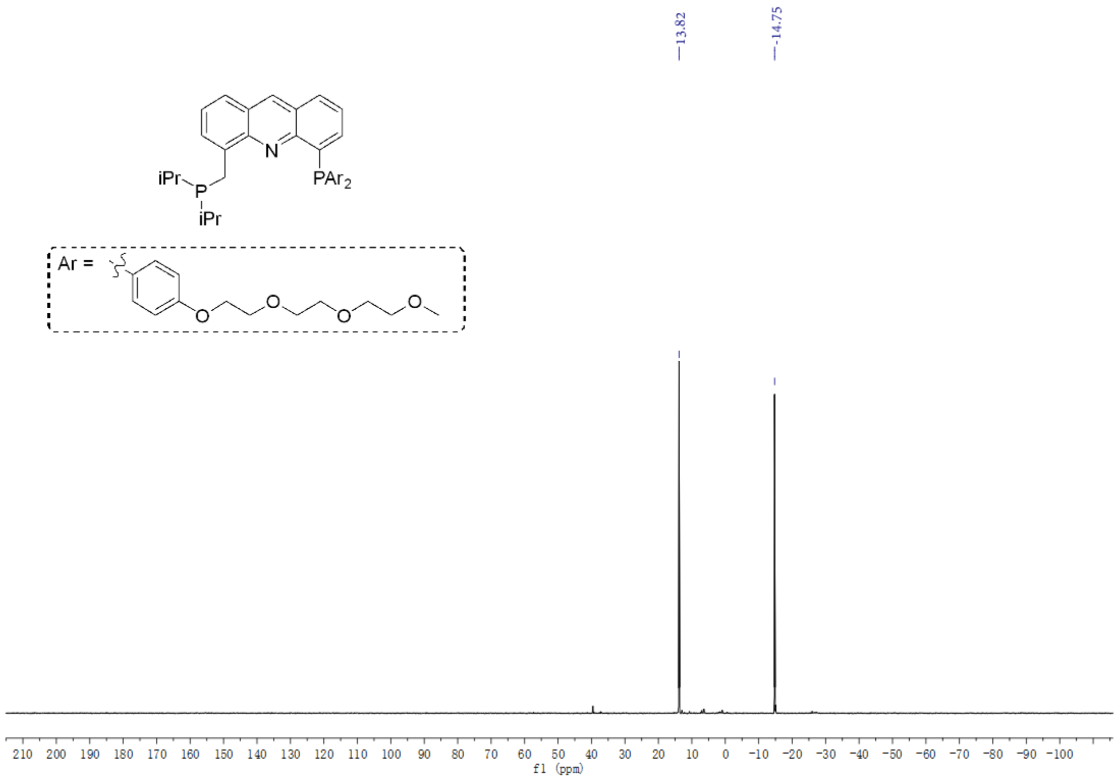

Figure S46. ^{31}P NMR (121 MHz, $CDCl_3$) spectrum of **L5**

3. Preparation of Ru-complexes

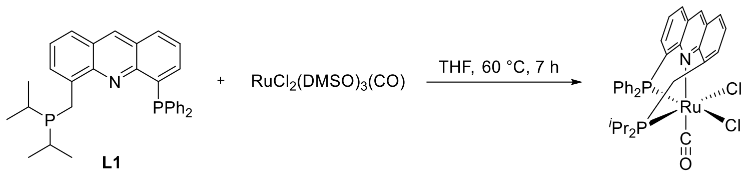

Synthesis of LS-Ru-1-Cl:

In a glovebox, **L1** (118.5 mg, 0.24 mmol) and $RuCl_2(CO)(DMSO)_3$[7,8] (86.9 mg, 0.20 mmol) were suspended in tetrahydrofuran (8.0 mL) in an oven-dried 100 mL Schlenk flask equipped with a magnetic stirring bar. The flask was sealed and taken out of the glovebox, and stirred at 60 ˚C for 7 hours. After cooling the reaction mixture to room temperature, the Schlenk flask was taken into the glovebox again and the solvent was removed under vacuum. The resulting orange solid was washed with ether for several times and dried under vacuum to give the desired product **LS-Ru-1-Cl** as an orange solid (128.2 mg, 92% yield). Crystals suitable for X-ray analysis were obtained by slow evaporation of the dichloromethane solution of **LS-Ru-1-Cl**.

^{31}P NMR (202 MHz, CD_2Cl_2) δ 57.24 (d, *J* = 22.9 Hz), 56.43 (d, *J* = 22.0 Hz).

^{1}H NMR (500 MHz, CD_2Cl_2) δ 9.03 (s, 1H, aryl), 8.27 – 8.20 (m, 2H, aryl), 8.18 (d, *J* = 8.3 Hz, 1H, aryl), 8.08 (dd, *J* = 10.1, 6.7 Hz, 1H, aryl), 8.02 (d, *J* = 8.3 Hz, 1H, aryl), 7.84 (d, *J* = 6.9 Hz, 1H, aryl), 7.63 (t, *J* = 7.6 Hz, 1H, aryl), 7.58 (t, *J* = 7.7 Hz, 1H, aryl), 7.55 – 7.51 (m, 1H, aryl), 7.51 – 7.46 (m, 2H, aryl), 7.43 – 7.37 (m, 1H, aryl), 7.36 – 7.25 (m, 4H, aryl), 4.67 (dd, *J* = 16.5, 11.7 Hz, 1H, C***H***$_2$P), 2.85 (dd, *J* = 16.6, 11.8 Hz, 1H, C***H***$_2$P), 2.53 – 2.43 (m, 1H, PC***H***$(CH_3)_2$), 1.64 (dd, *J* = 15.6, 7.1 Hz, 3H, PCH(C***H***$_3)_2$), 1.30 (dd, *J* = 16.3, 7.1 Hz, 3H, PCH(C***H***$_3)_2$), 0.76 (dd, *J* = 12.2, 7.0 Hz, 3H, PCH(C***H***$_3)_2$), 0.53 – 0.44 (m, 1H, PC***H***$(CH_3)_2$), -0.28 (dd, *J* = 14.7, 7.0 Hz, 3H, PCH(C***H***$_3)_2$).

^{13}C NMR (126 MHz, CD_2Cl_2) δ 142.45 (s, Ar), 138.41 (s, Ar), 138.05 (s, Ar), 137.86 (d, *J* = 1.3 Hz, Ar), 137.42 (d, *J* = 10.3 Hz, Ar), 135.77 (d, *J* = 11.3 Hz, Ar), 135.60 (s, Ar), 134.89 (s, Ar), 132.81 – 132.64 (m, Ar), 131.80 (d, *J* = 2.1 Hz, Ar), 131.55 (d, *J* = 2.5 Hz, Ar), 130.81 (d, *J* = 2.3 Hz, Ar), 130.71 (s, Ar), 129.04 (s, Ar), 128.80 (d, *J* = 10.1 Hz, Ar), 128.44 (d, *J* = 7.4 Hz, Ar), 128.33 (s, Ar), 126.77 (d, *J* = 1.0 Hz, Ar), 125.86 (d, *J* = 7.1 Hz, Ar), 28.13 (d, *J* = 29.4 Hz, P***C***H$(CH_3)_2$), 24.81 (d, *J* = 24.7 Hz, P***C***H$(CH_3)_2$), 24.55 (d, *J* = 28.2 Hz, ***C***H$_2$P), 19.09 (s PCH(***C***H$_3)_2$), 18.36 (d, *J* = 1.7 Hz

PCH($\boldsymbol{C}H_3$)$_2$), 17.79 (d, J = 6.1 Hz PCH($\boldsymbol{C}H_3$)$_2$).

IR (KBr) = 1962 cm^{-1} (CO).

HRMS (ESI): Exact mass calculated for $C_{33}H_{32}NOP_2Ru^+$ ([M-2Cl-H]$^+$): 622.0997, mass found: 622.1017.

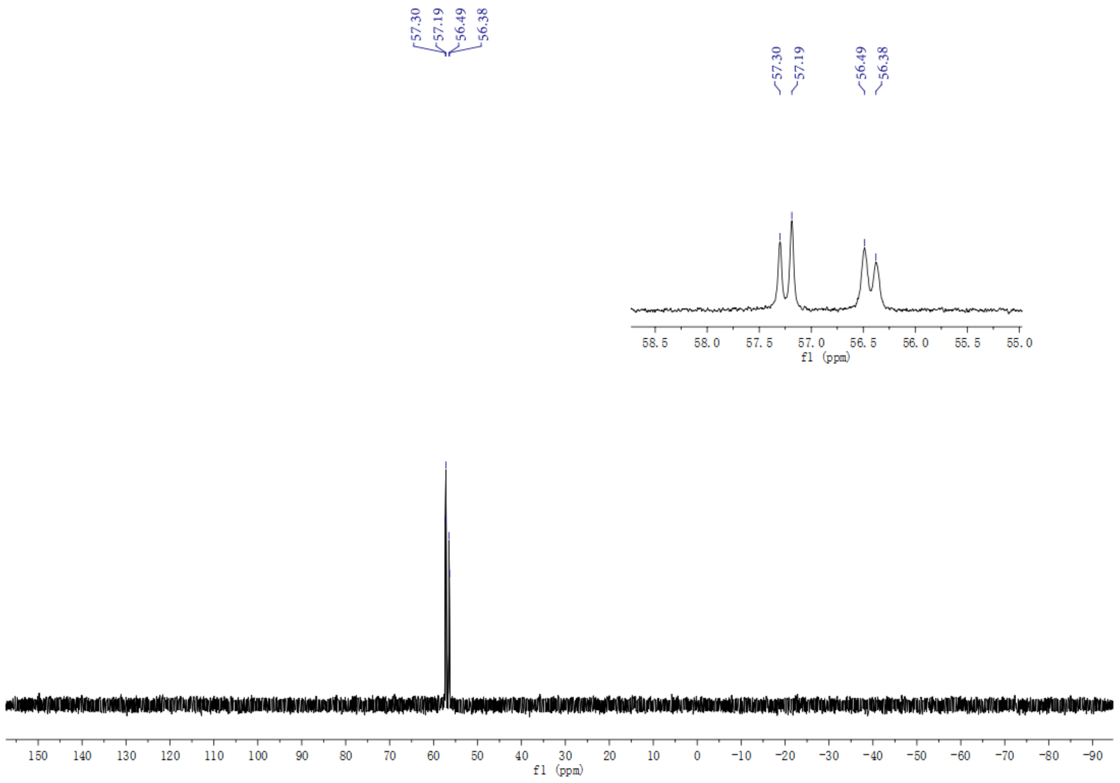

Figure S47. ^{31}P NMR (202 MHz, CD_2Cl_2) spectrum of **LS-Ru-1-Cl**

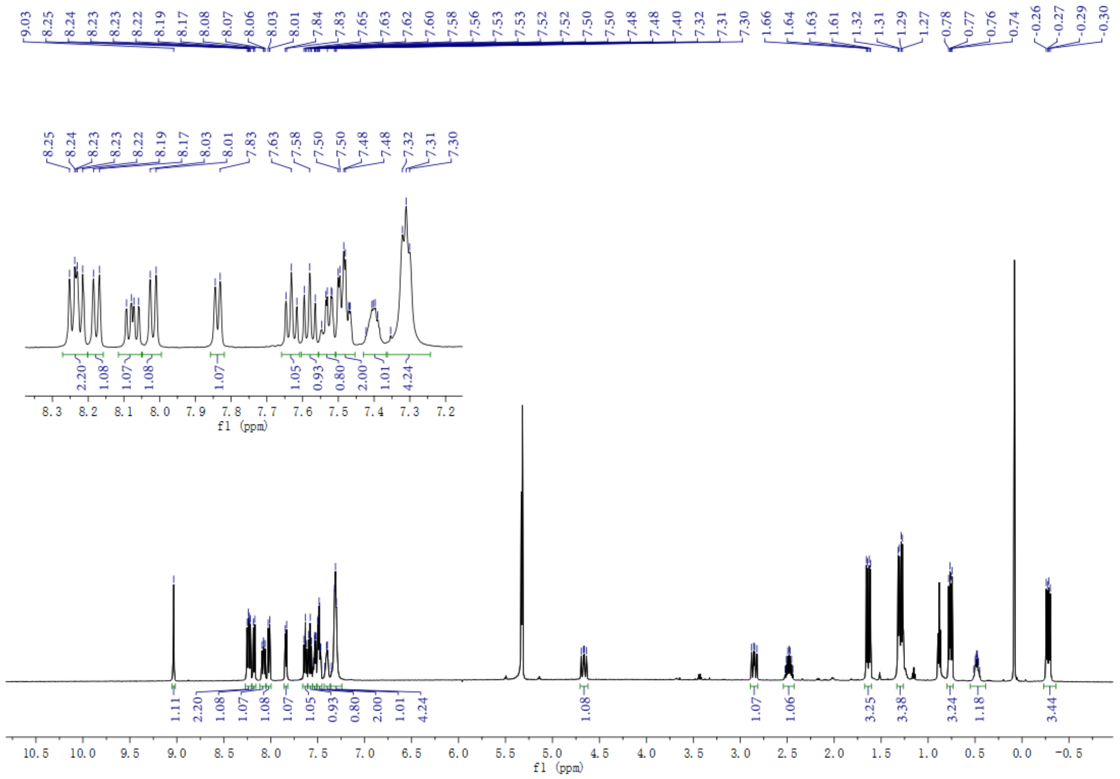

Figure S48. ^{1}H NMR (500 MHz, CD_2Cl_2) spectrum of **LS-Ru-1-Cl**

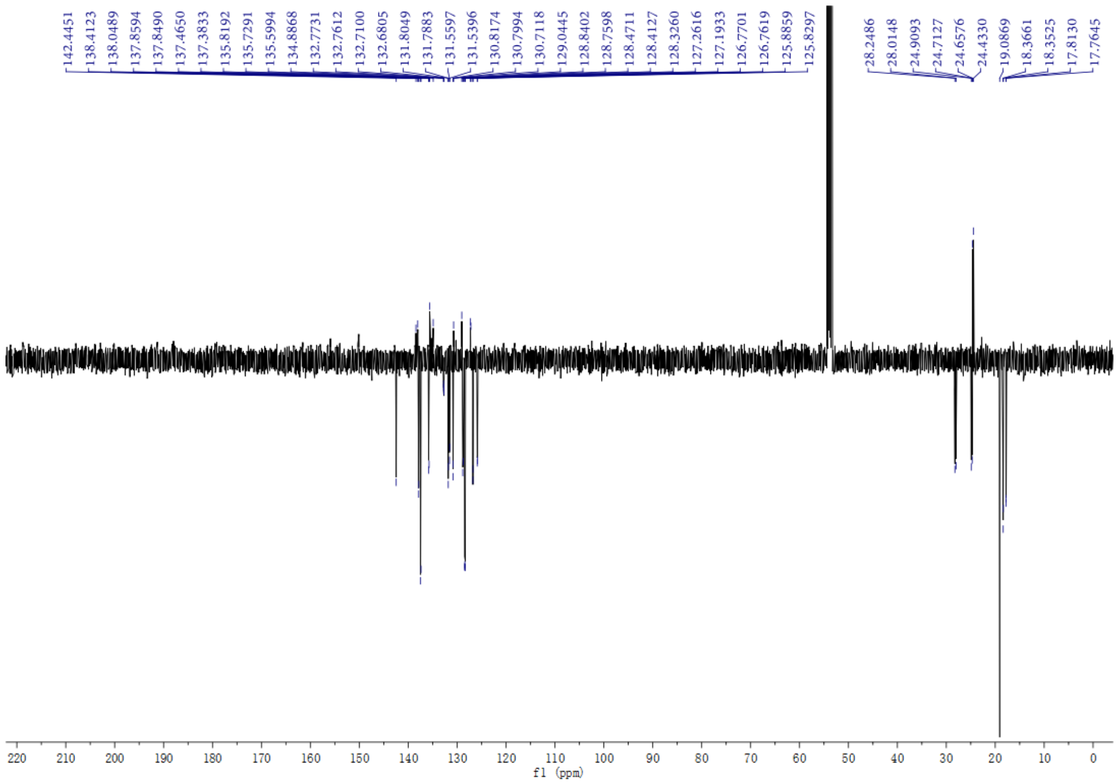

Figure S49. ^{13}C-DEPTQ NMR (126 MHz, CD_2Cl_2) spectrum of **LS-Ru-1-Cl** (CO ligand peaks not observed due to the low solubility of the complex in CD_2Cl_2)

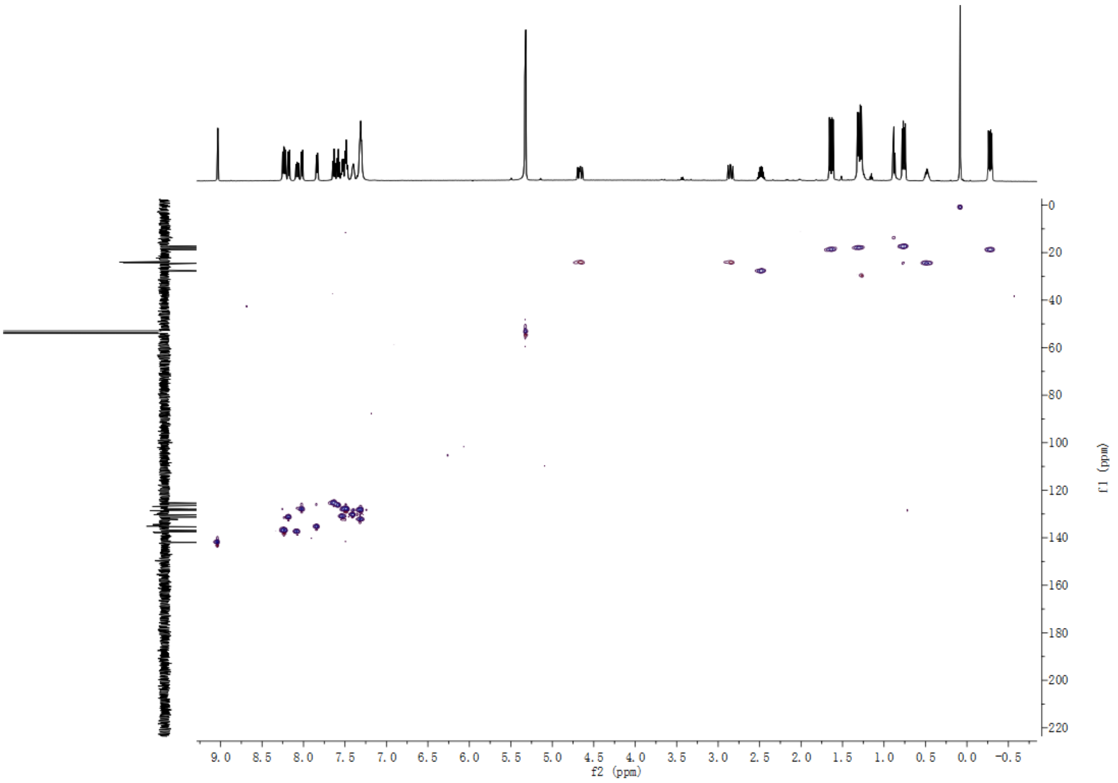

Figure S50. ^{1}H-^{13}C HSQC NMR (500 MHz, CD_2Cl_2) spectrum of **LS-Ru-1-Cl**

The diffraction data from single crystals of **LS-Ru-1-Cl** were at 100 K on a Rigaku Synergy-S diffractometer dual source equipped with Dectris Pilatus3 R CdTe 300K detector, MoKα (λ=0.71073Å). All datasets were processed with CrysAlisPRO and structures were solved with SHELXT[9]. All non-hydrogen atoms were further refined by SHELXL[10] with anisotropic displacement coefficients. Hydrogens were placed in calculated positions and refined in a riding mode. Hydride atoms were located in the electron density map, incorporated and refined. Refinement was carried out with the OLEX-2[11] GUI. Crystallographic data and refinement parameters are summarized in Table S1.

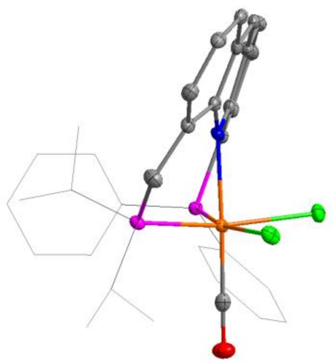

Figure S51. X-ray crystal structure of **LS-Ru-1-Cl**

Table S1. Crystallographic data for complex **LS-Ru-1-Cl**

**CCDC No.**	2382562
**Formula**	$C_{33}H_{33}Cl_2NOP_2Ru + 2CH_2Cl_2$
**Molecular weight**	863.36
**Crystal system**	Triclinic
**Space group**	$P\bar{1}$
**Crystal size (mm)**	0.415×0.268×0.078
**Crystal color and shape**	Yellow Chunk
**Temperature (K)**	100
**Wavelength (Å)**	0.71073
**a (Å)**	12.1470(4)
**b (Å)**	12.1541(4)
**c (Å)**	14.7138(4)
**α (º)**	102.276(3)
**β (º)**	97.576(2)
**γ (º)**	116.988(3)
**Volume (Å^3)**	1825.27(11)
**Z**	2
**r_{calcd} (g · cm^{-1})**	1.571
**μ (mm^{-1})**	0.987
**No. of reflections (unique)**	32320 (7440)
**R_{int}**	0.0398
**Completeness to θ (%)**	99.8
**θ max**	26.372
**Data / restraints / parameters**	7440 / 5 / 432
**Goodness-of-fit on F^2**	1.056
**Final R_1 and wR_2 indices [I>2s(I)]**	0.0390, 0.0971
**R_1 and wR_2 indices (all data)**	0.0460, 0.1005

Highest diff Peak and Deepest hole	1.252, -0.799

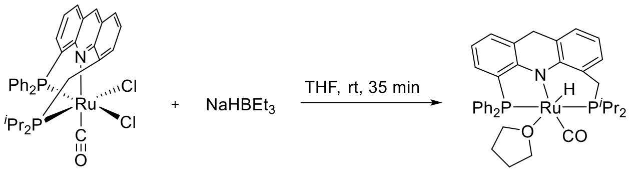

Synthesis of LS-Ru-1:

A THF solution of $NaBEt_3H$ was prepared by adding 200 μl of 1.0 M $NaBEt_3H$ (0.20 mmol) in THF to 1.8 mL of THF. The afforded solution was added to a stirring suspension of 69.3 mg (0.10 mmol) of **LS-Ru-1-Cl** in 8.0 mL of THF. The resulting mixture was stirred at room temperature for 35 minutes, before passed through a 0.2 μm PTFE filter. The solvent was then removed under vacuum. The residue was washed twice with 2 mL of n-pentane to remove a dark impurity, and the remaining solid was dried under vacuum, affording **LS-Ru-1** as a yellow solid (66.7 mg; 96% yield).

^{31}P NMR (202 MHz, THF-d_8) δ 60.38 (d, J = 255.0 Hz), 50.70 (d, J = 257.8 Hz).

^{1}H NMR (500 MHz, THF-d_8) δ 8.01 – 7.94 (m, 2H, aryl), 7.39 – 7.29 (m, 8H, aryl), 7.00 (t, J = 8.5 Hz, 1H, aryl), 6.89 (d, J = 6.7 Hz, 1H, aryl), 6.86 – 6.81 (m, 2H, aryl), 6.57 (t, J = 7.2 Hz, 1H, aryl), 6.51 (t, J = 7.2 Hz, 2H, aryl), 3.80 – 3.70 (m, 2H, ArC***H***$_2$Ar), 3.63 – 3.59 (m, 4H, $CH_2C\boldsymbol{H}_2OC\boldsymbol{H}_2CH_2$), 3.26 (dd, J = 12.9, 5.0 Hz, 1H, C***H***$_2$P), 2.95 (td, J = 12.8, 4.6 Hz, 1H, C***H***$_2$P), 2.46 – 2.37 (m, 1H, PC***H***(CH_3)$_2$), 1.98 – 1.88 (m, 1H, PC***H***(CH_3)$_2$), 1.78 – 1.75 (m, 4H, $C\boldsymbol{H}_2CH_2OCH_2C\boldsymbol{H}_2$), 1.40 (dd, J = 14.9, 7.0 Hz, 3H, PCH(C***H***$_3$)$_2$), 1.32 (dd, J = 13.8, 6.9 Hz, 3H, PCH(C***H***$_3$)$_2$), 1.16 (dd, J = 14.1, 7.7 Hz, 3H, PCH(C***H***$_3$)$_2$), 1.12 (dd, J = 12.0, 7.7 Hz, 3H, PCH(C***H***$_3$)$_2$), -18.80 (t, J = 20.5 Hz, 1H, Ru-***H***).

^{13}C NMR (126 MHz, THF-d_8) δ 207.15 (s, Ru-***C***O), 165.86 (dd, J = 28.4, 2.8 Hz, Ar), 148.79 (d, J = 5.2 Hz, Ar), 138.63 (d, J = 36.6 Hz, Ar), 137.08 (d, J = 49.7 Hz, Ar), 134.72 (s, Ar), 134.62 (s, Ar), 132.96 (s, Ar), 132.88 (s, Ar), 131.21 (s, Ar), 130.80 (d, J = 6.2 Hz, Ar), 130.26 (d, J = 2.1 Hz, Ar), 129.50 (d, J = 1.7 Hz, Ar), 129.22 (s, Ar), 128.72 (s, Ar), 128.65 (s, Ar), 128.61 (s, Ar), 128.53 (s, Ar), 127.41 (s, Ar), 126.40 (s, Ar), 123.64 (s, Ar), 122.23 (d, J = 13.5 Hz, Ar), 118.10 (s, Ar), 117.67 (d, J = 6.7 Hz, Ar), 112.74 (d, J = 41.3 Hz, Ar), 68.03 (s, $CH_2\boldsymbol{C}H_2O\boldsymbol{C}H_2CH_2$), 35.84 (s, Ar***C***H$_2$Ar), 32.21 (d, J = 22.5 Hz, ***C***H$_2$P), 26.77 (d, J = 25.9 Hz, P***C***H(CH_3)$_2$), 26.18 (s,

C$H_2CH_2OCH_2$***C***H_2), 25.11 (d, J = 11.2 Hz, P***C***$H(CH_3)_2$), 19.68 (s, $PCH($***C***$H_3)_2$), 19.44 (d, J = 2.0 Hz, $PCH($***C***$H_3)_2$), 19.16 (d, J = 3.8 Hz, $PCH($***C***$H_3)_2$), 17.32 (s, $PCH($***C***$H_3)_2$).

IR (KBr) = 1937 cm^{-1} (CO).

HRMS (ESI): Exact mass calculated for $C_{33}H_{32}NOP_2Ru^+$ ($[M-THF-3H]^+$): 622.0997, mass found: 622.1028.

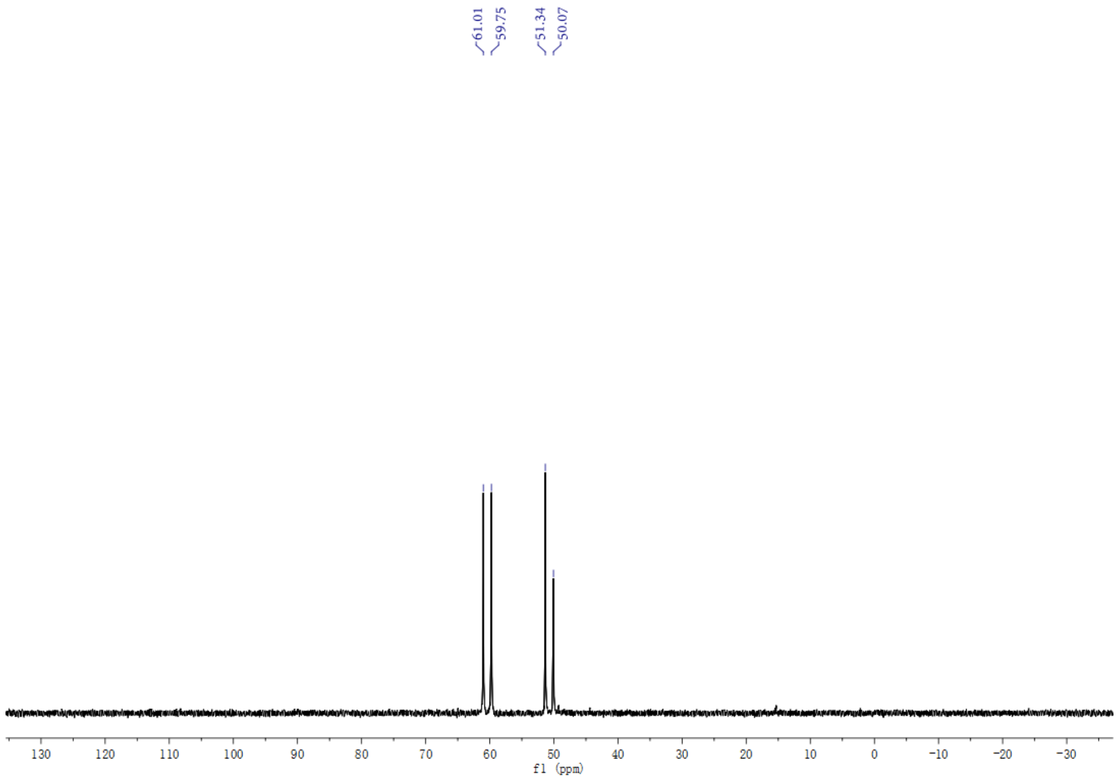

Figure S52. ^{31}P NMR (202 MHz, THF-d_8) spectrum of **LS-Ru-1**

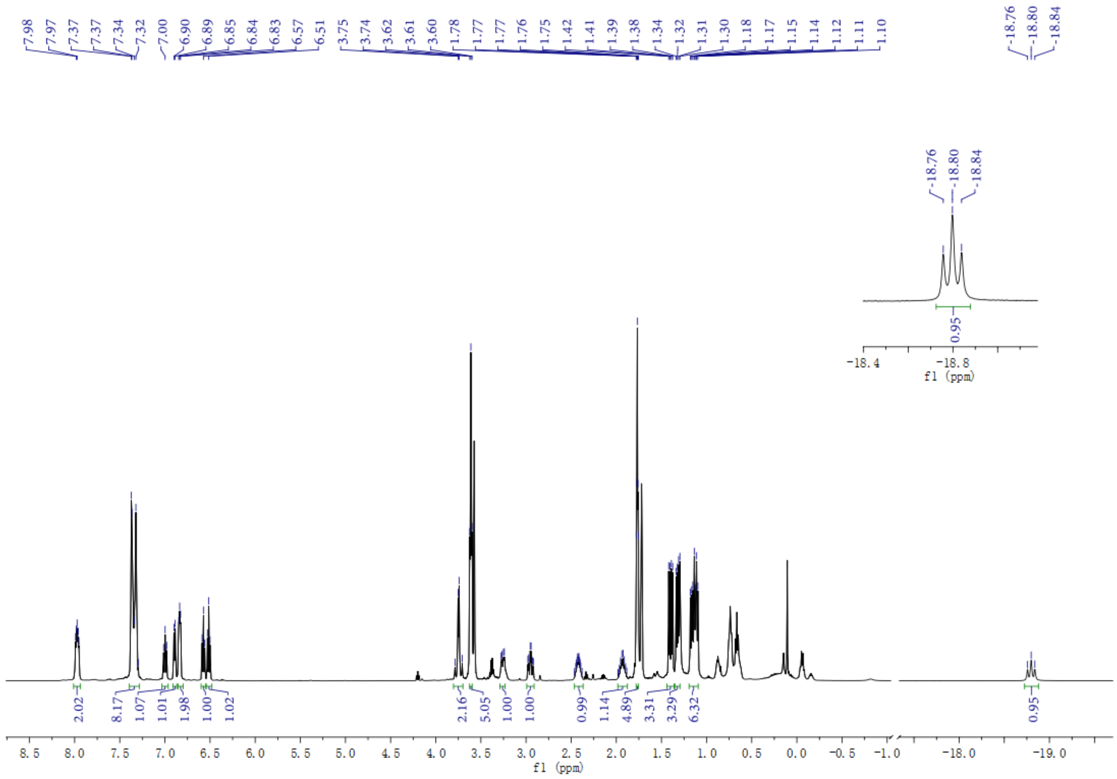

Figure S53. ^{1}H NMR (500 MHz, THF-d_8) spectrum of **LS-Ru-1-**

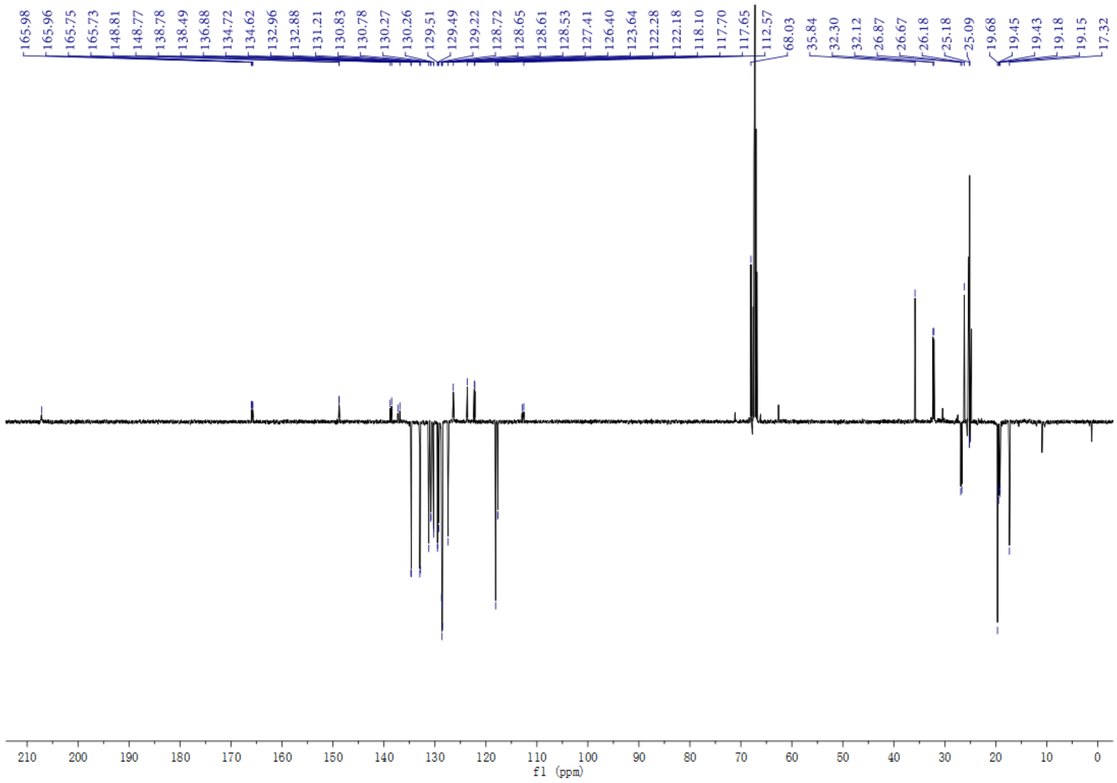

Figure S54. ^{13}C-DEPTQ NMR (126 MHz, THF-d_8) spectrum of **LS-Ru-1**

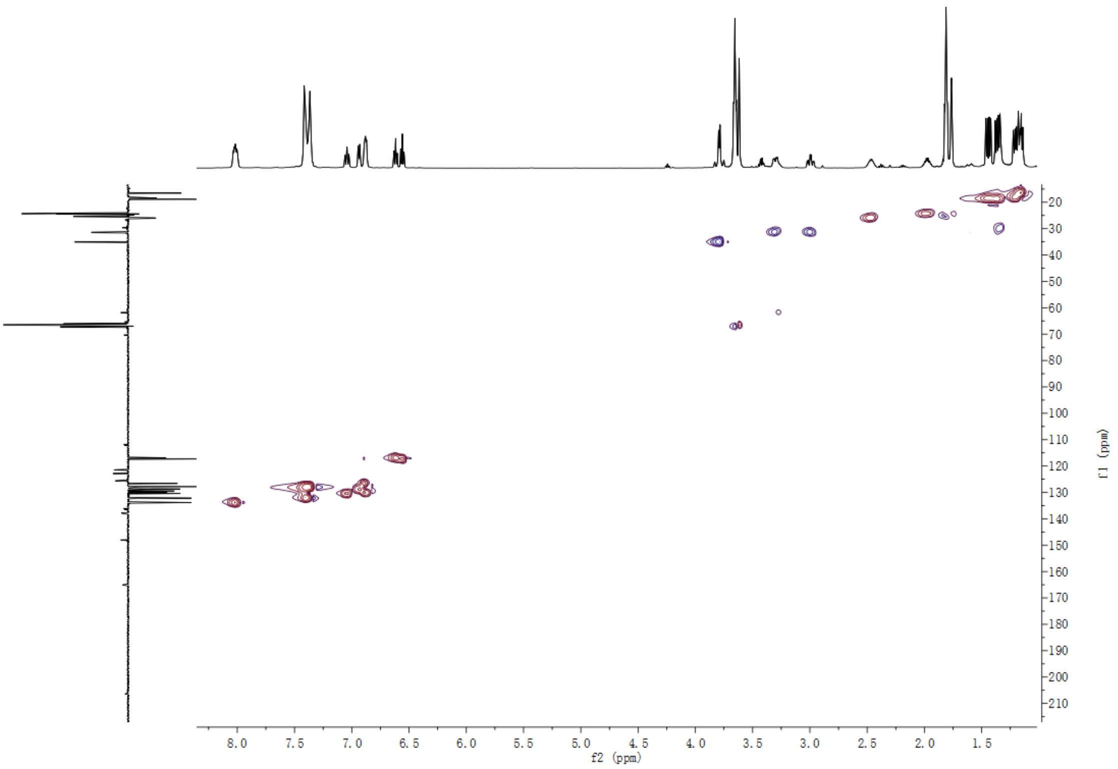

Figure S55. ^{1}H-^{13}C HSQC NMR (500 MHz, THF-d_8) spectrum of **LS-Ru-1**

The diffraction data from single crystals of **LS-Ru-1** were at 100 K on a Rigaku Synergy-S diffractometer dual source equipped with Dectris Pilatus3 R CdTe 300K detector, MoKα (λ=0.71073Å). All datasets were processed with CrysAlisPRO and structures were solved with SHELXT[9]. All non-hydrogen atoms were further refined by SHELXL[10] with anisotropic displacement coefficients. Hydrogens were placed in calculated positions and refined in a riding mode. Hydride atoms were located in the electron density map, incorporated and refined. Refinement was carried out with the OLEX-2[11] GUI. Crystallographic data and refinement parameters are summarized in Table S2.

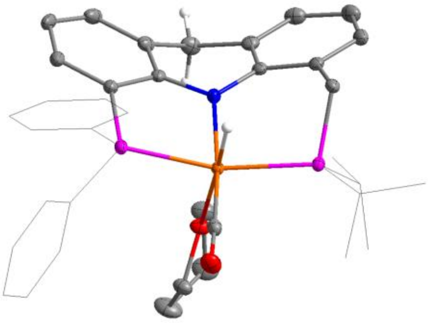

Figure S56. X-ray crystal structure of **LS-Ru-1**

Table S2. Crystallographic data for complex **LS-Ru-1**

**CCDC No.**	2382564
**Formula**	$2C_{37}H_{43}NO_2P_2Ru + C_4H_8O$
**Molecular weight**	1465.57
**Crystal system**	Monoclinic
**Space group**	$P2_1/c$
**Crystal size (mm)**	0.219×0.090×0.070
**Crystal color and shape**	Yellow prism
**Temperature (K)**	100
**Wavelength (Å)**	0.71073
**a (Å)**	12.54529(17)
**b (Å)**	29.8644(4)
**c (Å)**	18.5709(3)
**α (º)**	90
**β (º)**	91.1248(13)
**γ (º)**	90
**Volume (Å^3)**	6956.37(17)
**Z**	4
**r_{calcd} (g·cm^{-1})**	1.399
**μ (mm^{-1})**	0.579
**No. of reflections (unique)**	294958 (15950)
**R_{int}**	0.0553
**Completeness to θ (%)**	100.0
**θ max**	27.484
**Data / restraints / parameters**	15950 / 0 / 841
**Goodness-of-fit on F^2**	1.054
**Final R_1 and wR_2 indices [I>2s(I)]**	0.0310, 0.0720
**R_1 and wR_2 indices (all data)**	0.0354, 0.0735

Highest diff Peak and Deepest hole	0.861, -0.473

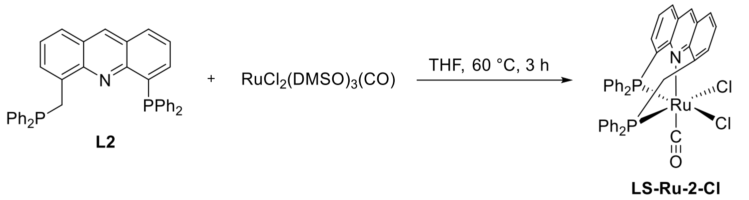

Synthesis of LS-Ru-2-Cl:

In a glovebox, **L2** (24.7 mg, 0.044 mmol) and $RuCl_2(CO)(DMSO)_3$ (17.4 mg, 0.04 mmol) were suspended in tetrahydrofuran (2.0 mL) in an oven-dried 15 mL sealed tube equipped with a magnetic stirring bar. The tube was sealed and taken out of the glovebox, and stirred at 60 ˚C for 3 hours. After cooling the reaction mixture to room temperature, the sealed tube was taken into the glovebox again and the solvent was removed under vacuum. The resulting orange solid was washed with ether for several times and dried under vacuum to give the desired product **LS-Ru-2-Cl** as an orange solid (27.4 mg, 90% yield).

^{31}P NMR (202 MHz, CD_2Cl_2) δ 56.33 (d, *J* = 21.5 Hz), 48.02 (d, *J* = 21.5 Hz).

^{1}H NMR (500 MHz, CD_2Cl_2) δ 9.06 (s, 1H, aryl), 8.17 – 8.08 (m, 4H, aryl), 7.95 – 7.88 (m, 3H, aryl), 7.76 (dd, *J* = 9.9, 6.9 Hz, 1H, aryl), 7.59 (t, *J* = 7.6 Hz, 2H, aryl), 7.55 – 7.46 (m, 6H, aryl), 7.02 – 6.96 (m, 2H, aryl), 6.72 (t, *J* = 7.0 Hz, 4H, aryl), 6.69 – 6.62 (m, 4H, aryl), 4.96 (vt, *J* = 14.6 Hz, 1H, C***H***$_2$P), 3.27 (vt, *J* = 12.8 Hz, 1H, C***H***$_2$P).

^{13}C NMR (126 MHz, CD_2Cl_2) δ 203.58 (t, *J* = 13.5 Hz, Ru-***C***O), 156.53 (d, *J* = 16.1 Hz, Ar), 150.92 (s, Ar), 142.66 (s, Ar), 137.54 (s, Ar), 137.10 (d, *J* = 10.4 Hz, Ar), 136.39 (s, Ar), 135.67 (d, *J* = 11.4 Hz, Ar), 135.24 (d, *J* = 4.2 Hz, Ar), 134.90 (s, Ar), 134.79 (s, Ar), 134.36 (s, Ar), 133.98 (s, Ar), 133.59 (s, Ar), 132.89 (d, *J* = 9.4 Hz, Ar), 131.86 (d, *J* = 2.0 Hz, Ar), 131.64 (d, *J* = 9.9 Hz, Ar), 131.18 (s, Ar), 130.59 (d, *J* = 2.0 Hz, Ar), 130.49 (d, *J* = 9.2 Hz, Ar), 129.97 (d, *J* = 1.6 Hz, Ar), 129.70 (d, *J* = 1.9 Hz, Ar), 129.30 (s, Ar), 128.98 (s, Ar), 128.89 (d, *J* = 2.5 Hz, Ar), 128.69 (d, *J* = 1.3 Hz, Ar), 128.61 (s, Ar), 128.53 (s, Ar), 127.02 (s, Ar), 126.91 (d, *J* = 7.6 Hz, Ar), 126.12 (d, *J* = 7.3 Hz, Ar), 33.27 (d, *J* = 33.0 Hz, ***C***H$_2$P).

IR (KBr) = 1958 cm^{-1} (CO).

HRMS (ESI): Exact mass calculated for $C_{39}H_{28}NOP_2Ru^+$ ([M-2Cl-H]$^+$): 690.0684, mass found: 690.0703

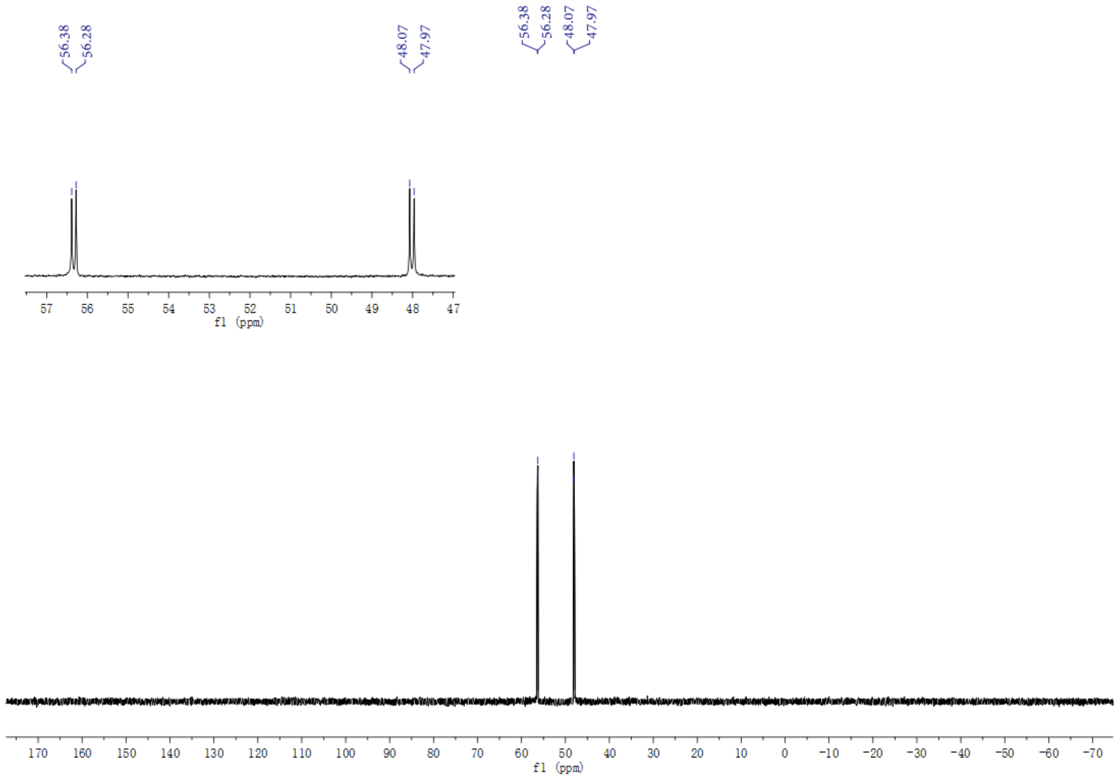

Figure S57. ^{31}P NMR (202 MHz, CD_2Cl_2) spectrum of **LS-Ru-2-Cl**

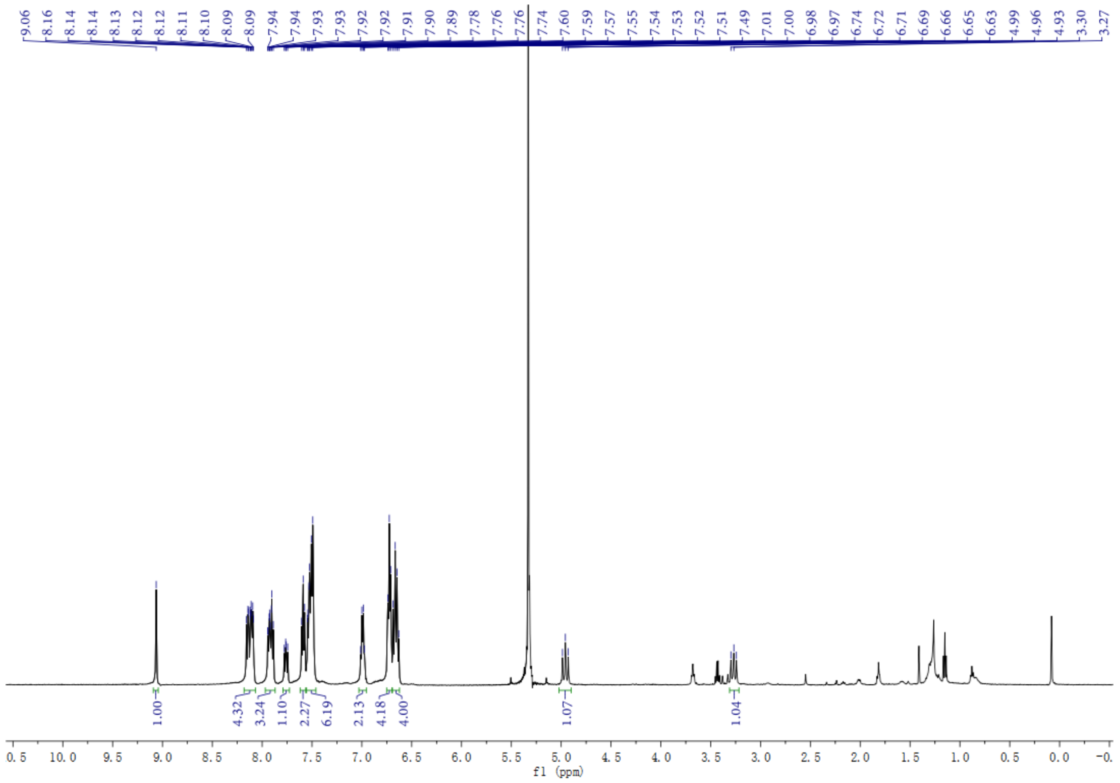

Figure S58. ^{1}H NMR (500 MHz, CD_2Cl_2) spectrum of **LS-Ru-2-Cl**

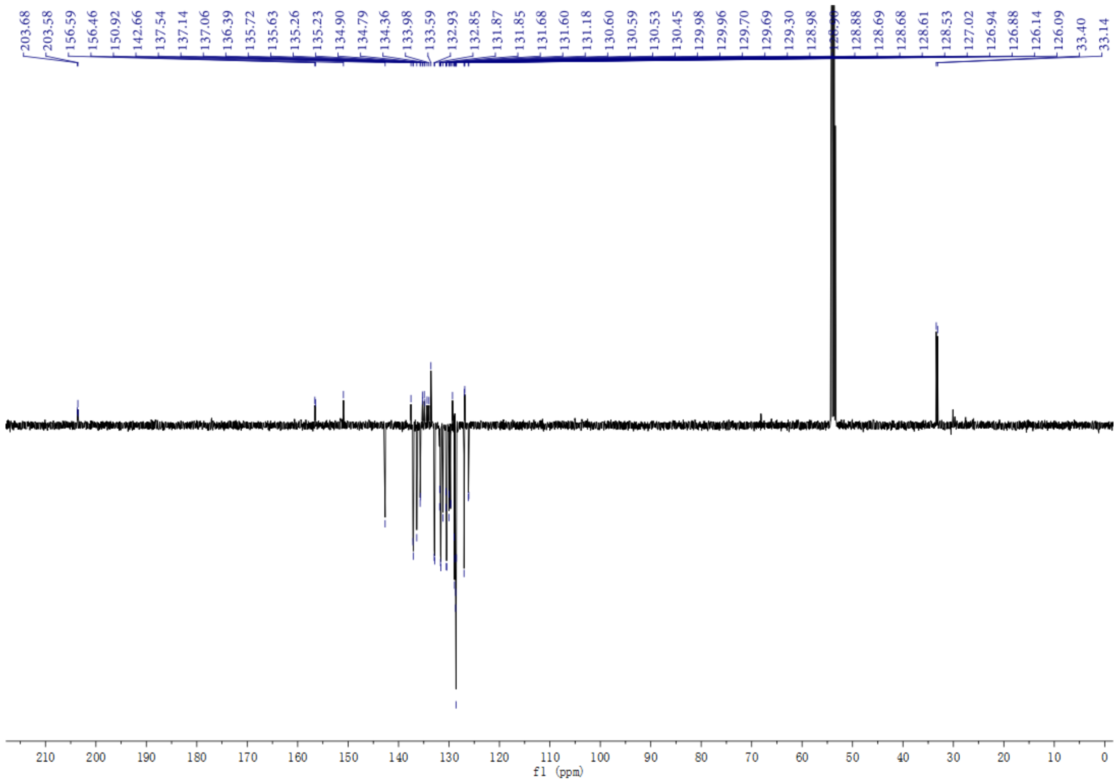

Figure S59. ^{13}C-DEPTQ NMR (126 MHz, CD_2Cl_2) spectrum of **LS-Ru-2-Cl**

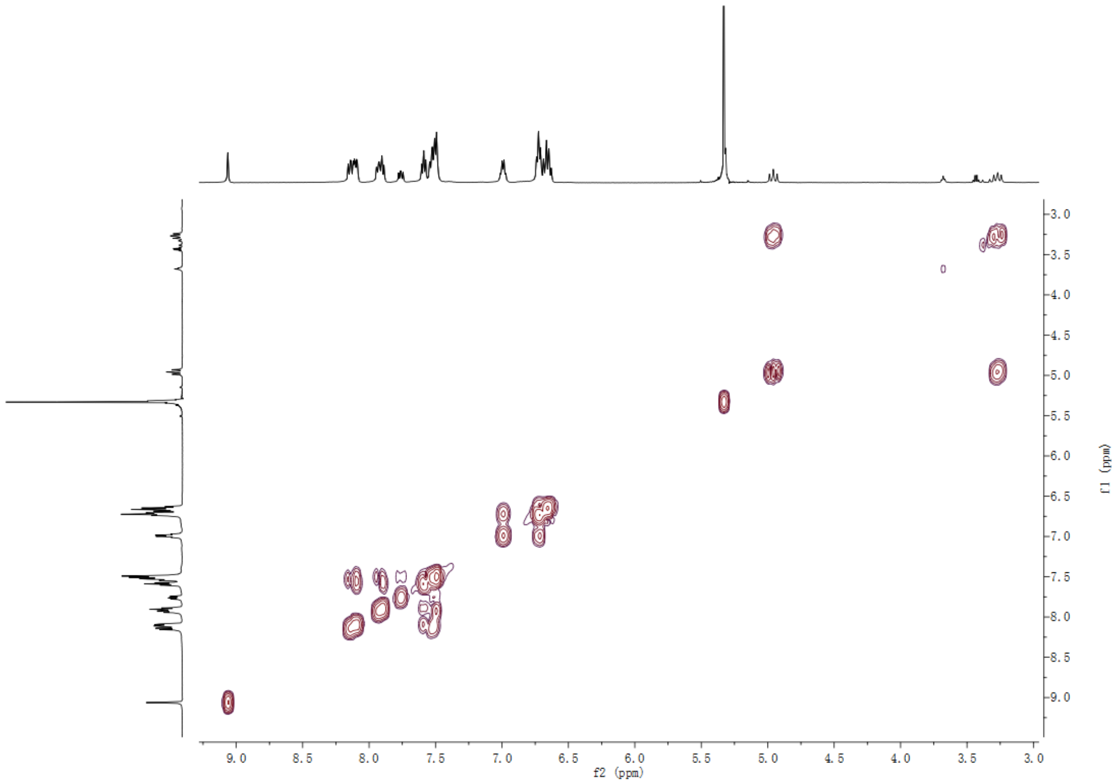

Figure S60. ^{1}H-^{1}H COSY NMR (500 MHz, CD_2Cl_2) spectrum of **LS-Ru-2-Cl**

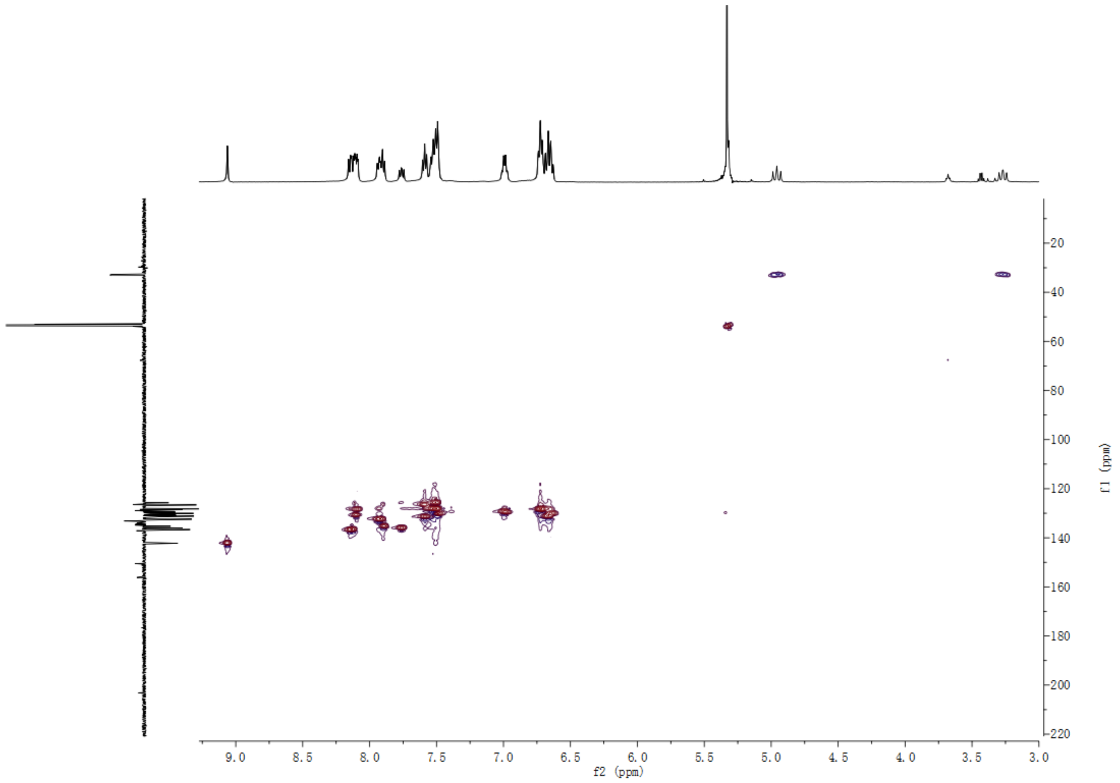

Figure S61. ^{1}H-^{13}C HSQC NMR (500 MHz, CD_2Cl_2) spectrum of **LS-Ru-2-Cl**

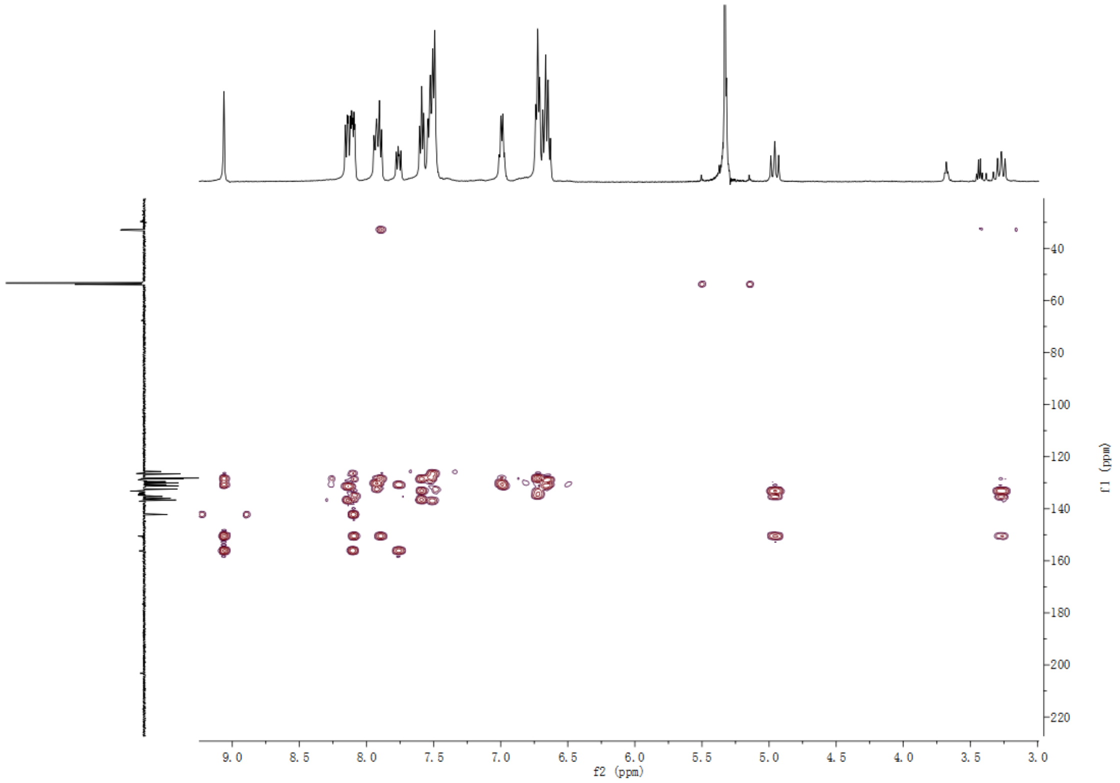

Figure S62. ^{1}H-^{13}C HMBC NMR (500 MHz, CD_2Cl_2) spectrum of **LS-Ru-2-Cl**

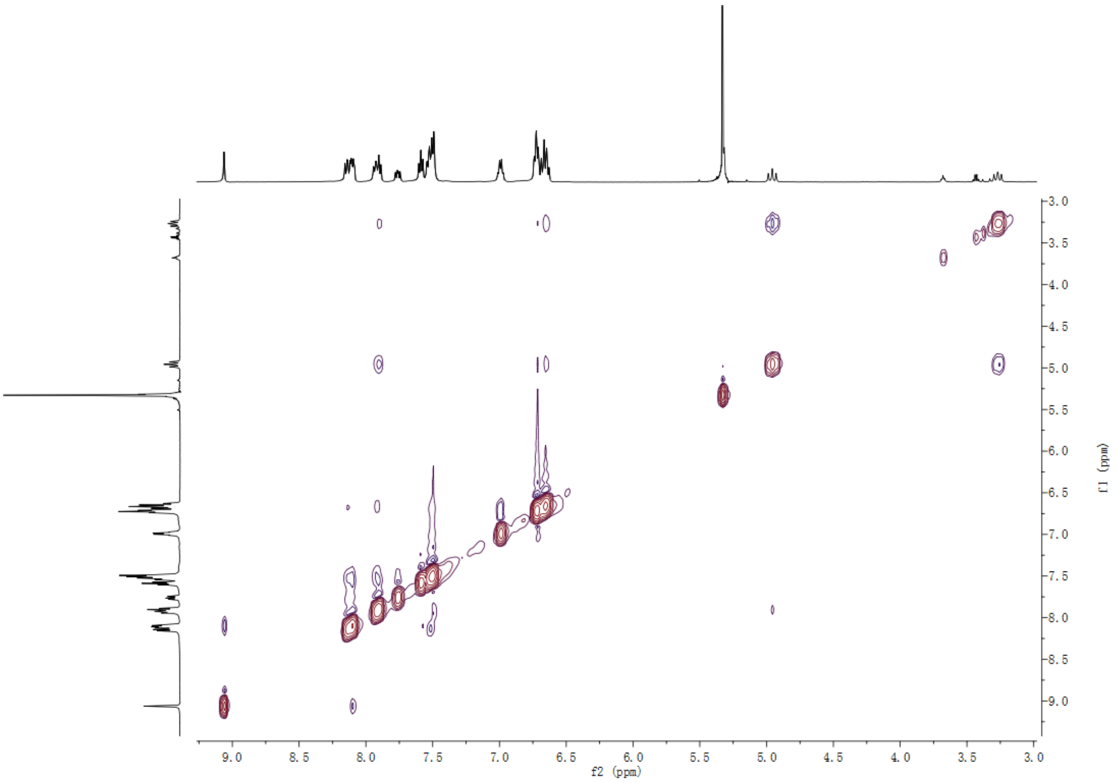

Figure S63. ^{1}H-^{1}H NOESY NMR (500 MHz, CD_2Cl_2) spectrum of **LS-Ru-2-Cl**

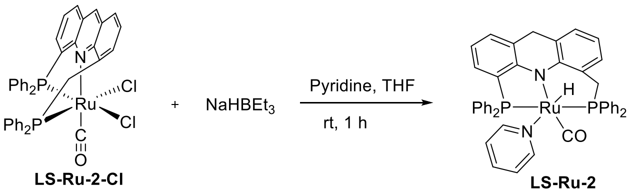

Synthesis of LS-Ru-2:

A THF solution of $NaBEt_3H$ was prepared by adding 42 µl of 1.0 M $NaBEt_3H$ (0.042 mmol) in THF to 0.4 mL of THF. The afforded solution was added to a stirring suspension of **LS-Ru-2-Cl** (16.0 mg, 0.021 mmol) and pyridine (3.3 mg, 0.042 mmol) in 2.5 mL of THF. The resulting mixture was stirred at room temperature for 1 hour. The solvent was then removed under vacuum. The residue was redissolved in a mixture of THF (4.0 mL) and diethyl ether (2.0 mL), before passed through a 0.2 µm PTFE filter. The solvent was then removed under vacuum and the residue was washed twice with 1.0 mL of n-pentane to remove a dark impurity. The remaining solid was dried under vacuum, affording **LS-Ru-2** as a yellow solid (15.1 mg; 93% yield).

^{31}P NMR (121 MHz, THF-d_8) δ 56.87 (d, J = 255.3 Hz), 45.21 (d, J = 255.2 Hz).

^{1}H NMR (400 MHz, THF-d_8) δ 8.27 – 8.17 (m, 2H, aryl), 7.90 (d, J = 5.1 Hz, 2H, aryl), 7.71 – 7.64

(m, 2H, aryl), 7.48 – 7.41 (m, 3H, aryl), 7.38 – 7.31 (m, 3H, aryl), 7.28 (t, *J* = 7.4 Hz, 1H, aryl), 7.20 – 7.12 (m, 3H, aryl), 7.12 – 7.01 (m, 3H, aryl), 6.99 – 6.92 (m, 4H, aryl), 6.91 – 6.81 (m, 4H, aryl), 6.63 – 6.57 (m, 2H, aryl), 6.54 – 6.47 (m, 2H, aryl), 3.90 (dd, *J* = 13.6, 6.7 Hz, 1H, C$\boldsymbol{H}_2$P), 3.82 – 3.71 (m, 3H, C$\boldsymbol{H}_2$P, ArC$\boldsymbol{H}_2$Ar), -13.25 (t, *J* = 20.3 Hz, 1H, Ru-***H***).

^{13}C NMR (126 MHz, THF-d_8) δ 206.68 (t, *J* = 13.8 Hz, Ru-***C***O), 164.94 (dd, *J* = 27.1, 2.0 Hz, Ar), 153.38 (s, Ar), 148.75 (d, *J* = 5.6 Hz, Ar), 140.01 (d, *J* = 45.5 Hz, Ar), 137.42 (d, *J* = 40.7 Hz, Ar), 136.73 (d, *J* = 51.3 Hz, Ar), 136.00 (s, Ar), 135.36 (s, Ar), 135.26 (s, Ar), 134.23 (s, Ar), 134.15 (s, Ar), 133.34 (d, *J* = 4.1 Hz, Ar), 133.10 (s, Ar), 133.02 (s, Ar), 132.51 (s, Ar), 132.45 (s, Ar), 132.33 (s, Ar), 132.25 (s, Ar), 131.17 (s, Ar), 130.61 (s, Ar), 129.88 (d, *J* = 1.2 Hz, Ar), 129.78 (s, Ar), 129.31 (s, Ar), 128.89 (s, Ar), 128.82 (s, Ar), 128.54 (s, Ar), 128.47 (s, Ar), 127.91 (s, Ar), 126.72 (s, Ar), 124.62 (s, Ar), 122.83 (d, *J* = 13.6 Hz, Ar), 122.67 (s, Ar), 118.38 (s, Ar), 117.99 (d, *J* = 6.7 Hz, Ar), 113.19 (d, *J* = 41.6 Hz, Ar), 38.44 (d, *J* = 27.0 Hz, ***C***H_2P), 36.33 (s, Ar***C***H_2Ar).

IR (KBr) = 1914 cm^{-1} (CO).

HRMS (ESI): Exact mass calculated for $C_{39}H_{28}NOP_2Ru^+$ ([M-Pyridine-3H]$^+$): 690.0684, mass found: 690.0715.

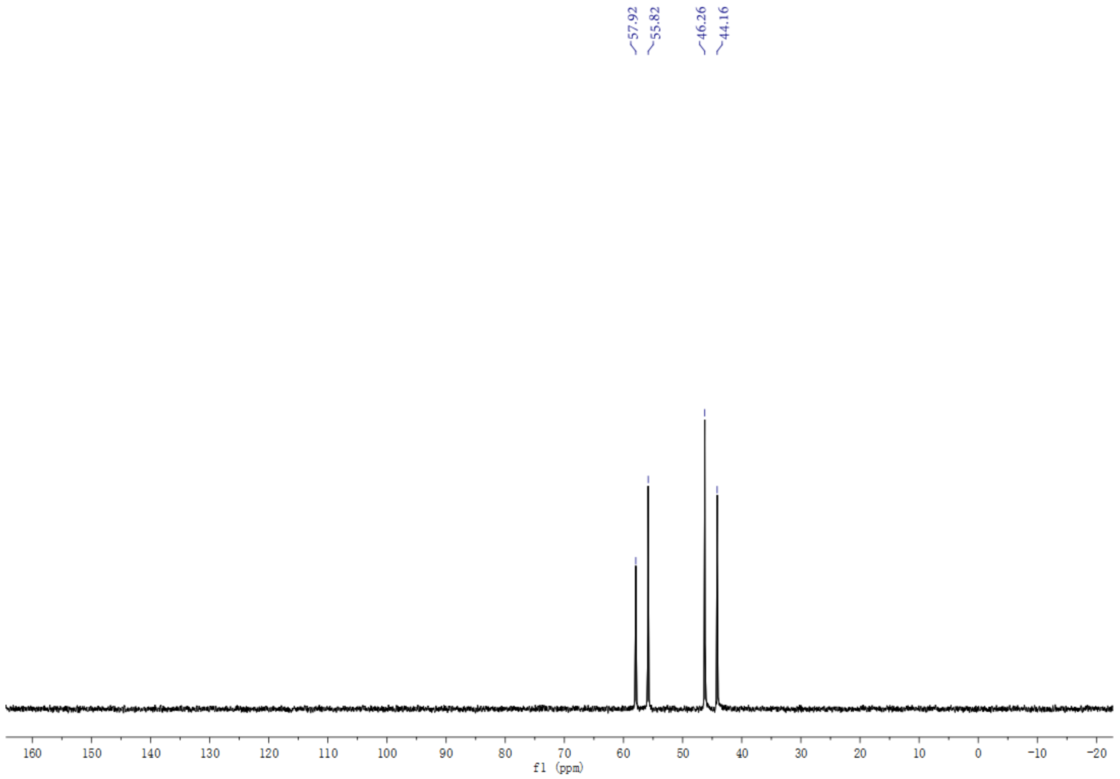

Figure S64. ^{31}P NMR (121 MHz, THF-d_8) spectrum of **LS-Ru-2**

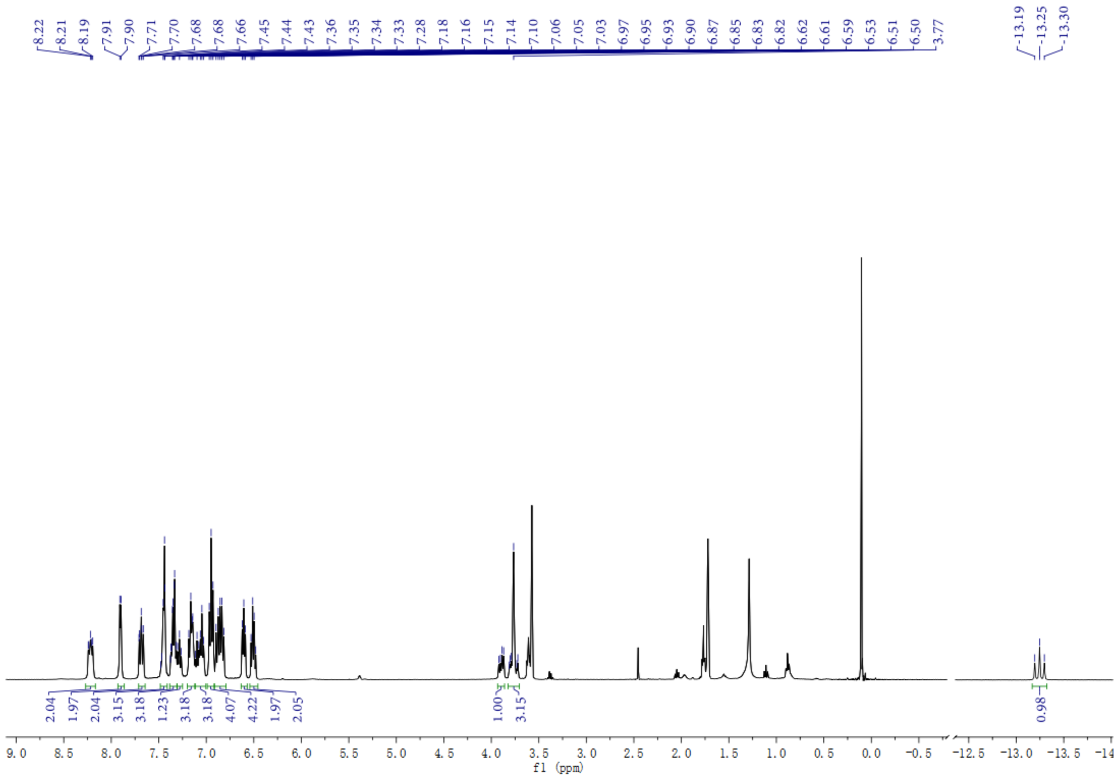

Figure S65. ^{1}H NMR (400 MHz, THF-d_8) spectrum of **LS-Ru-2**

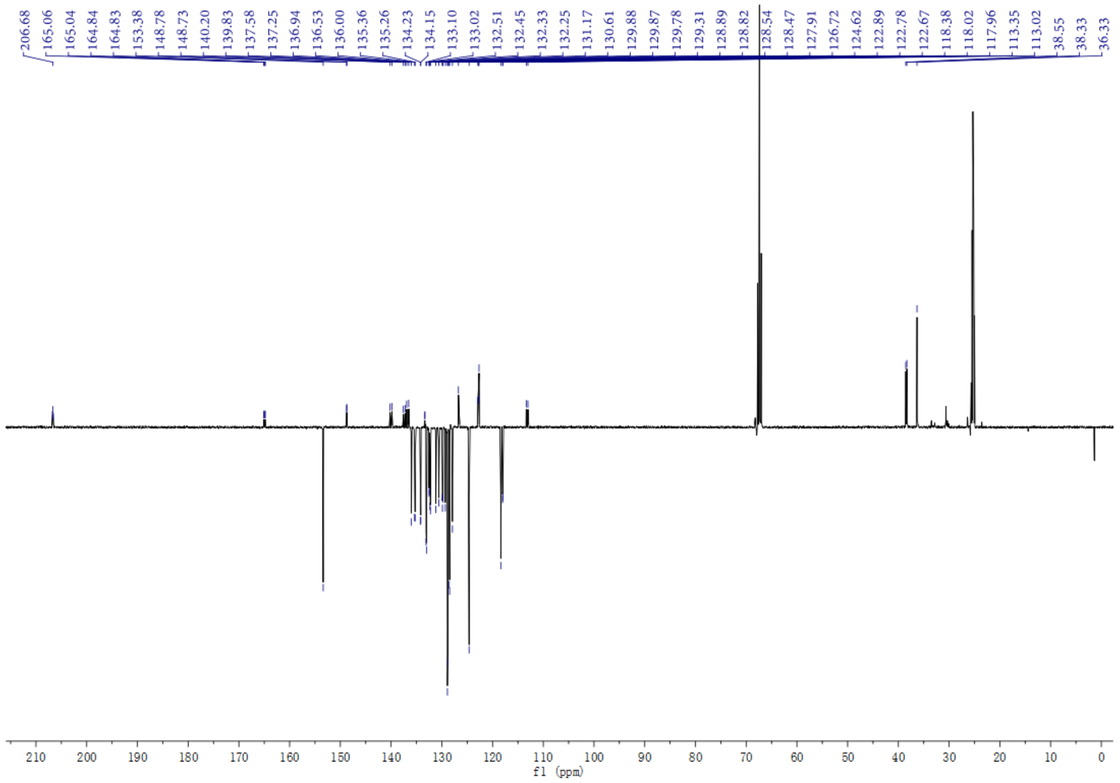

Figure S66. ^{13}C-DEPTQ NMR (126 MHz, THF-d_8) spectrum of **LS-Ru-2**

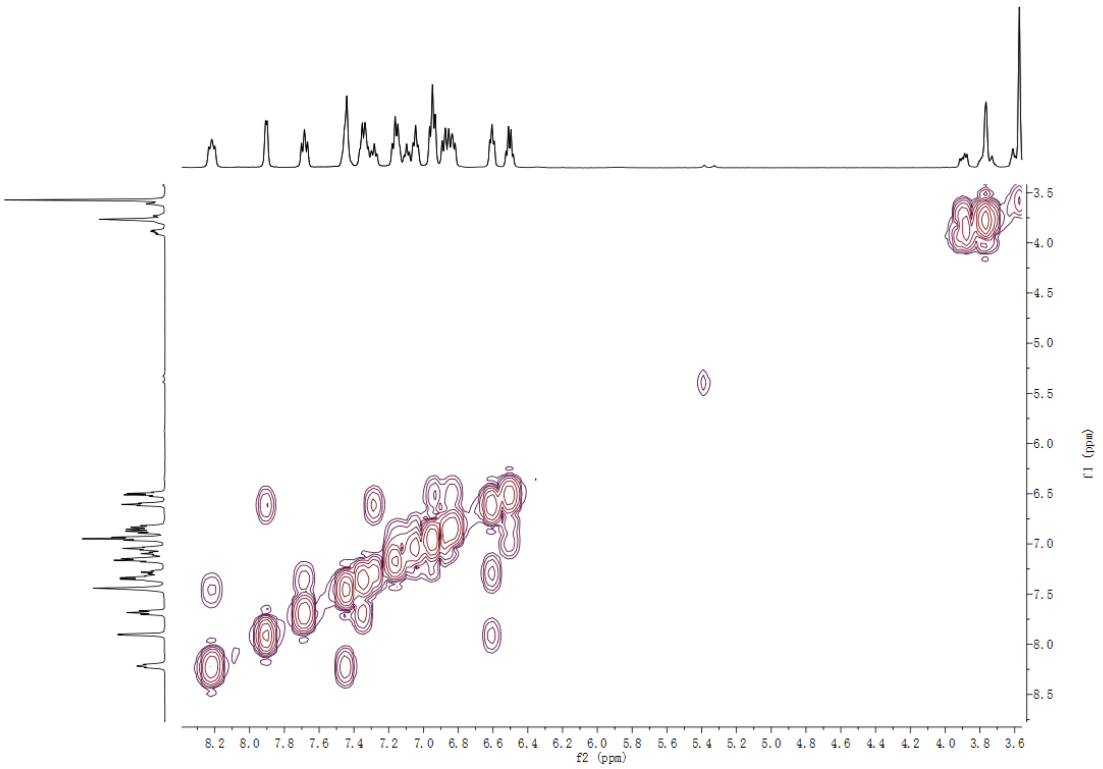

Figure S67. ^{1}H-^{1}H COSY NMR (500 MHz, THF-d_8) spectrum of **LS-Ru-2**

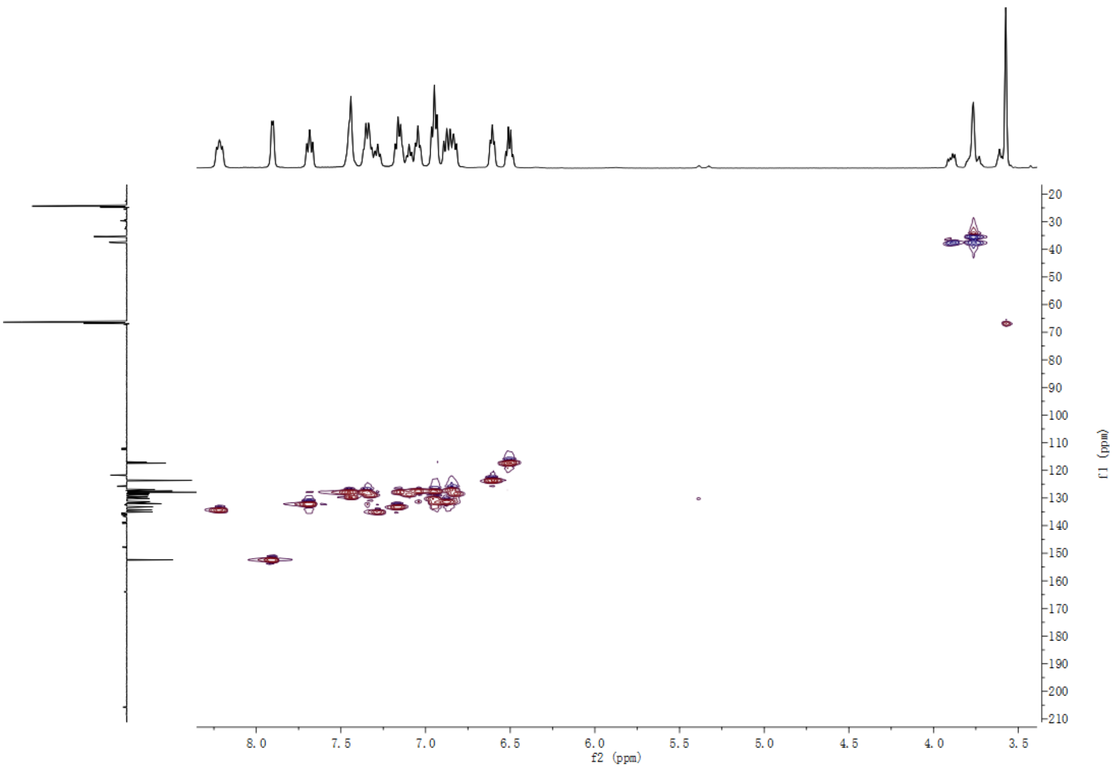

Figure S68. ^{1}H-^{13}C HSQC NMR (500 MHz, THF-d_8) spectrum of **LS-Ru-2**

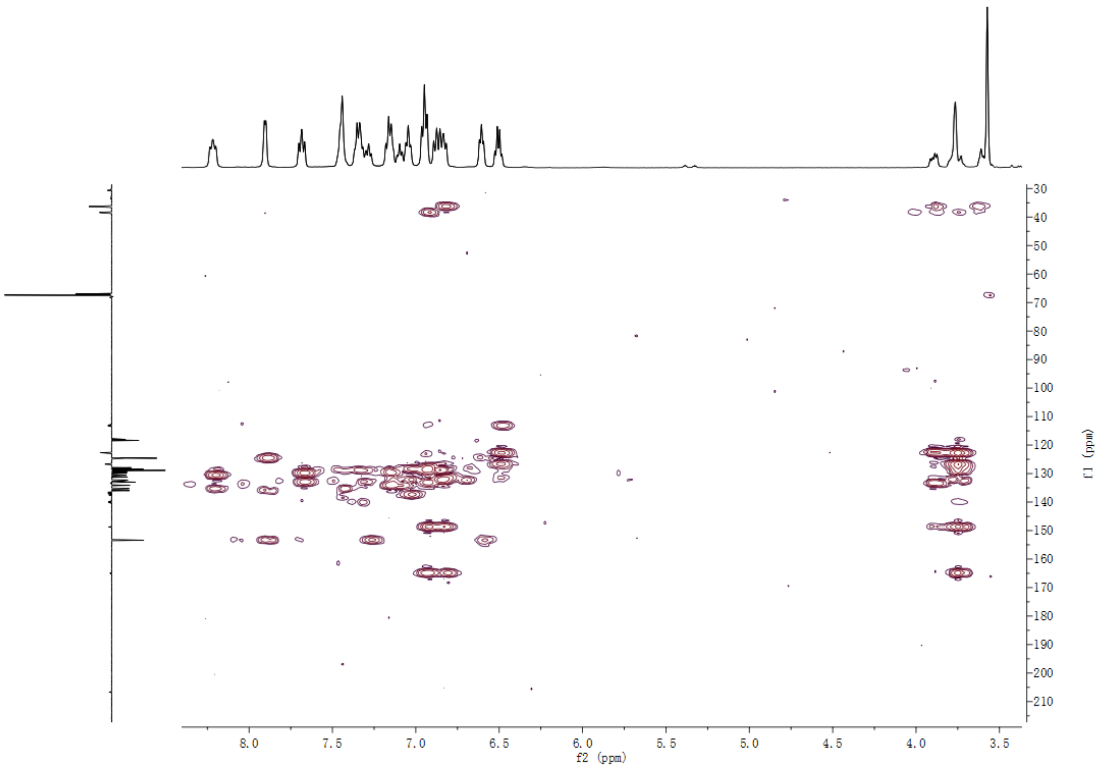

Figure S69. ^{1}H-^{13}C HMBC NMR (500 MHz, THF-d_8) spectrum of **LS-Ru-2**

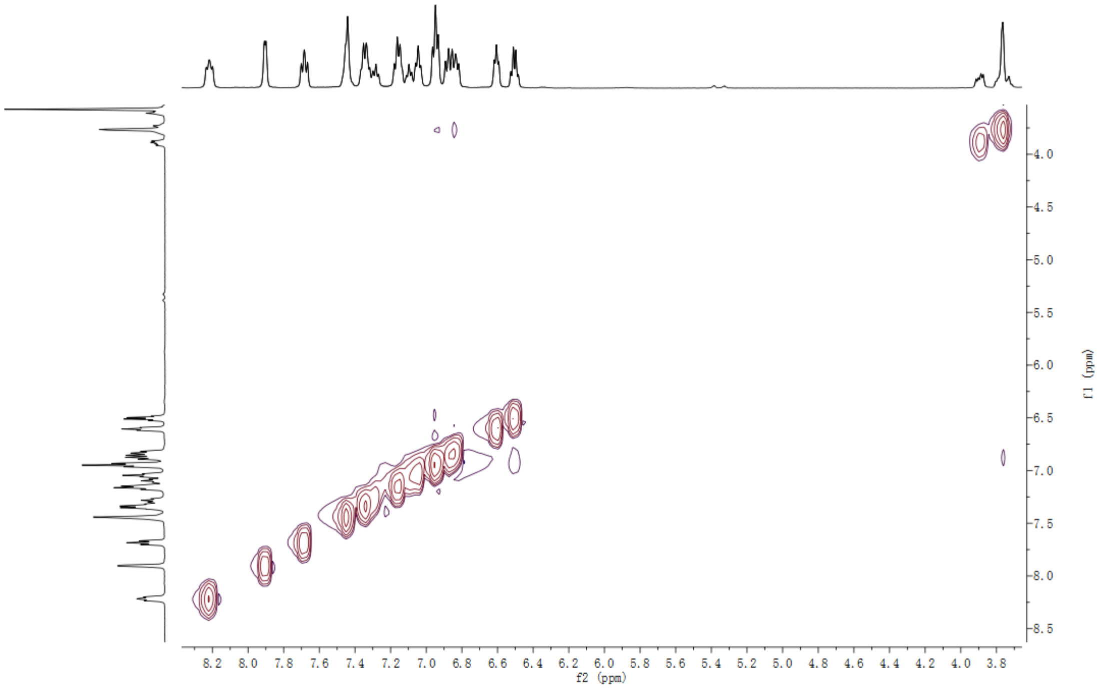

Figure S70. ^{1}H-^{1}H NOESY NMR (500 MHz, THF-d_8) spectrum of **LS-Ru-2**

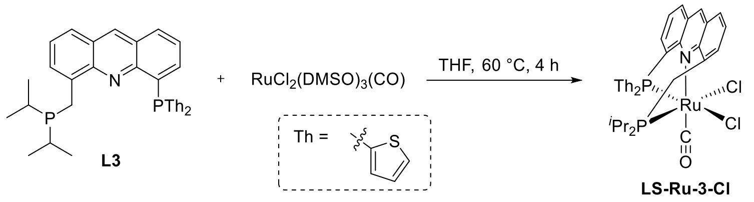

Synthesis of LS-Ru-3-Cl:

In a glovebox, **L3** (80.9 mg, 0.16 mmol) and $RuCl_2(CO)(DMSO)_3$ (56.5 mg, 0.13 mmol) were suspended in tetrahydrofuran (6.0 mL) in an oven-dried 100 mL Schlenk flask equipped with a magnetic stirring bar. The flask was sealed and taken out of the glovebox, and stirred at 60 ˚C for 4 hours. After cooling the reaction mixture to room temperature, the Schlenk flask was taken into the glovebox again and the solvent was removed under vacuum. The resulting orange solid was washed with ether for several times and dried under vacuum to give the desired product **LS-Ru-3-Cl** as an orange solid (88.0 mg, 96% yield). Crystals suitable for X-ray analysis were obtained from a dichloromethane/THF solution of **LS-Ru-3-Cl** at -40 ˚C.

^{31}P NMR (202 MHz, CD_2Cl_2) δ 57.03 (d, *J* = 24.7 Hz), 38.30 (d, *J* = 24.7 Hz).

^{1}H NMR (500 MHz, CD_2Cl_2) δ 9.04 (s, 1H, aryl), 8.21 – 8.13 (m, 3H, aryl), 8.03 (d, *J* = 8.3 Hz, 1H, aryl), 7.86 (d, *J* = 6.9 Hz, 1H, aryl), 7.82 (t, *J* = 3.8 Hz, 1H, aryl), 7.72 – 7.68 (m, 1H, aryl), 7.65 (t, *J* = 7.6 Hz, 1H, aryl), 7.58 (t, *J* = 7.7 Hz, 1H, aryl), 7.26 – 7.22 (m, 1H, aryl), 7.21 – 7.16 (m, 1H, aryl), 7.11 (t, *J* = 4.0 Hz, 1H, aryl), 4.70 (dd, *J* = 16.6, 11.7 Hz, 1H, C***H***$_2$P), 2.92 (dd, *J* = 16.6, 12.0 Hz, 1H, C***H***$_2$P), 2.62 – 2.48 (m, 1H, PC***H***$(CH_3)_2$), 1.66 (dd, *J* = 15.8, 7.1 Hz, 3H, PCH(C***H***$_3)_2$), 1.39 (dd, *J* = 16.4, 7.1 Hz, 3H, PCH(C***H***$_3)_2$), 1.08 – 0.99 (m, 1H, PC***H***$(CH_3)_2$), 0.90 (dd, *J* = 12.2, 6.9 Hz, 3H, PCH(C***H***$_3)_2$), -0.22 (dd, *J* = 14.9, 7.0 Hz, 3H, PCH(C***H***$_3)_2$).

^{13}C NMR (126 MHz, CD_2Cl_2) δ 154.66 (s, Ar), 154.50 (s, Ar), 149.57 (s, Ar), 142.09 (s, Ar), 141.16 (d, *J* = 11.4 Hz, Ar), 137.28 (s, Ar), 136.19 (d, *J* = 9.3 Hz, Ar), 135.51 (d, *J* = 11.1 Hz, Ar), 135.03 (s, Ar), 132.97 (s, Ar), 131.69 (s, Ar), 128.73 (s, Ar), 128.19 (s, Ar), 127.93 (s, Ar), 127.85 (s, Ar), 127.76 (s, Ar), 126.35 (s, Ar), 125.50 (d, *J* = 7.9 Hz, Ar), 27.76 (d, *J* = 29.3 Hz, P***C***H$(CH_3)_2$), 25.06 (d, *J* = 24.8 Hz, P***C***H$(CH_3)_2$), 23.99 (d, *J* = 28.6 Hz, ***C***H$_2$P), 18.71 (s, PCH(***C***H$_3)_2$), 18.49 (s, PCH(***C***H$_3)_2$), 18.09 (s, PCH(***C***H$_3)_2$), 17.50 (d, *J* = 6.4 Hz, PCH(***C***H$_3)_2$).

IR (KBr) = 1961 cm^{-1} (CO).

HRMS (ESI): Exact mass calculated for $C_{29}H_{28}NOP_2S_2Ru^+$ ($[M\text{-}2Cl\text{-}H]^+$): 634.0126, mass found: 634.0149; $C_{29}H_{29}NOP_2S_2ClRu^+$ ($[M\text{-}Cl]^+$): 669.9892, mass found: 669.9918.

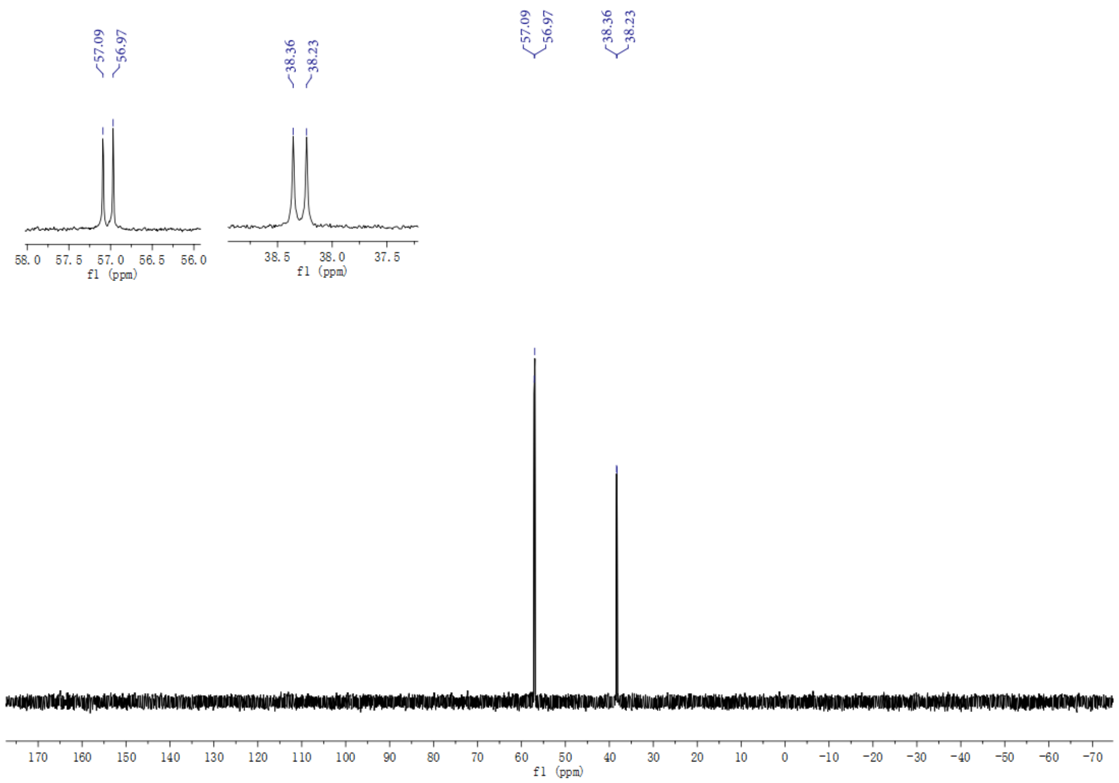

Figure S71. ^{31}P NMR (202 MHz, CD_2Cl_2) spectrum of **LS-Ru-3-Cl**

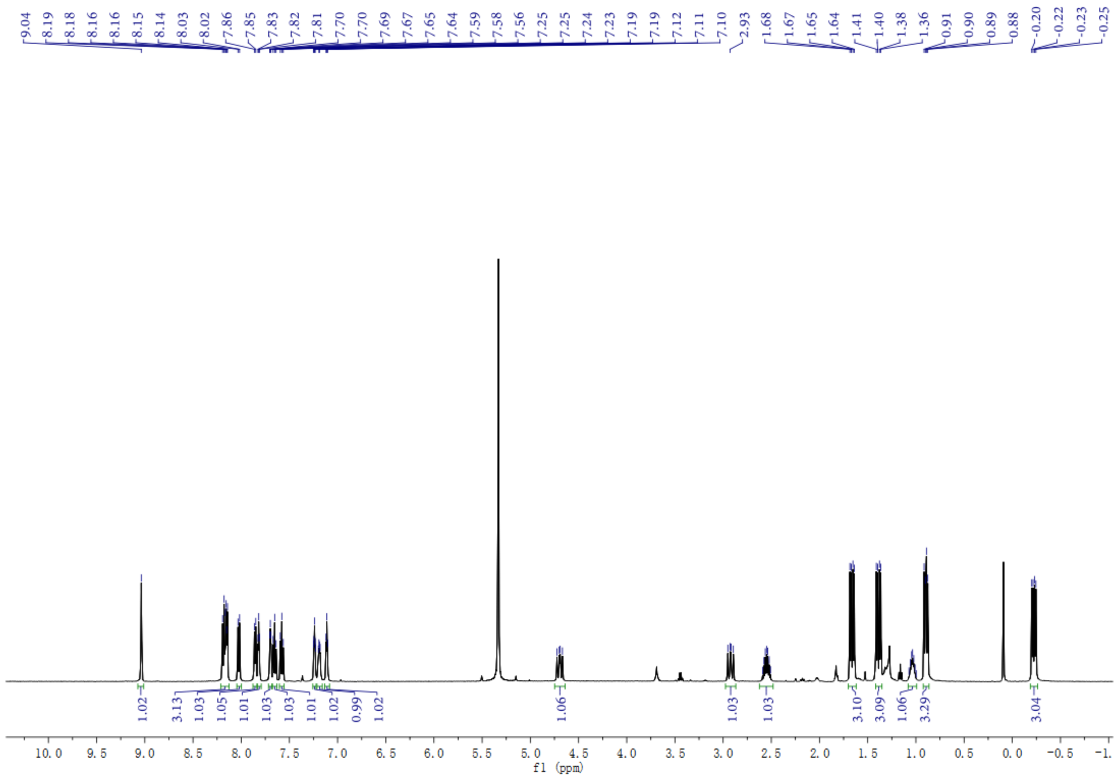

Figure S72. ^{1}H NMR (500 MHz, CD_2Cl_2) spectrum of **LS-Ru-3-Cl**

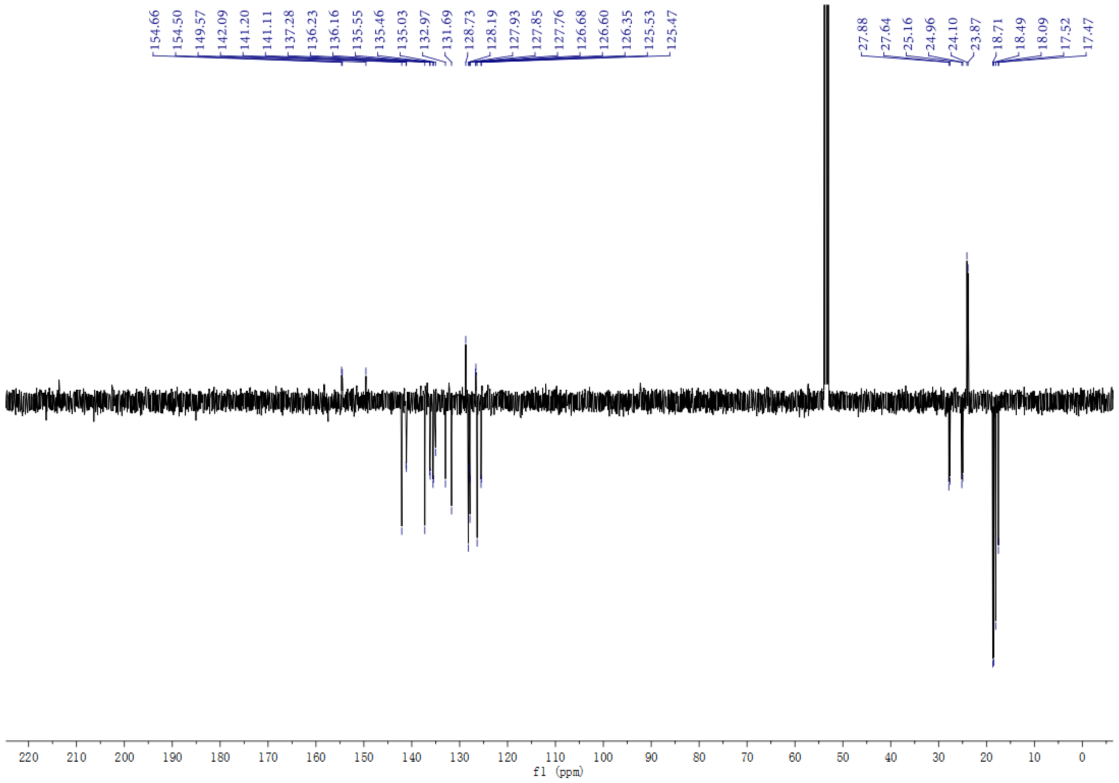

Figure S73. ^{13}C-DEPTQ NMR (126 MHz, CD_2Cl_2) spectrum of **LS-Ru-3-Cl** (CO ligand peaks not observed due to the low solubility of the complex in CD_2Cl_2)

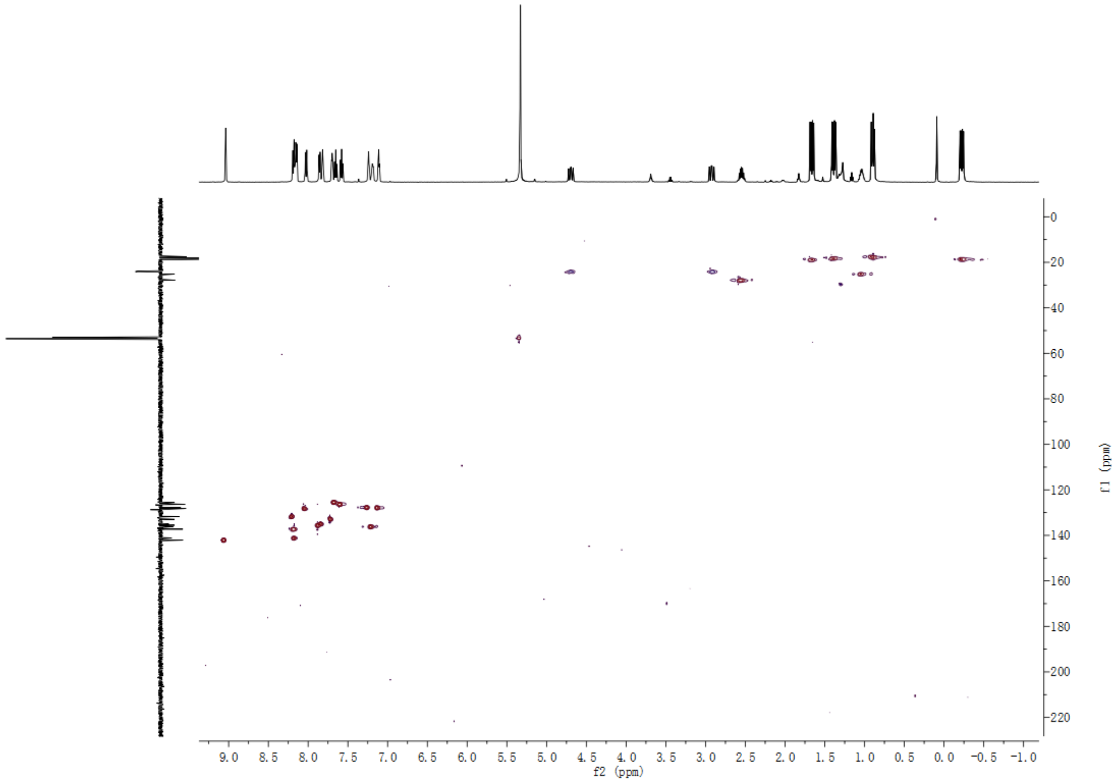

Figure S74. ^{1}H-^{13}C HSQC NMR (500 MHz, CD_2Cl_2) spectrum of **LS-Ru-3-Cl**

The diffraction data from single crystals of **LS-Ru-3-Cl** were at 100 K on Rigaku Xtalab Pro dual source equipped Pilatus 200K detector MoKα (λ=0.71073Å). All datasets were processed with CrysAlisPRO and structures were solved with SHELXT[9]. All non-hydrogen atoms were further refined by SHELXL[10] with anisotropic displacement coefficients. Hydrogens were placed in calculated positions and refined in a riding mode. Hydride atoms were located in the electron density map, incorporated and refined. Refinement was carried out with the OLEX-2[11] GUI. Crystallographic data and refinement parameters are summarized in Table S3.

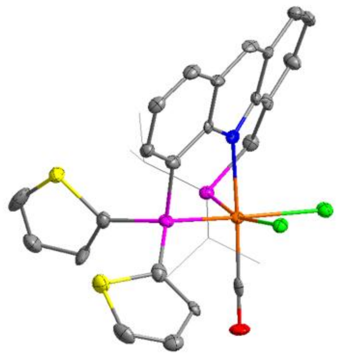

Figure S75. X-ray crystal structure of **LS-Ru-3-Cl**

Table S3. Crystallographic data for complex **LS-Ru-3-Cl**

**CCDC No.**	2382560
**Formula**	$C_{29}H_{29}Cl_2NOP_2RuS_2 + CH_2Cl_2$
**Molecular weight**	790.49
**Crystal system**	Triclinic
**Space group**	$P\bar{1}$
**Crystal size (mm)**	0.123×0.044×0.031
**Crystal color and shape**	Yellow prism
**Temperature (K)**	100
**Wavelength (Å)**	0.71073
**a (Å)**	10.7807(7)
**b (Å)**	11.5170(10)
**c (Å)**	15.2302(9)

**α (º)**	86.011(6)
**β (º)**	75.374(5)
**γ (º)**	63.999(8)
**Volume (Å^3)**	1642.7(2)
**Z**	2
**r_{calcd} (g · cm^{-1})**	1.598
**μ (mm^{-1})**	1.053
**No. of reflections (unique)**	17282 (5968)
**R_{int}**	0.0943
**Completeness to θ (%)**	99.3
**θ max**	25.348
**Data / restraints / parameters**	5968 / 0 / 393
**Goodness-of-fit on F^2**	1.019
**Final R_1 and wR_2 indices [I>2s(I)]**	0.0580, 0.1238
**R_1 and wR_2 indices (all data)**	0.1106, 0.1462
Highest diff Peak and Deepest hole	0.944, -0.831

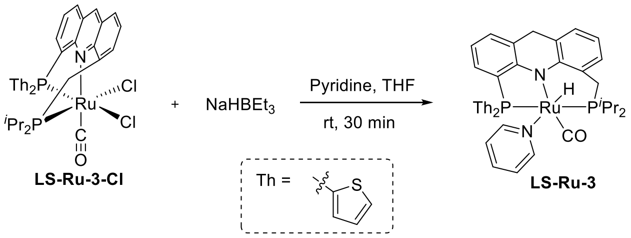

Synthesis of LS-Ru-3:

A THF solution of $NaBEt_3H$ was prepared by adding 200 μl of 1.0 M $NaBEt_3H$ (0.20 mmol) in THF to 1.8 mL of THF. The afforded solution was added to a stirring suspension of **LS-Ru-3-Cl** (70.5 mg, 0.10 mmol) and pyridine (15.8 mg, 0.20 mmol) in 8.0 mL of THF. The resulting mixture was stirred at room temperature for 30 minutes. The solvent was then removed under vacuum. The residue was redissolved in 10 mL diethyl ether, before passed through a 0.2 μm PTFE filter. Diethyl ether was then removed under vacuum and the residue was washed twice with 2 mL of n-pentane

to remove a dark impurity. The remaining solid was dried under vacuum, affording **LS-Ru-3** as a yellow solid (64.1 mg; 91% yield).

^{31}P NMR (202 MHz, THF-d_8) δ 58.42 (d, *J* = 261.2 Hz), 34.60 (d, *J* = 261.1 Hz).

^{1}H NMR (500 MHz, THF-d_8) δ 8.30 (d, *J* = 5.0 Hz, 2H, aryl), 7.78 (dd, *J* = 6.7, 3.4 Hz, 1H, aryl), 7.76 – 7.73 (m, 1H, aryl), 7.41 (t, *J* = 7.4 Hz, 1H, aryl), 7.38 – 7.33 (m, 1H, aryl), 7.26 – 7.21 (m, 1H, aryl), 7.17 (t, *J* = 4.1 Hz, 1H, aryl), 6.91 (d, *J* = 7.1 Hz, 1H, aryl), 6.87 – 6.82 (m, 4H, aryl), 6.82 – 6.79 (m, 1H, aryl), 6.65 – 6.60 (m, 1H, aryl), 6.57 – 6.50 (m, 2H, aryl), 3.91 (d, *J* = 16.8 Hz, 1H, ArC***H***$_2$Ar), 3.80 (d, *J* = 16.9 Hz, 1H, ArC***H***$_2$Ar), 3.44 – 3.40 (m, 1H, C***H***$_2$P), 2.97 (td, *J* = 12.9, 5.0 Hz, 1H, C***H***$_2$P), 2.51 – 2.41 (m, 1H, PC***H***(CH$_3$)$_2$), 1.84 – 1.75 (m, 1H, PC***H***(CH$_3$)$_2$), 1.44 – 1.37 (m, 6H, PCH(C***H***$_3$)$_2$), 1.01 (dd, *J* = 10.6, 7.2 Hz, 3H, PCH(C***H***$_3$)$_2$), 0.59 (dd, *J* = 14.3, 7.4 Hz, 3H, PCH(C***H***$_3$)$_2$), -13.87 (dd, *J* = 22.9, 19.7 Hz, 1H, Ru-***H***).

^{13}C NMR (126 MHz, THF-d_8) δ 207.45 (t, *J* = 13.8 Hz, Ru-***C***O), 164.59 (d, *J* = 30.8 Hz, Ar), 153.07 (s, Ar), 148.44 (d, *J* = 4.7 Hz, Ar), 141.13 (d, *J* = 53.1 Hz, Ar), 139.41 (d, *J* = 40.7 Hz, Ar), 136.76 (d, *J* = 9.9 Hz, Ar), 136.36 (s, Ar), 133.77 (d, *J* = 9.0 Hz, Ar), 133.41 (d, *J* = 3.4 Hz, Ar), 131.21 (d, *J* = 6.3 Hz, Ar), 131.07 (s, Ar), 130.72 (s, Ar), 129.70 (s, Ar), 128.63 (d, *J* = 10.9 Hz, Ar), 127.92 (d, *J* = 10.0 Hz, Ar), 127.63 (s, Ar), 127.11 (s, Ar), 124.98 (s, Ar), 123.77 (s, Ar), 122.61 (d, *J* = 14.6 Hz, Ar), 118.51 (s, Ar), 117.78 (d, *J* = 7.1 Hz, Ar), 114.50 (d, *J* = 44.6 Hz, Ar), 36.06 (d, *J* = 1.7 Hz, Ar***C***H$_2$Ar), 33.69 (d, *J* = 23.6 Hz, ***C***H$_2$P), 27.03 (d, *J* = 25.6 Hz, P***C***H(CH$_3$)$_2$), 24.53 (dd, *J* = 11.4, 4.9 Hz, P***C***H(CH$_3$)$_2$), 20.06 (s, PCH(***C***H$_3$)$_2$), 19.77 (d, *J* = 2.9 Hz, PCH(***C***H$_3$)$_2$), 19.08 (d, *J* = 5.3 Hz, PCH(***C***H$_3$)$_2$), 17.00 (d, *J* = 2.1 Hz, PCH(***C***H$_3$)$_2$).

IR (KBr) = 1907 cm^{-1} (CO).

HRMS (ESI): Exact mass calculated for $C_{29}H_{28}NOP_2RuS_2^+$ ([M-Pyridine-3H]$^+$): 634.0126, mass found: 634.0151.

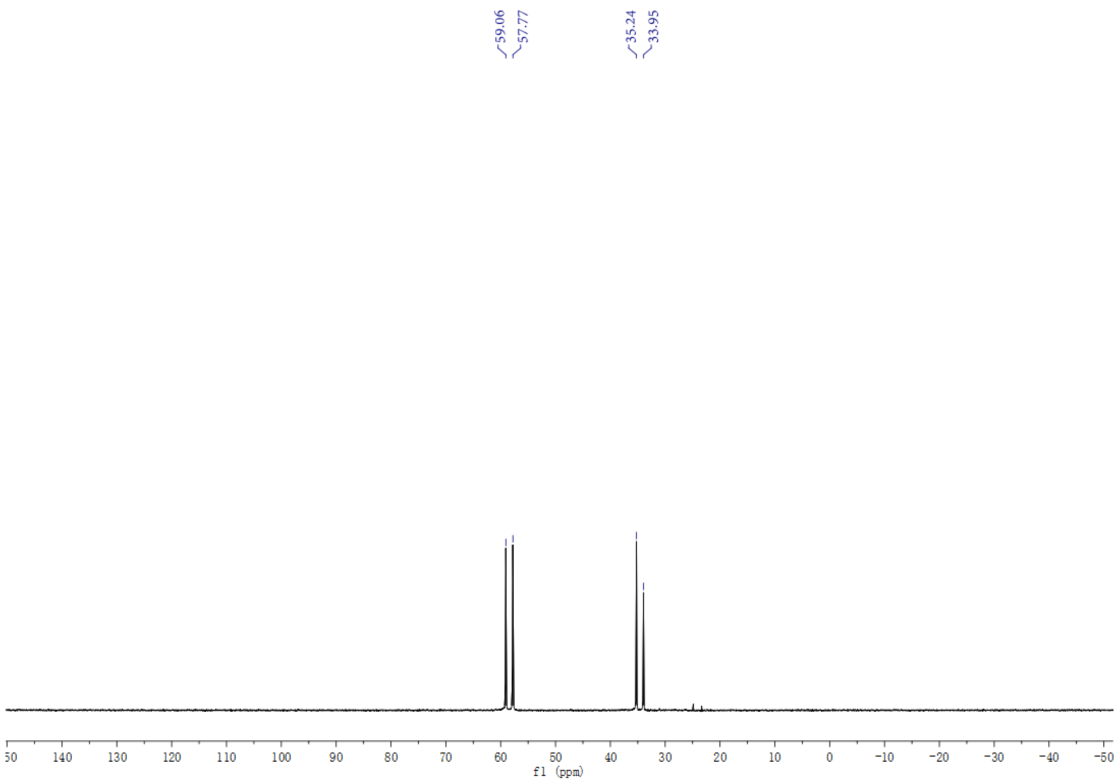

Figure S76. ^{31}P NMR (202 MHz, THF-d_8) spectrum of **LS-Ru-3**

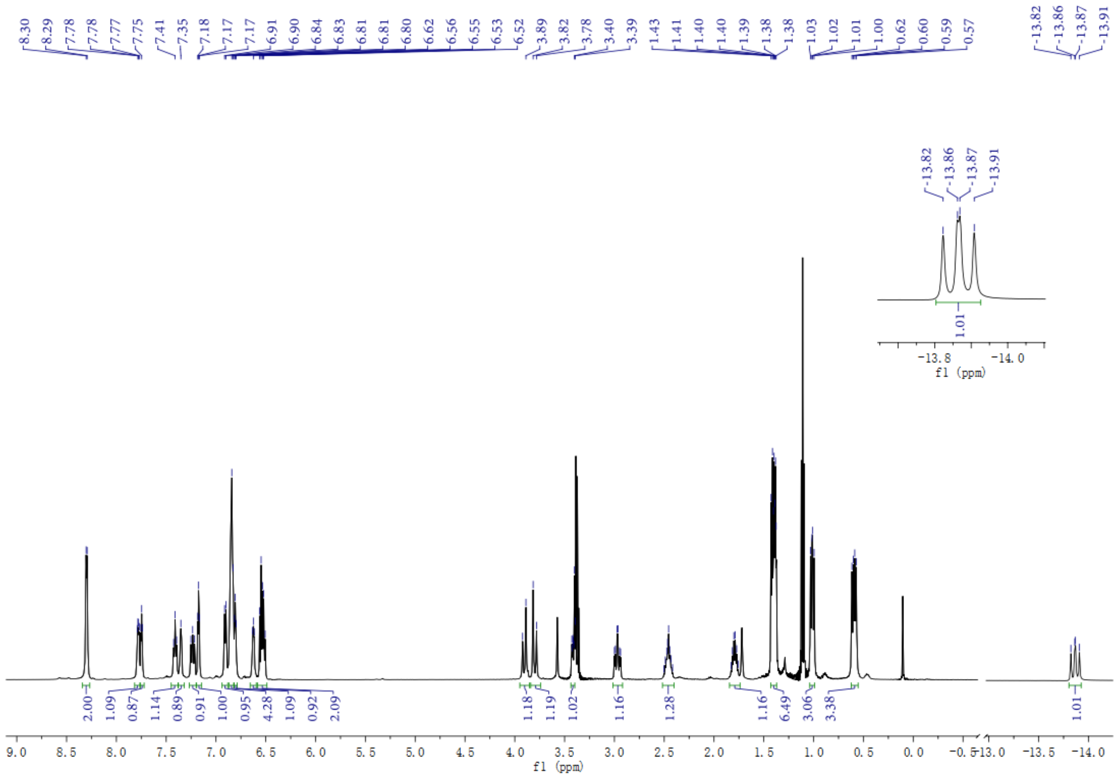

Figure S77. ^{1}H NMR (500 MHz, THF-d_8) spectrum of **LS-Ru-3**

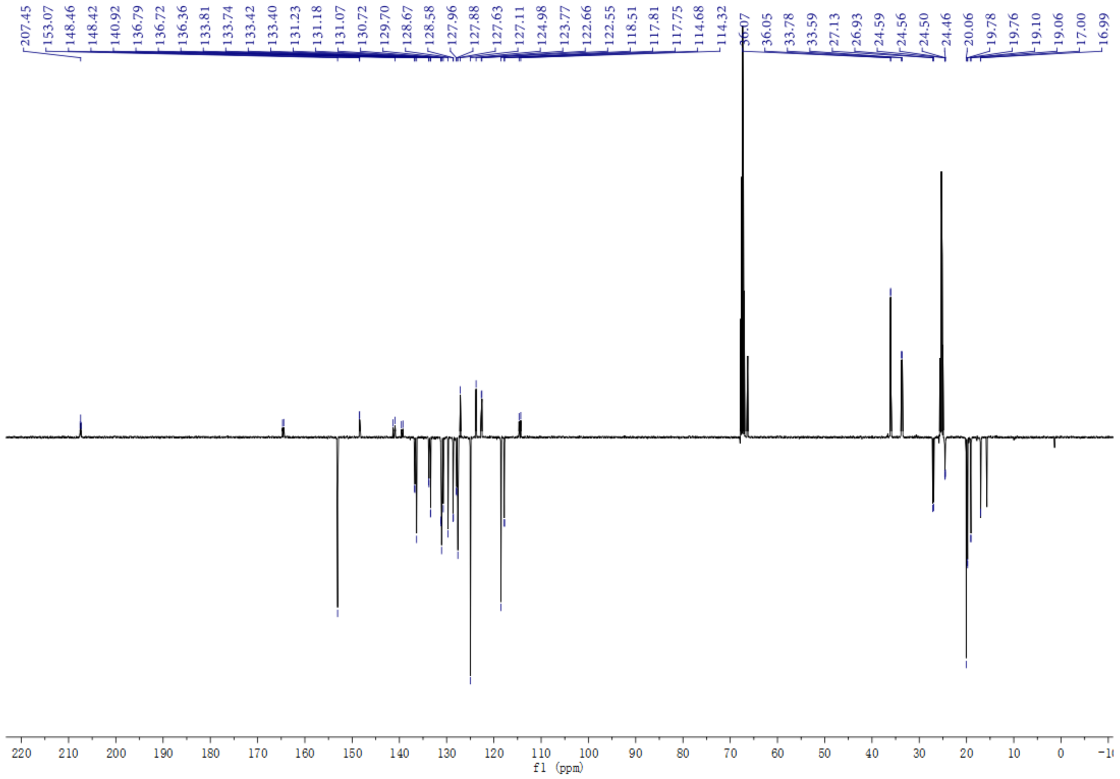

Figure S78. ^{13}C-DEPTQ NMR (126 MHz, THF-d_8) spectrum of **LS-Ru-3**

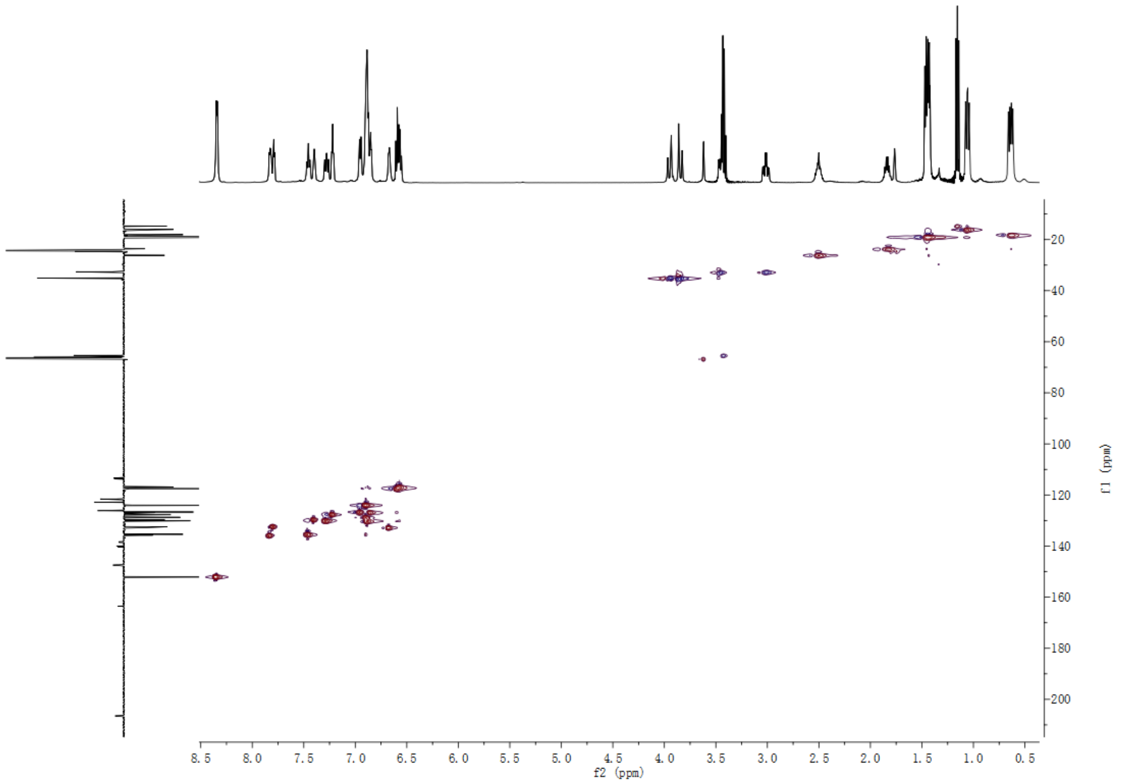

Figure S79. ^{1}H-^{13}C HSQC NMR (500 MHz, THF-d_8) spectrum of **LS-Ru-3**

The diffraction data from single crystals of **LS-Ru-3** were at 100 K on a Rigaku Synergy-S diffractometer dual source equipped with Dectris Pilatus3 R CdTe 300K detector, MoKα (λ=0.71073Å). All datasets were processed with CrysAlisPRO and structures were solved with SHELXT[9]. All non-hydrogen atoms were further refined by SHELXL[10] with anisotropic displacement coefficients. Hydrogens were placed in calculated positions and refined in a riding mode. Hydride atoms were located in the electron density map, incorporated and refined. Refinement was carried out with the OLEX-2[11] GUI. Crystallographic data and refinement parameters are summarized in Table S4.

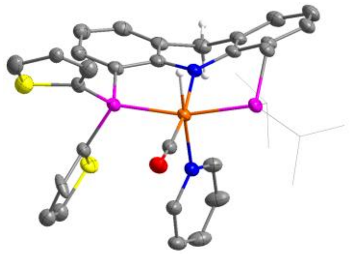

Figure S80. X-ray crystal structure of **LS-Ru-3**

Table S4. Crystallographic data for complex **LS-Ru-3**

**CCDC No.**	2382561
**Formula**	$2C_{34}H_{36}N_2OP_2RuS_2 + C_4H_{10}O$
**Molecular weight**	1505.67
**Crystal system**	Monoclinic
**Space group**	$I2_a$
**Crystal size (mm)**	0.334×0.156×0.151
**Crystal color and shape**	Yellow prism
**Temperature (K)**	100
**Wavelength (Å)**	0.71073
**a (Å)**	24.3111(7)
**b (Å)**	14.5463(3)
**c (Å)**	22.3501(7)

**α (º)**	90
**β (º)**	119.466(4)
**γ (º)**	90
**Volume (Å³)**	6881.4(4)
**Z**	4
**r_{calcd} (g·cm⁻¹)**	1.453
**μ (mm⁻¹)**	0.704
**No. of reflections (unique)**	59269 (11373)
**R_{int}**	0.0457
**Completeness to θ (%)**	99.1
**θ max**	31.505
**Data / restraints / parameters**	11373 / 67 / 487
**Goodness-of-fit on F^2**	1.064
**Final R_1 and wR_2 indices [I>2s(I)]**	0.0337, 0.0834
**R_1 and wR_2 indices (all data)**	0.0448, 0.0876
Highest diff Peak and Deepest hole	1.188, -0.798

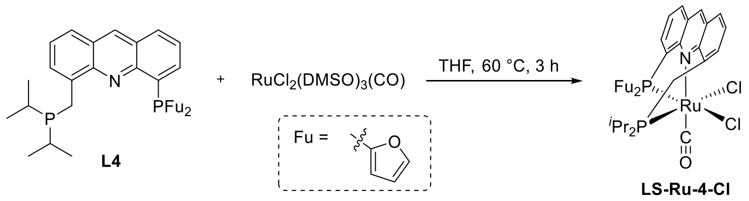

Synthesis of LS-Ru-4-Cl:

In a glovebox, **L4** (25.5 mg, 0.054 mmol) and $RuCl_2(CO)(DMSO)_3$ (21.3 mg, 0.049 mmol) were suspended in tetrahydrofuran (4.0 mL) in an oven-dried 15 mL sealed tube equipped with a magnetic stirring bar. The tube was sealed and taken out of the glovebox, and stirred at 60 ˚C for 3 hours. After cooling the reaction mixture to room temperature, the sealed tube was taken into the glovebox again and the solvent was removed under vacuum. The resulting orange solid was washed with ether for several times and dried under vacuum to give the desired product **LS-Ru-4-Cl** as an orange solid

(29.8 mg, 90% yield).

^{31}P NMR (202 MHz, CD_2Cl_2) δ 60.16 (d, *J* = 25.7 Hz), 27.62 (d, *J* = 25.7 Hz).

^{1}H NMR (500 MHz, CD_2Cl_2) δ 9.04 (s, 1H, aryl), 8.41 (dd, *J* = 11.8, 7.0 Hz, 1H, aryl), 8.21 (d, *J* = 8.2 Hz, 1H, aryl), 8.03 (d, *J* = 8.3 Hz, 1H, aryl), 7.89 (s, 1H, aryl), 7.86 (d, *J* = 6.9 Hz, 1H, aryl), 7.77 (s, 1H, aryl), 7.73 (s, 1H, aryl), 7.69 (t, *J* = 7.5 Hz, 1H, aryl), 7.60 (t, *J* = 7.5 Hz, 1H, aryl), 6.64 (s, 1H, aryl), 6.45 (s, 1H, aryl), 6.35 (s, 1H, aryl), 4.69 (dd, *J* = 16.5, 11.9 Hz, 1H, C***H***$_2$P), 2.93 (dd, *J* = 16.1, 12.7 Hz, 1H, C***H***$_2$P), 2.58 – 2.48 (m, 1H, PC***H***$(CH_3)_2$), 1.64 (dd, *J* = 15.9, 6.9 Hz, 3H, PCH(C***H***$_3$)$_2$), 1.35 (dd, *J* = 16.5, 6.9 Hz, 3H, PCH(C***H***$_3$)$_2$), 0.91 (dd, *J* = 12.1, 6.9 Hz, 3H, PCH(C***H***$_3$)$_2$), 0.73 – 0.65 (m, 1H, PC***H***$(CH_3)_2$), -0.23 (dd, *J* = 14.9, 6.8 Hz, 3H, PCH(C***H***$_3$)$_2$).

^{13}C NMR (126 MHz, CD_2Cl_2) δ 148.82 (d, *J* = 5.2 Hz, Ar), 148.30 (d, *J* = 6.2 Hz, Ar), 142.45 (s, Ar), 138.28 (d, *J* = 2.2 Hz, Ar), 136.01 (d, *J* = 11.4 Hz, Ar), 135.36 (s, Ar), 132.15 (d, *J* = 2.1 Hz, Ar), 129.20 (s, Ar), 128.49 (s, Ar), 127.39 (d, *J* = 23.0 Hz, Ar), 126.85 (s, Ar), 126.27 (d, *J* = 7.7 Hz, Ar), 120.64 (d, *J* = 11.1 Hz, Ar), 112.23 (d, *J* = 9.1 Hz, Ar), 111.78 (d, *J* = 5.5 Hz, Ar), 27.75 (d, *J* = 29.6 Hz, P***C***H$(CH_3)_2$), 26.15 (d, *J* = 26.0 Hz, P***C***H$(CH_3)_2$), 23.91 (d, *J* = 27.9 Hz, ***C***H$_2$P), 18.86 (s, PCH(***C***H$_3$)$_2$), 18.80 (s, PCH(***C***H$_3$)$_2$), 18.06 (d, *J* = 6.5 Hz, PCH(***C***H$_3$)$_2$), 17.97 (d, *J* = 1.9 Hz, PCH(***C***H$_3$)$_2$).

IR (KBr) = 1968 cm^{-1} (CO).

HRMS (ESI): Exact mass calculated for $C_{29}H_{28}NO_3P_2Ru^+$ ($[M\text{-}2Cl\text{-}H]^+$): 602.0582, mass found: 602.0605.

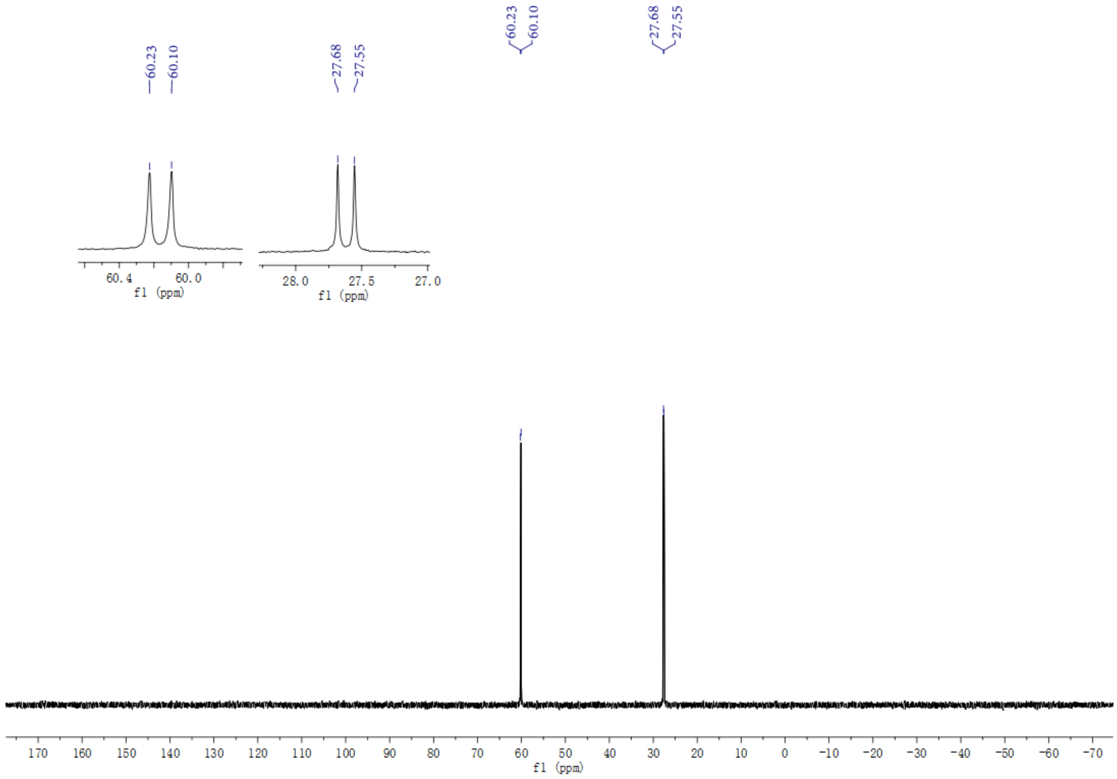

Figure S81. ^{31}P NMR (202 MHz, CD_2Cl_2) spectrum of **LS-Ru-4-Cl**

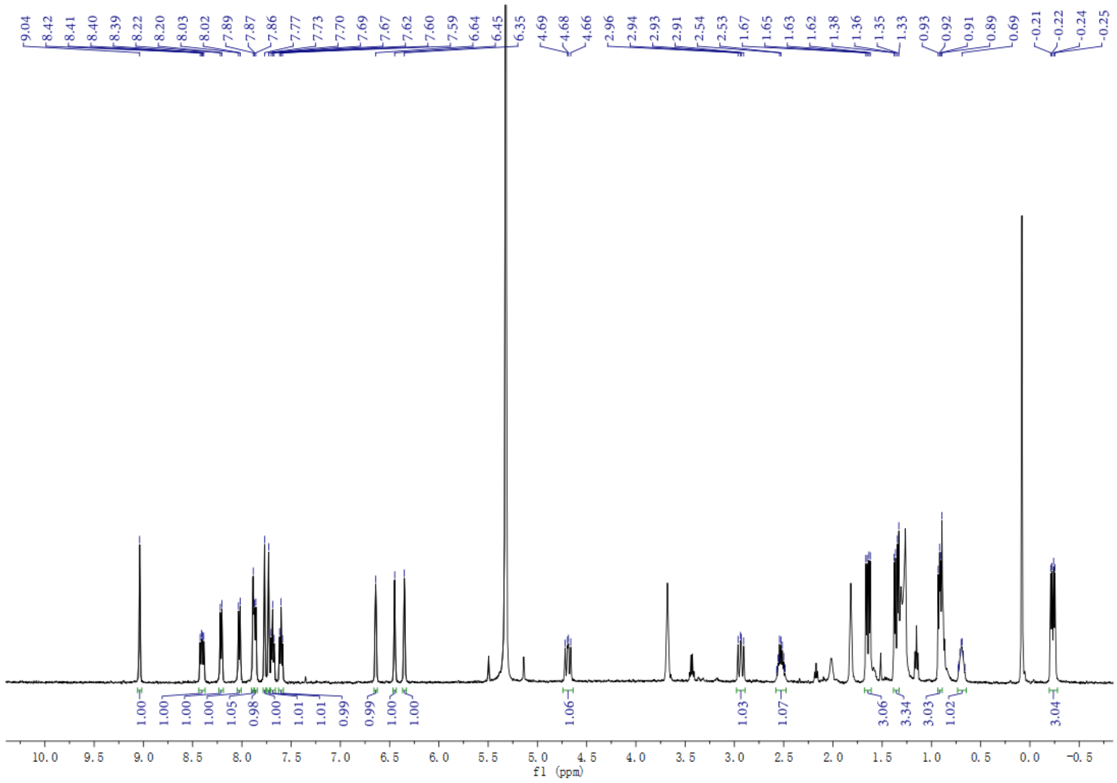

Figure S82. ^{1}H NMR (500 MHz, CD_2Cl_2) spectrum of **LS-Ru-4-Cl**

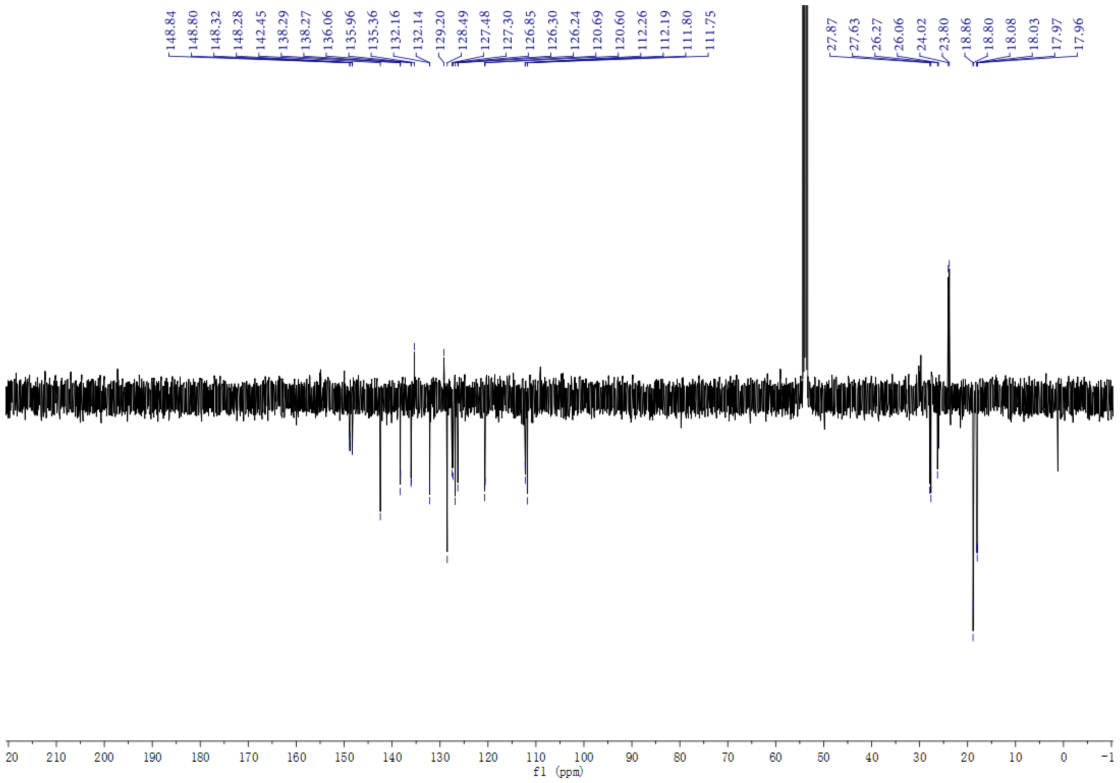

Figure S83. ^{13}C-DEPTQ NMR (126 MHz, CD_2Cl_2) spectrum of **LS-Ru-4-Cl** (CO ligand peaks not observed due to the low solubility of the complex in CD_2Cl_2)

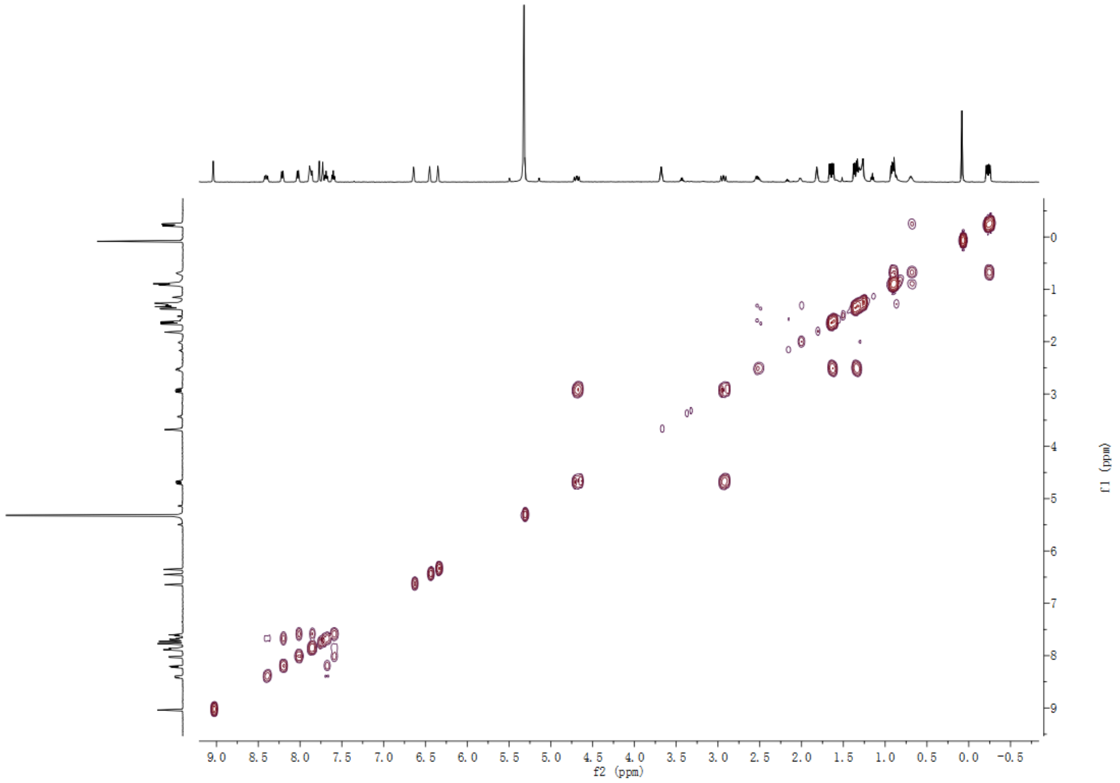

Figure S84. ^1H-^1H COSY NMR (500 MHz, CD_2Cl_2) spectrum of complex **LS-Ru-4-Cl**

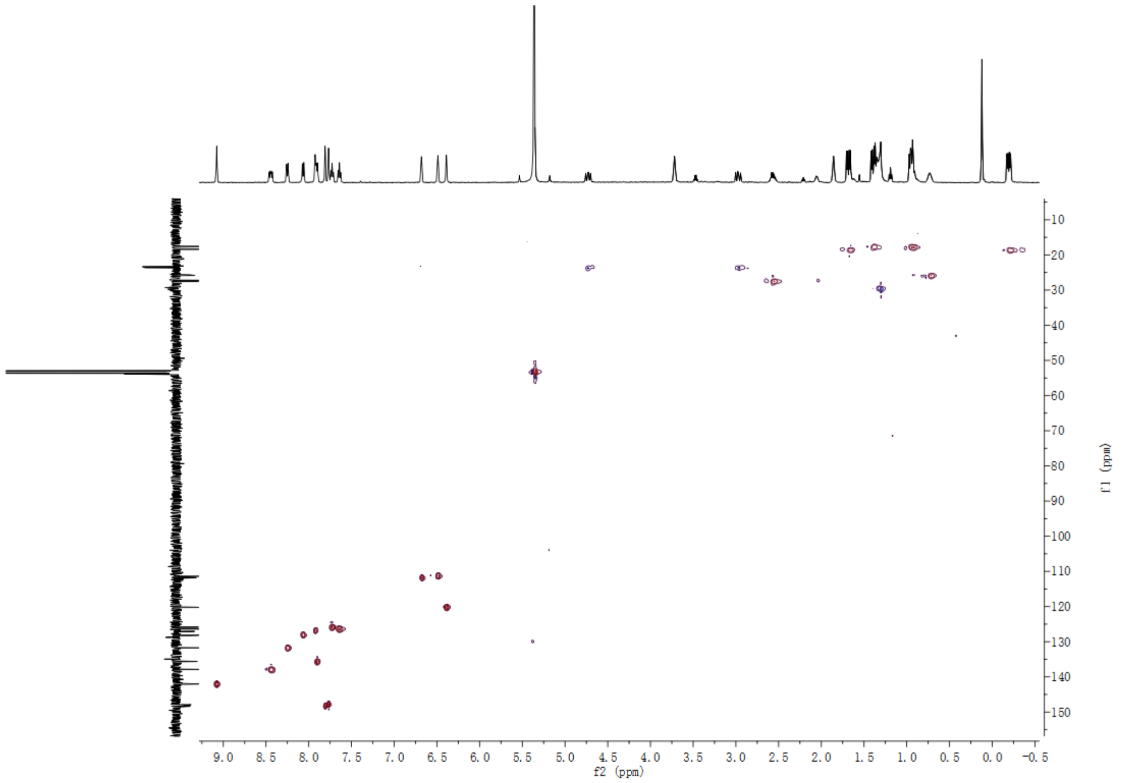

Figure S85. ^{1}H-^{13}C HSQC NMR (500 MHz, CD_2Cl_2) spectrum of complex **LS-Ru-4-Cl**

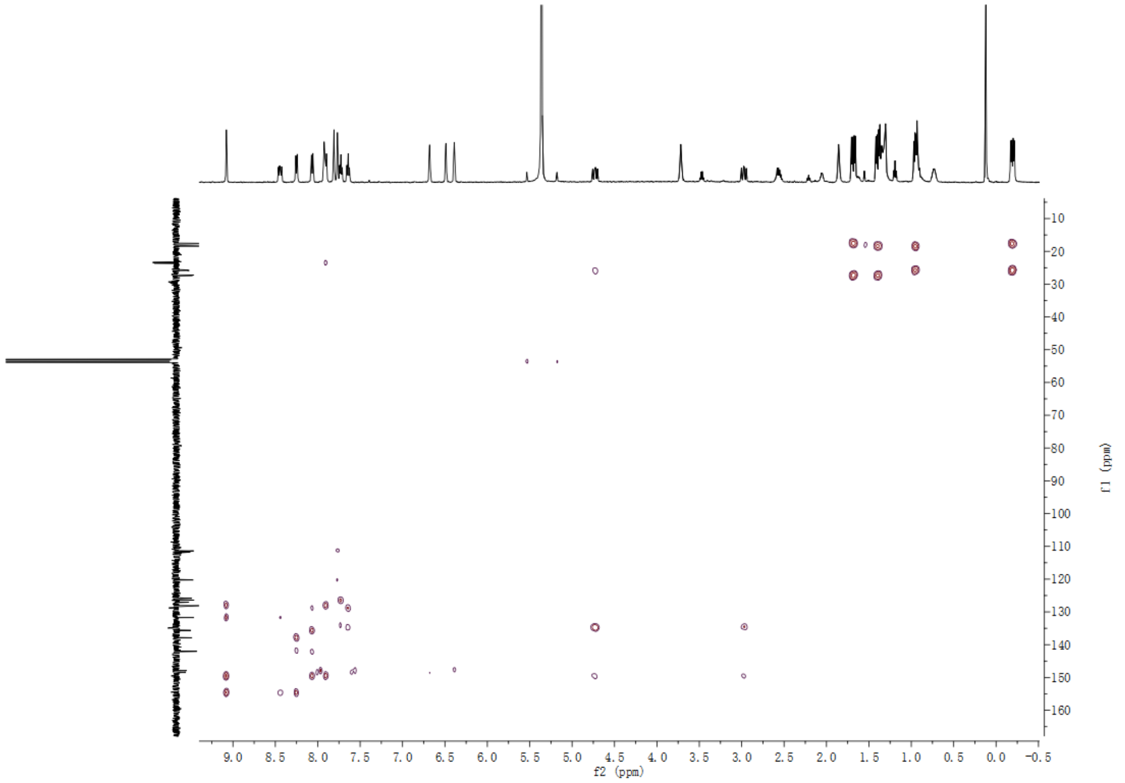

Figure S86. ^{1}H-^{13}C HMBC NMR (500 MHz, CD_2Cl_2) spectrum of complex **LS-Ru-4-Cl**

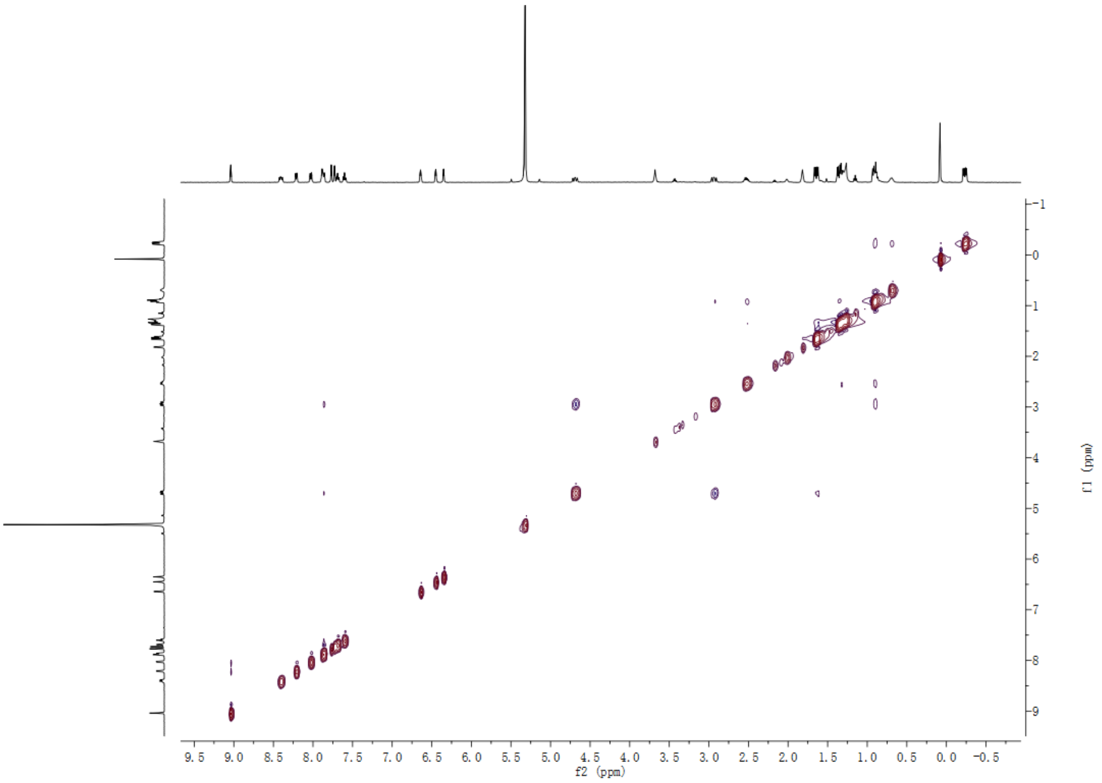

Figure S87. ^{1}H-^{1}H NOESY NMR (500 MHz, CD_2Cl_2) spectrum of **LS-Ru-4-Cl**

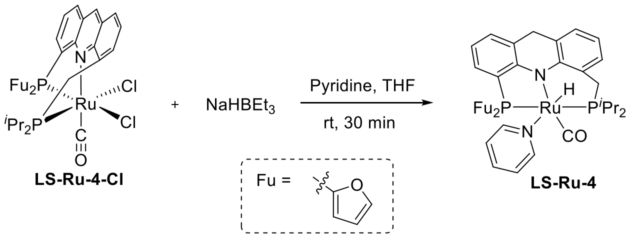

Synthesis of LS-Ru-4:

A THF solution of $NaBEt_3H$ was prepared by adding 98 μl of 1.0 M $NaBEt_3H$ (0.098 mmol) in THF to 1.0 mL of THF. The afforded solution was added to a stirring suspension of **LS-Ru-4-Cl** (32.8 mg, 0.049 mmol) and pyridine (7.8 mg, 0.098 mmol) in 4.0 mL of THF. The resulting mixture was stirred at room temperature for 30 minutes. The solvent was then removed under vacuum. The residue was redissolved in 6.0 mL diethyl ether, before passed through a 0.2 μm PTFE filter. Diethyl ether was then removed under vacuum and the residue was washed twice with 1.0 mL of n-pentane to remove a dark impurity. The remaining solid was dried under vacuum, affording **LS-Ru-4** as a yellow solid (29.3 mg; 87% yield).

^{31}P NMR (121 MHz, C_6D_6) δ 58.17 (d, J = 257.9 Hz), 21.62 (d, J = 257.8 Hz).

^{1}**H NMR** (400 MHz, C_6D_6) δ 8.48 (d, *J* = 4.8 Hz, 2H, aryl), 8.24 – 8.10 (m, 2H, aryl), 7.94 – 7.88 (m, 1H, aryl), 7.55 – 7.49 (m, 1H, aryl), 7.22 (s, 1H, aryl), 6.99 (s, 1H, aryl), 6.89 (s, 1H, aryl), 6.78 – 6.74 (m, 1H, aryl), 6.68 – 6.62 (m, 1H, aryl), 6.56 (t, *J* = 7.4 Hz, 1H, aryl), 6.37 – 6.31 (m, 1H, aryl), 6.14 – 6.10 (m, 1H, aryl), 6.09 – 6.07 (m, 1H, aryl), 6.00 (d, *J* = 3.2 Hz, 1H, aryl), 4.09 (d, *J* = 16.9 Hz, 1H, ArC$\boldsymbol{H}_2$Ar), 3.93 (d, *J* = 17.0 Hz, 1H, ArC$\boldsymbol{H}_2$Ar), 3.33 (dd, *J* = 12.9, 5.8 Hz, 1H, C$\boldsymbol{H}_2$P), 2.62 (td, *J* = 12.7, 5.1 Hz, 1H, C$\boldsymbol{H}_2$P), 2.12 – 2.05 (m, 1H, PC***H***$(CH_3)_2$), 1.87 – 1.78 (m, 1H, PC***H***$(CH_3)_2$), 1.38 (dd, *J* = 15.0, 7.0 Hz, 3H, PCH(C$\boldsymbol{H}_3)_2$), 1.29 (dd, *J* = 14.3, 6.8 Hz, 3H, PCH(C$\boldsymbol{H}_3)_2$), 0.86 (dd, *J* = 10.7, 7.1 Hz, 3H, PCH(C$\boldsymbol{H}_3)_2$), 0.58 (dd, *J* = 14.3, 7.5 Hz, 3H, PCH(C$\boldsymbol{H}_3)_2$), -13.78 (dd, *J* = 24.0, 19.4 Hz, 1H, Ru-***H***).

^{13}**C NMR** (126 MHz, C_6D_6) δ 206.97 (t, *J* = 13.8 Hz, Ru-***C***O), 164.76 (d, *J* = 32.0 Hz, Ar), 152.87 (s, Ar), 152.13 (d, *J* = 67.7 Hz, Ar), 151.30 (d, *J* = 64.0 Hz, Ar), 148.30 (d, *J* = 4.6 Hz, Ar), 147.99 (d, *J* = 3.9 Hz, Ar), 146.55 (d, *J* = 5.1 Hz, Ar), 145.43 (s, Ar), 137.64 (s, Ar), 135.15 (s, Ar), 132.01 (s, Ar), 131.05 (d, *J* = 6.3 Hz, Ar), 129.98 (s, Ar), 126.73 (d, *J* = 0.7 Hz, Ar), 124.34 (d, *J* = 25.1 Hz, Ar), 124.33 (s, Ar), 123.88 (s, Ar), 123.36 (s, Ar), 122.02 (d, *J* = 14.9 Hz, Ar), 118.41 (s, Ar), 118.14 (s, Ar), 118.09 (s, Ar), 111.00 (d, *J* = 8.9 Hz, Ar), 110.32 (d, *J* = 5.0 Hz, Ar), 110.00 (s, Ar), 35.97 (s, Ar***C***H_2Ar), 33.32 (d, *J* = 23.8 Hz, ***C***H_2P), 26.32 (d, *J* = 25.9 Hz, P***C***$H(CH_3)_2$), 23.79 (dd, *J* = 11.9, 4.7 Hz, P***C***$H(CH_3)_2$), 19.93 (s, PCH(***C***$H_3)_2$), 19.48 (d, *J* = 2.3 Hz, PCH(***C***$H_3)_2$), 18.86 (d, *J* = 5.4 Hz, PCH(***C***$H_3)_2$), 16.72 (d, *J* = 2.6 Hz, 2.1 Hz, PCH(***C***$H_3)_2$).

IR (KBr) = 1911 cm^{-1} (CO).

HRMS (ESI): Exact mass calculated for $C_{29}H_{28}NO_3P_2Ru^+$ ([M-Pyridine-3H]$^+$): 602.0582, mass found: 602.0598.

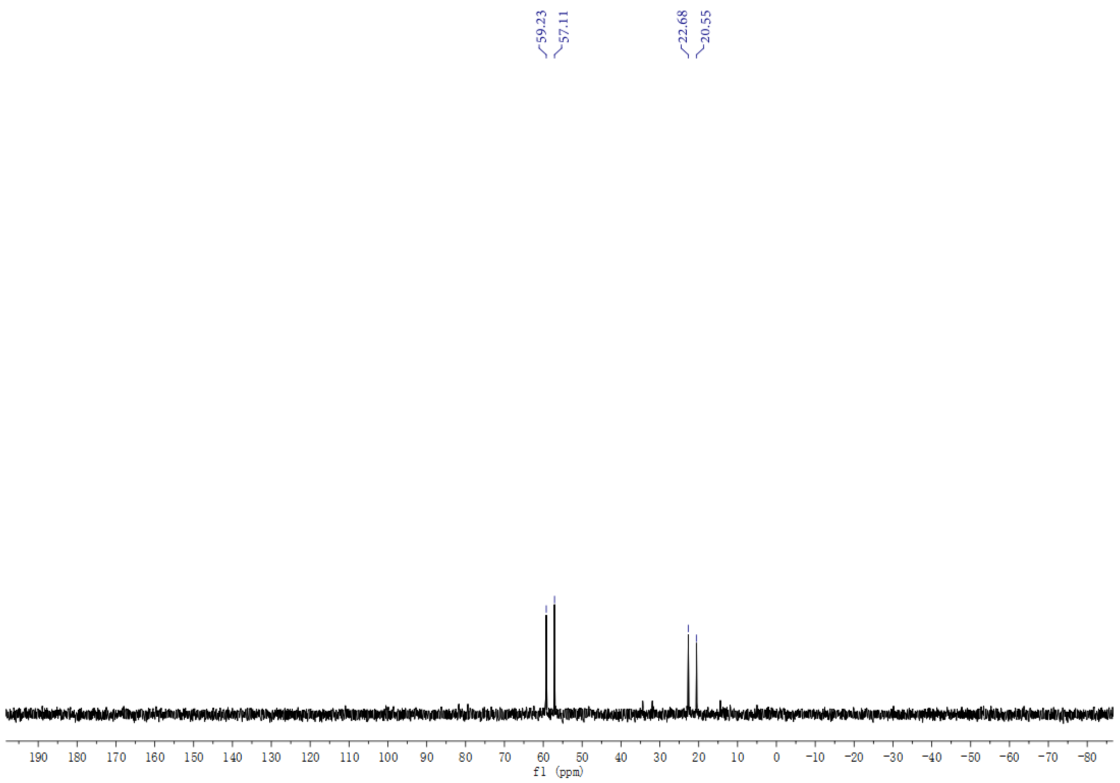

Figure S88. ^{31}P NMR (121 MHz, C_6D_6) spectrum of **LS-Ru-4**

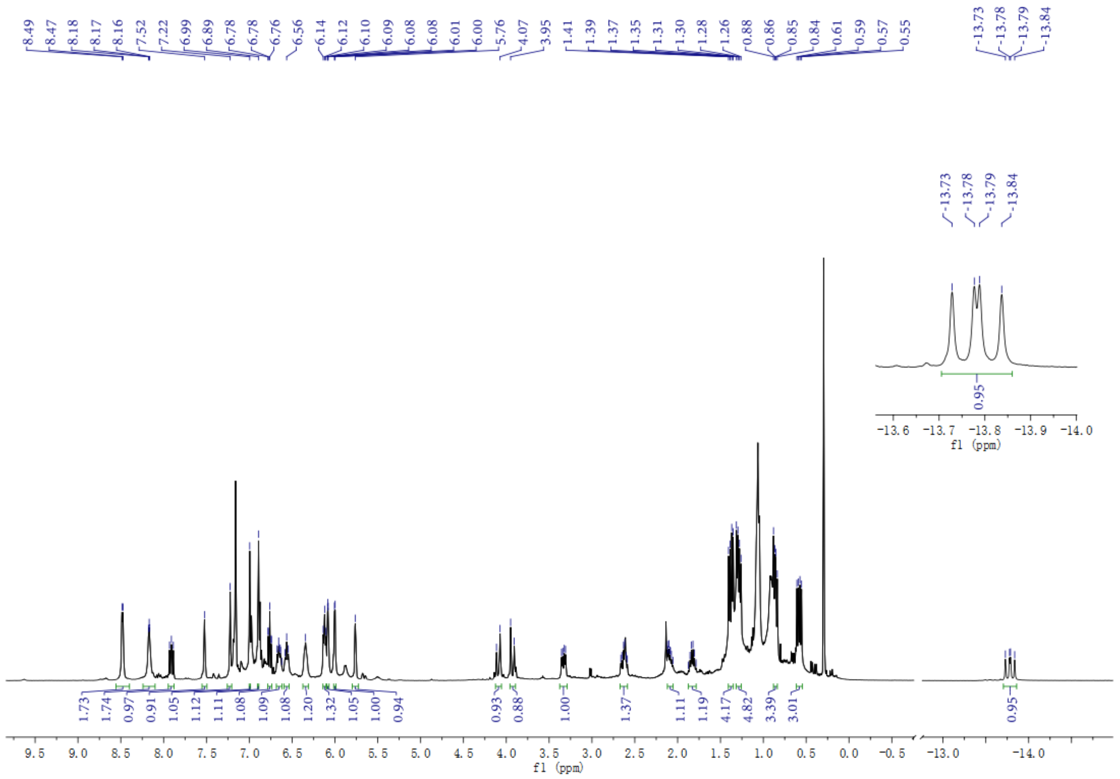

Figure S89. ^{1}H NMR (400 MHz, C_6D_6) spectrum of **LS-Ru-4**

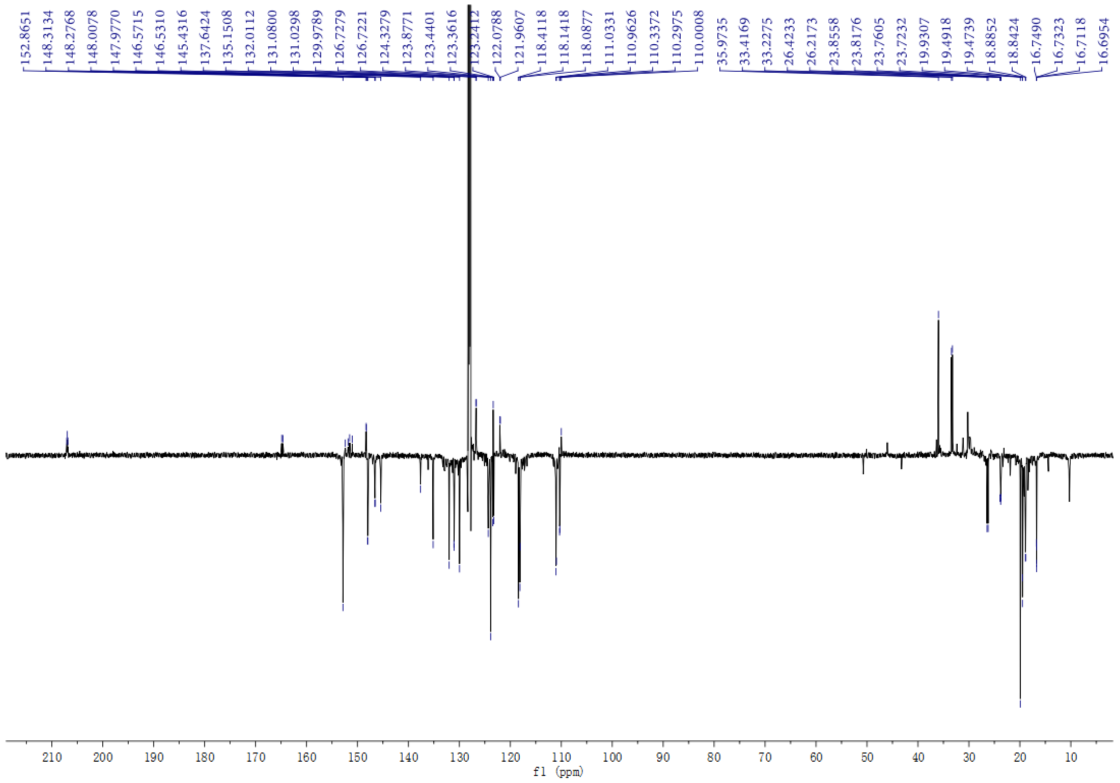

Figure S90. ^{13}C-DEPTQ NMR (126 MHz, C_6D_6) spectrum of **LS-Ru-4**

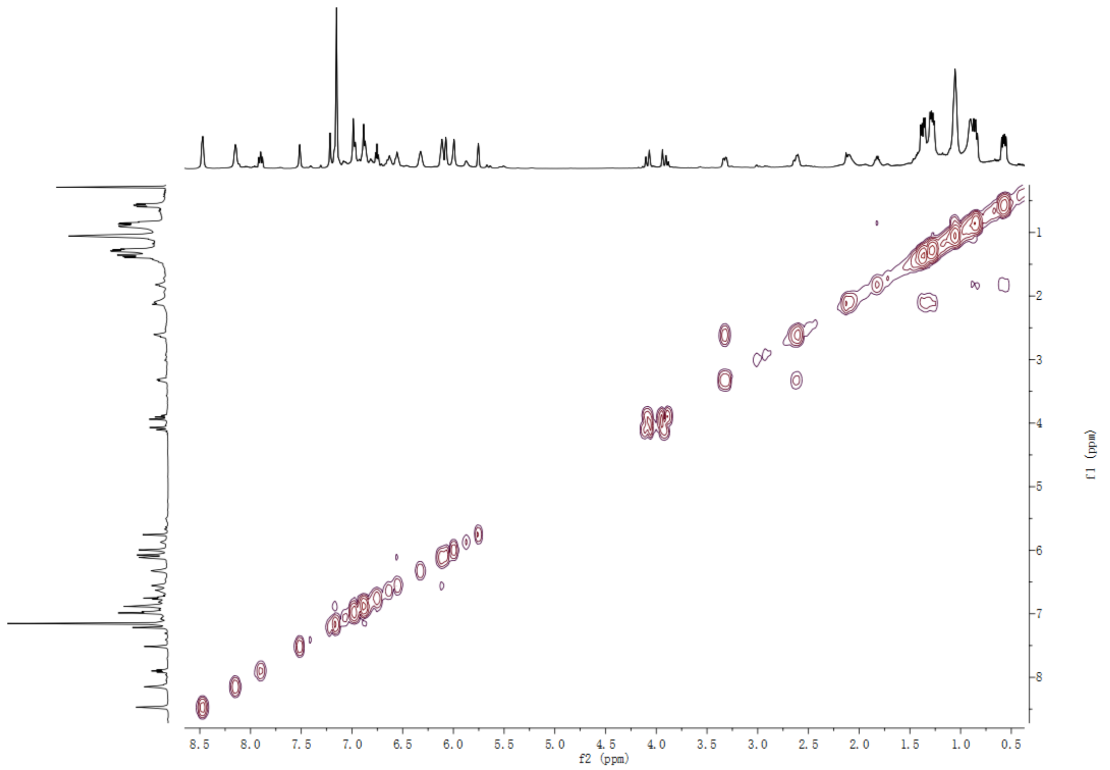

Figure S91. ^{1}H-^{1}H COSY NMR (500 MHz, C_6D_6) spectrum of **LS-Ru-4**

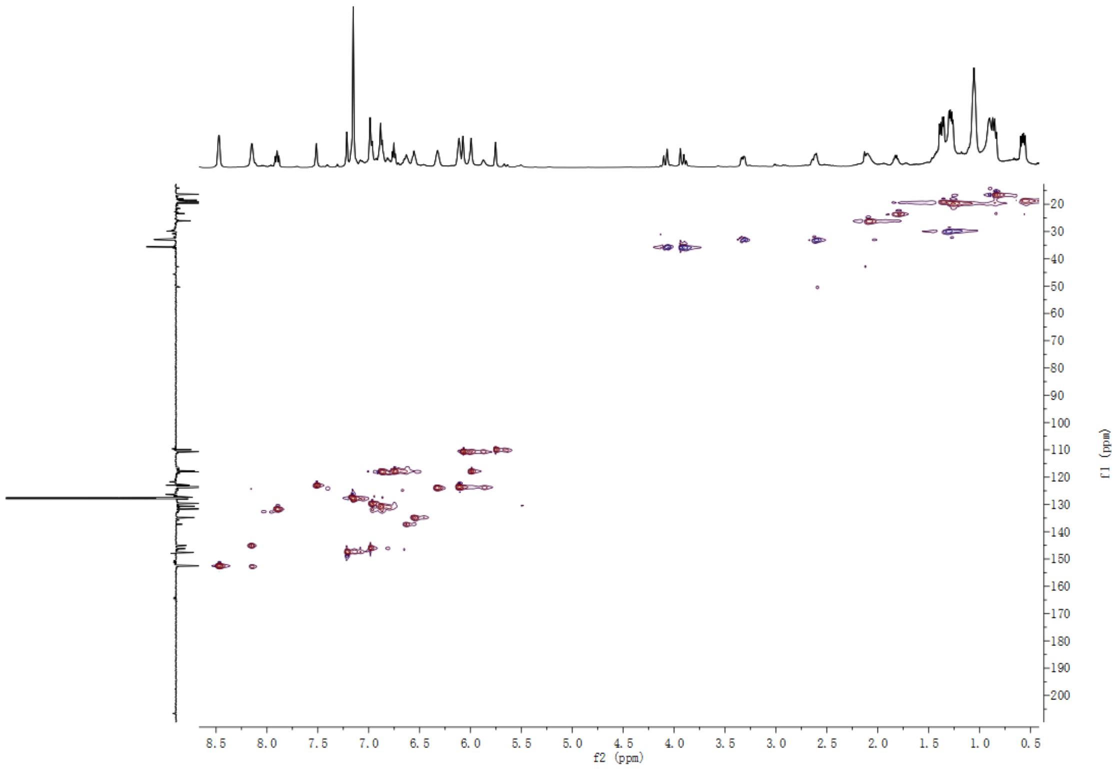

Figure S92. ^{1}H-^{13}C HSQC NMR (500 MHz, C_6D_6) spectrum of complex **LS-Ru-4**

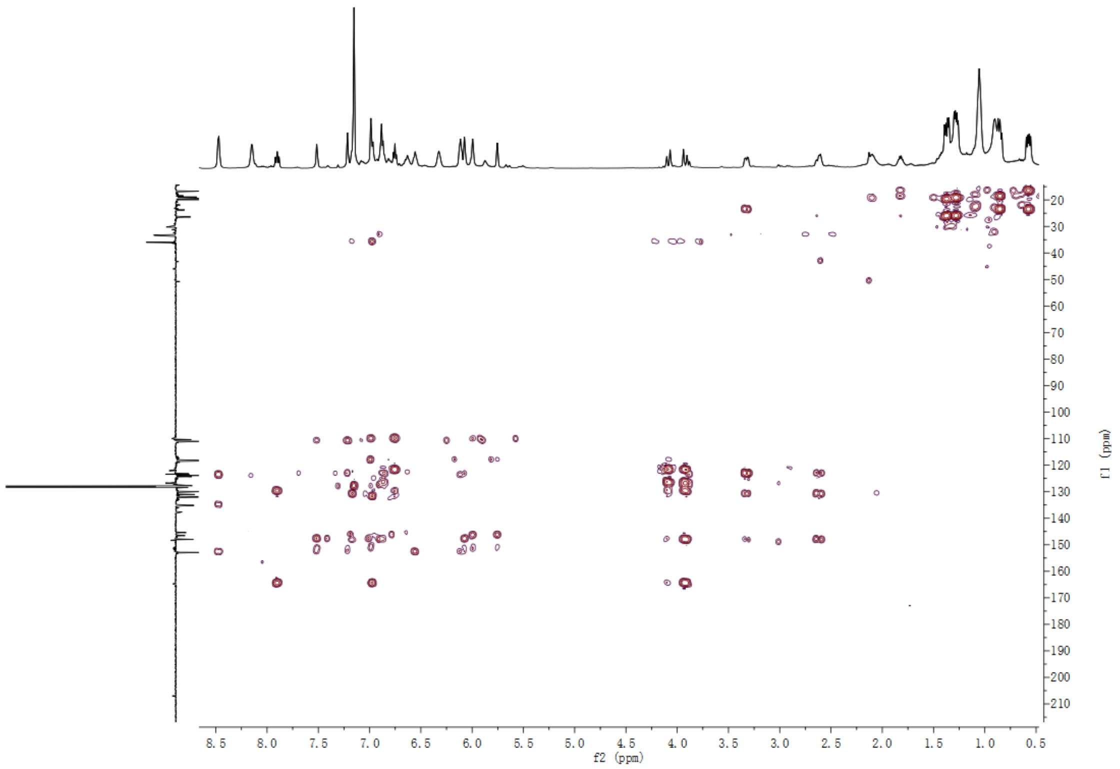

Figure S93. ^{1}H-^{13}C HMBC NMR (500 MHz, C_6D_6) spectrum of complex **LS-Ru-4**

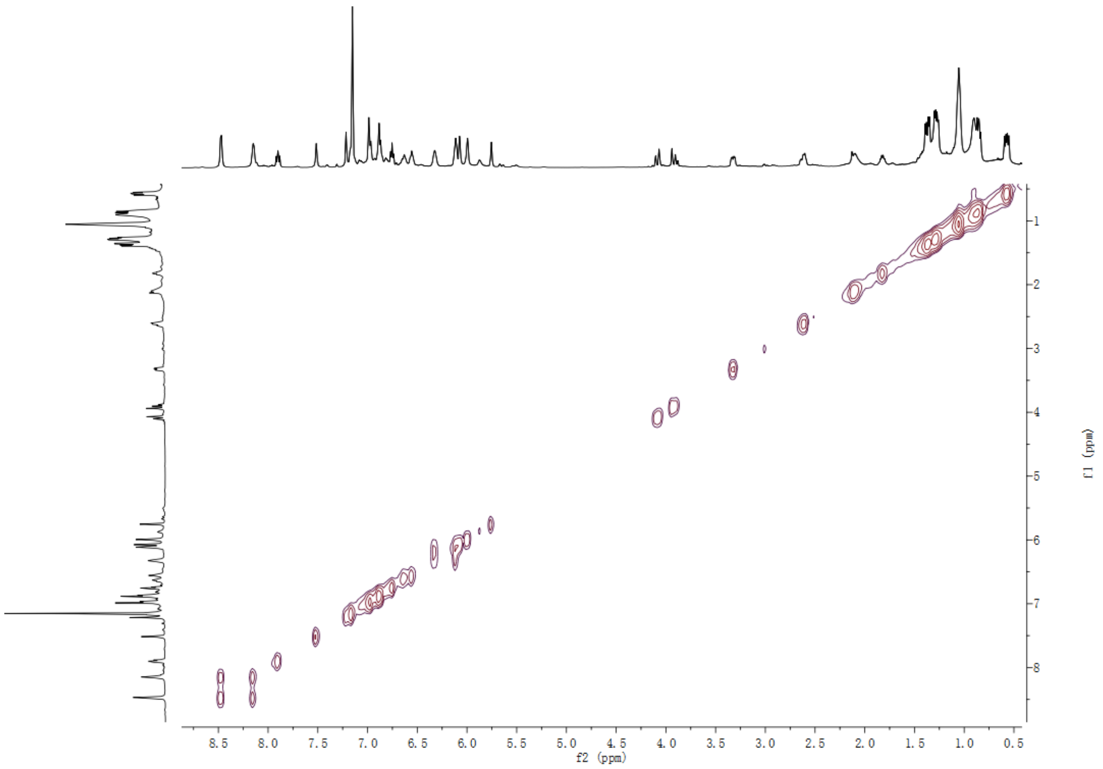

Figure S94. ^{1}H-^{1}H NOESY NMR (500 MHz, C_6D_6) spectrum of **LS-Ru-4**

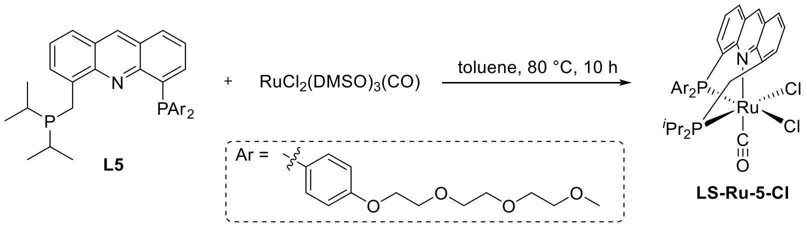

Synthesis of LS-Ru-5-Cl:

In a glovebox, **L5** (130.9 mg, 0.160 mmol) and $RuCl_2(CO)(DMSO)_3$ (63.0 mg, 0.145 mmol) were suspended in toluene (10 mL) in an oven-dried 100 mL Schlenk flask equipped with a magnetic stirring bar. The flask was sealed and taken out of the glovebox, and stirred at 80 ˚C for 10 hours. After cooling the reaction mixture to room temperature, the flask was taken into the glovebox again and the solvent was removed under vacuum. The residue was dissolved in a small amount of toluene, and ether was added to precipitate an orange solid. The resulting solid was then washed with ether for several times and dried under vacuum to give the desired product **LS-Ru-5-Cl** as an orange solid (140.1 mg, 95% yield).

^{31}P NMR (202 MHz, C_6D_6) δ 56.09 (d, J = 22.6 Hz), 54.80 (d, J = 23.1 Hz).

^{1}H NMR (400 MHz, C_6D_6) δ 9.19 (s, 1H, aryl), 8.52 (t, J = 9.5 Hz, 2H, aryl), 8.43 (d, J = 6.5 Hz, 1H, aryl), 7.85 – 7.77 (m, 2H, aryl), 7.36 (d, J = 4.7 Hz, 1H, aryl), 7.05 – 6.95 (m, 2H, aryl), 6.91 – 6.79 (m, 4H, aryl), 6.59 (d, J = 6.4 Hz, 2H, aryl), 4.95 – 4.81 (m, 1H, C$\boldsymbol{H}_2$P), 3.83 – 3.76 (m, 4H, OC$\boldsymbol{H}_2$), 3.52 – 3.46 (m, 10H, OC$\boldsymbol{H}_2$), 3.41 – 3.36 (m, 8H, OC$\boldsymbol{H}_2$), 3.30 – 3.27 (m, 2H, OC$\boldsymbol{H}_2$), 3.16 (s, 3H, OC$\boldsymbol{H}_3$), 3.08 (s, 3H, OC$\boldsymbol{H}_3$), 2.61 – 2.50 (m, 1H, C$\boldsymbol{H}_2$P), 2.28 – 2.17 (m, 1H, PC$\boldsymbol{H}$(CH_3)$_2$), 1.84 (dd, J = 15.1, 6.8 Hz, 3H, PCH(C$\boldsymbol{H}_3$)$_2$), 1.23 (dd, J = 16.1, 6.8 Hz, 3H, PCH(C$\boldsymbol{H}_3$)$_2$), 0.57 – 0.44 (m, 4H, PC$\boldsymbol{H}$(CH_3)$_2$, PCH(C$\boldsymbol{H}_3$)$_2$), -0.34 – -0.48 (m, 3H, PCH(C$\boldsymbol{H}_3$)$_2$).

^{13}C NMR (126 MHz, C_6D_6) δ 205.29 (t, J = 13.0 Hz, Ru-***C***O), 161.64 (s, Ar), 160.69 (s, Ar), 155.56 (d, J = 17.1 Hz, Ar), 150.16 (s, Ar), 144.71 (s, Ar), 139.78 (s, Ar), 139.69 (s, Ar), 136.29 (s, Ar), 135.77 (s, Ar), 135.41 (d, J = 12.1 Hz, Ar), 133.88 (s, Ar), 133.13 (s, Ar), 131.08 (s, Ar), 130.20 (d, J = 50.6 Hz, Ar), 129.63 (s, Ar), 127.31 (d, J = 8.0 Hz, Ar), 125.60 (s, Ar), 124.51 (s, Ar), 124.47 (s, Ar), 122.08 (d, J = 63.3 Hz, Ar), 114.60 (s, Ar), 114.51 (s, Ar), 72.43 (s, O$\boldsymbol{C}H_2$), 72.34 (s, O$\boldsymbol{C}H_2$), 71.11 (s, O$\boldsymbol{C}H_2$), 71.07 (s, O$\boldsymbol{C}H_2$), 70.99 (s, O$\boldsymbol{C}H_2$), 70.86 (s, O$\boldsymbol{C}H_2$), 69.71 (s, O$\boldsymbol{C}H_2$), 69.65 (s, O$\boldsymbol{C}H_2$), 68.04 (s, O$\boldsymbol{C}H_2$), 67.82 (s, O$\boldsymbol{C}H_2$), 58.77 (s, O$\boldsymbol{C}H_3$), 58.68 (s, O$\boldsymbol{C}H_3$), 27.72 (d, J = 29.2 Hz, P$\boldsymbol{C}$H(CH_3)$_2$), 24.77 (d, J = 28.6 Hz, $\boldsymbol{C}H_2$P), 24.66 (d, J = 18.3 Hz, P$\boldsymbol{C}$H(CH_3)$_2$), 19.52 (s, PCH($\boldsymbol{C}H_3$)$_2$), 18.98 (s, PCH($\boldsymbol{C}H_3$)$_2$), 18.38 (s, PCH($\boldsymbol{C}H_3$)$_2$), 17.71 (d, J = 5.6 Hz, PCH($\boldsymbol{C}H_3$)$_2$).

IR (KBr) = 1951 cm^{-1} (CO).

HRMS (ESI): Exact mass calculated for $C_{47}H_{60}NO_9P_2Ru^+$ ([M-2Cl-H]$^+$): 946.2781, mass found: 946.2805.

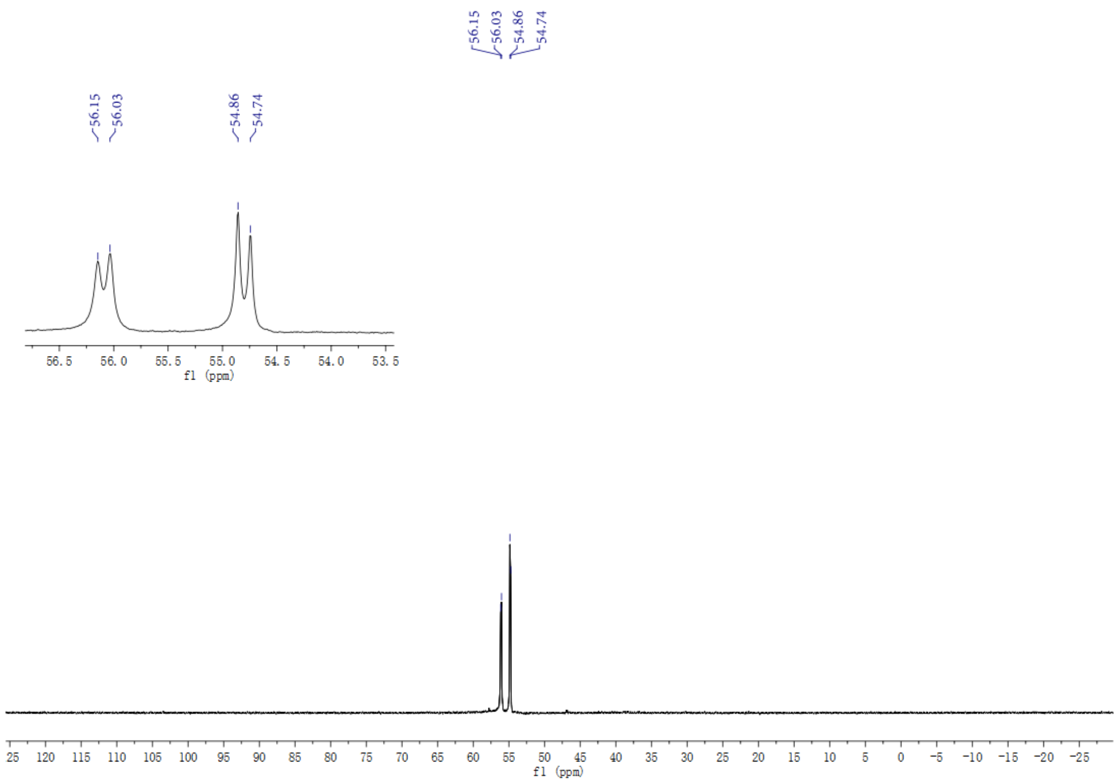

Figure S95. ^{31}P NMR (202 MHz, C_6D_6) spectrum of **LS-Ru-5-Cl**

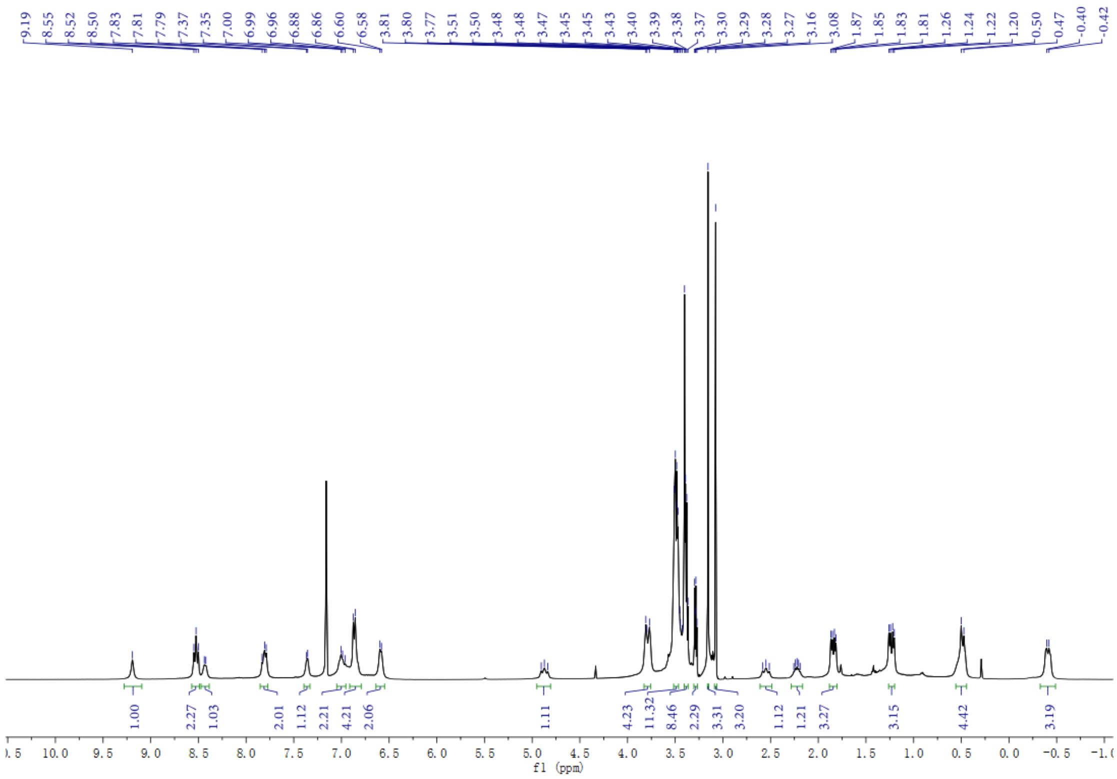

Figure S96. ^{1}H NMR (400 MHz, C_6D_6) spectrum of **LS-Ru-5-Cl**

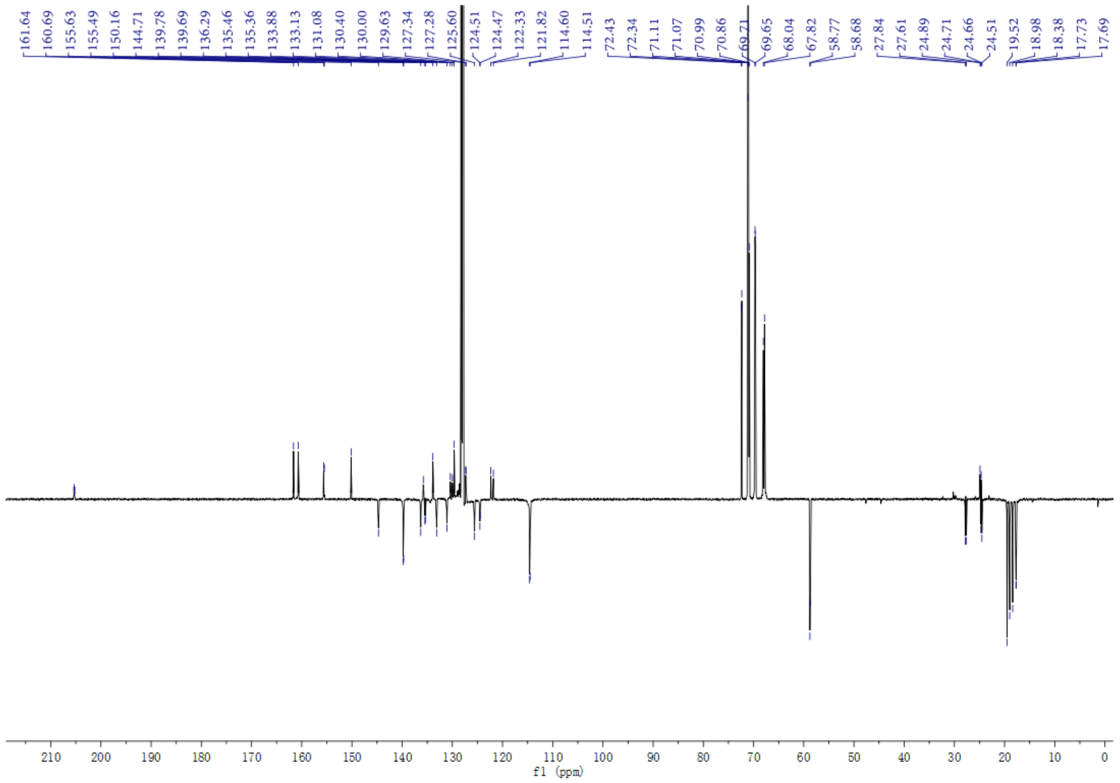

Figure S97. ^{13}C-DEPTQ NMR (126 MHz, C_6D_6) spectrum of **LS-Ru-5-Cl**

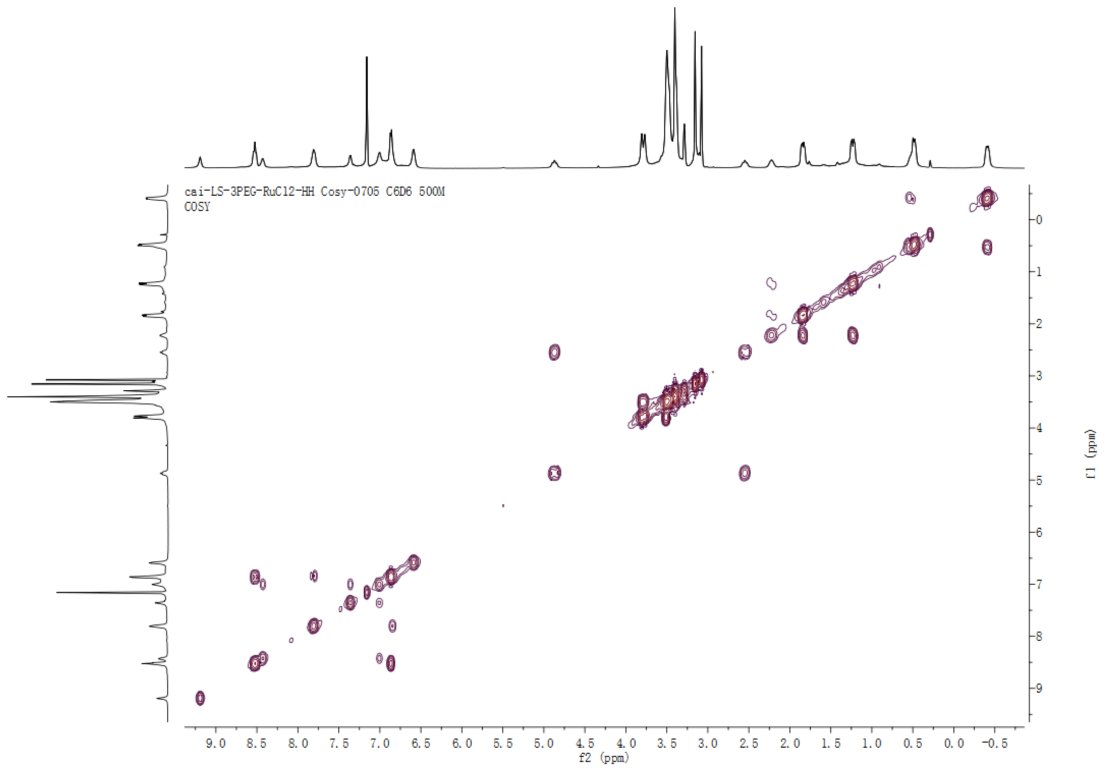

Figure S98. ^{1}H-^{1}H COSY NMR (500 MHz, C_6D_6) spectrum of **LS-Ru-5-Cl**

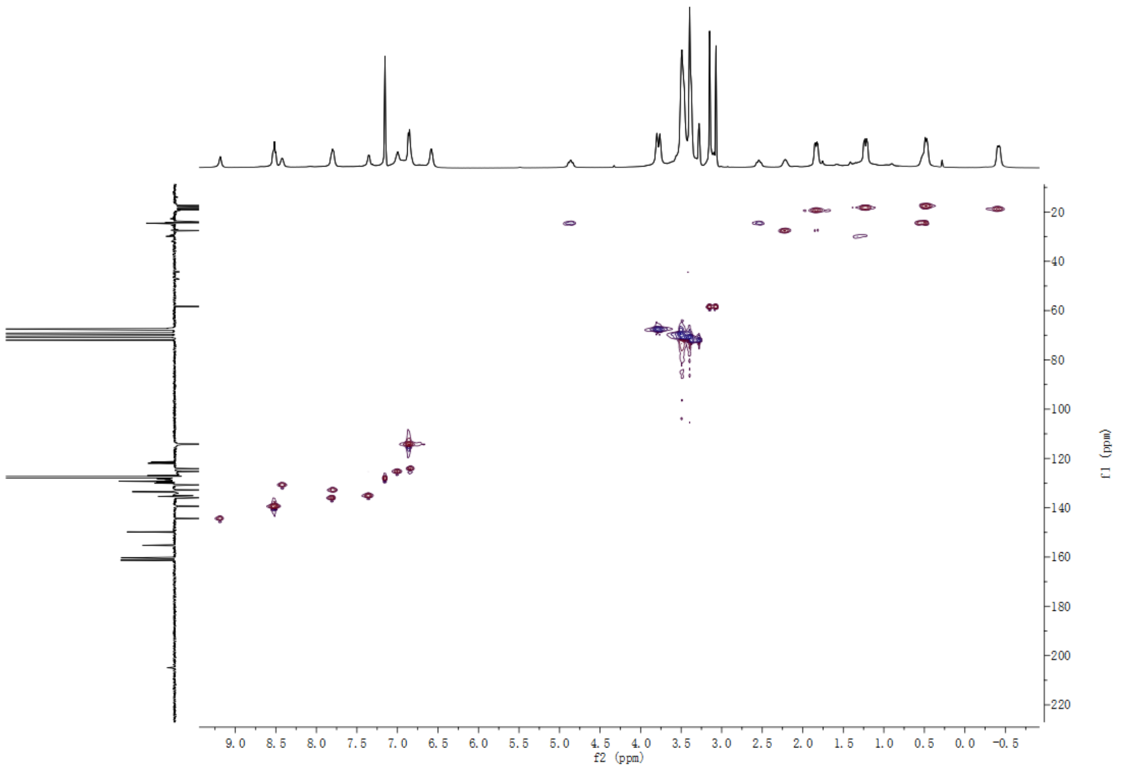

Figure S99. ^{1}H-^{13}C HSQC NMR (500 MHz, C_6D_6) spectrum of complex **LS-Ru-5-Cl**

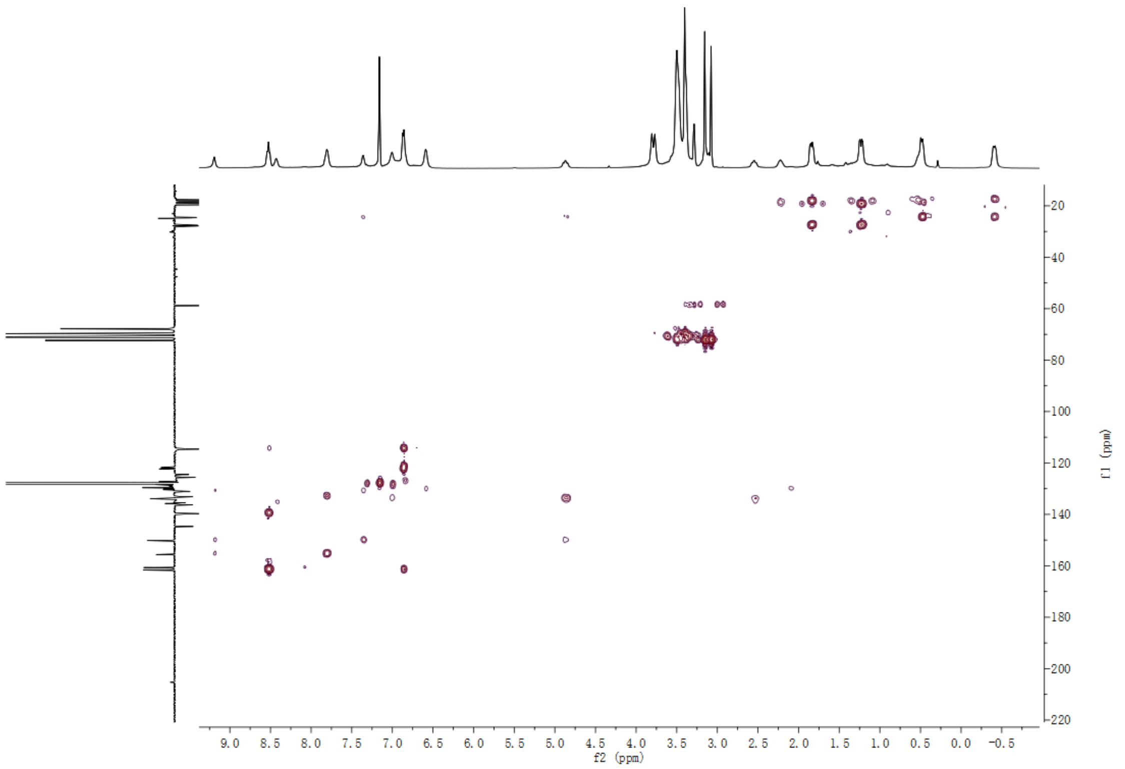

Figure S100. ^{1}H-^{13}C HMBC NMR (500 MHz, C_6D_6) spectrum of complex **LS-Ru-5-Cl**

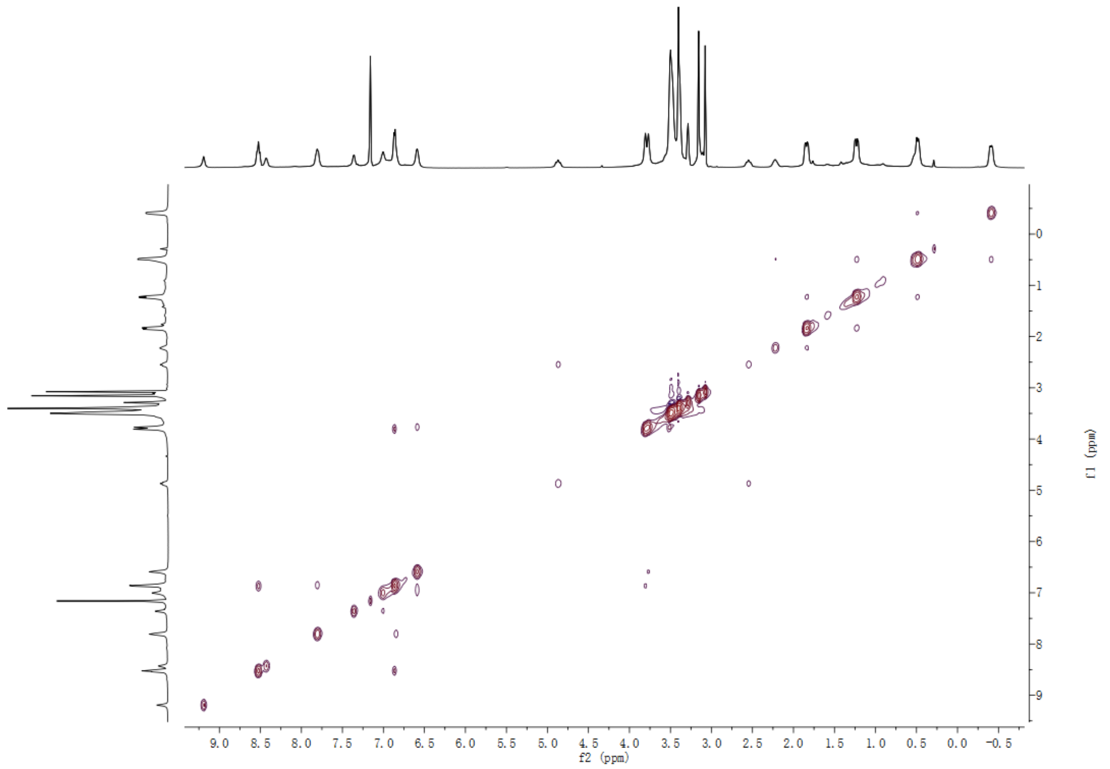

Figure S101. ^{1}H-^{1}H NOESY NMR (500 MHz, C_6D_6) spectrum of **LS-Ru-5-Cl**

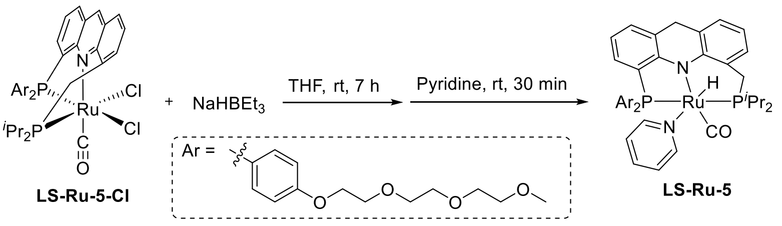

Synthesis of LS-Ru-5:

A THF solution of $NaBEt_3H$ was prepared by adding 258 µl of 1.0 M $NaBEt_3H$ (0.258 mmol) in THF to 2.5 mL of THF. The afforded solution was added dropwise to a stirring solution of **LS-Ru-5-Cl** (131.2 mg, 0.129 mmol) in 7.5 mL of THF. The resulting mixture was stirred at room temperature for 7 hours, after which pyridine (20.4 mg, 0.258 mmol) was added, and the mixture was stirred for an additional 30 minutes at room temperature. The solvent was then removed under vacuum. The residue was redissolved in 10 mL toluene, before passed through a 0.2 µm PTFE filter. Toluene was then removed under vacuum and the residue was washed twice with 4.0 mL of n-pentane to remove a dark impurity. The remaining product was dried under vacuum, affording **LS-Ru-5** as a brown semi-solid (125.0 mg; 94% yield).

^{31}P NMR (121 MHz, C_6D_6) δ 58.36 (d, J = 248.1 Hz), 53.46 (d, J = 248.1 Hz).

^{1}H NMR (400 MHz, C_6D_6) δ 8.37 – 8.31 (m, 2H, aryl), 8.16 (d, J = 5.2 Hz, 2H, aryl), 7.28 (t, J = 8.4 Hz, 1H, aryl), 7.20 (d, J = 6.9 Hz, 1H, aryl), 7.02 (d, J = 6.7 Hz, 1H, aryl), 6.95 (d, J = 8.5 Hz, 1H, aryl), 6.89 (t, J = 7.3 Hz, 2H, aryl), 6.76 (t, J = 7.2 Hz, 1H, aryl), 6.67 (t, J = 7.4 Hz, 2H, aryl), 6.62 – 6.55 (m, 1H, aryl), 6.42 – 6.32 (m, 4H, aryl), 6.12 – 6.06 (m, 1H, aryl), 4.21 (d, J = 16.8 Hz, 1H, ArC***H***$_2$Ar), 4.00 (d, J = 17.0 Hz, 1H, ArC***H***$_2$Ar), 3.79 – 3.73 (m, 2H, OC***H***$_2$), 3.73 – 3.64 (m, 2H, OC***H***$_2$), 3.54 – 3.38 (m, 17H, C***H***$_2$P, OC***H***$_2$), 3.36 – 3.30 (m, 4H, OC***H***$_2$), 3.15 – 3.09 (m, 6H, OC***H***$_3$), 2.73 (td, J = 12.3, 3.6 Hz, 1H, C***H***$_2$P), 2.23 – 2.11 (m, 1H, PC***H***(CH_3)$_2$), 1.83 (td, J = 13.9, 7.0 Hz, 1H, PC***H***(CH_3)$_2$), 1.45 – 1.34 (m, 6H PCH(C***H***$_3$)$_2$), 0.89 – 0.86 (m, 3H PCH(C***H***$_3$)$_2$), 0.54 (dd, J = 13.8, 7.4 Hz, 3H), -13.46 (t, J = 19.8 Hz, 1H, Ru-***H***).

^{13}C NMR (126 MHz, C_6D_6) δ 207.80 (t, J = 13.1 Hz, Ru-***C***O), 164.41 (dd, J = 27.1, 2.6 Hz, Ar), 161.03 (d, J = 1.8 Hz, Ar), 159.32 (s, Ar), 152.52 (s, Ar), 148.48 (d, J = 4.5 Hz, Ar), 145.34 (s, Ar), 137.77 (s, Ar), 136.61 (d, J = 13.6 Hz, Ar), 134.79 (s, Ar), 133.01 (d, J = 11.1 Hz, Ar), 131.18 (d, J = 6.1 Hz, Ar), 130.91 (s, Ar), 129.36 (s, Ar), 128.76 (d, J = 44.7 Hz, Ar), 127.76 (s, Ar), 127.34 (d, J = 54.3 Hz, Ar), 126.48 (s, Ar), 124.40 (s, Ar), 123.83 (s, Ar), 123.52 (s, Ar), 121.85 (d, J = 13.2 Hz, Ar), 118.29 (s, Ar), 118.17 (d, J = 6.6 Hz, Ar), 114.74 (d, J = 11.1 Hz, Ar), 114.33 (s, Ar), 114.26 (s, Ar), 113.88 (s, Ar), 72.39 (s, O***C***H$_2$), 72.38 (s, O***C***H$_2$), 71.16 (s, O***C***H$_2$), 71.15 (s, O***C***H$_2$), 71.07 (s, O***C***H$_2$), 71.06 (s, O***C***H$_2$), 70.94 (s, O***C***H$_2$), 70.91 (s, O***C***H$_2$), 69.78 (s, O***C***H$_2$), 69.77 (s, O***C***H$_2$), 67.69 (s, O***C***H$_2$), 67.61 (s, O***C***H$_2$), 58.71 (s, O***C***H$_3$), 58.70 (s, O***C***H$_3$), 36.24 (s, Ar***C***H$_2$Ar), 33.78 (d, J = 22.7 Hz, ***C***H$_2$P), 26.44 (d, J = 24.8 Hz, P***C***H(CH_3)$_2$), 24.00 (dd, J = 10.9, 4.6 Hz, P***C***H(CH_3)$_2$), 20.02 (s, PCH(***C***H$_3$)$_2$), 19.64 (d, J = 2.3 Hz, PCH(***C***H$_3$)$_2$), 18.90 (d, J = 5.6 Hz, PCH(***C***H$_3$)$_2$), 16.87 (d, J = 2.8 Hz, PCH(***C***H$_3$)$_2$).

IR (KBr) = 1900 cm^{-1} (CO).

HRMS (ESI): Exact mass calculated for $C_{47}H_{60}NO_9P_2Ru^+$ ([M-Pyridine-3H]$^+$): 946.2781, mass found: 946.2803.

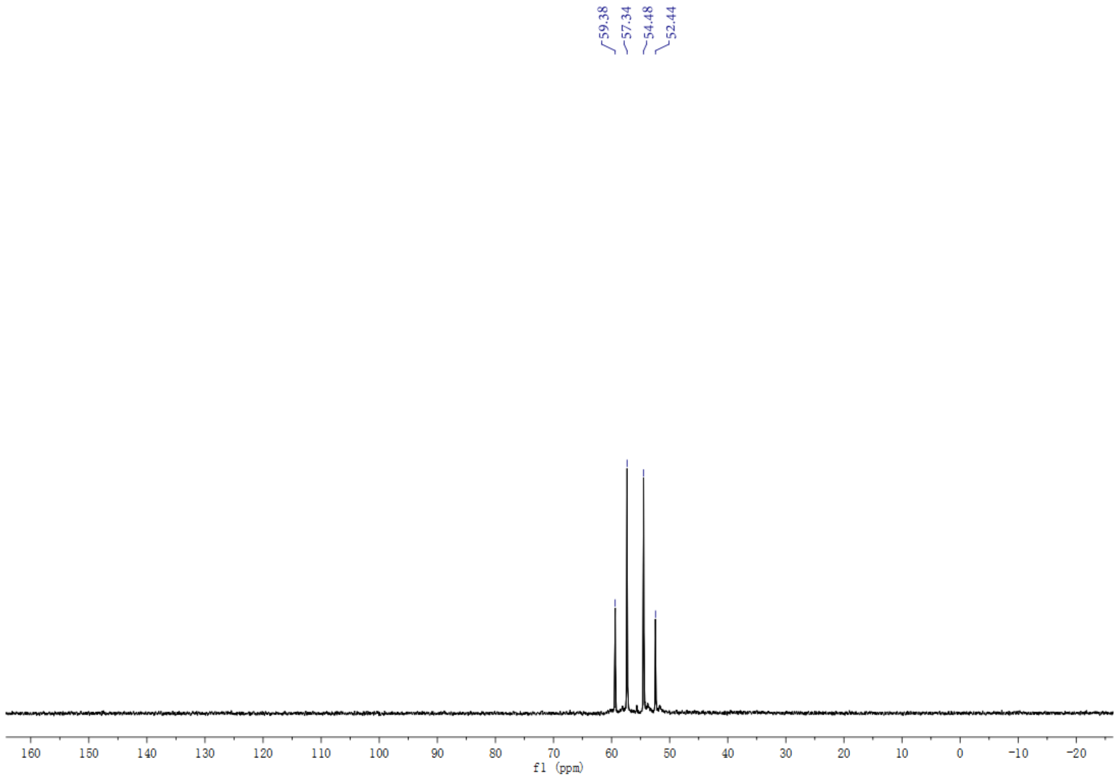

Figure S102. ^{31}P NMR (121 MHz, C_6D_6) spectrum of **LS-Ru-5**

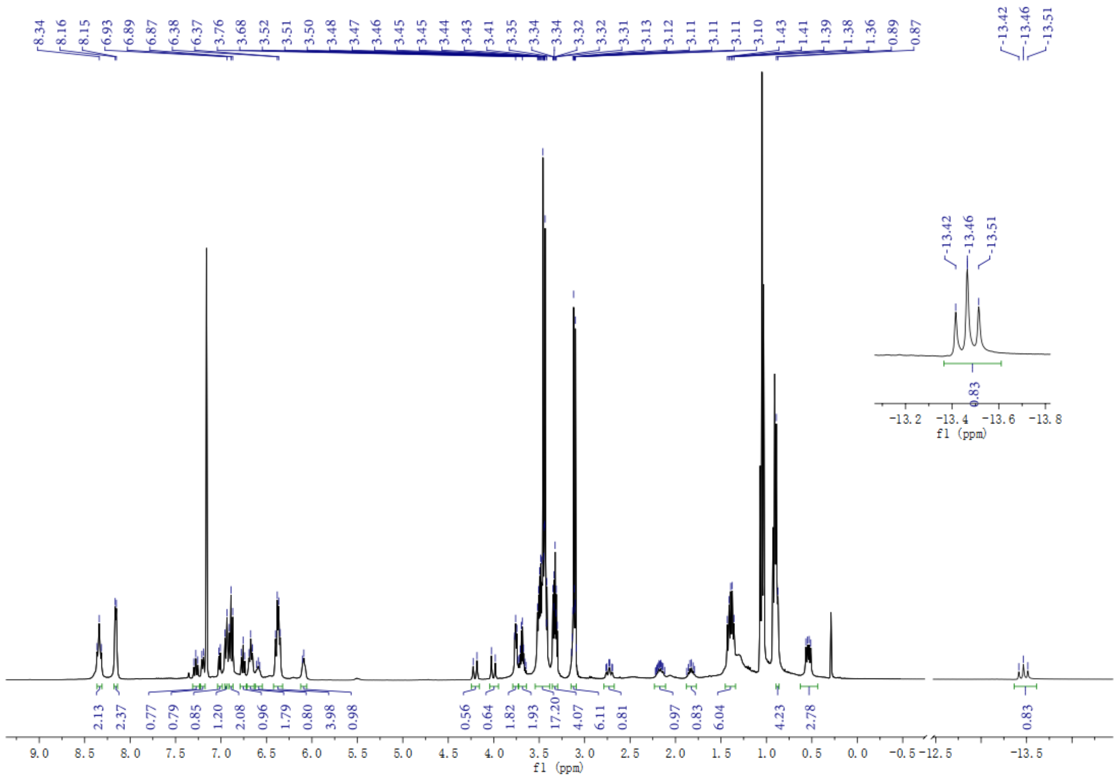

Figure S103. ^{1}H NMR (400 MHz, C_6D_6) spectrum of **LS-Ru-5**

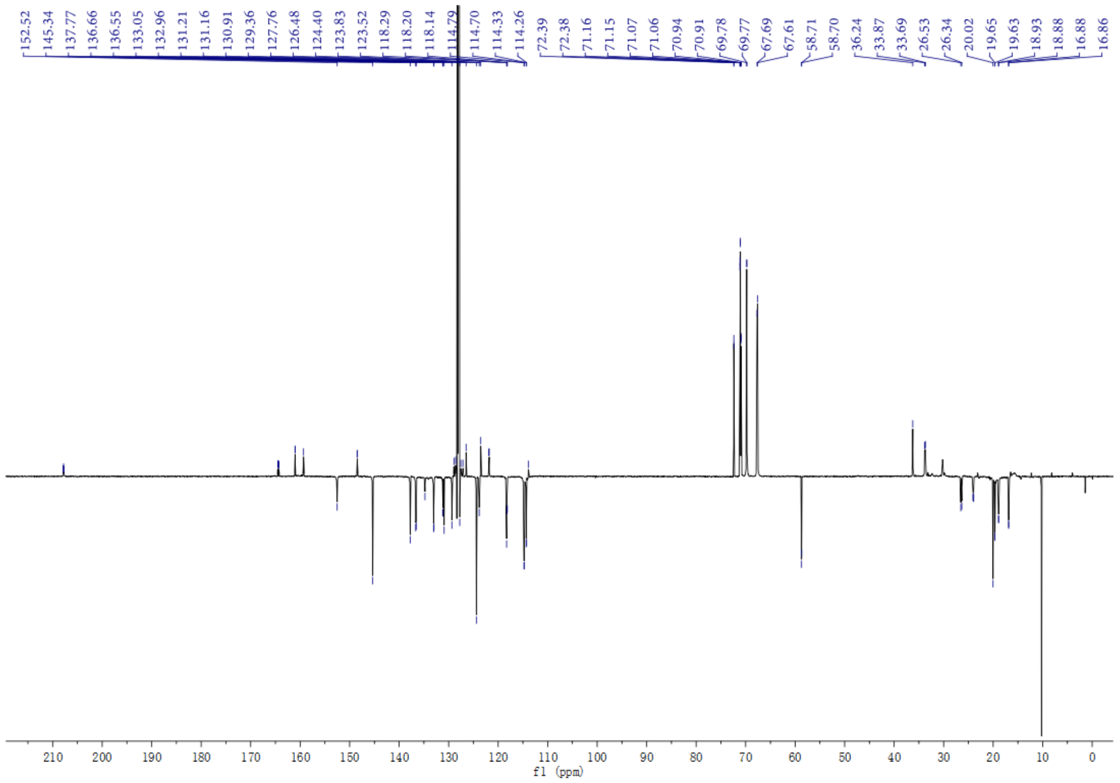

Figure S104. ^{13}C-DEPTQ NMR (126 MHz, C_6D_6) spectrum of **LS-Ru-5**

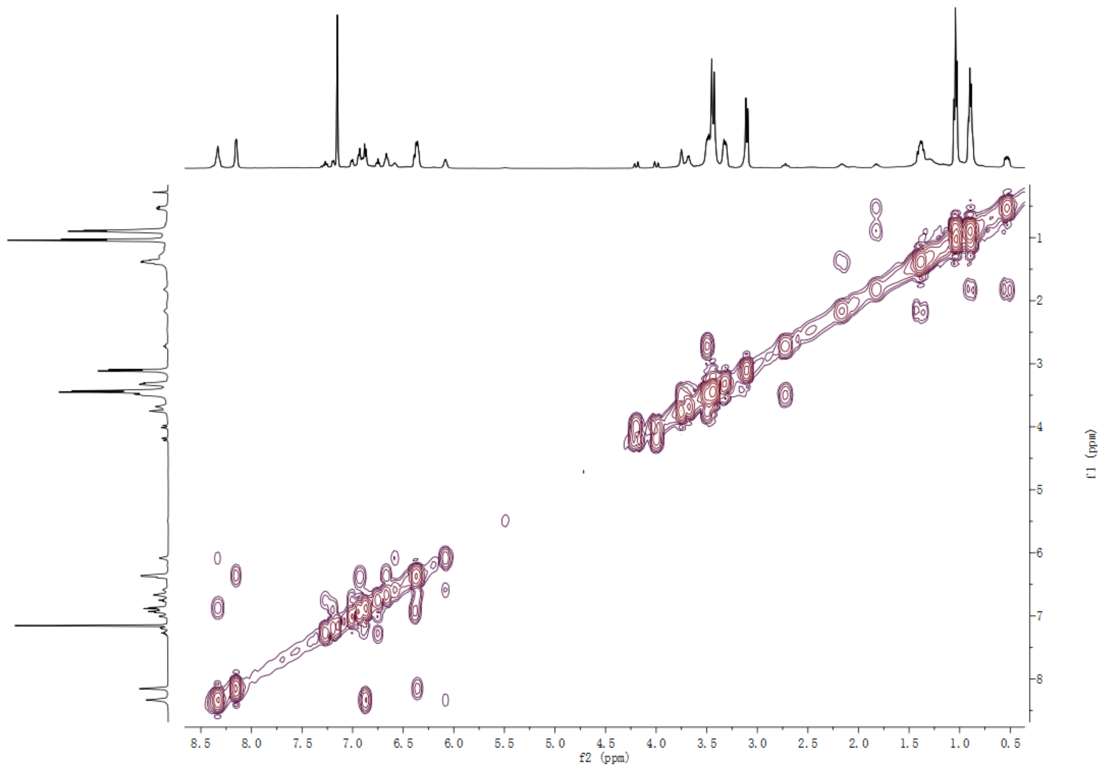

Figure S105. ^{1}H-^{1}H COSY NMR (500 MHz, C_6D_6) spectrum of **LS-Ru-5**

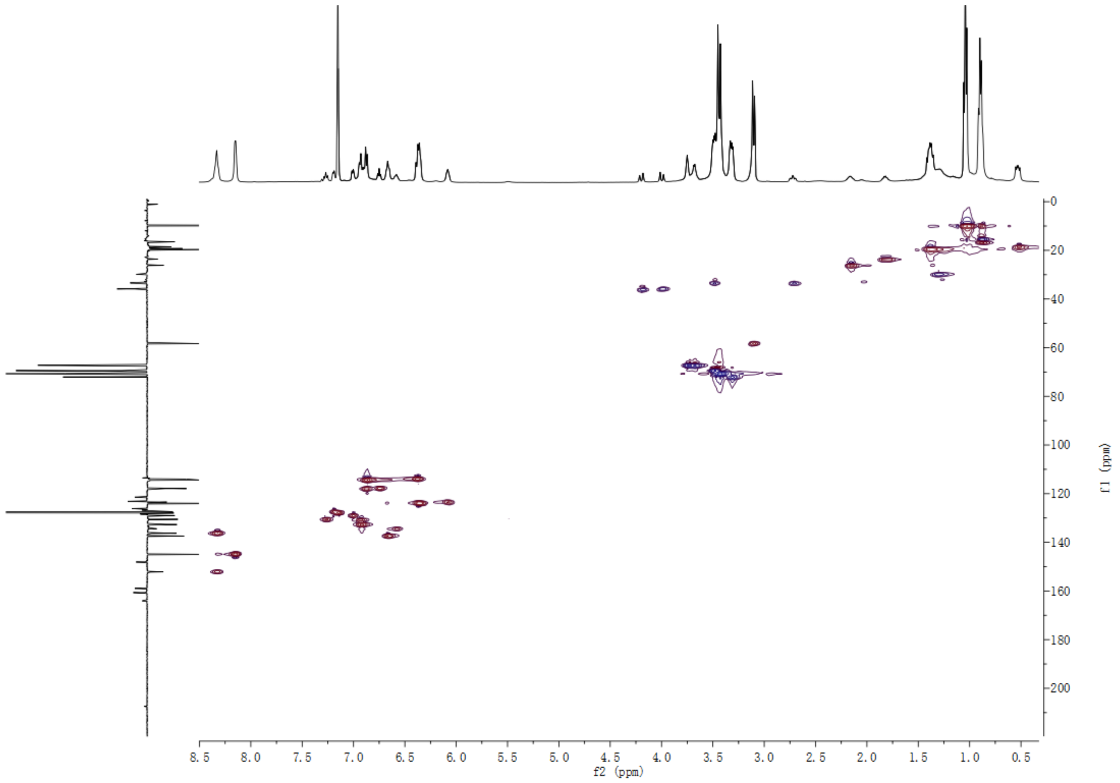

Figure S106. ^{1}H-^{13}C HSQC NMR (500 MHz, C_6D_6) spectrum of complex **LS-Ru-5**

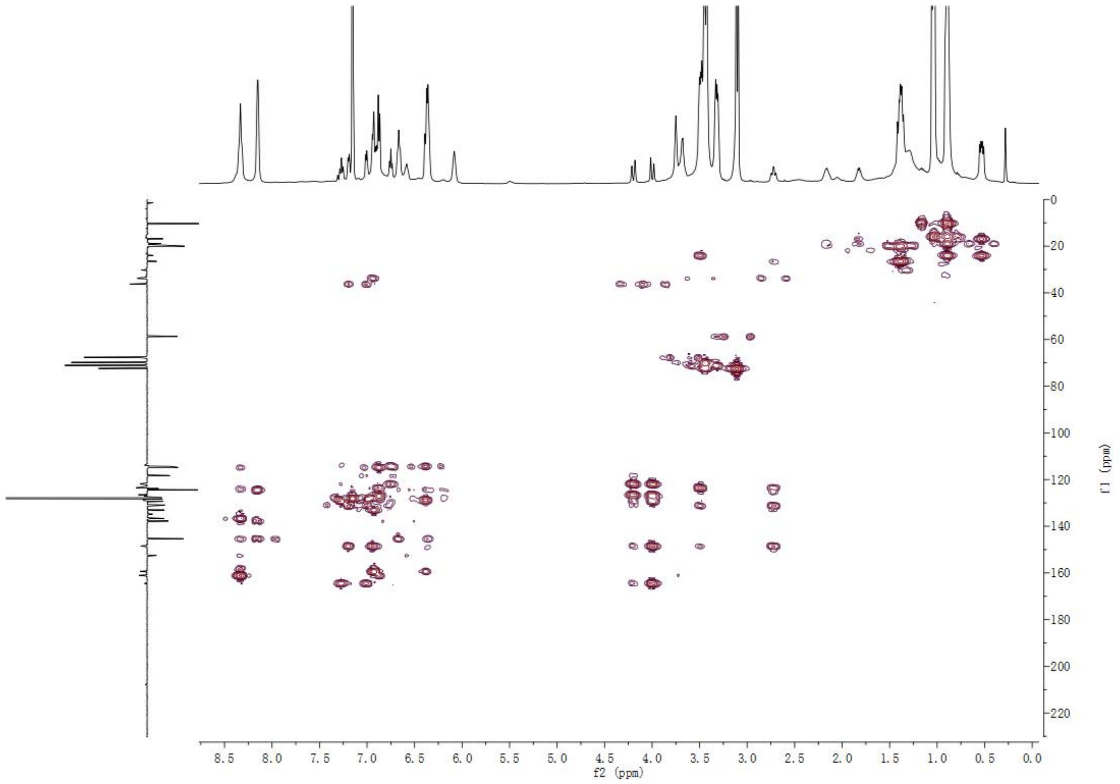

Figure S107. ^{1}H-^{13}C HMBC NMR (500 MHz, C_6D_6) spectrum of complex **LS-Ru-5**

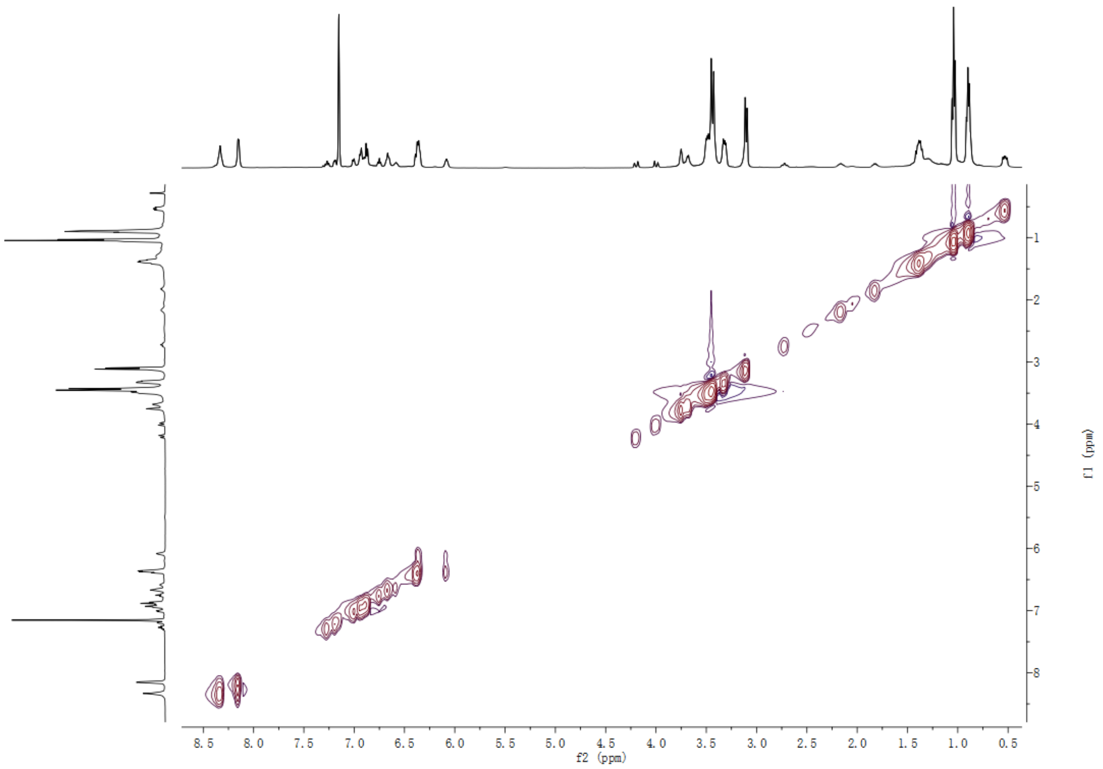

Figure S108. ^{1}H-^{1}H NOESY NMR (500 MHz, C_6D_6) spectrum of **LS-Ru-5**

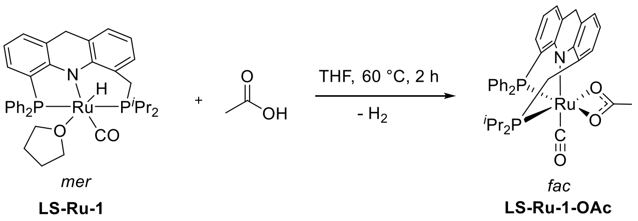

Formation of the acetate complex LS-Ru-1-OAc:

Complex **LS-Ru-1** (13.9 mg, 0.020 mmol) was dissolved in 3 mL of THF in a 15 mL sealed tube. To this solution was added dropwise a solution of 1.2 equiv. of acetic acid in THF (0.024 mmol, 240 μL, 1M). The tube was sealed and taken out of the glovebox, and stirred at 60 ˚C for 2 hours. After cooling the reaction mixture to room temperature, the sealed tube was taken into the glovebox again and the solvent was removed under vacuum. The resulting solid was washed two times with 1 mL of cold pentane to afford complex **LS-Ru-1-OAc** as an orange brown solid (12.1 mg, 88% yield). Crystals suitable for X-ray analysis were obtained by slow evaporation of a Et_2O/n-hexane solution of **LS-Ru-1-OAc**.

^{31}P NMR (202 MHz, C_6D_6) δ 65.01 (d, J = 29.5 Hz), 56.48 (d, J = 29.5 Hz).

^{1}H NMR (500 MHz, C_6D_6) δ 7.99 (dd, *J* = 11.2, 8.2 Hz, 2H, aryl), 7.69 (dd, *J* = 10.2, 8.5 Hz, 2H, aryl), 7.08 – 7.03 (m, 3H, aryl), 7.01 – 6.91 (m, 5H, aryl), 6.91 – 6.84 (m, 3H, aryl), 6.64 (t, *J* = 7.2 Hz, 1H, aryl), 4.03 (d, *J* = 16.2 Hz, 1H, ArC***H***$_2$Ar), 3.84 (dd, *J* = 16.7, 11.7 Hz, 1H, C***H***$_2$P), 3.79 (d, *J* = 16.8 Hz, 1H, ArC***H***$_2$Ar), 2.35 (t, *J* = 15.6 Hz, 1H, C***H***$_2$P), 1.67 (s, 3H, OOCC***H***$_3$), 1.58 – 1.49 (m, 1H, PC***H***(CH$_3$)$_2$), 1.38 (dd, *J* = 15.2, 6.7 Hz, 3H, PCH(C***H***$_3$)$_2$), 0.89 (dd, *J* = 16.0, 6.9 Hz, 3H, PCH(C***H***$_3$)$_2$), 0.44 – 0.38 (m, 3H, PCH(C***H***$_3$)$_2$), 0.30 – 0.20 (m, 4H, PC***H***(CH$_3$)$_2$, PCH(C***H***$_3$)$_2$).

^{13}C NMR (126 MHz, C_6D_6) δ 201.74 (dd, *J* = 15.3, 12.0 Hz, Ru-***C***O), 189.73 (t, *J* = 2.5 Hz, OO***C***CH$_3$), 162.36 (d, *J* = 21.1 Hz, Ar), 148.53 (d, *J* = 3.1 Hz, Ar), 136.25 (d, *J* = 59.0 Hz, Ar), 135.68 (dd, *J* = 49.2, 1.3 Hz, Ar), 133.74 (s, Ar), 133.65 (s, Ar), 133.17 (s, Ar), 133.09 (s, Ar), 130.73 (s, Ar), 130.51 (d, *J* = 2.6 Hz, Ar), 130.20 (d, *J* = 2.4 Hz, Ar), 130.05 (d, *J* = 1.9 Hz, Ar), 129.74 (d, *J* = 9.8 Hz, Ar), 128.71 (s, Ar), 128.62 (s, Ar), 128.19 (s, Ar), 128.11 (s, Ar), 126.96 (d, *J* = 11.2 Hz, Ar), 126.58 (s, Ar), 125.83 (d, *J* = 1.0 Hz, Ar), 122.67 (d, *J* = 1.7 Hz, Ar), 118.34 (s, Ar), 118.27 (s, Ar), 114.35 (d, *J* = 50.5 Hz, Ar), 35.42 (d, *J* = 1.6 Hz, Ar***C***H$_2$Ar), 26.99 (d, *J* = 30.4 Hz, P***C***H(CH$_3$)$_2$), 24.56 (s, OOC***C***H$_3$), 23.95 (d, *J* = 24.4 Hz, P***C***H(CH$_3$)$_2$), 23.67 (d, *J* = 26.3 Hz, ***C***H$_2$P), 19.34 (s, PCH(***C***H$_3$)$_2$), 17.99 (s, PCH(***C***H$_3$)$_2$), 16.89 (d, *J* = 2.8 Hz, PCH(***C***H$_3$)$_2$), 16.51 (d, *J* = 7.0 Hz, PCH(***C***H$_3$)$_2$).

IR (KBr) = 1941 cm^{-1} (CO), 1459 cm^{-1} (COO), 1414 cm^{-1} (COO).

HRMS (ESI): Exact mass calculated for $C_{33}H_{32}NOP_2Ru^+$ ([M-HOAc-H]$^+$): 622.0997, mass found: 622.1016.

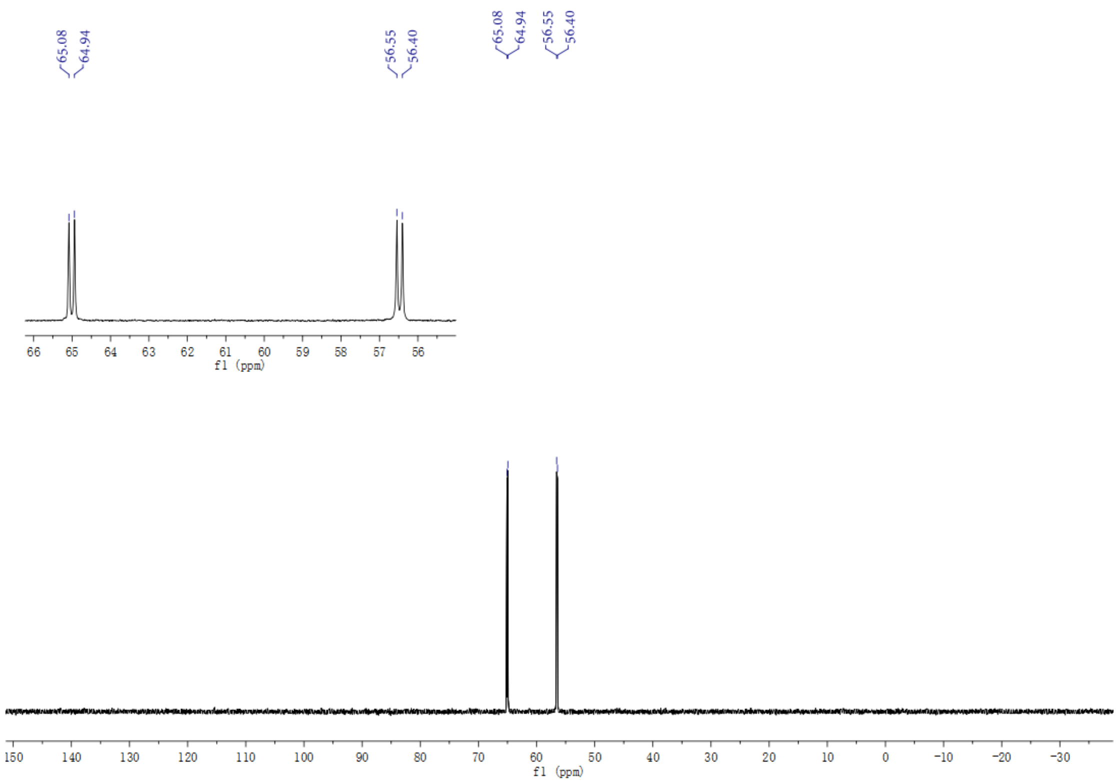

Figure S109. ^{31}P NMR (202 MHz, C_6D_6) spectrum of **LS-Ru-1-OAc**

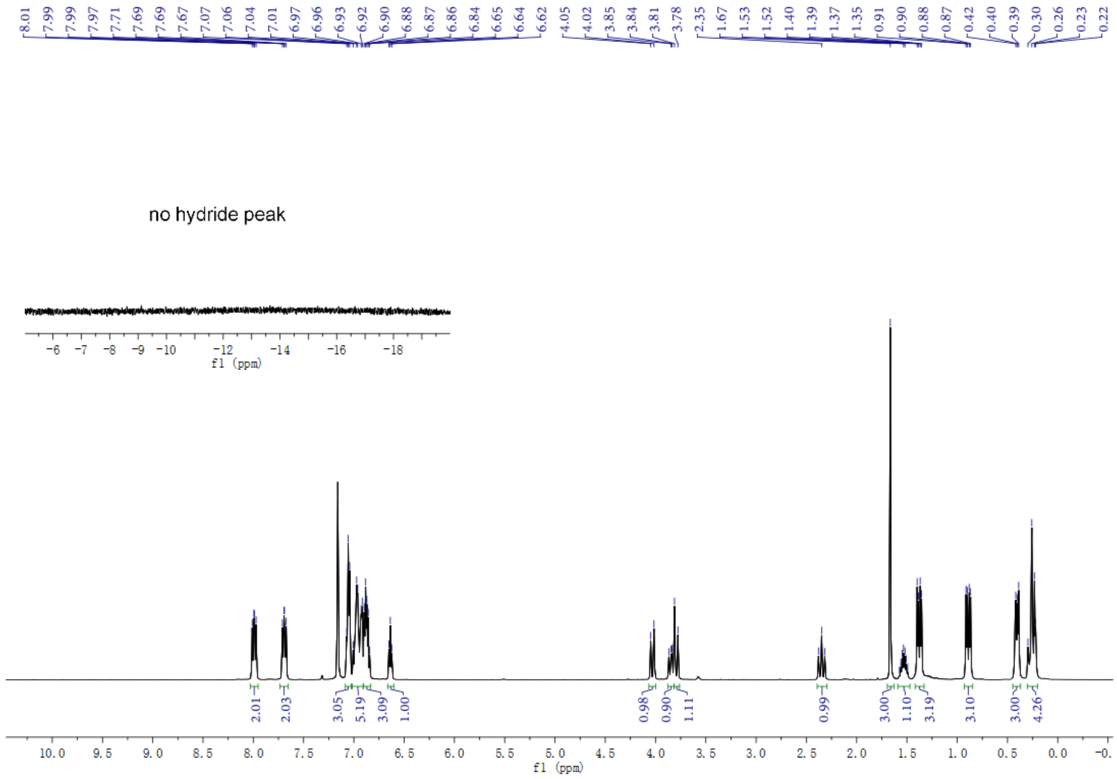

Figure S110. ^{1}H NMR (500 MHz, C_6D_6) spectrum of **LS-Ru-1-OAc**

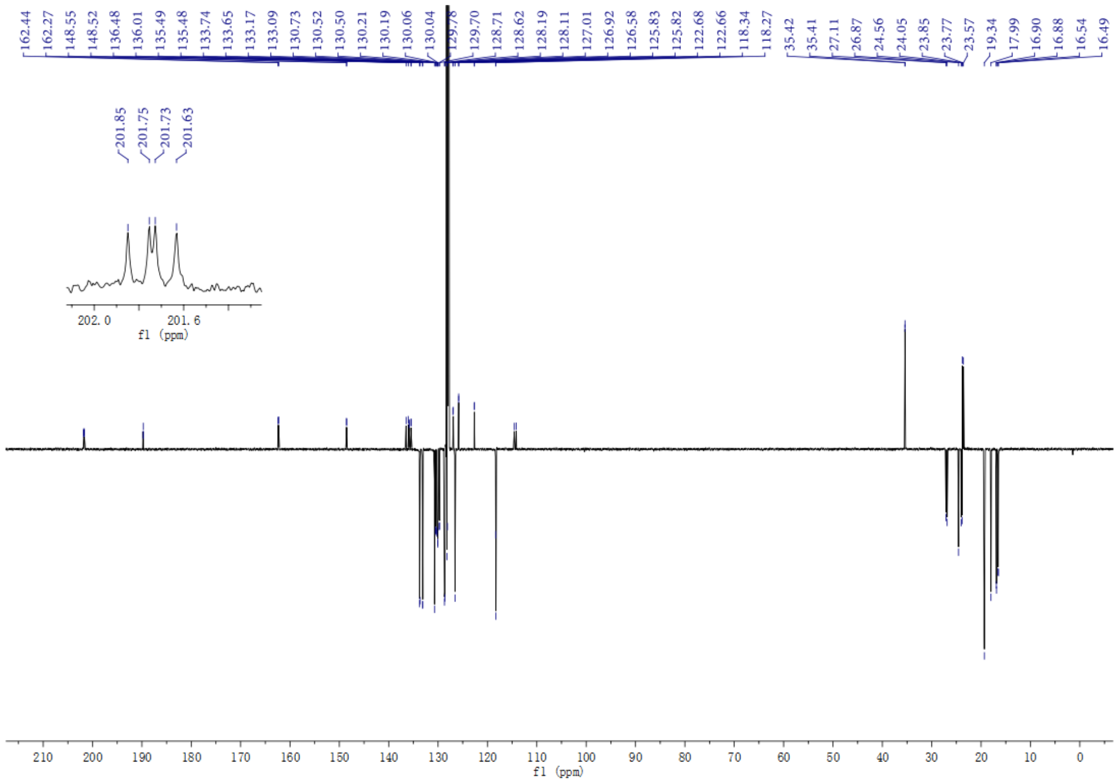

Figure S111. ^{13}C-DEPTQ NMR (126 MHz, C_6D_6) spectrum of **LS-Ru-1-OAc**

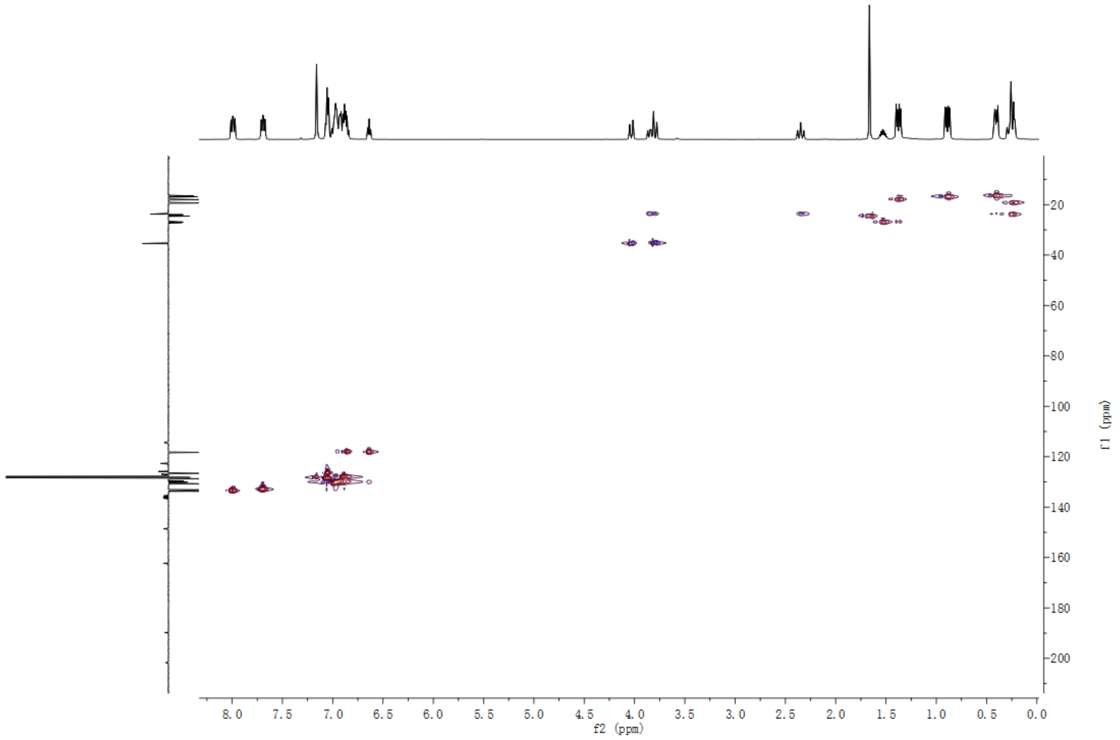

Figure S112. ^{1}H-^{13}C HSQC NMR (500 MHz, C_6D_6) spectrum of complex **LS-Ru-1-OAc**

The diffraction data from single crystals of **LS-Ru-1-OAc** were at 100 K on on a Rigaku Synergy-R diffractometer equipped with a HyPix ARC 150° detector and CuKα (λ=1.54184Å). All datasets were processed with CrysAlisPRO and structures were solved with SHELXT[9]. All non-hydrogen atoms were further refined by SHELXL[10] with anisotropic displacement coefficients. Hydrogens were placed in calculated positions and refined in a riding mode. Hydride atoms were located in the electron density map, incorporated and refined. Refinement was carried out with the OLEX-2[11] GUI. Crystallographic data and refinement parameters are summarized in Table S5.

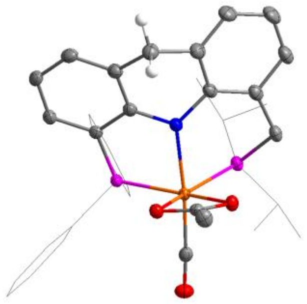

Figure S113. X-ray crystal structure of **LS-Ru-1-OAc**

Table S5. Crystallographic data for complex **LS-Ru-1-OAc**

**CCDC No.**	2382563
**Formula**	$C_{35}H_{37}NO_3P_2Ru + 0.5C_6H_{14}$
**Molecular weight**	725.75
**Crystal system**	Orthorhombic
**Space group**	*Pbca*
**Crystal size (mm)**	0.333×0.135×0.021
**Crystal color and shape**	Orange prism
**Temperature (K)**	100
**Wavelength (Å)**	1.54184
**a (Å)**	10.89264(5)
**b (Å)**	16.70247(8)
**c (Å)**	37.71001(19)

**α (º)**	90
**β (º)**	90
**γ (º)**	90
**Volume (Å^3)**	6860.73(6)
**Z**	8
**r_{calcd} (g · cm^{-1})**	1.405
**μ (mm^{-1})**	4.873
**No. of reflections (unique)**	173888 (7042)
**R_{int}**	0.0521
**Completeness to θ (%)**	99.4
**θ max**	75.153
**Data / restraints / parameters**	7042 / 0 / 426
**Goodness-of-fit on F^2**	1.038
**Final R_1 and wR_2 indices [I>2s(I)]**	0.0250, 0.0674
**R_1 and wR_2 indices (all data)**	0.0278, 0.0697
Highest diff Peak and Deepest hole	0.621, -0.769

4. Base-free dehydrogenative coupling of EG using a mixed solvent

General procedure: In a glovebox, ethylene glycol (62.1 mg, 1.0 mmol) was added into a 100 mL Schlenk tube equipped with a magnetic stirring bar through a glass pipette. A 5 mL vial containing a magnetic stirring bar was charged with the ruthenium pincer complex (0.005 mmol) and dry and degassed dimethoxyethane (0.5 mL) and the solution was transferred into the above Schlenk tube using the same glass pipette. The vial was washed with toluene (2 * 0.25 mL) and the solution was transferred into the Schlenk tube. The Schlenk tube was taken out of the glovebox and stirred at 150 ˚C for 72 hours. Then the reaction mixture was firstly cooled to room temperature, and then the Schlenk tube was connected to the gas collecting system to measure the volume of gas. Finally, the solvent was removed under vacuum, mesitylene was added into Schlenk tube as an internal standard. The residue was dissolved in d_6-Acetone, and the resulting solution was passed through a short Celite column and then submitted to NMR analysis.

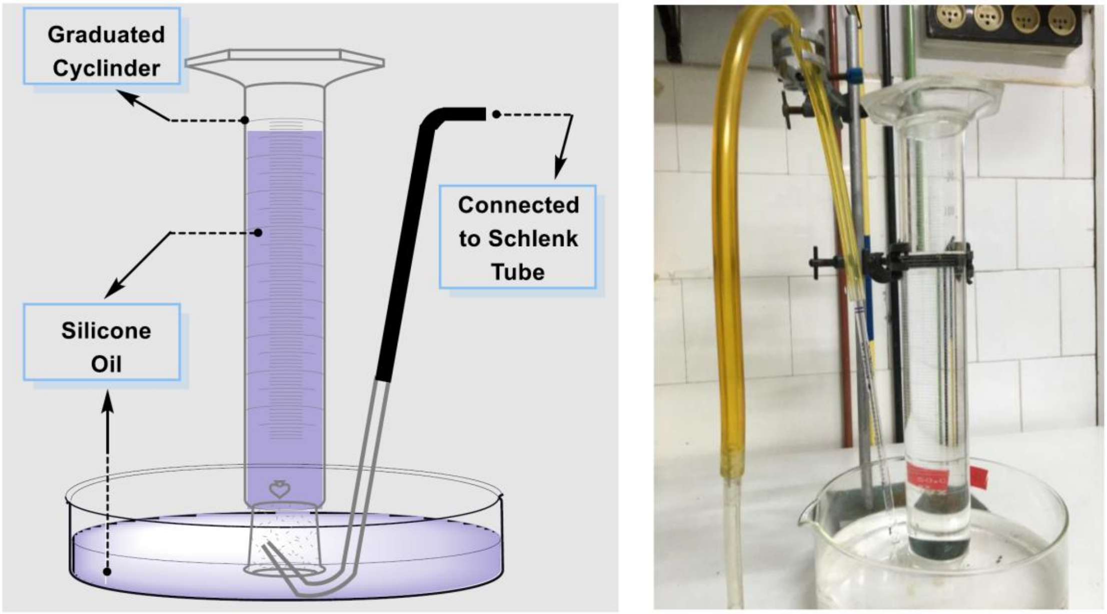

Figure S114. Schematic drawing and sideview of the gas collecting system

Table S6. Long-short-arm acridine Ru-pincer complexes catalyzed base-free dehydrogenative coupling of EG.

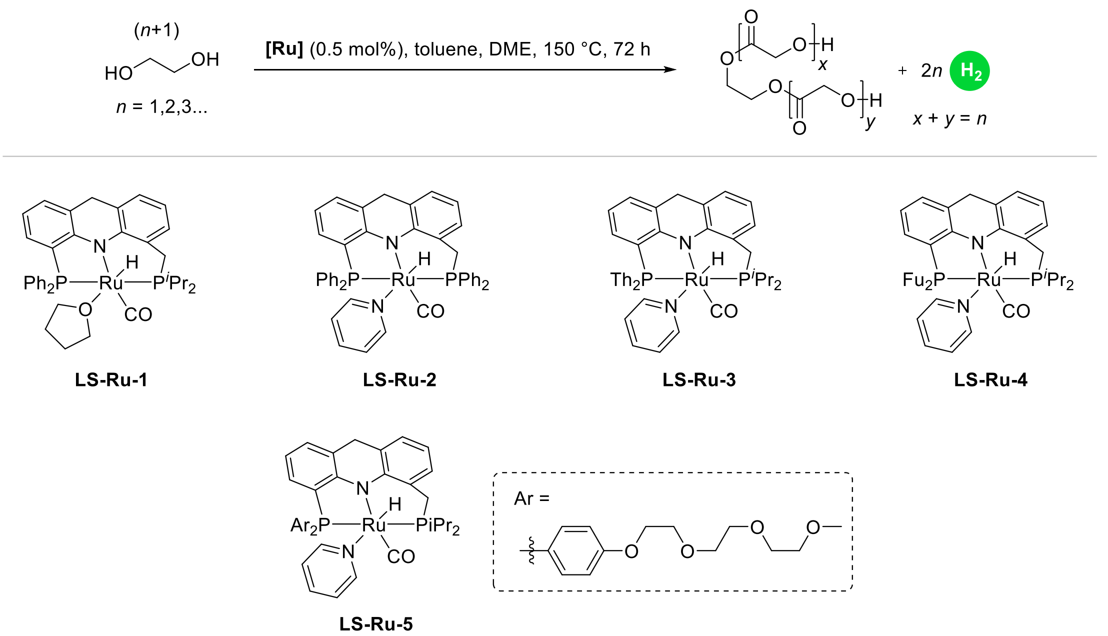

entry	[Ru]	Conv. (%)	V (H_2, mL)
1	**LS-Ru-1**	>99	44
2	**LS-Ru-2**	94	30
3	**LS-Ru-3**	>99	46
4	**LS-Ru-4**	94	28
5	**LS-Ru-5**	>99	44

Reaction conditions: ethylene glycol (1.0 mmol), **[Ru]** (0.5 mol%), toluene (0.5 mL)/DME (0.5 mL) at 150 °C (bath temperature) for 72 hours. Conversions were determined by ^{1}H NMR of the reaction mixture using mesitylene as an internal standard. Th = 2-Thienyl, Fu = 2-Furyl.

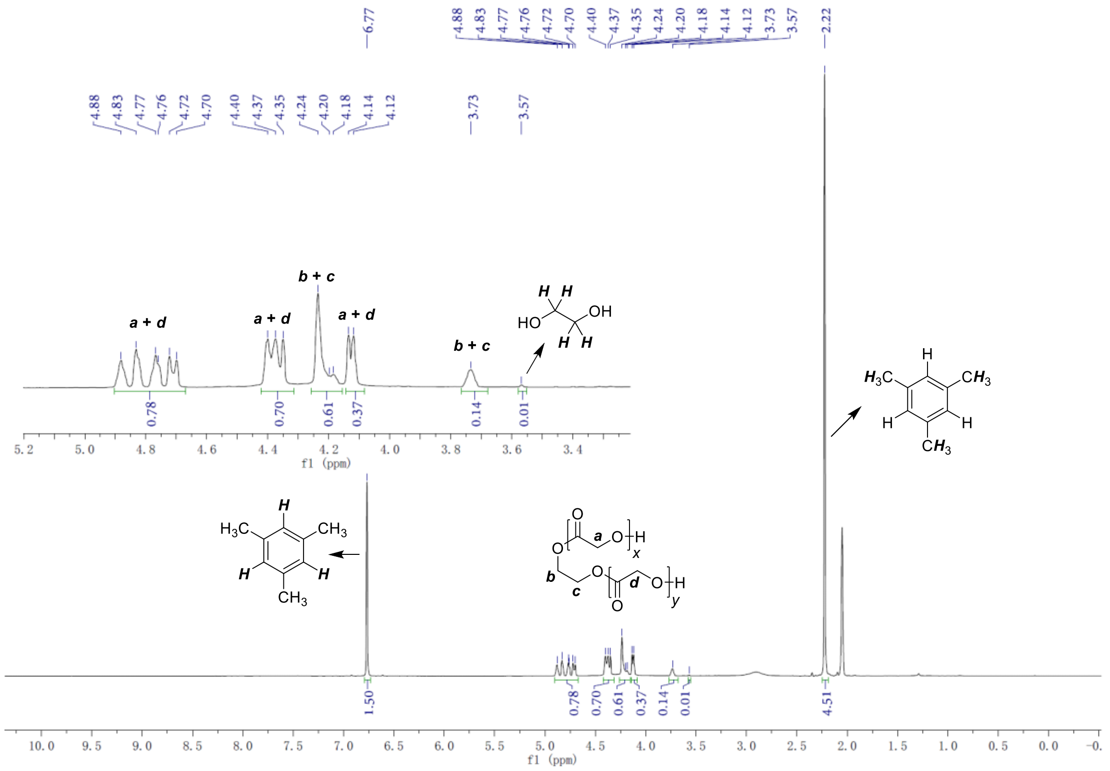

Figure S115. ^{1}H NMR (500 MHz, acetone-d_6) spectrum of reaction mixture of Table S6, entry 1 (0.5 mmol mesitylene as the internal standard)

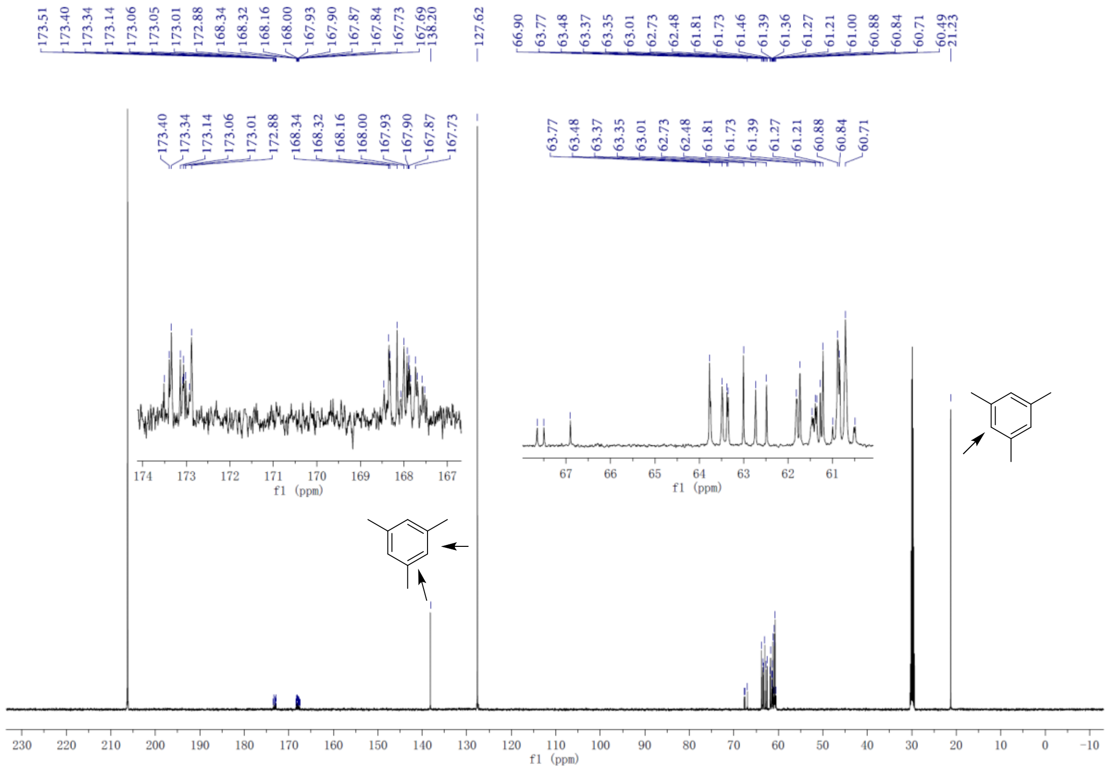

Figure S116. ^{13}C NMR (126 MHz, acetone-d_6) spectrum of reaction mixture of Table S6, entry 1 (0.5 mmol mesitylene as the internal standard)

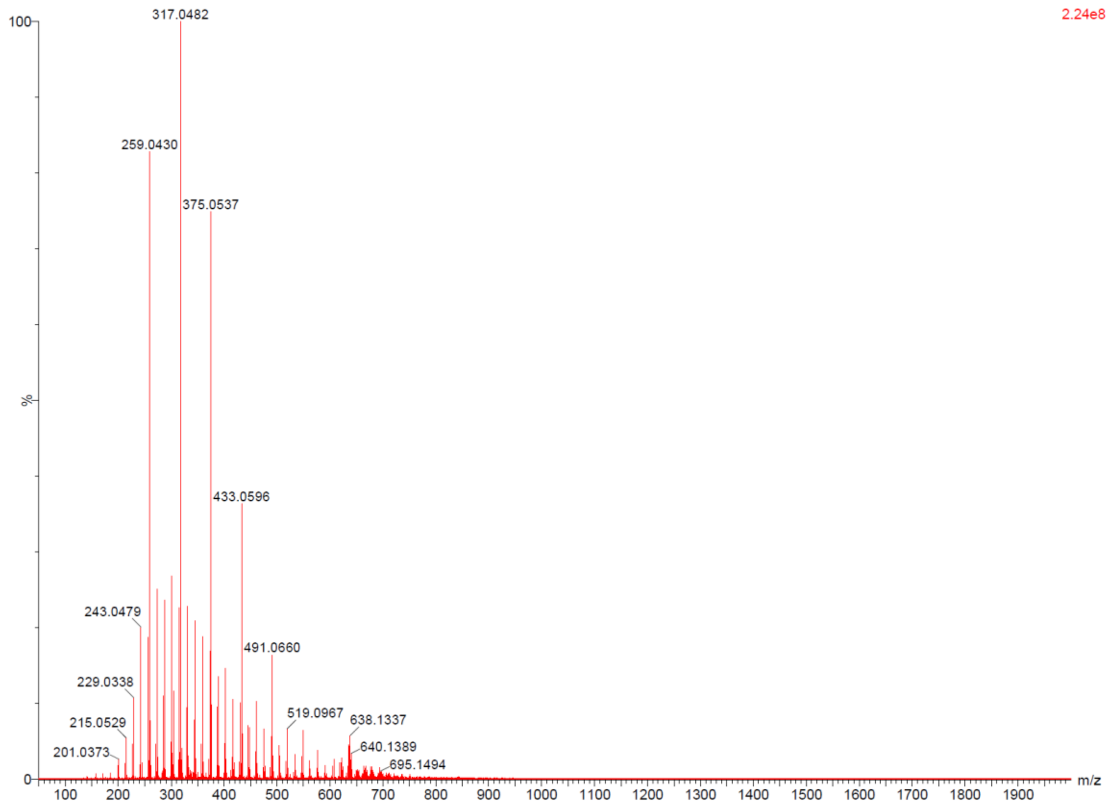

n	Calc. Mass $[M+Na]^+$	Mass $[M+Na]^+$
1	143.0315	Not detected
2	201.0370	201.0373
3	259.0424	259.0430
4	317.0479	317.0482
5	375.0534	375.0537
6	433.0589	433.0596
7	491.0644	491.0660
8	549.0698	549.0726

Figure S117. Mass spectrum of reaction mixture of Table S6, entry 1

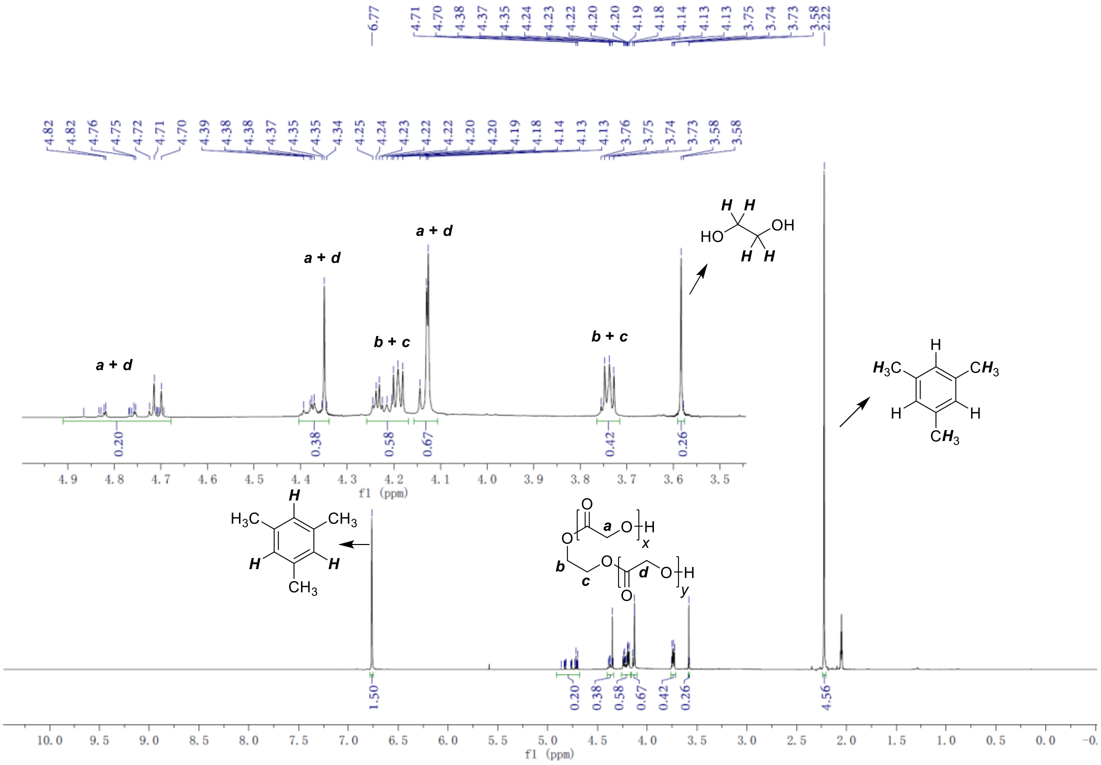

Figure S118. ^{1}H NMR (500 MHz, acetone-d_6) spectrum of reaction mixture of Table S6, entry 2 (0.5 mmol mesitylene as the internal standard)

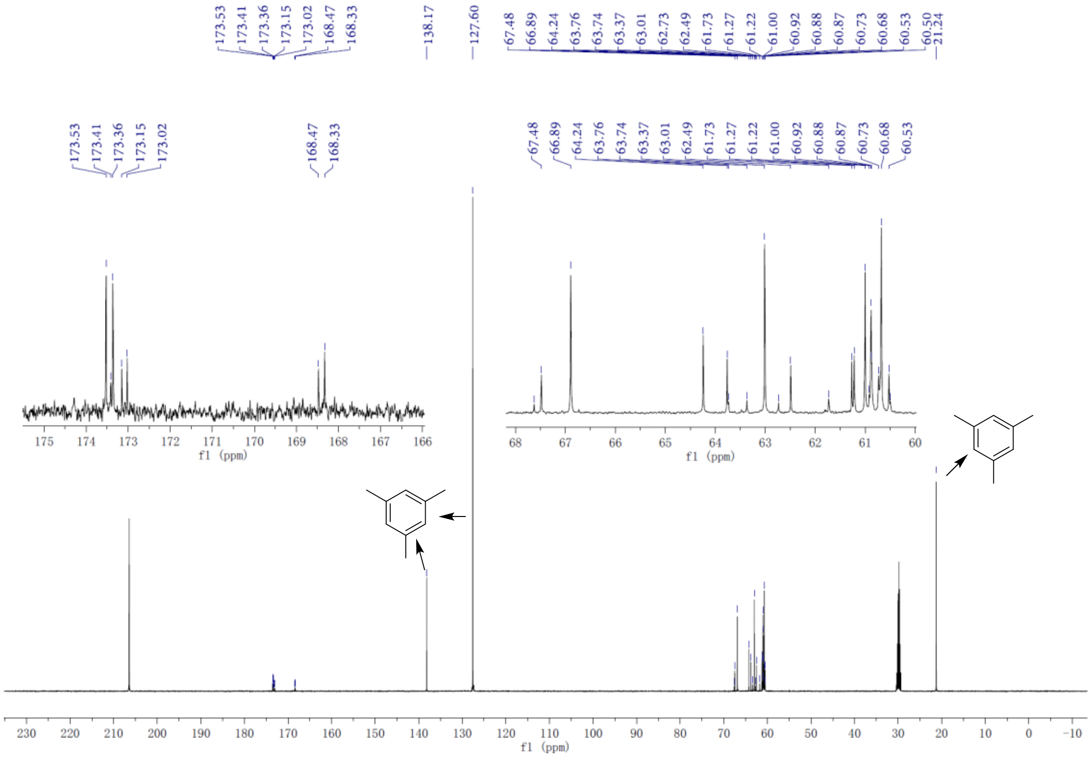

Figure S119. ^{13}C NMR (126 MHz, acetone-d_6) spectrum of reaction mixture of Table S6, entry 2 (0.5 mmol mesitylene as the internal standard)

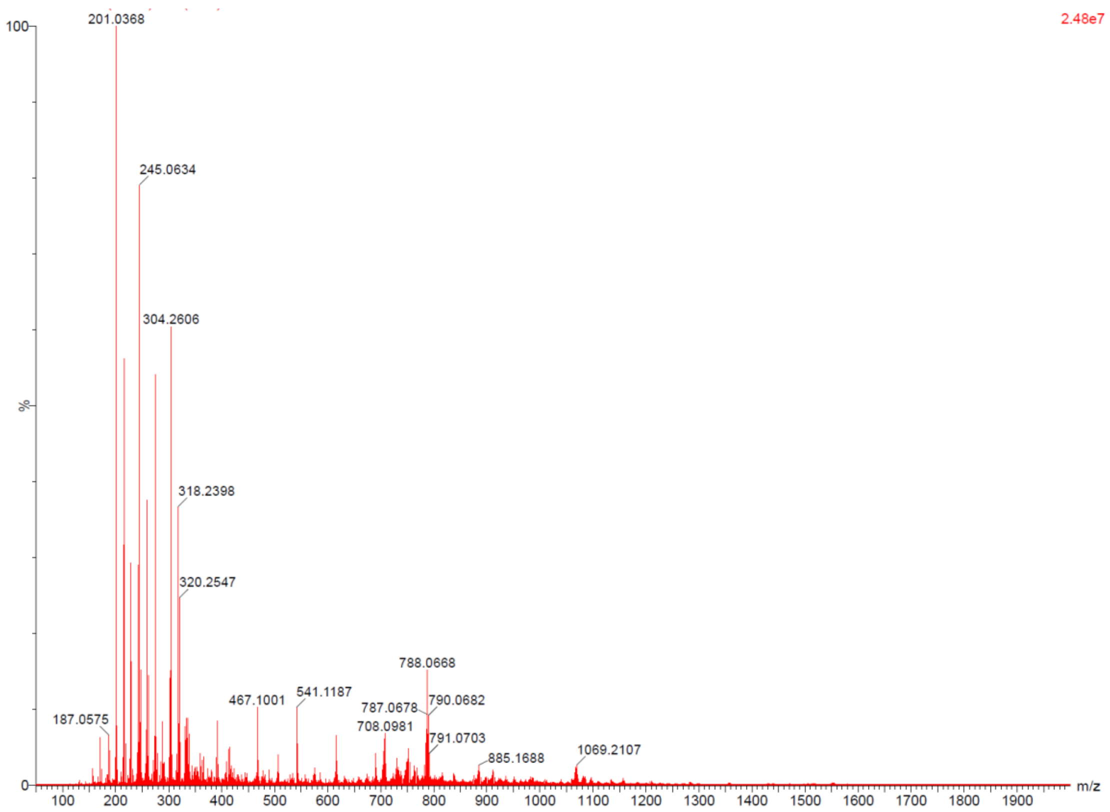

n	Calc. Mass $[M+Na]^+$	Mass $[M+Na]^+$
1	143.0315	Not detected
2	201.0370	201.0368
3	259.0424	259.0426

Figure S120. Mass spectrum of reaction mixture of Table S6, entry 2

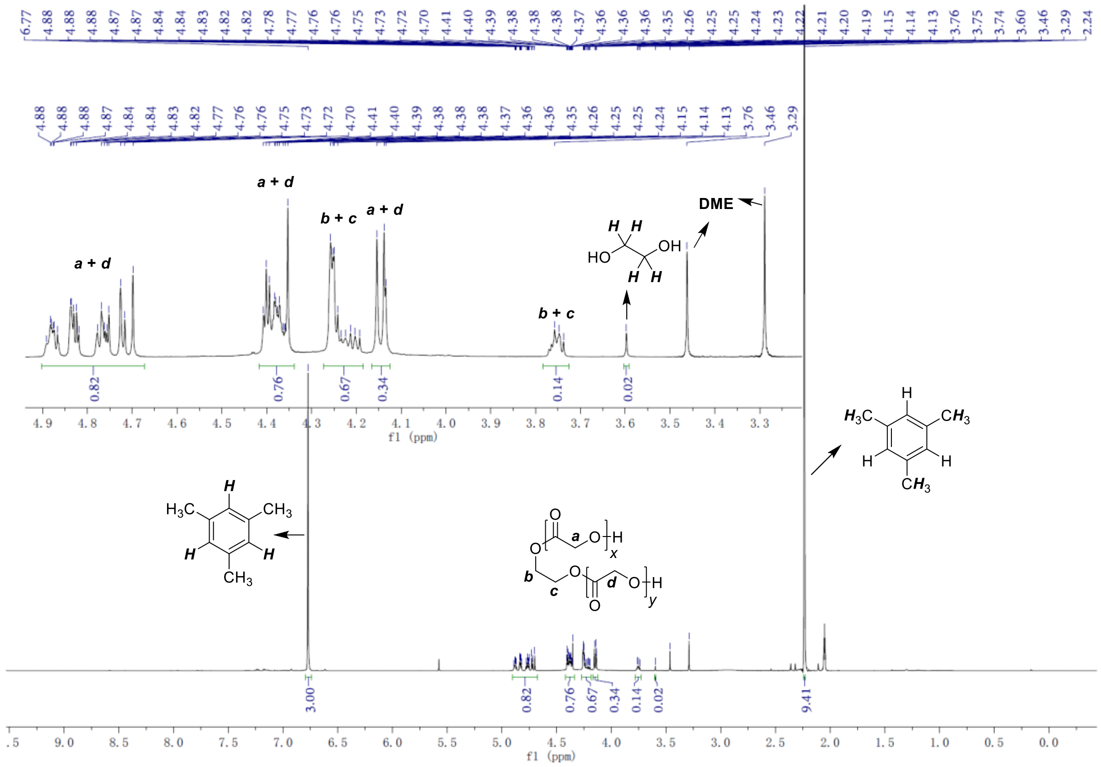

Figure S121. ^{1}H NMR (500 MHz, acetone-d_6) spectrum of reaction mixture of Table S6, entry 3 (1.0 mmol mesitylene as the internal standard)

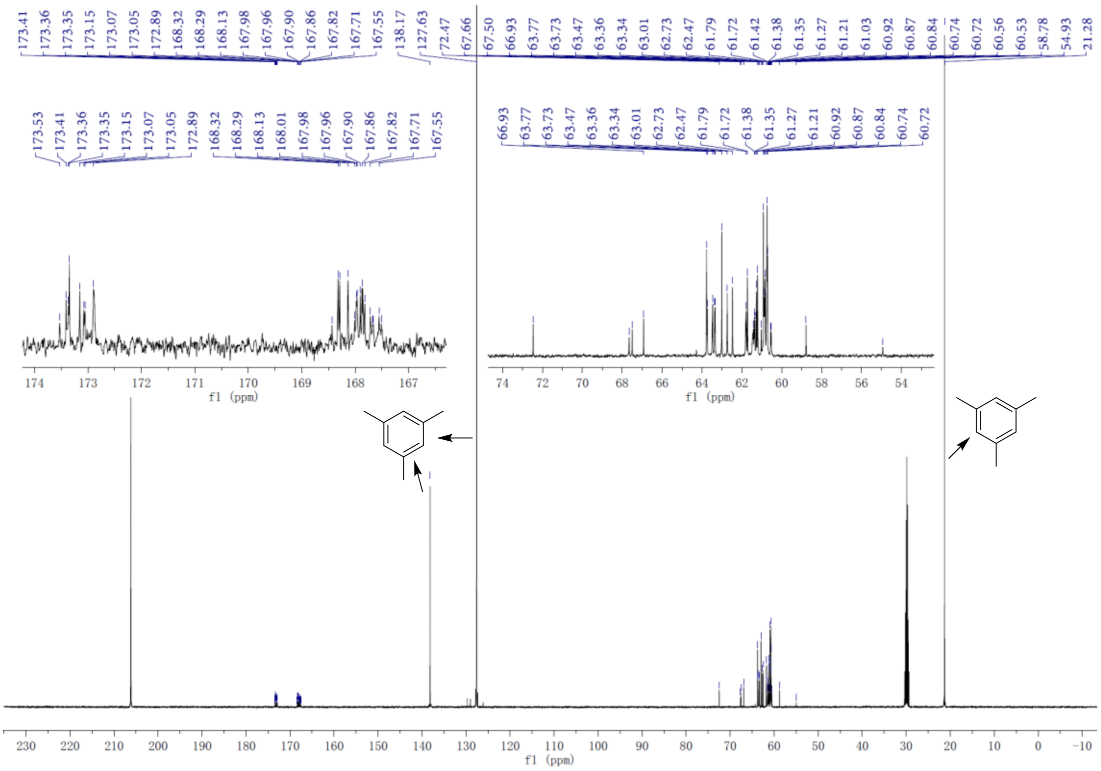

Figure S122. ^{13}C NMR (126 MHz, acetone-d_6) spectrum of reaction mixture of Table S6, entry 3 (1.0 mmol mesitylene as the internal standard)

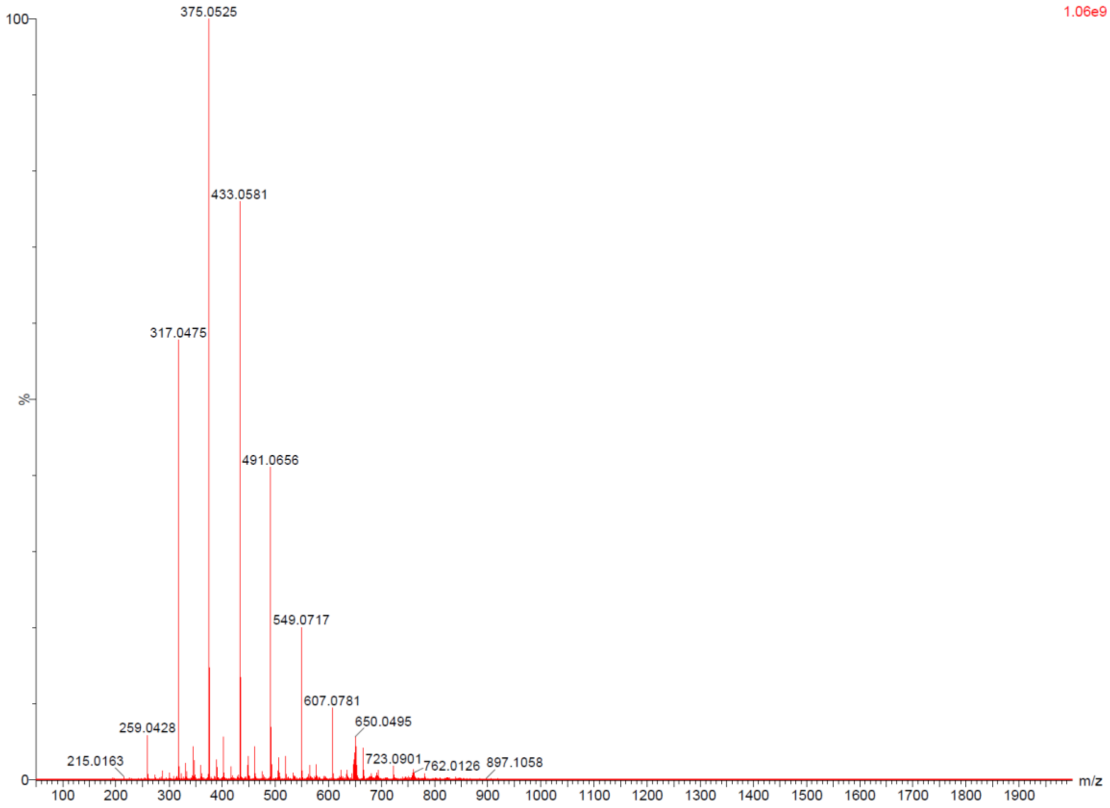

n	Calc. Mass $[M+Na]^+$	Mass $[M+Na]^+$
1	143.0315	Not detected
2	201.0370	201.0367
3	259.0424	259.0427
4	317.0479	317.0474
5	375.0534	375.0525
6	433.0589	433.0581
7	491.0644	491.0656
8	549.0698	549.0718
9	607.0753	607.0781
10	665.0808	665.0843
11	723.0863	723.0900
12	781.0918	781.0958
13	839.0972	839.1005
14	897.1027	897.1058

Figure S123. Mass spectrum of reaction mixture of Table S6, entry 3

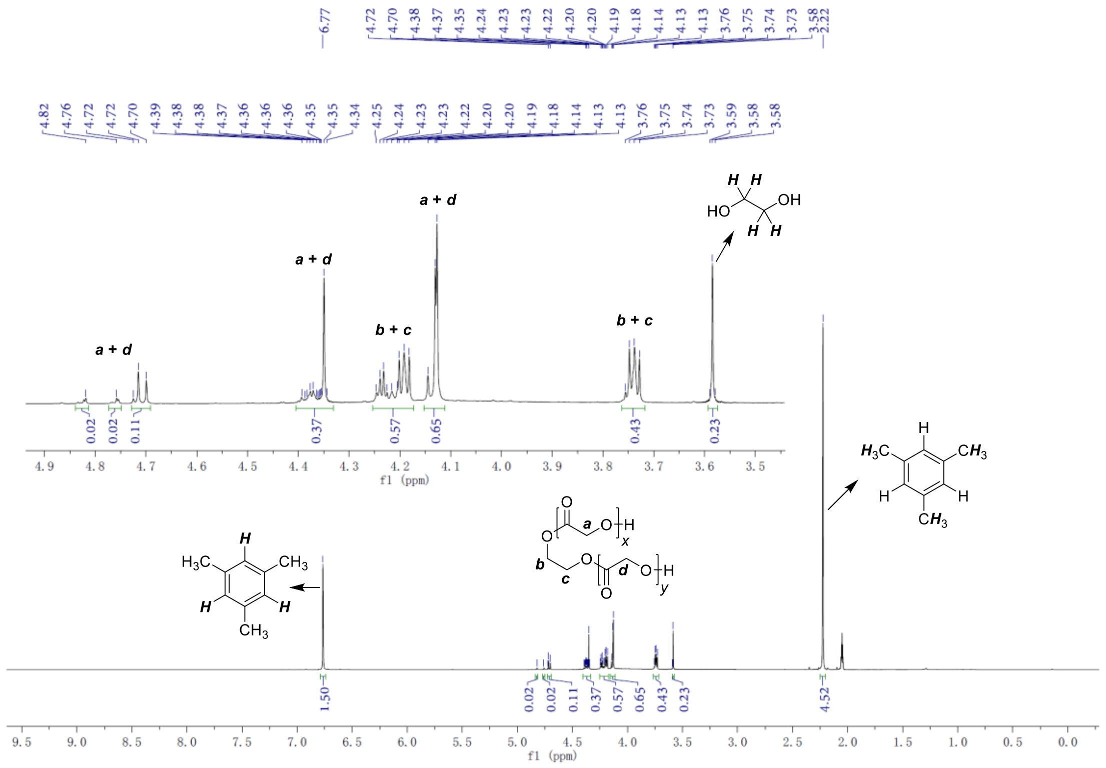

Figure S124. ^{1}H NMR (500 MHz, acetone-d_6) spectrum of reaction mixture of Table S6, entry 4 (0.5 mmol mesitylene as the internal standard)

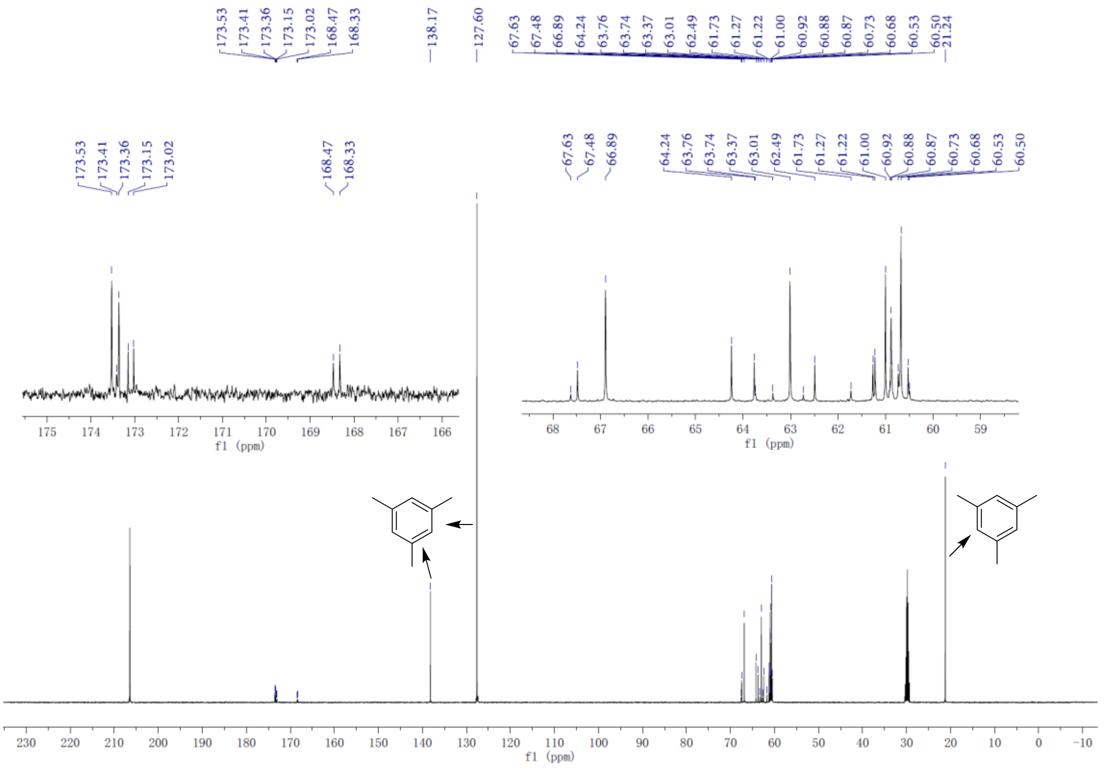

Figure S125. ^{13}C NMR (126 MHz, acetone-d_6) spectrum of reaction mixture of Table S6, entry 4 (0.5 mmol mesitylene as the internal standard)

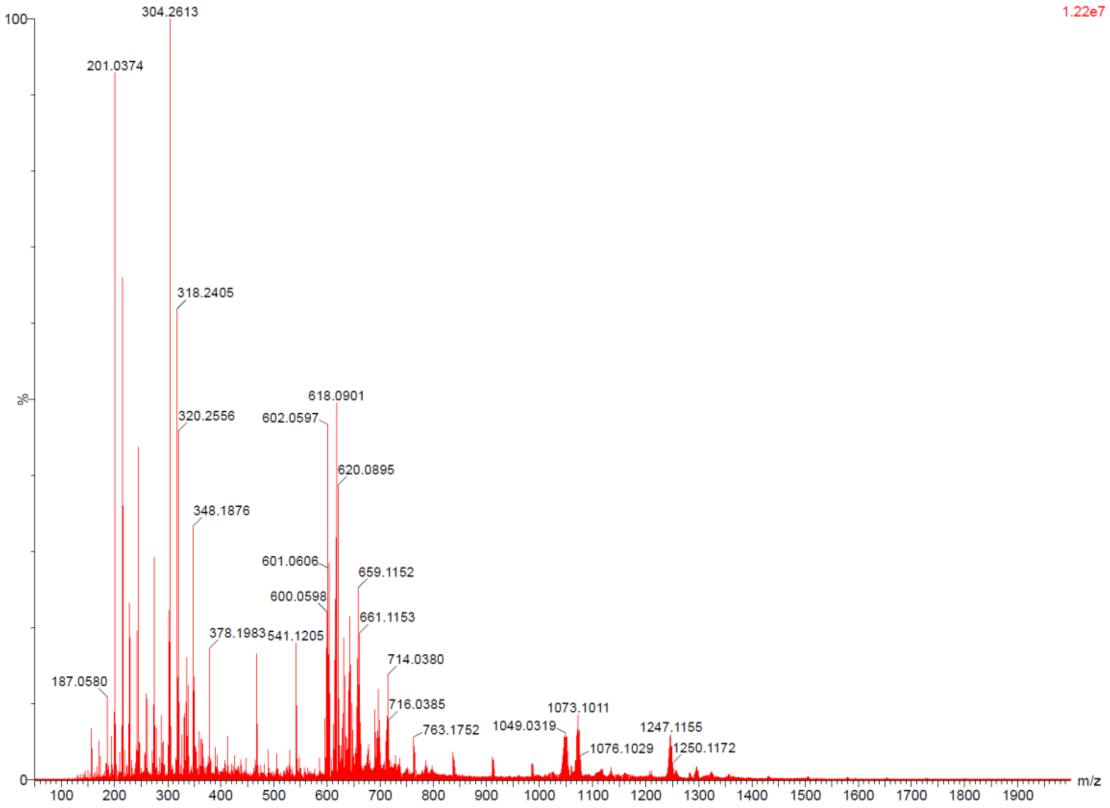

n	Calc. Mass $[M+Na]^+$	Mass $[M+Na]^+$
1	143.0315	Not detected
2	201.0370	201.0374
3	259.0424	259.0435

Figure S126. Mass spectrum of reaction mixture of Table S6, entry 4

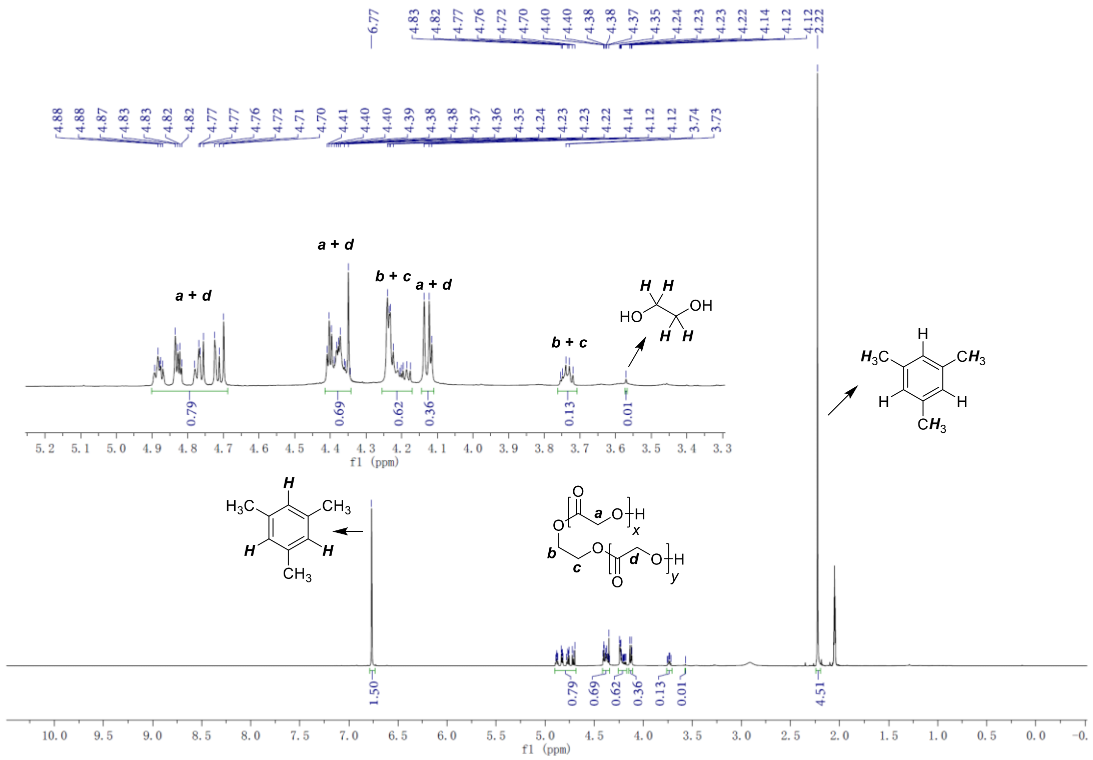

Figure S127. ^{1}H NMR (500 MHz, acetone-d_6) spectrum of reaction mixture of Table S6, entry 5 (0.5 mmol mesitylene as the internal standard)

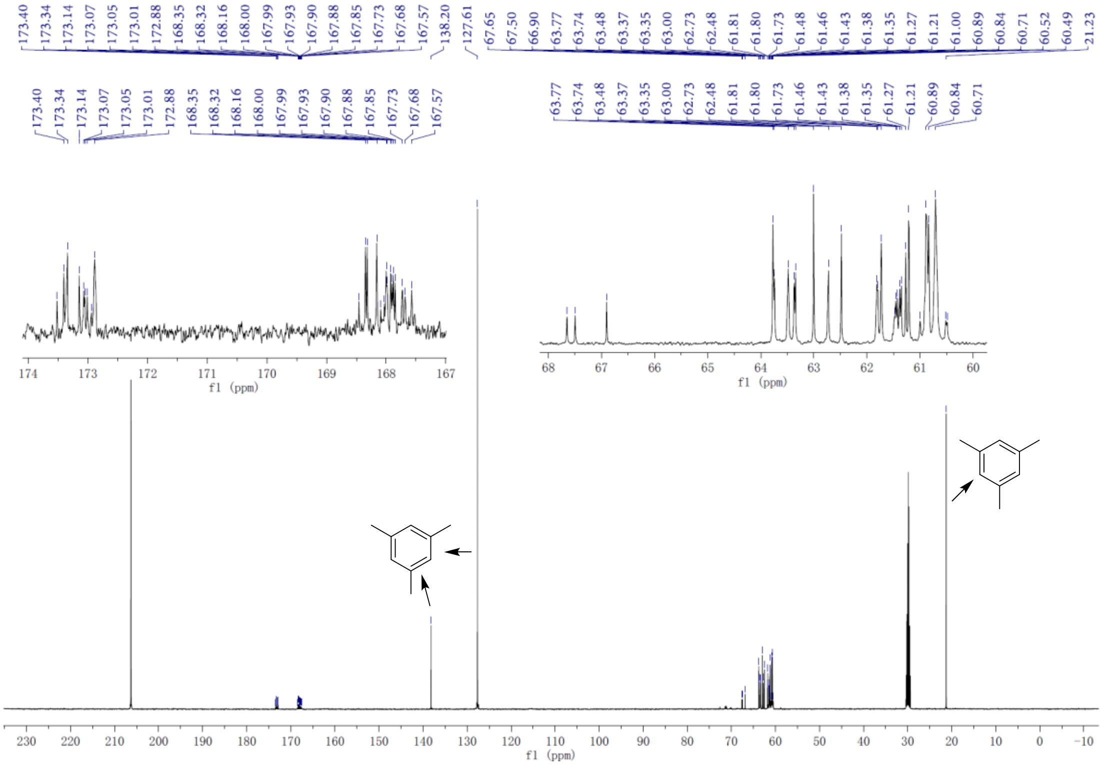

Figure S128. ^{13}C NMR (126 MHz, acetone-d_6) spectrum of reaction mixture of Table S6, entry 5 (0.5 mmol mesitylene as the internal standard)

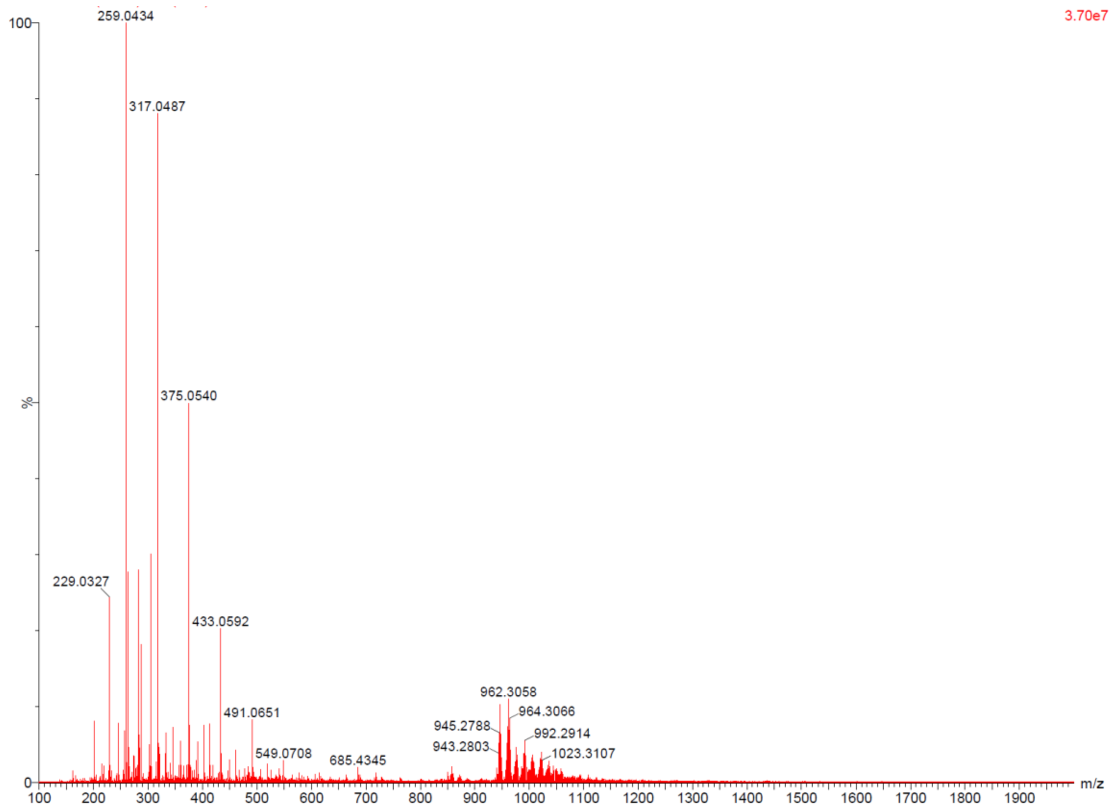

n	Calc. Mass [M+Na]$^+$	Mass [M+Na]$^+$
1	143.0315	Not detected
2	201.0370	201.0374
3	259.0424	259.0434
4	317.0479	317.0487
5	375.0534	375.0540
6	433.0589	433.0592
7	491.0644	491.0651
8	549.0698	549.0708
9	607.0753	607.0752

Figure S129. Mass spectrum of reaction mixture of Table S6, entry 5

Table S7. Dehydrogenative coupling of EG with different reaction times

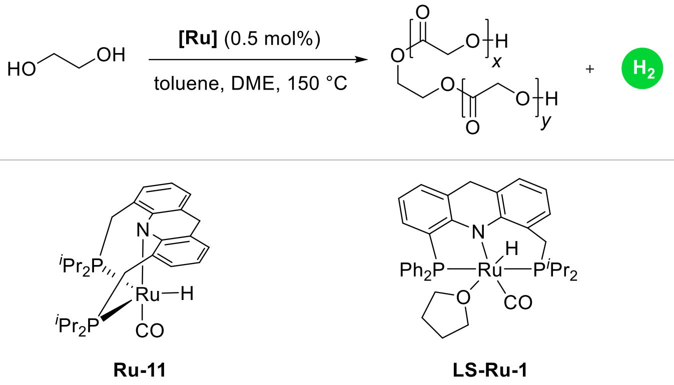

Entry	[Ru]	t (min)	Conv. (%)	V (H_2, mL)
1	**Ru-11**	10	4	1
2	**Ru-11**	20	9	2
3	**Ru-11**	30	13	3
4	**Ru-11**	60	28	7
5	**LS-Ru-1**	10	28	6
6	**LS-Ru-1**	20	35	8
7	**LS-Ru-1**	30	50	11
8	**LS-Ru-1**	60	75	18

Reaction conditions: EG (1.0 mmol), Ru catalyst (0.5 mol%), toluene (0.5 mL), 1,2-dimethoxyethane (0.5 mL) at 150 °C (bath temperature) for 10 - 60 minutes. Conversions were determined by ^{1}H NMR spectroscopy from the reaction mixture using mesitylene as an internal standard.

5. Continuous experiments with Ru-11

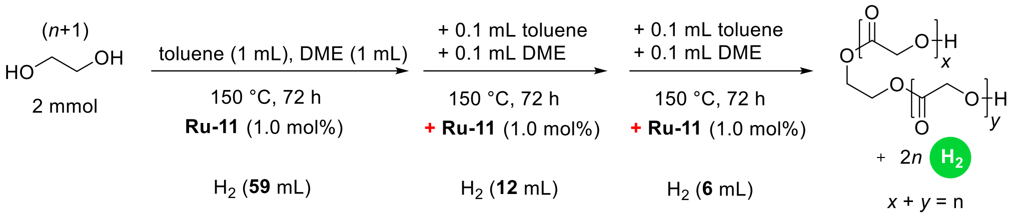

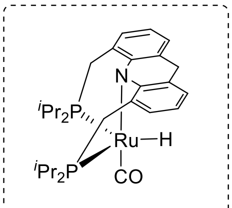

Ru-11

General procedure: In a glovebox, ethylene glycol (124.1 mg, 2.0 mmol) was added into a 100 mL Schlenk tube equipped with a magnetic stirring bar through a glass pipette. A 5 mL vial containing a magnetic stirring bar was charged with **Ru-11** (0.02 mmol) and dry and degassed dimethoxyethane (1.0 mL) and the solution was transferred into the above Schlenk tube using the same glass pipette. The vial was washed with toluene (2 * 0.5 mL) and the solution was transferred into the Schlenk tube. The Schlenk tube was taken out of the glovebox and stirred at 150 ˚C (oil bath temperature) for 72 hours. Then the reaction mixture was firstly cooled to room temperature and submitted to the gas collecting system for measurement of the volume of formed hydrogen (59 mL of H_2 was collected). The reaction tube was taken into the glovebox, and 0.02 mmol of **Ru-11** in toluene/DME (0.1 mL/0.1 mL) was added. The reaction mixture was stirred at 150 ˚C (oil bath temperature) for another 72 hours, then cooled to room temperature and the hydrogen evolved was collected (12 mL of H_2). The reaction tube was taken into the glovebox again, and 0.02 mmol of **Ru-11** in toluene/DME (0.1 mL/0.1 mL) was added. The reaction mixture was stirred at 150 ˚C (oil bath temperature) for another 72 hours, then cooled to room temperature and the hydrogen evolved was collected (6 mL of H_2). In total, we collected 77 mL of H_2. Then, the solvent was removed under vacuum, mesitylene was added into Schlenk tube as an internal standard. The residue was dissolved in acetone-d6, and the resulting solution was passed through a short Celite column and then submitted to NMR analysis. The EG conversion is >99%.

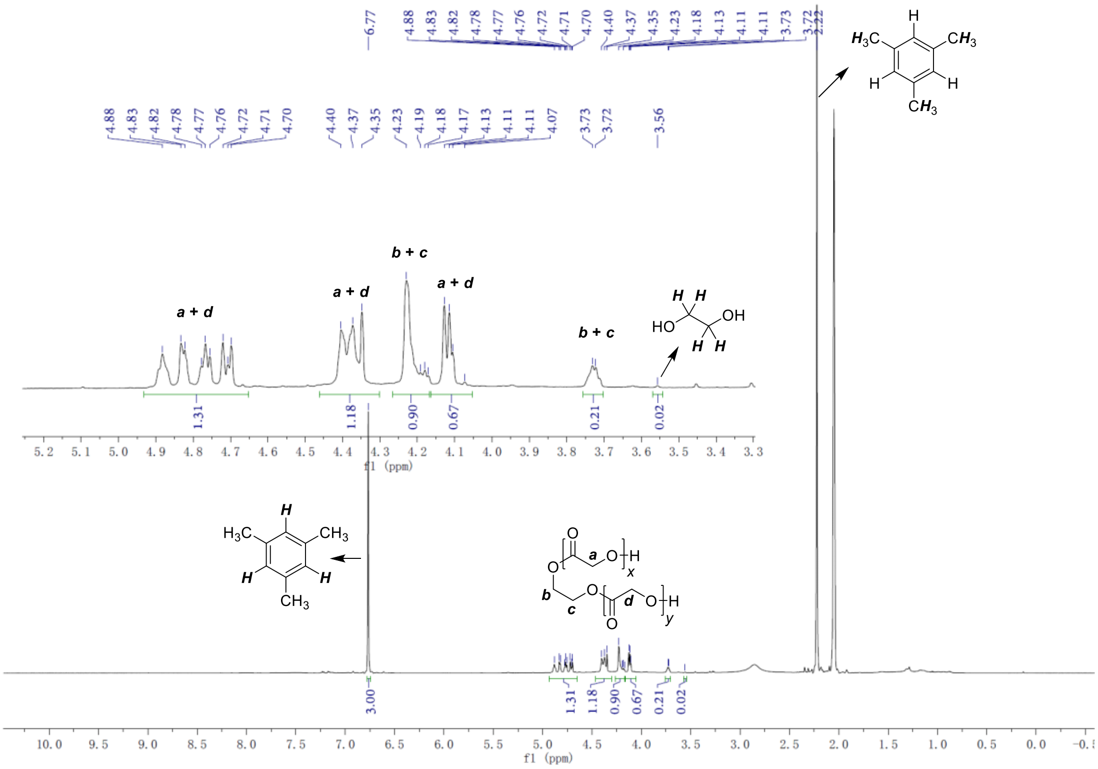

Figure S130. ^{1}H NMR (500 MHz, acetone-d_6) spectrum of reaction mixture of continuous experiments with **Ru-11** (1.0 mmol mesitylene as the internal standard)

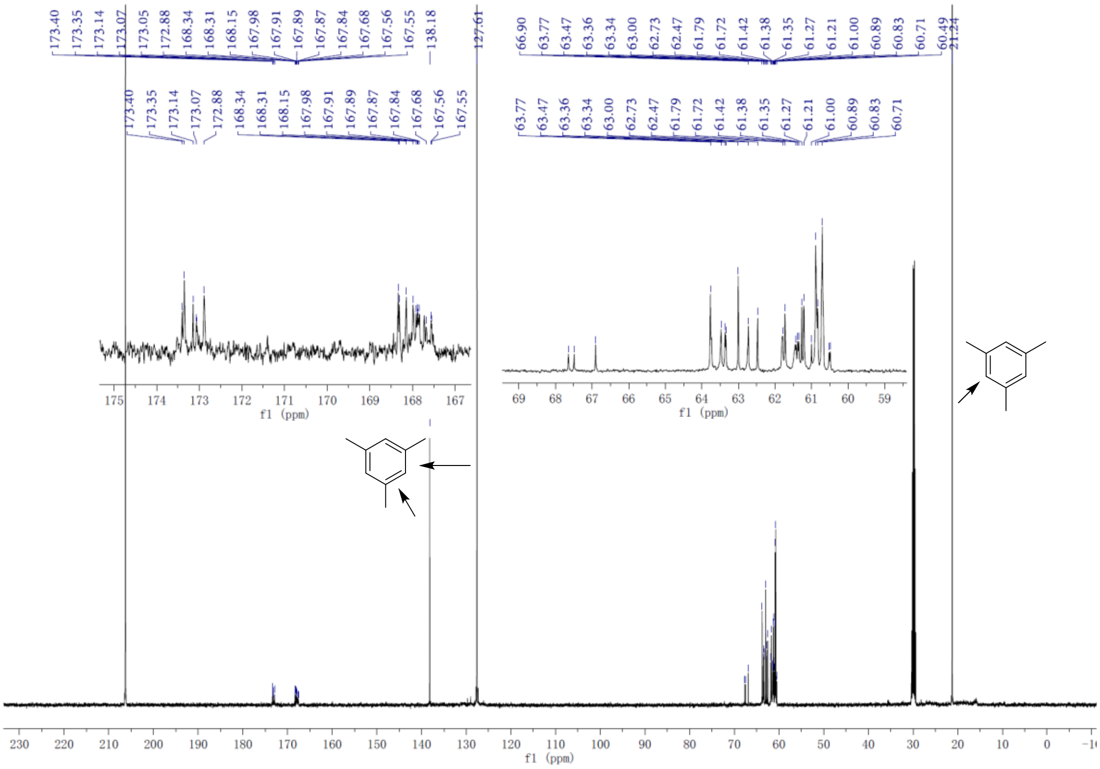

Figure S131. ^{13}C NMR (126 MHz, acetone-d_6) spectrum of reaction mixture of continuous experiments with **Ru-11** (1.0 mmol mesitylene as the internal standard)

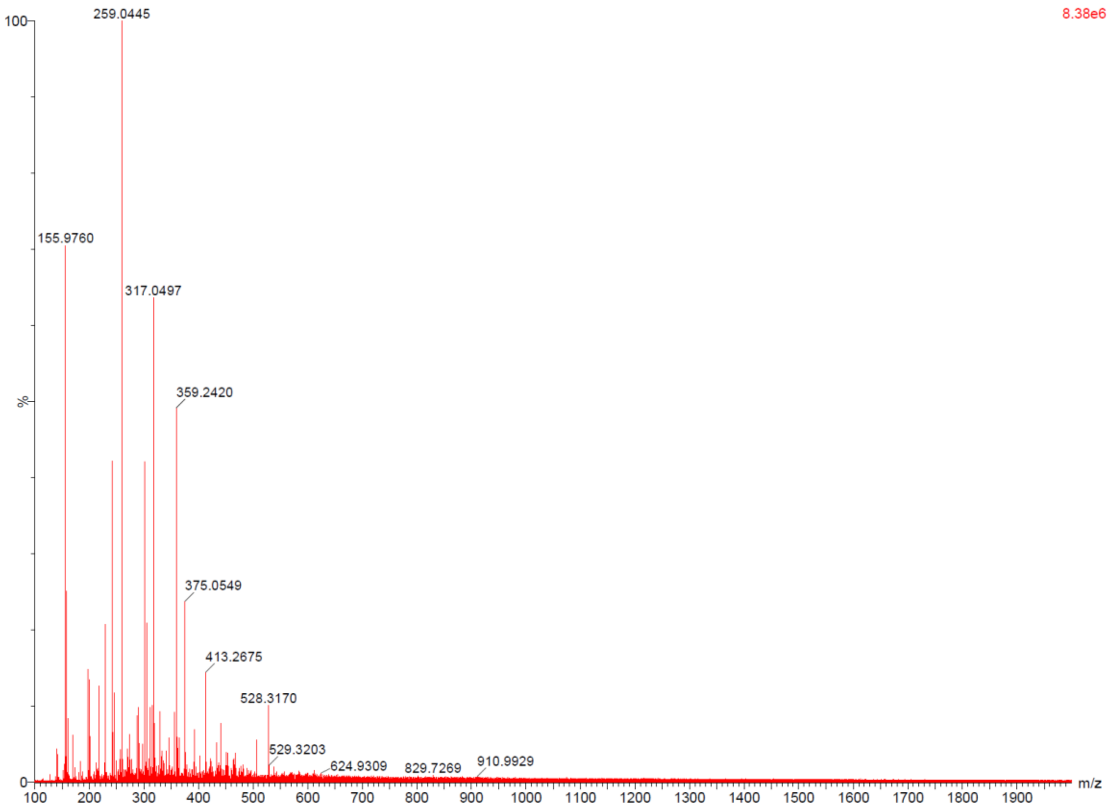

n	Calc. Mass $[M+Na]^+$	Mass $[M+Na]^+$
1	143.0315	Not detected
2	201.0370	201.0383
3	259.0424	259.0441
4	317.0479	317.0497
5	375.0534	375.0549
6	433.0589	433.0603

Figure S132. Mass spectrum of reaction mixture of continuous experiments with **Ru-11**

6. Base-free dehydrogenative coupling of HEG using a mixed solvent

General procedure: In a glovebox, 2-hydroxyethyl glycolate (HEG) (60.1 mg, 0.5 mmol) was added into a 100 mL Schlenk tube equipped with a magnetic stirring bar through a glass pipette. A 5 mL vial containing a magnetic stirring bar was charged with the ruthenium pincer complex (0.005 mmol) and dry and degassed dimethoxyethane (0.5 mL) and the solution was transferred into the above Schlenk tube using the same glass pipette. The vial was washed with toluene (2 * 0.25 mL) and the solution was transferred into the Schlenk tube. The Schlenk tube was taken out of the glovebox and stirred at 150 ˚C for 12 hours. Then the reaction mixture was firstly cooled to room temperature, and then the Schlenk tube was connected to the gas collecting system to measure the volume of gas. Finally, the solvent was removed under vacuum, mesitylene was added into Schlenk tube as an internal standard. The residue was dissolved in d_6-Acetone, and the resulting solution was passed through a short Celite column and then submitted to NMR analysis.

Table S8. Dehydrogenative coupling of HEG using a mixed solvent

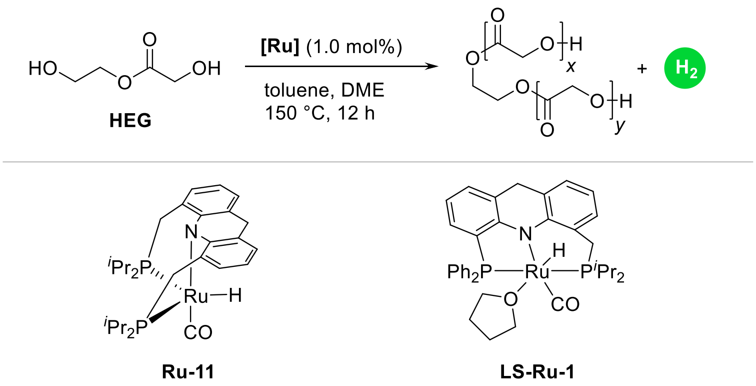

entry	[Ru]	Conv. (%)	V (H_2, mL)
1	**Ru-11**	62	4
2	**LS-Ru-1**	84	16

Reaction conditions: HEG (0.5 mmol), Ru catalyst (1.0 mol%), toluene (0.5 mL), 1,2-dimethoxyethane (0.5 mL) at 150 °C (bath temperature) for 12 hours. Conversions were determined by ^{1}H NMR spectroscopy from the reaction mixture using mesitylene (0.5 mmol) as an internal standard.

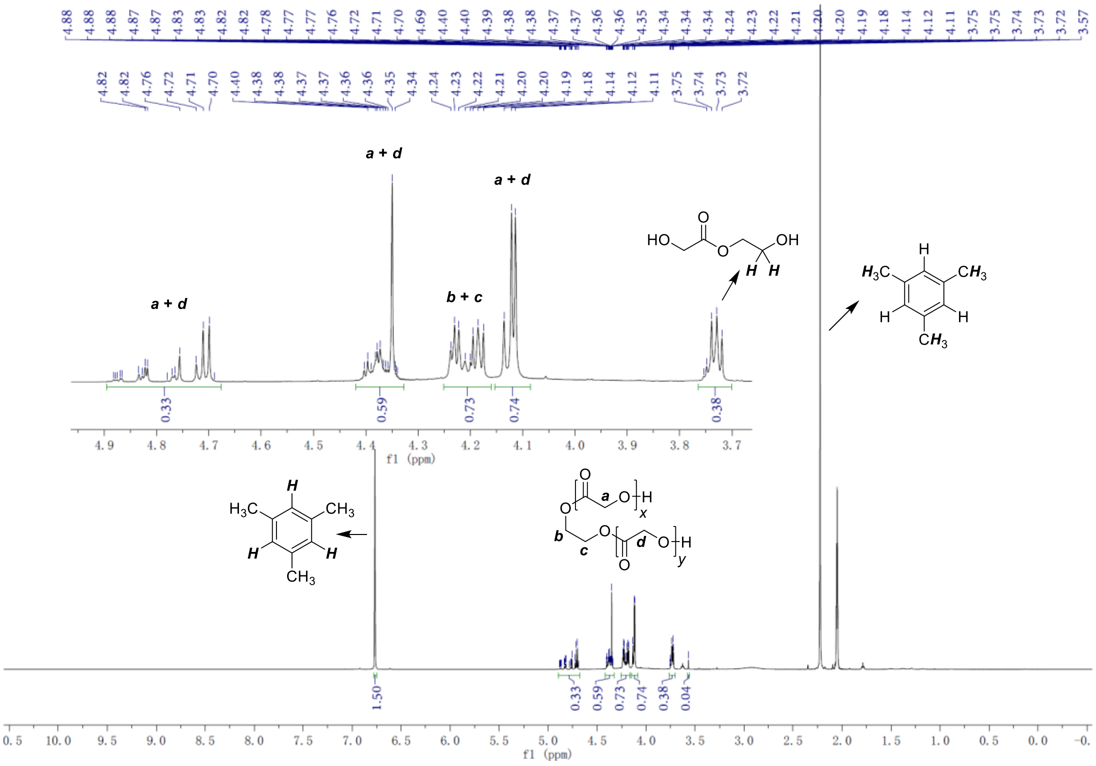

Figure S133. ^{1}H NMR (500 MHz, acetone-d_6) spectrum of reaction mixture of Table S8, entry 1 (0.5 mmol mesitylene as the internal standard)

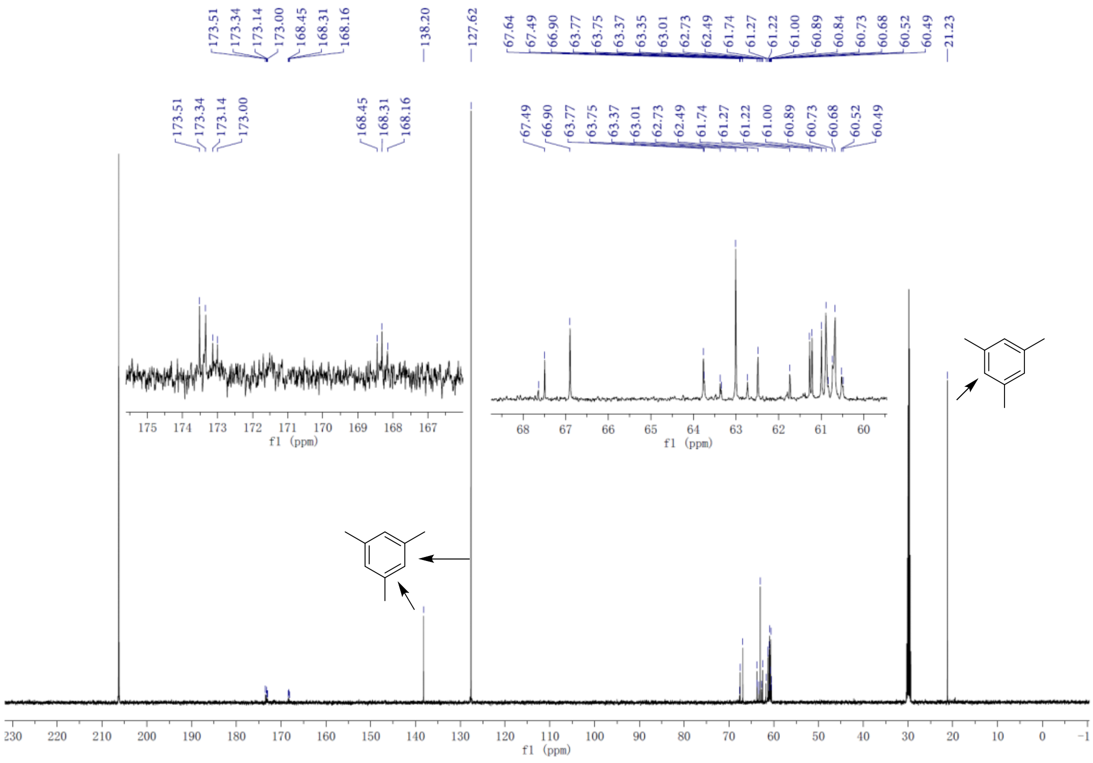

Figure S134. ^{13}C NMR (126 MHz, acetone-d_6) spectrum of reaction mixture of Table S8, entry 1 (0.5 mmol mesitylene as the internal standard)

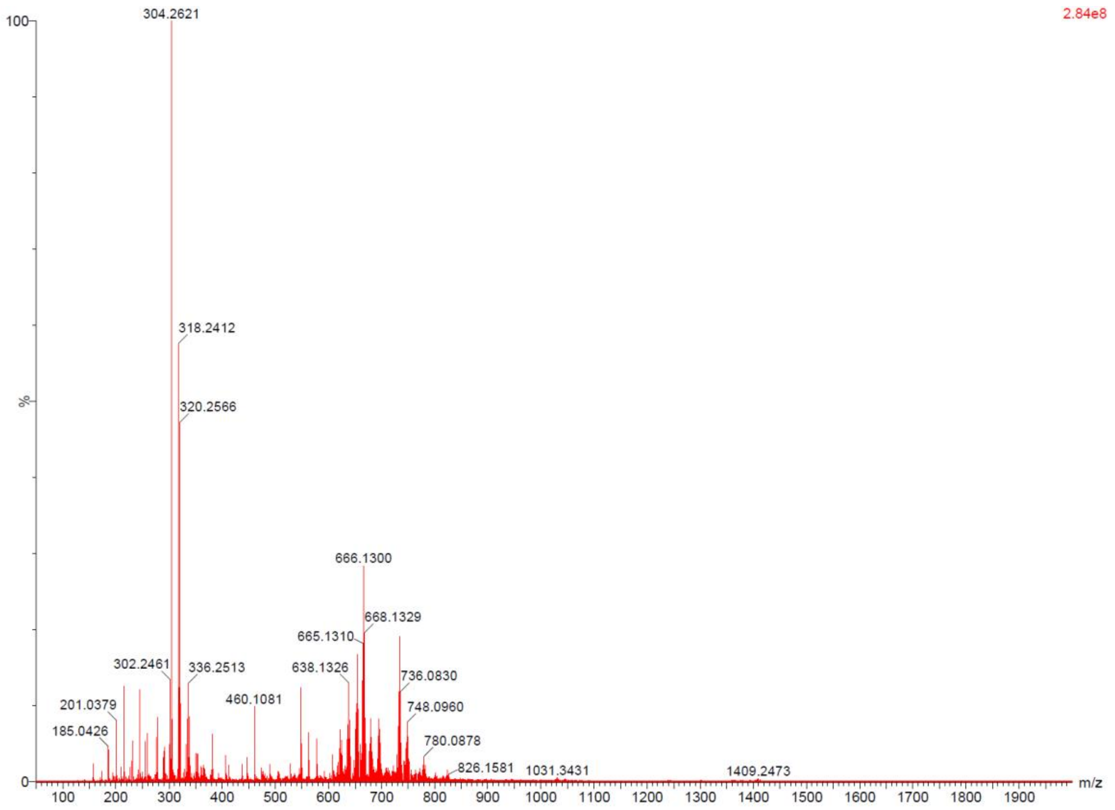

n	Calc. Mass $[M+Na]^+$	Mass $[M+Na]^+$
2	201.0370	201.0379

Figure S135. Mass spectrum of reaction mixture of Table S8, entry 1

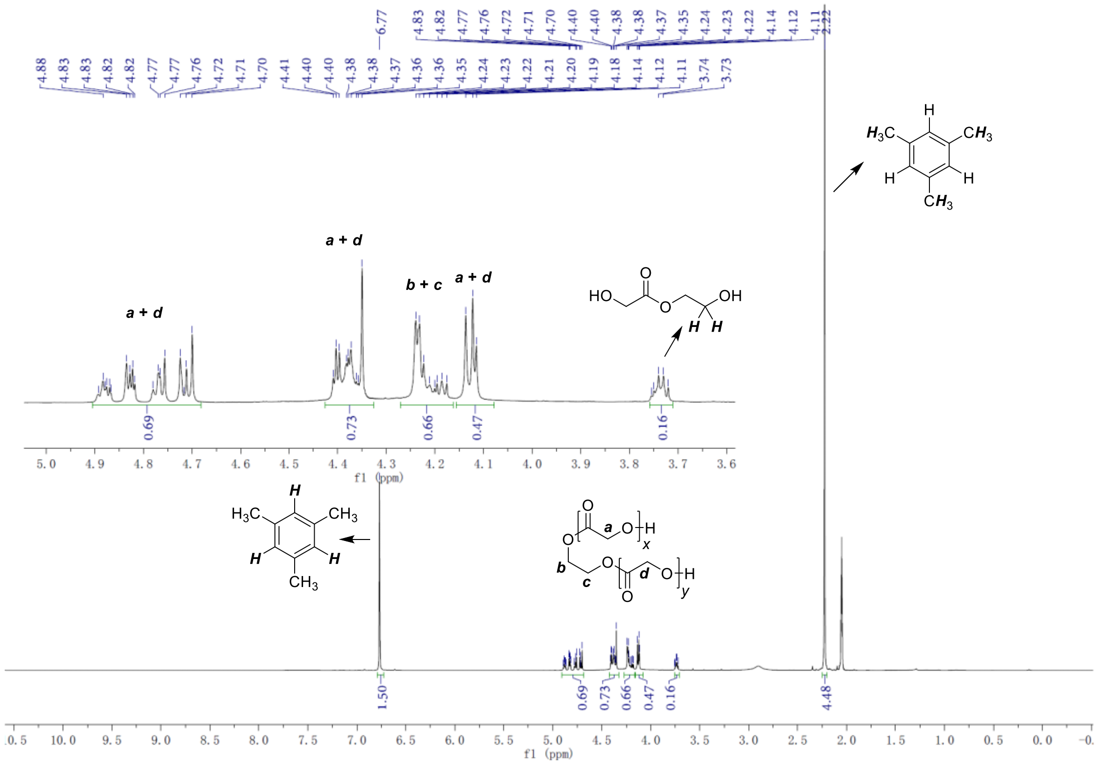

Figure S136. ^{1}H NMR (500 MHz, acetone-d_6) spectrum of reaction mixture of Table S8, entry 2 (0.5 mmol mesitylene as the internal standard)

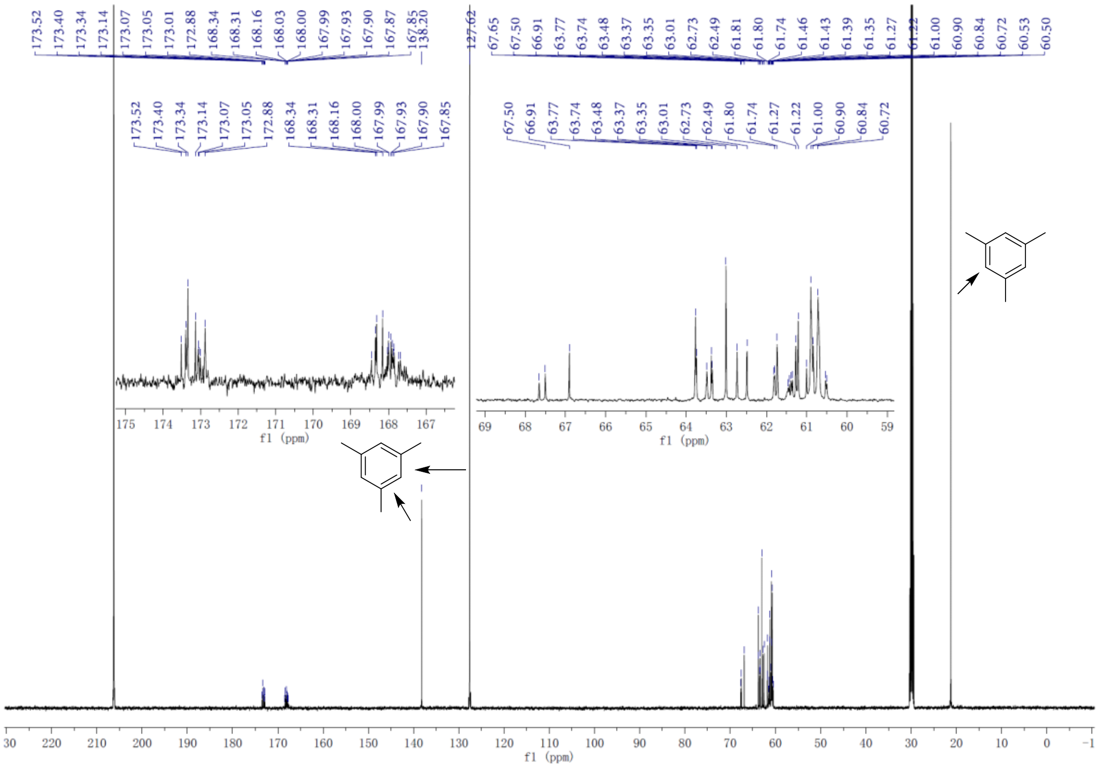

Figure S137. ^{13}C NMR (126 MHz, acetone-d_6) spectrum of reaction mixture of Table S8, entry 2 (0.5 mmol mesitylene as the internal standard)

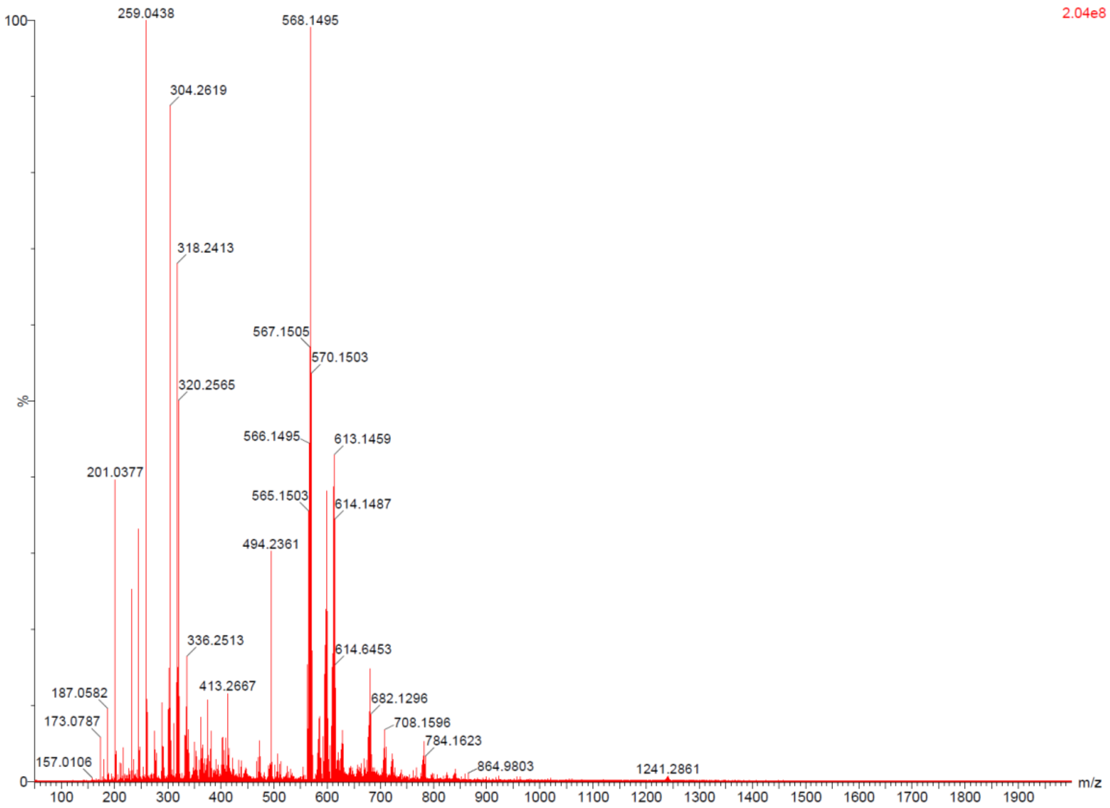

n	Calc. Mass $[M+Na]^+$	Mass $[M+Na]^+$
2	201.0370	201.0377
3	259.0424	259.0438

Figure S138. Mass spectrum of reaction mixture of Table S8, entry 2

7. Solvent- and additive-free dehydrogenation/hydrogenation cycle

Dehydrogenation: In a glovebox, ethylene glycol (1.0 mL, 17.8 mmol) and **LS-Ru-5** (0.036 mmol) were added into a 5.0 mL flask equipped with a magnetic stirring bar and a reflux condenser. The reaction flask was taken out of the glovebox under nitrogen protected conditions. Then the reaction system was connected to the vacuum pump through the top of the reflux condenser (connecting quickly). The resulting mixture was stirred at 150 °C for 168 hours under a pressure of 95 mbar. Then the reaction mixture was firstly cooled to room temperature, and mesitylene (270.4 mg, 2.25 mmol) was added as an internal standard. The residue was dissolved in acetone-d_6, and the resulting solution was passed through a short Celite column and then submitted to NMR analysis. ^{1}H NMR indicated that the conversion was 95%.

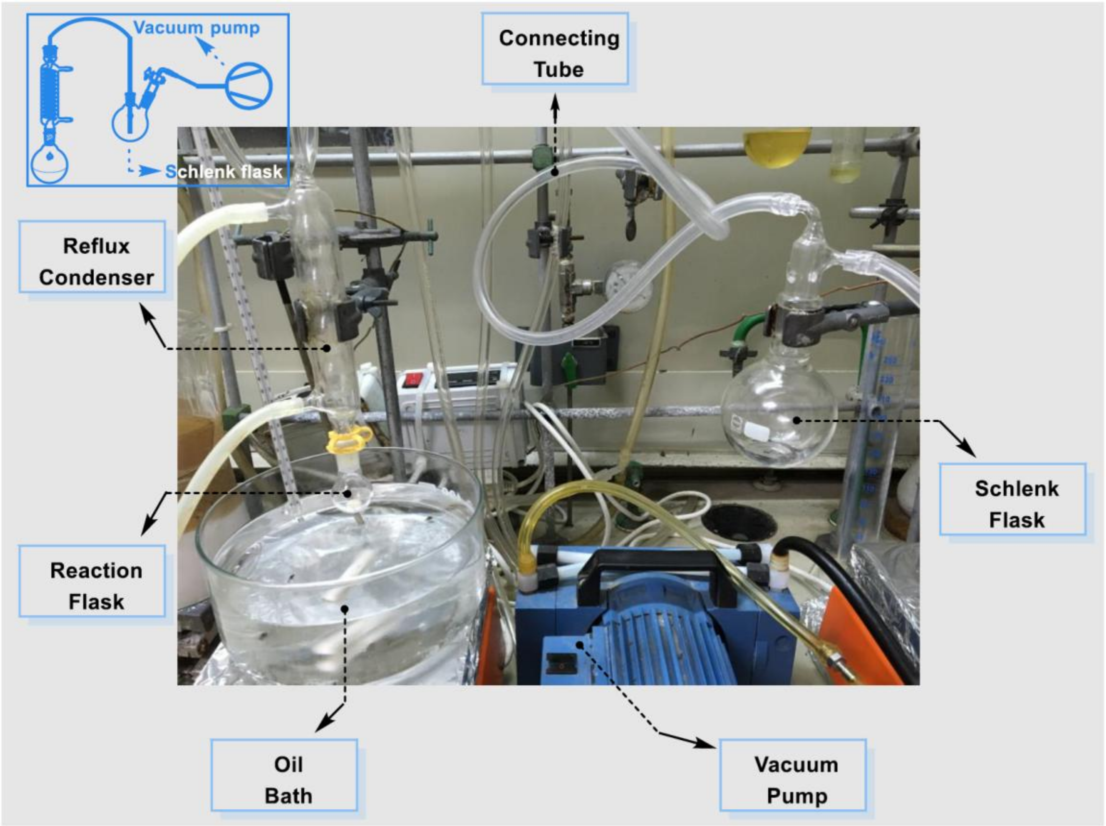

Figure S139. Schematic drawing and sideview of the reduced pressure reaction system.

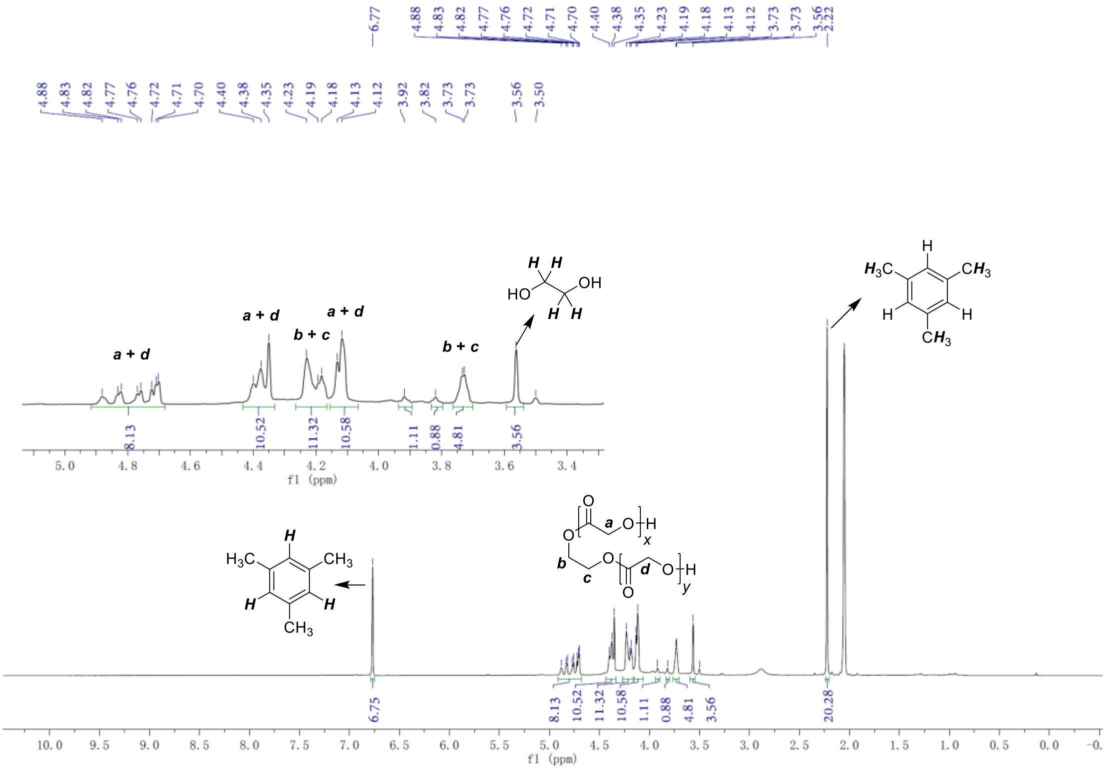

Figure S140. ^{1}H NMR (500 MHz, acetone-d_6) spectrum of reaction mixture of the dehydrogenative coupling of EG under partial vacuum

Conversion = (17.8 × 4 – 3.56)/(17.8 × 4) = 95%

$\mathbf{V_{H2}}$ = A_{a+d} × V_m = (8.13 + 10.52 + 10.58) × 24 mL = 701 mL (A: integral area; V_m: molar volume of hydrogen at 20 ˚C)

Yield (H_2) = 701 mL/(17.8 × 48 mL) = 82%

[17.8 × Conversion/(n + 1)] × 2n × V_m = V_{H2} → [17.8 × 95%/(n + 1)] × 2n × V_m = (8.13 + 10.52 + 10.58) × V_m → n = 6.37 (**n is the average degree of polymerizaiton**)

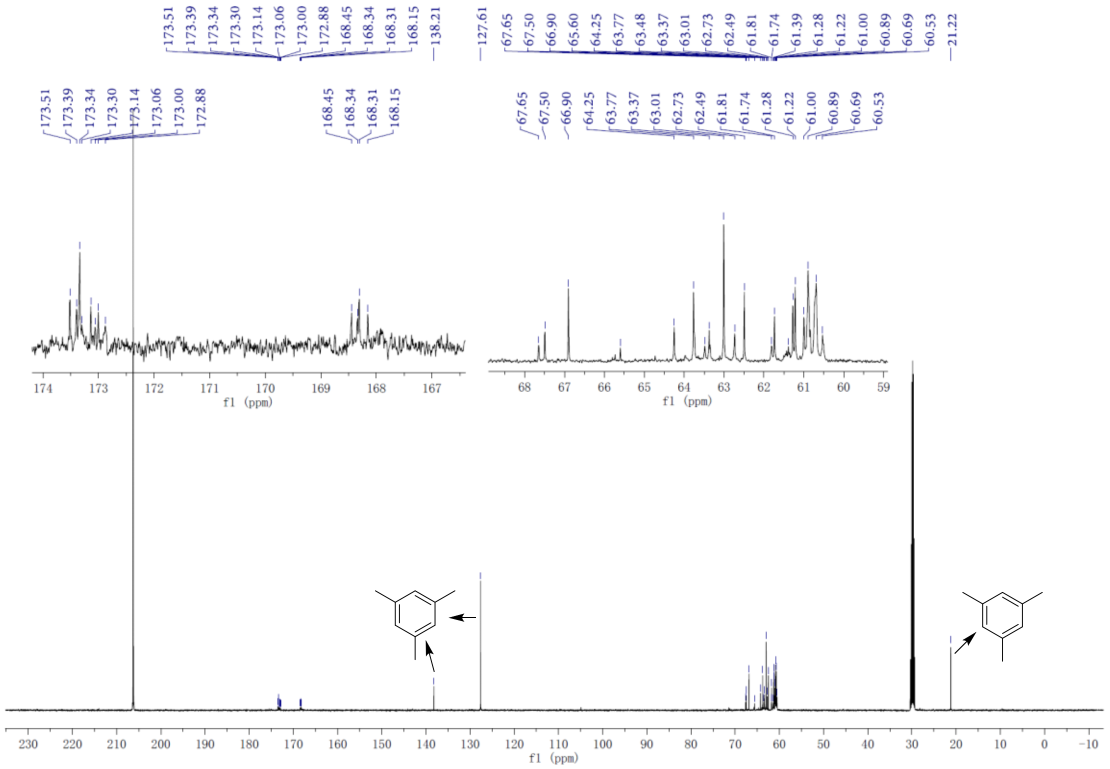

Figure S141. ^{13}C NMR (126 MHz, acetone-d_6) spectrum of reaction mixture of the dehydrogenative coupling of EG under partial vacuum

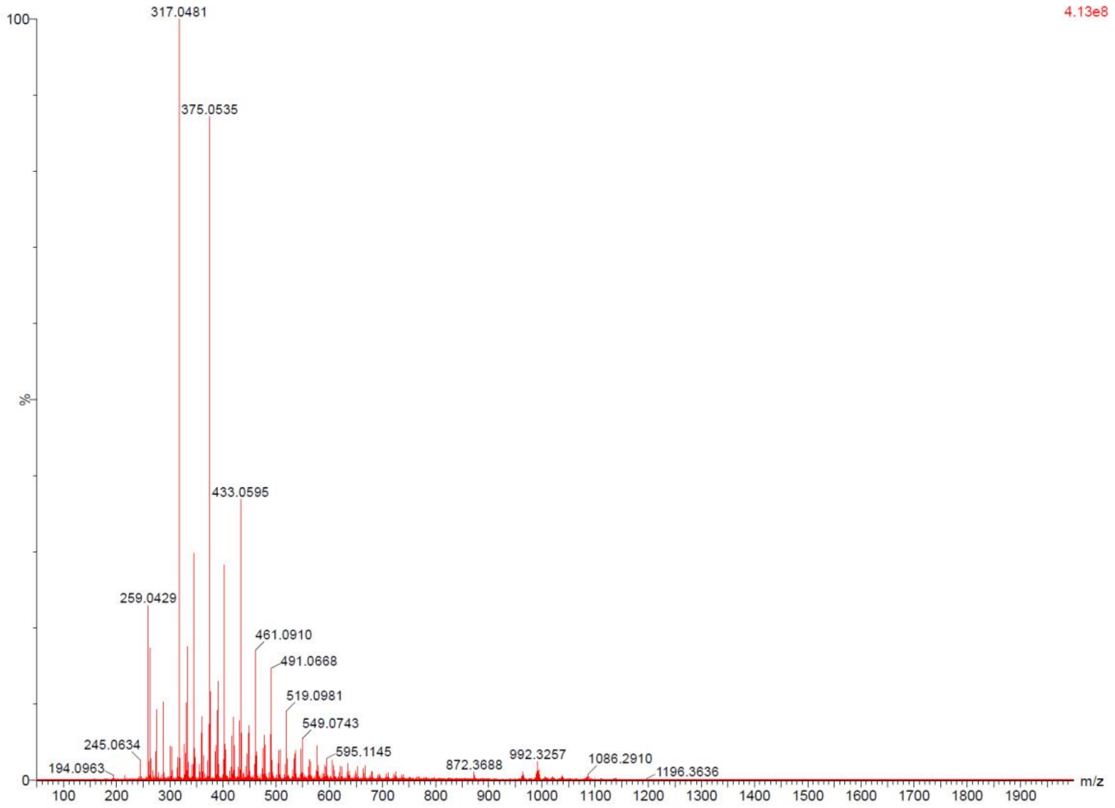

n	Calc. Mass [M+Na]$^+$	Mass [M+Na]$^+$
1	143.0315	Not detected
2	201.0370	Not detected
3	259.0424	259.0429
4	317.0479	317.0481
5	375.0534	375.0535
6	433.0589	433.0595
7	491.0644	491.0668
8	549.0698	549.0743

Figure S142. Mass spectrum of reaction mixture of the dehydrogenative coupling of EG under partial vacuum

Hydrogenation: In a glovebox, a 30 mL stainless steel autoclave with a Teflon tube containing a magnetic stirring bar was charged with the reaction mixture from the dehydrogenation reaction (from 17.8 mmol EG). The autoclave was taken out of the glovebox and purged five times with hydrogen and finally pressurized to 50 bar. The reaction mixture was stirred at 150 ˚C (oil bath temperature) for 24 hours, and then was cooled to room temperature in an ice bath. Then the reaction mixture was transferred into a 25 mL vial and the solvent was removed under vacuum and mesitylene (270.4 mg, 2.25 mmol) was added into Schlenk tube as an internal standard. The residue was dissolved in d_6-acetone, and the resulting solution was passed through a short Celite column and then submitted to NMR analysis. ^{1}H NMR indicated that the conversion was >99% and the yield of ethylene glycol was 92%.

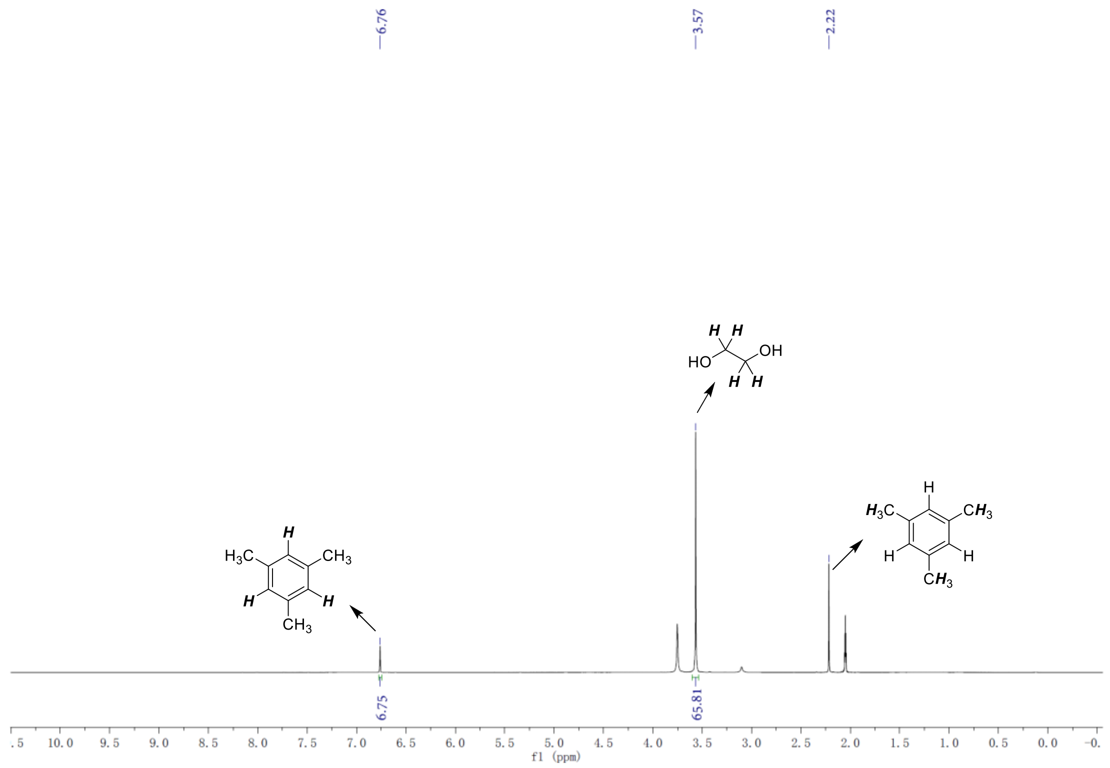

Figure 143. ^{1}H NMR (500 MHz, acetone-d_6) spectrum of the hydrogenation mixture.

Analysis of the purity of H_2 under partial vacumm: A large-scale reaction (17.8 mmol EG) was set under partial vacuum (95 mbar). After connecting to the vacuum, the reaction system was closed, and the mixture was stirred at 150 ˚C under the static partial vacuum. After 5 hours, the gas phase was analyzed by GC, showing that only hydrogen gas was released (purity of hydrogen: 100%). Because of the increasing volume of hydrogen, it is difficult to keep the vacuum of the reaction system as 95 mbar, therefore we stopped the reaction. This result indicates that almost no CO was formed in the large-scale reaction under partial vacuum. Please see the results below for detail (Figure S149).

8. GC Data

Preparation of standard curve by GC for H_2 and CO

GC conditions: HP 6890 Series GC System; column: SUPELCO 1-2382, 5Ft×1/8In S.S. SUPPORT 45/60 CARBOXENTM 1000, Packed Column. Inlets: 87 ˚C; Detector: TCD 250 ˚C; Carrier Gas: He; Flow: 29.1 mL/min; Oven: 35 ˚C, hold 2 min; 10 ˚C /min to 60 ˚C, hold 0 min; 30 ˚C /min to 200 ˚C.

	CO (μL)	H_2 (μL)	CO (peak area)	H_2 (peak area)
Mixture 1	20	100	2478	365
Mixture 2	20	200	3462	1074
Mixture 3	20	300	3599	1877
Mixture 4	20	400	3517	2810
Mixture 5	20	500	3701	3821

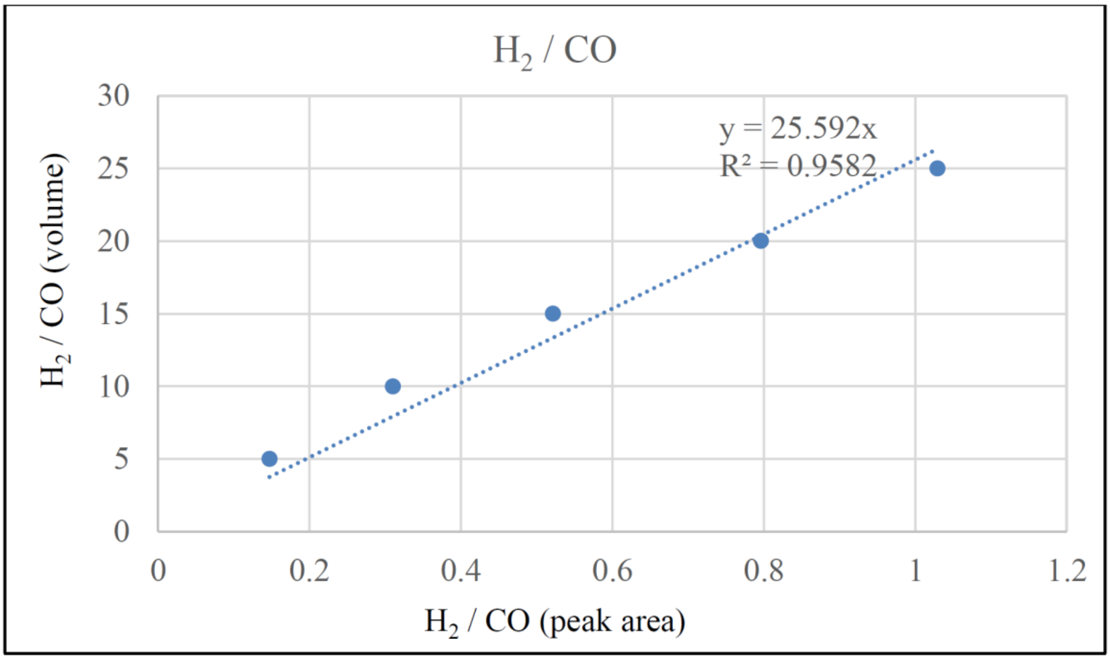

$V_{H2}/V_{CO} = A_{H2}/A_{CO} \times 25.592$ (V: volume; A: peak area)

H_2 (%) = V_{H2}/V_{H2+CO}

CO (%) = 1 – H_2 (%)

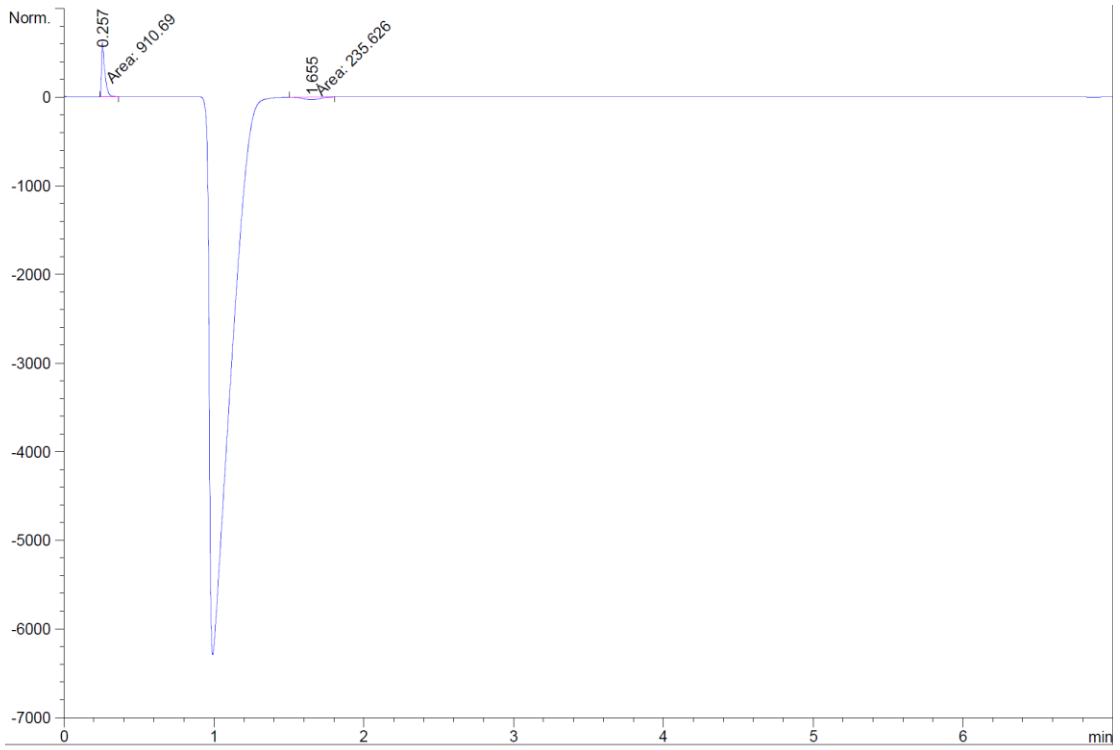

Peak #	RetTime [min]	Type	Width [min]	Area [25 uV*s]	Height [25 uV]	Area %
1	0.257	MM	0.0258	910.68951	588.26471	79.44492
2	1.655	MM N	0.1384	235.62613	28.38511	20.55508

Figure S144. GC analysis (gas phase) of the reaction mixture of Table S6, entry 1. (t_1 = 0.257 min [H_2, 99.00%], t_2 = 1.655 min [CO, 1.00%])

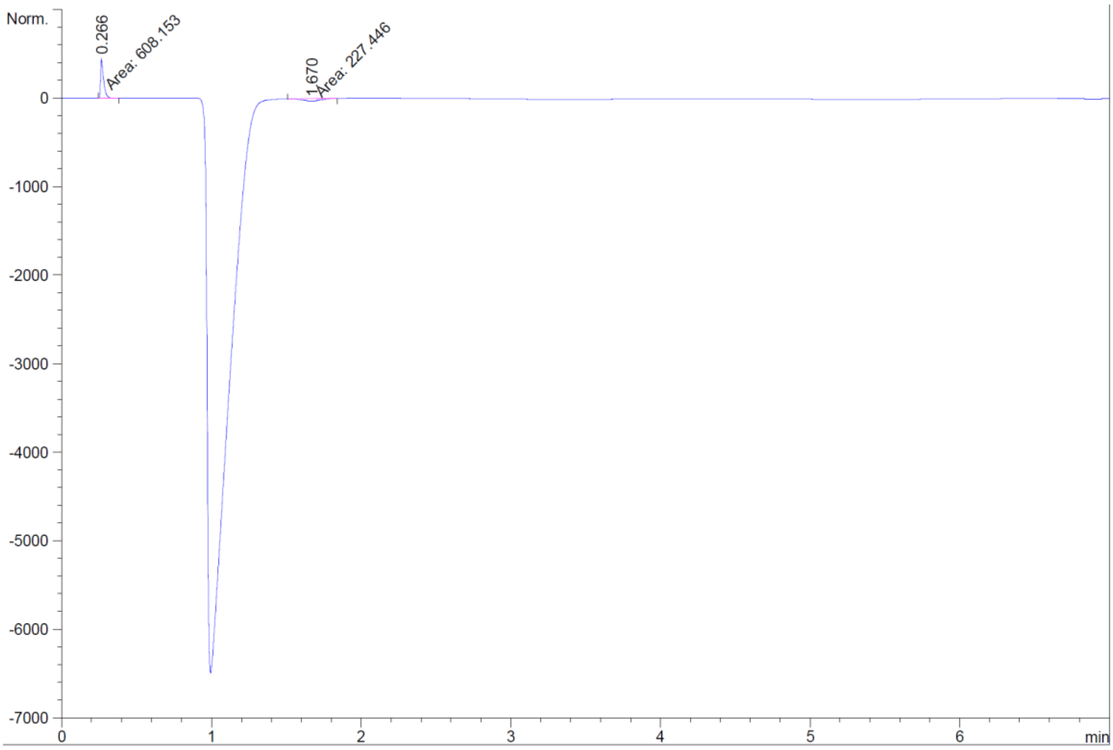

Peak #	RetTime [min]	Type	Width [min]	Area [25 uV*s]	Height [25 uV]	Area %
1	0.266	MM	0.0225	608.15326	451.14551	72.78045
2	1.670	MM N	0.1396	227.44644	27.15590	27.21955

Figure S145. GC analysis (gas phase) of the reaction mixture of Table S6, entry 2. (t_1 = 0.266 min [H_2, 98.56%], t_2 = 1.670 min [CO, 1.44%])

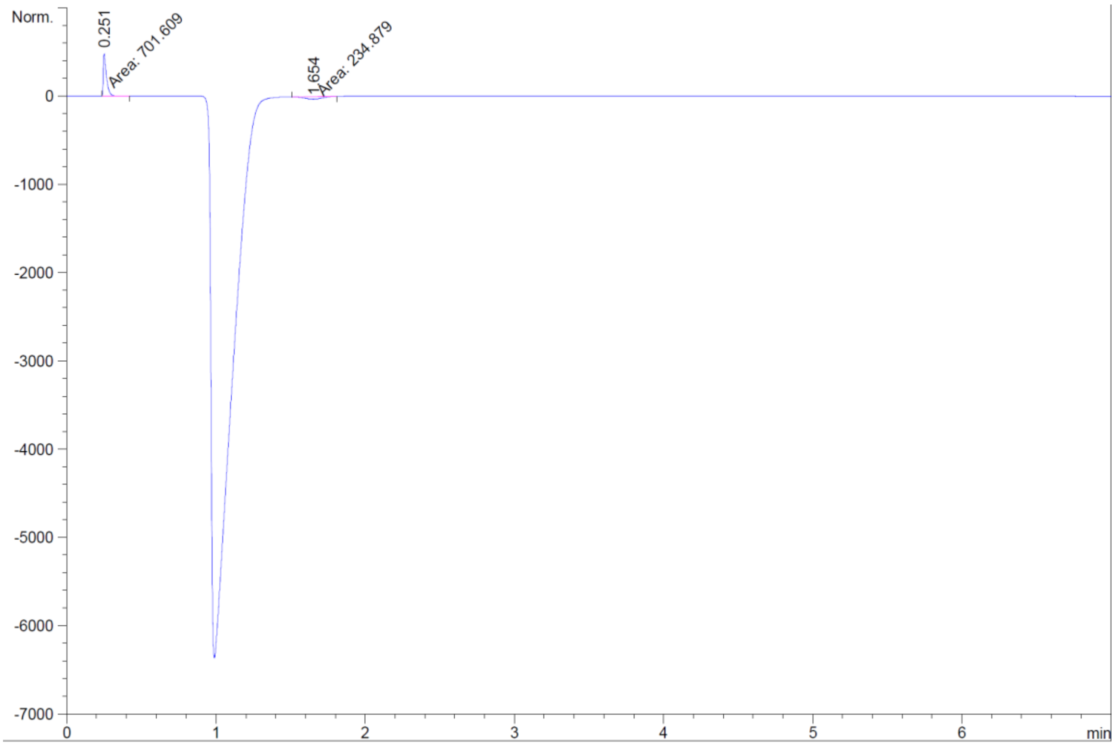

Peak #	RetTime [min]	Type	Width [min]	Area [25 uV*s]	Height [25 uV]	Area %
1	0.251	MM	0.0236	701.60895	494.87042	74.91917
2	1.654	MM N	0.1368	234.87894	28.61708	25.08083

Figure S146. GC analysis (gas phase) of the reaction mixture of Table S6, entry 3. (t_1 = 0.251 min [H_2, 98.71%], t_2 = 1.654 min [CO, 1.29%])

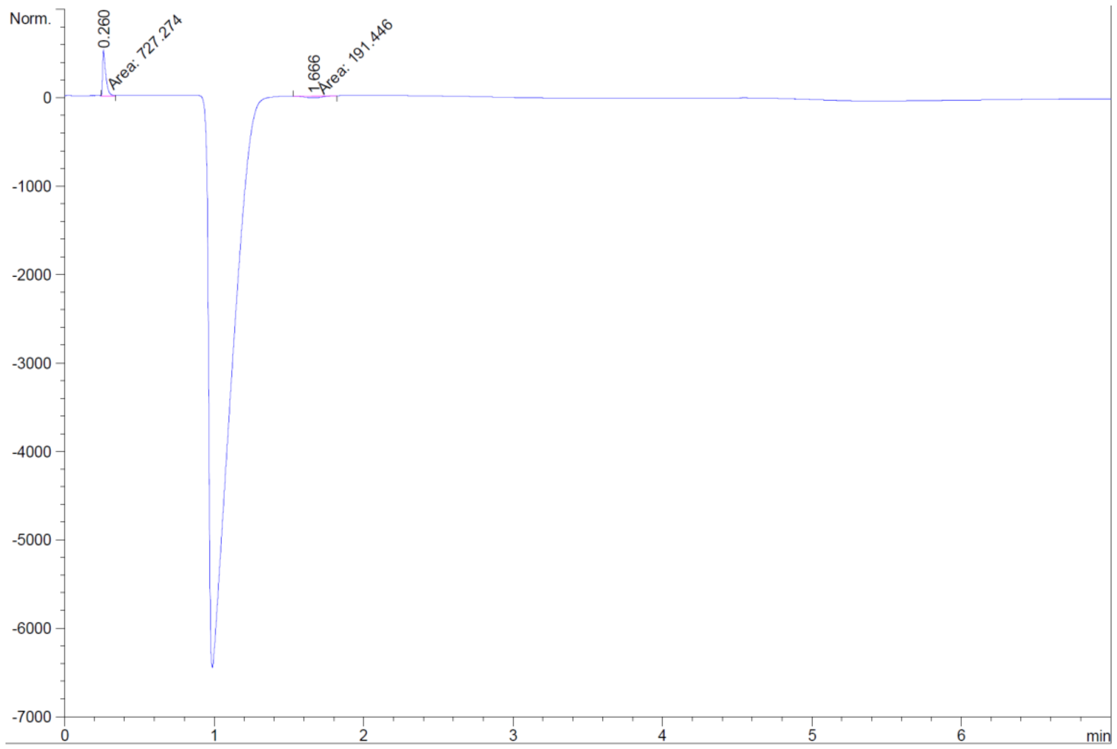

Peak #	RetTime [min]	Type	Width [min]	Area [25 uV*s]	Height [25 uV]	Area %
1	0.260	MM	0.0236	727.27435	512.55640	79.16167
2	1.666	MM N	0.1396	191.44594	22.86376	20.83833

Figure S147. GC analysis (gas phase) of the reaction mixture of Table S6, entry 4. (t_1 = 0.260 min [H_2, 98.98%], t_2 = 1.666 min [CO, 1.02%])

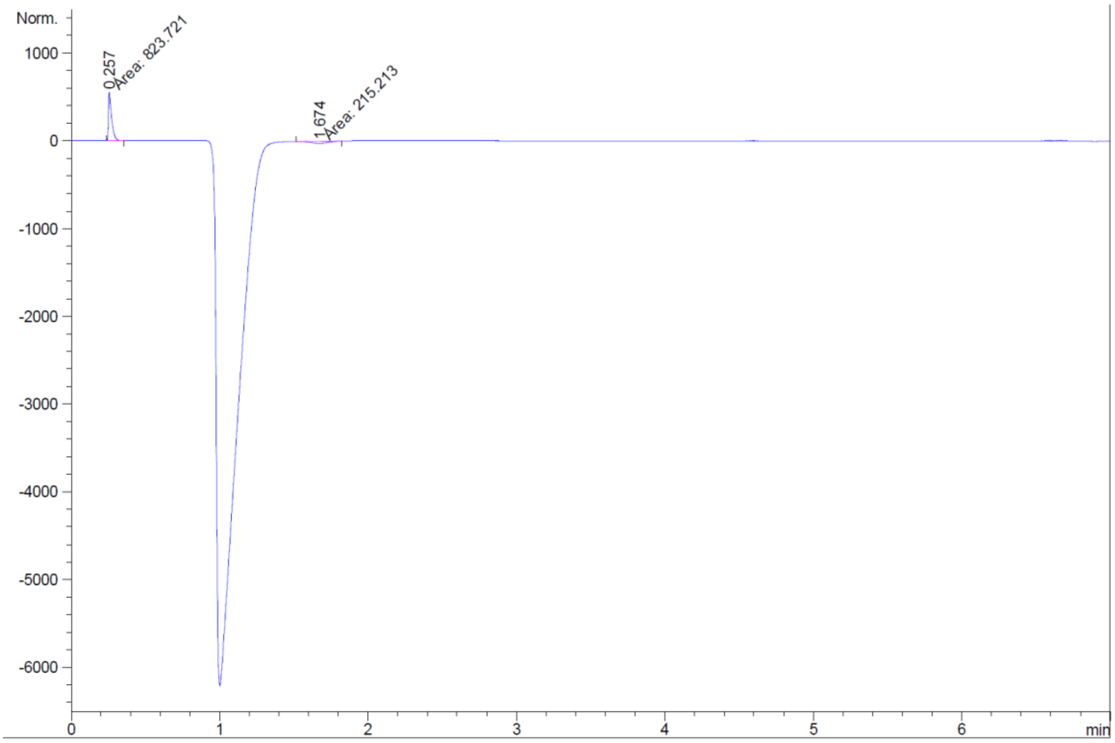

Peak #	RetTime [min]	Type	Width [min]	Area [25 uV*s]	Height [25 uV]	Area %
1	0.257	MM	0.0254	823.72125	540.48804	79.28518
2	1.674	MM N	0.1394	215.21346	25.72209	20.71482

Figure S148. GC analysis (gas phase) of the reaction mixture of Table S6, entry 5. (t_1 = 0.257 min [H_2, 98.99%], t_2 = 1.674 min [CO, 1.01%])

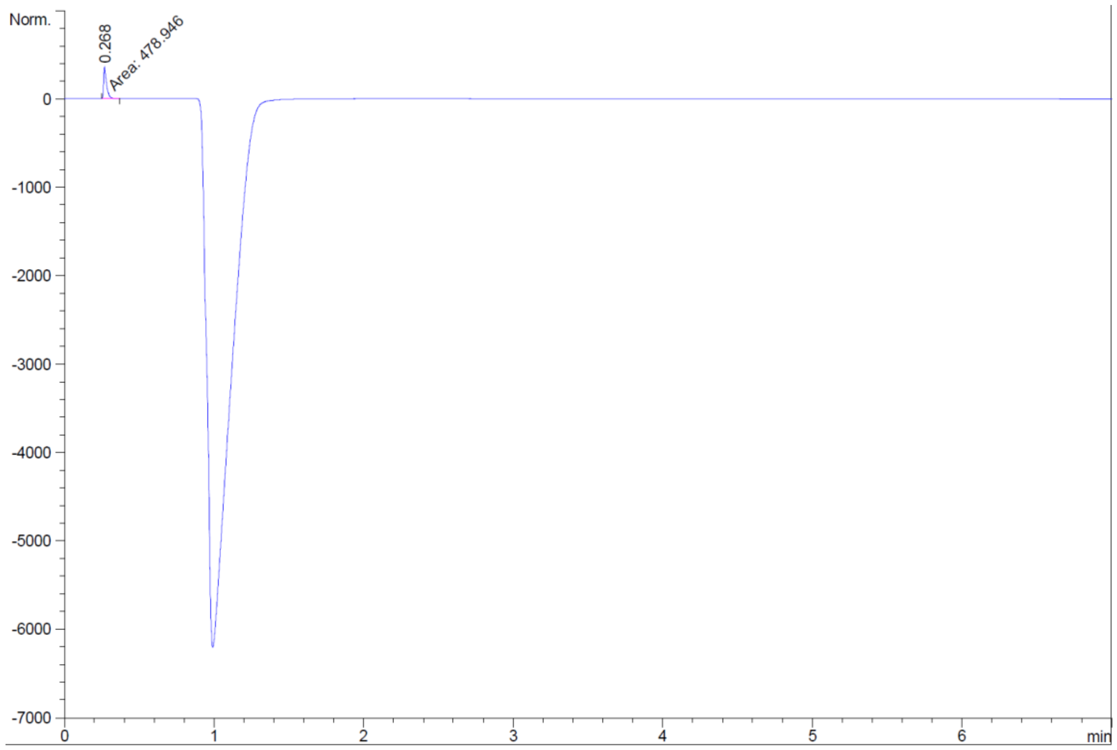

Peak #	RetTime [min]	Type	Width [min]	Area [25 uV*s]	Height [25 uV]	Area %
1	0.268	MM	0.0228	478.94553	349.79675	1.000e2

Figure S149. GC analysis (gas phase) of the purity of H_2 under partial vacuum. (t_1 = 0.268 min [H_2, 100% purity])

9. Computational studies

Density functional theory (DFT) calculations were performed with Gaussian 16 (C.01 revision),[12] using Truhlar's M06-L functional,[13] the triple-ξ def2-TZVP basis set,[14] W06 density fitting,[15] and Grimme's D3(0) empirical dispersion correction.[16] Frequency calculations at this level of theory were run at 423.15K to confirm stationary points and transition states, and to obtain thermodynamic corrections. Single point energies of the M06-L optimized structures were computed with ORCA (5.0.3),[17] using the range-separated meta-GGA hybrid functional ωB97M-V of the Head-Gordon group,[18] including dispersion correction,[19,20] together with the triple-ξ def2-TZVPP basis set,[14] and the corresponding auxiliary basis sets, def2/J[15] and def2-TZVPP/C,[21] for RIJCOSX density fitting. The functional and basis set selections are based on recent benchmark studies.[22]

Gibbs free energies at 423.15 K were computed by adding the free energy correction term from the frequency calculation to the single point energy in the gas phase, according to the following equation:

$$\mathbf{G^{o\omega B97M\text{-}V}_{(gas,\ 423.15K)} = E^{\omega B97M\text{-}V}_{gas} + corr^{\mathit{M06\text{-}L}}_{\mathit{freq}(gas,\ 423.15K)}}$$

where $\mathbf{E^{\omega B97M\text{-}V}_{gas}}$ is the single point energy, and $\mathbf{corr^{\mathit{M06\text{-}L}}_{\mathit{freq}}}$ is the thermal correction to the Gibbs free energy from the frequency calculation (at T = 423.15 K).

Free energy values (G^o) were corrected to account for changes in standard states ($G^o \rightarrow G$).

Standard state corrections[23] were employed, such that all species are treated as 1 M (using an ideal gas approximation), with the exception of H_2 (maintained as 1 atm).[24-36]

Table S9. Energy data for calculated complexes, transition states, substrates and products.

Structure	$E^{\omega B97M\text{-}V}_{gas}$	$G^{o\omega B97M\text{-}V}_{(gas,\ 423.15K)}$	Imaginary Frequency	G T = 423.15K
Energy Unit	Hartree	Hartree	cm^{-1}	kcal
**mer-Ru-L-^{i}Pr-1**	-1999.27955	-1998.763765	-	-1254221.573
**fac-Ru-L -^{i}Pr-1**	-1999.267493	-1998.746937	-	-1254211.013
**Ru-L-iPr-2-EG**	-2229.570557	-2228.976329	-	-1398679.957
**Ru-L-iPr-3-EG-H2**	-2228.393288	-2227.809278	-	-1397947.632
**Ru-L-iPr-4-CHO**	-2228.365271	-2227.792123	-	-1397936.867
**Ru-L-iPr-5-acetal**	-2457.487835	-2456.846926	-	-1541668.756
**mer-Ru-LS -Ph-1**	-2418.707069	-2418.124148	-	-1517370.213
**fac-Ru-LS -Ph-1**	-2418.700503	-2418.113898	-	-1517363.782
**Ru-LS-Ph-2-EG**	-2416.531678	-2415.979629	-	-1516024.527
**Ru-LS-Ph-3-EG-H2**	-2415.358504	-2414.815268	-	-1515293.891
**Ru-LS-Ph-4-CHO**	-2415.332941	-2414.799628	-	-1515284.077
**Ru-LS-Ph-5-acetal**	-2644.453786	-2643.856416	-	-1659017.211
**L-iPr-TS2,3**	-2229.527476	-2228.935607	-1031.3960	-1398654.404
**L-iPr-TS3,4**	-2228.360797	-2227.785298	-455.5671	-1397932.585

**L-iPr-TS4,5**	-2458.628937	-2457.978315	-1014.0270	-1542378.703
**L-iPr-TS5,1**	-2457.449314	-2456.817019	-551.8908	-1541649.99
**LS-Ph-TS2,3**	-2416.495249	-2415.94721	-916.6701	-1516004.184
**LS-Ph-TS3,4**	-2415.329501	-2414.796865	-407.7009	-1515282.343
**LS-Ph-TS4,5**	-2645.59573	-2644.988203	-1019.5815	-1659727.408
**LS-Ph-TS5,1**	-2644.428646	-2643.838046	-598.3833	-1659005.684
**H_2**	-1.161799848	-1.169859848	-	-734.0870549
**EG**	-230.2717914	-230.2296574	-	-144466.4203
**HEG**	-458.1881543	-458.1152983	-	-287464.66
**THF**	-232.4377317	-232.3638087	-	-145805.6002
**Glycolaldehyde**	-229.069651	-229.051137		-143726.8987

Cartesian coordinates for calculated structures

mer-Ru-L-^{i}Pr-1

Ru	20.1854270000	0.3205730000	6.2061230000
P	18.4223550000	-1.1469820000	5.8361110000
P	21.6619760000	2.0630070000	5.7798150000
O	22.2814700000	-1.7921250000	6.3615320000
N	18.8401270000	1.6956900000	7.1749330000
C	21.4640000000	-0.9690480000	6.2511670000
C	18.9021760000	-3.3027180000	7.5635800000
C	16.5216940000	-3.0410260000	6.7937220000
C	17.8178660000	-2.3242190000	7.1360930000
C	18.2171560000	-1.2676920000	3.0420060000
C	19.8107180000	-2.8683620000	4.1043150000
C	18.4870470000	-2.1333600000	4.2646790000
C	23.5940820000	3.9580360000	6.6712060000
C	23.8373610000	1.5904220000	7.4857680000

C	22.8663730000	2.6780710000	7.0497100000
C	21.7326630000	2.2159500000	2.9809320000
C	23.3454380000	0.6345370000	4.0432390000
C	22.6193120000	1.9642950000	4.1924120000
C	17.5938190000	1.2920760000	7.7029420000
C	19.2638540000	2.9471410000	7.6741780000
C	17.0083510000	0.0049400000	5.6029750000
C	16.6547280000	0.5670790000	6.9376530000
C	15.4055050000	0.2754710000	7.4766530000
C	15.0510450000	0.6573720000	8.7582060000
C	15.9862510000	1.3235550000	9.5315530000
C	17.2383310000	1.6259750000	9.0233940000
C	18.2896200000	2.2975730000	9.8384480000
C	18.9563550000	3.3284990000	8.9938500000
C	19.2792850000	4.5863130000	9.4745680000
C	19.9404340000	5.5015140000	8.6734090000
C	20.2965970000	5.1212980000	7.3917480000
C	19.9838410000	3.8658520000	6.8800010000
C	20.5192270000	3.4836250000	5.5423310000
H	19.0811340000	-4.0608180000	6.8003130000
H	19.8491380000	-2.8047210000	7.7689150000
H	18.5995060000	-3.8304290000	8.4684710000
H	16.1486660000	-3.5811900000	7.6649520000
H	15.7359150000	-2.3556550000	6.4793920000
H	16.6652030000	-3.7774060000	6.0019930000
H	17.6234000000	-1.6456810000	7.9741520000
H	18.3137160000	-1.8647710000	2.1345150000
H	17.2201030000	-0.8316240000	3.0434060000
H	18.9400710000	-0.4526340000	2.9724160000

H	20.6176950000	-2.1617840000	3.9091930000
H	20.0941580000	-3.4445140000	4.9828350000
H	19.7601580000	-3.5541510000	3.2576330000
H	17.6794480000	-2.8677160000	4.3503200000
H	24.3190860000	3.7887960000	5.8740710000
H	22.9143830000	4.7452700000	6.3481350000
H	24.1491020000	4.3430330000	7.5277640000
H	24.3822570000	1.9054170000	8.3761640000
H	23.3305060000	0.6546230000	7.7186730000
H	24.5817230000	1.3852970000	6.7156280000
H	22.2024290000	2.8978580000	7.8931680000
H	20.9130930000	1.4959810000	2.9395310000
H	21.3013560000	3.2150350000	2.9718980000
H	22.3126250000	2.0998250000	2.0646770000
H	24.0131180000	0.6650200000	3.1812610000
H	23.9394430000	0.3666810000	4.9147000000
H	22.6318590000	-0.1732390000	3.8796380000
H	23.3586140000	2.7698500000	4.2497100000
H	16.1445470000	-0.4851730000	5.1489530000
H	17.3679740000	0.7701760000	4.9094630000
H	14.6954520000	-0.2636560000	6.8591290000
H	14.0687360000	0.4284430000	9.1486330000
H	15.7524140000	1.6117340000	10.5501930000
H	19.0362000000	1.5495590000	10.1456470000
H	17.8819980000	2.7247670000	10.7546300000
H	19.0112590000	4.8406240000	10.4938050000
H	20.1853000000	6.4881630000	9.0426500000
H	20.8316740000	5.8151920000	6.7527130000
H	19.7386990000	3.1193850000	4.8685530000

H	21.0093280000	4.3346370000	5.0647090000
H	20.3168760000	0.1602830000	4.6396530000

fac-Ru-L-^{i}Pr-1

H	0.6823480000	8.4543010000	5.3496700000
P	-2.3598460000	7.7507270000	2.7944700000
P	0.5065780000	6.1063250000	4.9673900000
O	-2.3519490000	7.7758030000	6.8440220000
N	0.7115610000	8.3745030000	2.7589260000
C	-1.5825870000	7.8657620000	1.1322450000
H	-0.9223780000	6.9964810000	1.0644320000
H	-2.3321750000	7.7836640000	0.3418470000
C	-0.7884730000	9.1193160000	0.9897560000
C	-1.1395540000	10.0773840000	0.0452890000
H	-2.0199810000	9.9062860000	-0.5655440000
C	-0.3656550000	11.2084260000	-0.1553380000
H	-0.6431700000	11.9356810000	-0.9063180000
C	0.7791450000	11.3850340000	0.6088190000
H	1.4044490000	12.2580610000	0.4572010000
C	1.1333640000	10.4702500000	1.5862720000
C	0.3458170000	9.3241540000	1.8023990000
C	2.3238390000	10.6540550000	2.4757510000
H	3.0434950000	11.3481430000	2.0389780000
H	1.9928580000	11.1166580000	3.4182540000
C	2.9542770000	9.3348940000	2.7967310000
C	4.3156190000	9.1687370000	2.9902660000
H	4.9761080000	10.0156440000	2.8394580000
C	4.8324860000	7.9480130000	3.4006300000
H	5.8957410000	7.8271750000	3.5582240000

C	3.9695530000	6.8830720000	3.6072080000
H	4.3625740000	5.9209670000	3.9190350000
C	2.6024480000	7.0105210000	3.3910250000
C	2.0790660000	8.2534580000	2.9837440000
C	1.6884770000	5.8438000000	3.5811450000
H	2.2628740000	4.9316170000	3.7506460000
H	1.0610530000	5.6892570000	2.6970460000
C	-3.4755640000	6.2758560000	2.6410160000
H	-4.2903940000	6.5760200000	1.9760820000
C	-4.0622460000	5.9045650000	3.9942850000
H	-3.2795790000	5.6514030000	4.7106980000
H	-4.7264960000	5.0440280000	3.9040280000
H	-4.6371770000	6.7206290000	4.4307450000
C	-2.7490960000	5.1093220000	1.9820440000
H	-2.5842540000	5.2804060000	0.9200390000
H	-3.3332380000	4.1935970000	2.0809350000
H	-1.7720330000	4.9226310000	2.4314020000
C	-3.4806840000	9.2376430000	2.7824280000
H	-2.8022340000	10.0083470000	2.3978670000
C	-3.9218980000	9.6645470000	4.1746830000
H	-4.5358970000	8.9090490000	4.6657810000
H	-4.5172460000	10.5766670000	4.1179430000
H	-3.0729360000	9.8641730000	4.8269090000
C	-4.6583840000	9.1352380000	1.8266030000
H	-4.3652740000	8.8003890000	0.8313980000
H	-5.1360460000	10.1089800000	1.7122210000
H	-5.4204430000	8.4498490000	2.2006630000
C	1.6301450000	6.1609890000	6.4392880000
H	2.3573480000	6.9036430000	6.0892890000

C	0.9852680000	6.7133850000	7.7007560000
H	0.5170530000	7.6780360000	7.5193110000
H	1.7451730000	6.8509900000	8.4708000000
H	0.2302810000	6.0432630000	8.1099160000
C	2.3770810000	4.8628240000	6.7125460000
H	1.7220690000	4.0893330000	7.1133650000
H	3.1529320000	5.0375610000	7.4589330000
H	2.8689510000	4.4622510000	5.8268280000
C	-0.5106580000	4.5522120000	5.0529790000
H	-1.3073290000	4.7812060000	4.3417250000
C	-1.1710090000	4.3572410000	6.4105830000
H	-0.4518960000	4.0559880000	7.1717100000
H	-1.9190780000	3.5655840000	6.3473250000
H	-1.6746730000	5.2563340000	6.7628800000
C	0.1813660000	3.2834460000	4.5720720000
H	0.5314950000	3.3668650000	3.5445540000
H	-0.5220990000	2.4499380000	4.6071040000
H	1.0323960000	3.0107650000	5.1940060000
C	-1.6549310000	7.8289800000	5.9125300000
Ru	-0.5686940000	7.9611370000	4.4413140000

Ru-L-iPr-2-EG

Ru	-0.1796140000	-0.4943040000	-0.9463910000
P	1.9954020000	-0.8206450000	0.0954140000
P	-1.8202650000	-1.2801340000	0.3665750000
N	-0.3026580000	1.4339890000	0.1915220000
C	2.1742720000	0.5756650000	1.2940000000
H	1.3669030000	0.4397110000	2.0205150000
H	3.1258610000	0.5231960000	1.8245060000

C	2.0343920000	1.8939430000	0.6071900000
C	3.1282380000	2.7410390000	0.4831800000
H	4.0796260000	2.4238180000	0.8967400000
C	3.0206360000	3.9828990000	-0.1241350000
H	3.8814560000	4.6332100000	-0.1978790000
C	1.7906210000	4.3809050000	-0.6250710000
H	1.6864700000	5.3498070000	-1.1020970000
C	0.6799440000	3.5530160000	-0.5449940000
C	0.7791550000	2.2875200000	0.0790050000
C	-0.6375410000	3.9415120000	-1.1515800000
H	-0.6476220000	3.6576060000	-2.2179720000
H	-0.7536230000	5.0279340000	-1.1567650000
C	-1.7882470000	3.2771010000	-0.4594170000
C	-3.0653200000	3.8189290000	-0.4617670000
H	-3.2255980000	4.7827690000	-0.9334430000
C	-4.1328740000	3.1396210000	0.1051320000
H	-5.1267090000	3.5660010000	0.0862970000
C	-3.9070700000	1.9111920000	0.7095780000
H	-4.7267220000	1.3778910000	1.1803830000
C	-2.6340030000	1.3625200000	0.7578910000
C	-1.5567030000	2.0328300000	0.1440380000
C	-2.3495900000	0.0909470000	1.4769320000
H	-3.2084960000	-0.2351710000	2.0672170000
H	-1.5064570000	0.2415020000	2.1551130000
C	2.4310510000	-2.3568040000	1.0568710000
H	1.4716810000	-2.6397980000	1.4902890000
C	2.8383500000	-3.4960590000	0.1324270000
H	3.8442390000	-3.3589860000	-0.2627450000
H	2.1594540000	-3.6043770000	-0.7142280000

H	2.8345800000	-4.4399890000	0.6795170000
C	3.4039930000	-2.1784480000	2.2129150000
H	3.5518580000	-3.1337050000	2.7198250000
H	3.0308660000	-1.4751040000	2.9562590000
H	4.3836930000	-1.8313970000	1.8891640000
C	3.4068740000	-0.4783350000	-1.0705990000
H	3.2026800000	0.5665620000	-1.3281690000
C	3.3385750000	-1.2741030000	-2.3672000000
H	4.0529770000	-0.8692080000	-3.0860320000
H	2.3513820000	-1.2262650000	-2.8213190000
H	3.5904710000	-2.3236340000	-2.2249280000
C	4.7994160000	-0.5283970000	-0.4594260000
H	5.1114200000	-1.5499530000	-0.2416660000
H	4.8766580000	0.0486500000	0.4615900000
H	5.5286300000	-0.1170270000	-1.1595240000
C	-3.4170910000	-1.6772370000	-0.5013500000
H	-3.7388060000	-0.6676730000	-0.7850550000
C	-4.4834570000	-2.2832560000	0.3999200000
H	-5.4500090000	-2.2616350000	-0.1045420000
H	-4.6016690000	-1.7510100000	1.3436570000
H	-4.2705520000	-3.3284910000	0.6291560000
C	-3.2655740000	-2.4852490000	-1.7794090000
H	-2.8579670000	-3.4798090000	-1.5955560000
H	-2.6181240000	-1.9855270000	-2.4949810000
H	-4.2409740000	-2.6201970000	-2.2487400000
C	-1.5694050000	-2.6720790000	1.5709970000
H	-2.5645040000	-2.9352020000	1.9386670000
C	-0.9844300000	-3.8881650000	0.8683810000
H	-0.0448650000	-3.6497690000	0.3696680000

H	-1.6561660000	-4.2784150000	0.1040520000
H	-0.7929620000	-4.6927440000	1.5801170000
C	-0.7433620000	-2.2144000000	2.7694980000
H	-0.3139240000	-3.0697210000	3.2935670000
H	-1.3474270000	-1.6612530000	3.4861780000
H	0.0806780000	-1.5598280000	2.4793680000
C	-0.0806040000	-2.0272460000	-1.9340440000
O	0.0041360000	-3.0010500000	-2.5658130000
H	-1.5546700000	-0.0022070000	-1.6856870000
O	0.9714920000	0.8563920000	-2.4596500000
H	1.1256360000	1.6765380000	-1.9581390000
C	0.2845010000	1.1961980000	-3.6656890000
H	0.1919950000	0.2727120000	-4.2370060000
H	-0.7315840000	1.5399910000	-3.4347680000
C	1.0527540000	2.2528850000	-4.4291540000
H	2.0069620000	1.8537900000	-4.7753220000
H	1.2870730000	3.0885890000	-3.7502130000
O	0.3554040000	2.6836640000	-5.5742440000
H	-0.4583750000	3.1080770000	-5.2839950000

Ru-L-iPr-3-EG-H_2

Ru	-0.2039520000	-0.4767020000	-1.0295010000
P	1.8332800000	-0.7697920000	-0.0589730000
P	-1.7761350000	-1.3285280000	0.4786800000
N	-0.3701880000	1.4405120000	0.1522400000
C	2.0535950000	0.5635670000	1.1940850000
H	1.2604190000	0.4107140000	1.9325050000
H	3.0143550000	0.4482320000	1.6957790000
C	1.9385700000	1.9164640000	0.5770740000

C	3.0257050000	2.7794010000	0.5307510000
H	3.9721790000	2.4562630000	0.9512530000
C	2.9044430000	4.0490710000	-0.0098800000
H	3.7519370000	4.7209760000	-0.0255520000
C	1.6793160000	4.4480280000	-0.5248390000
H	1.5724330000	5.4358930000	-0.9605400000
C	0.5851410000	3.5984540000	-0.5183950000
C	0.6987510000	2.3165380000	0.0437410000
C	-0.7097150000	3.9835950000	-1.1675880000
H	-0.6264730000	3.7931050000	-2.2475200000
H	-0.8814070000	5.0603230000	-1.0865420000
C	-1.8762250000	3.2230510000	-0.6072380000
C	-3.1813610000	3.6777440000	-0.7286380000
H	-3.3604030000	4.6220040000	-1.2321650000
C	-4.2545540000	2.9458410000	-0.2362620000
H	-5.2663810000	3.3081760000	-0.3562030000
C	-4.0090750000	1.7607920000	0.4433070000
H	-4.8310170000	1.2055050000	0.8837900000
C	-2.7115520000	1.2935400000	0.6159620000
C	-1.6251130000	1.9946640000	0.0364750000
C	-2.4006410000	0.1016490000	1.4567110000
H	-3.2645350000	-0.2046580000	2.0500740000
H	-1.5847170000	0.3568750000	2.1380970000
C	2.2538000000	-2.3559590000	0.8213440000
H	1.2883320000	-2.6681770000	1.2181640000
C	2.7213920000	-3.4582680000	-0.1191270000
H	3.7286510000	-3.2728640000	-0.4898480000
H	2.0654750000	-3.5857600000	-0.9781600000
H	2.7480370000	-4.4079740000	0.4175840000

C	3.1979510000	-2.2005770000	2.0065380000
H	3.3667110000	-3.1756060000	2.4666250000
H	2.7934050000	-1.5454190000	2.7764190000
H	4.1717550000	-1.8096430000	1.7143480000
C	3.1847190000	-0.3866670000	-1.2654820000
H	2.9217300000	0.6467090000	-1.5168930000
C	3.1309330000	-1.1756470000	-2.5650480000
H	3.8544320000	-0.7574670000	-3.2663500000
H	2.1515710000	-1.1046720000	-3.0288830000
H	3.3826190000	-2.2268950000	-2.4352330000
C	4.5815180000	-0.4047120000	-0.6599170000
H	4.9189950000	-1.4177250000	-0.4376440000
H	4.6584150000	0.1836890000	0.2534060000
H	5.2928060000	0.0165140000	-1.3717630000
C	-3.3467560000	-1.8492930000	-0.3865500000
H	-3.7302930000	-0.8851440000	-0.7417190000
C	-4.3960830000	-2.4438240000	0.5428050000
H	-5.3505730000	-2.5219420000	0.0211200000
H	-4.5659280000	-1.8453070000	1.4375560000
H	-4.1262580000	-3.4520550000	0.8603840000
C	-3.1361190000	-2.7337110000	-1.6057520000
H	-2.6281750000	-3.6663660000	-1.3604040000
H	-2.5550360000	-2.2285790000	-2.3721030000
H	-4.1008350000	-2.9966020000	-2.0421340000
C	-1.5335620000	-2.6138470000	1.7944990000
H	-2.5177490000	-2.7673860000	2.2447930000
C	-1.0900700000	-3.9378780000	1.1850360000
H	-0.2400410000	-3.8261210000	0.5113010000
H	-1.8910780000	-4.3953870000	0.6051770000

H	-0.8063080000	-4.6471190000	1.9639180000
C	-0.6111000000	-2.0871040000	2.8916510000
H	-0.1746270000	-2.9080920000	3.4623730000
H	-1.1505650000	-1.4517510000	3.5916390000
H	0.2112690000	-1.4898410000	2.4968680000
C	-0.0327600000	-2.0701210000	-1.9128950000
O	0.0940740000	-3.1007800000	-2.4375410000
H	-2.5560780000	0.7911280000	-1.7717210000
O	-1.9801940000	0.2343240000	-2.3182290000
C	-1.4681640000	1.0512250000	-3.4049320000
H	-2.0873560000	0.8738610000	-4.2882420000
H	-1.5517360000	2.0996860000	-3.1157680000
C	-0.0197520000	0.6698890000	-3.6121280000
H	0.4047290000	1.3495860000	-4.3678830000
H	0.0111230000	-0.3430680000	-4.0600550000
O	0.6920200000	0.7555820000	-2.4365130000

Ru-L-^{i}Pr-4-CHO

Ru	-0.1540950000	-0.5632190000	-1.0077890000
P	2.0226440000	-0.8722400000	0.0575410000
P	-1.7978640000	-1.3150140000	0.3288050000
N	-0.2885300000	1.4374230000	0.0819950000
C	2.2109920000	0.6017710000	1.1497480000
H	1.4157630000	0.5107200000	1.8966460000
H	3.1698830000	0.5821440000	1.6694940000
C	2.0576120000	1.8895110000	0.4062020000
C	3.1571030000	2.7142410000	0.2150210000
H	4.1262900000	2.3777180000	0.5680110000
C	3.0340050000	3.9742830000	-0.3564350000

H	3.8977700000	4.6150890000	-0.4684180000
C	1.7711050000	4.4140860000	-0.7219110000
H	1.6396410000	5.4233150000	-1.1047770000
C	0.6517440000	3.5977760000	-0.5993470000
C	0.7771690000	2.2971440000	-0.0528720000
C	-0.6981070000	4.0843350000	-1.0422940000
H	-0.7744500000	4.0377290000	-2.1393480000
H	-0.8053020000	5.1488700000	-0.8124440000
C	-1.8166130000	3.3033880000	-0.4224650000
C	-3.1051370000	3.8172060000	-0.3805040000
H	-3.2862460000	4.8034800000	-0.7946680000
C	-4.1530170000	3.0933120000	0.1641110000
H	-5.1551910000	3.4998450000	0.1765920000
C	-3.8888350000	1.8520020000	0.7220120000
H	-4.6840730000	1.2889370000	1.1998550000
C	-2.6045200000	1.3276490000	0.7230190000
C	-1.5511340000	2.0268450000	0.0965360000
C	-2.2913320000	0.0618370000	1.4389520000
H	-3.1305140000	-0.2572600000	2.0606110000
H	-1.4285490000	0.2205510000	2.0900200000
C	2.4259270000	-2.3385970000	1.1337300000
H	1.4568050000	-2.5779450000	1.5706310000
C	2.8303050000	-3.5444190000	0.2959960000
H	3.8449530000	-3.4498180000	-0.0891400000
H	2.1649990000	-3.6988400000	-0.5546120000
H	2.8009950000	-4.4495340000	0.9044940000
C	3.3848040000	-2.0933370000	2.2891350000
H	3.5027180000	-3.0100400000	2.8697800000
H	3.0170680000	-1.3271480000	2.9704690000

H	4.3772280000	-1.7941120000	1.9567930000
C	3.4603810000	-0.6331010000	-1.1021200000
H	3.2750570000	0.3959110000	-1.4281280000
C	3.4021460000	-1.5117430000	-2.3442500000
H	4.1256020000	-1.1582630000	-3.0812330000
H	2.4206440000	-1.4937810000	-2.8113820000
H	3.6511700000	-2.5494580000	-2.1283210000
C	4.8419250000	-0.6665410000	-0.4651850000
H	5.1343780000	-1.6764270000	-0.1776330000
H	4.9172180000	-0.0328240000	0.4179000000
H	5.5882980000	-0.3135230000	-1.1791790000
C	-3.4045410000	-1.6768120000	-0.5372550000
H	-3.6895620000	-0.6633870000	-0.8445640000
C	-4.4959540000	-2.2269700000	0.3696750000
H	-5.4596960000	-2.1742130000	-0.1379190000
H	-4.5955470000	-1.6773820000	1.3056150000
H	-4.3251690000	-3.2767310000	0.6132780000
C	-3.2739010000	-2.5175580000	-1.7963760000
H	-2.9058250000	-3.5225530000	-1.5869520000
H	-2.6037330000	-2.0580290000	-2.5179680000
H	-4.2503450000	-2.6268320000	-2.2701760000
C	-1.5823370000	-2.7116470000	1.5358970000
H	-2.5908410000	-2.9765680000	1.8631070000
C	-0.9705360000	-3.9274950000	0.8573610000
H	0.0044220000	-3.6997310000	0.4269730000
H	-1.5942640000	-4.2964620000	0.0434220000
H	-0.8400820000	-4.7436620000	1.5697080000
C	-0.8072310000	-2.2589240000	2.7701780000
H	-0.3993960000	-3.1169710000	3.3068020000

H	-1.4411930000	-1.7099040000	3.4638510000
H	0.0274550000	-1.6015540000	2.5202200000
C	-0.0746740000	-2.1375200000	-1.9230530000
O	-0.0055100000	-3.1433460000	-2.5029420000
H	-1.5073910000	-0.0623680000	-1.7756780000
O	1.0971150000	0.5751560000	-2.4894680000
C	0.8604650000	1.6917550000	-2.9109850000
H	-0.1453510000	2.1395260000	-2.8203250000
C	1.9136120000	2.5134300000	-3.5869770000
H	2.1713580000	2.0272010000	-4.5333410000
H	2.8180150000	2.4712130000	-2.9621320000
O	1.5041420000	3.8200260000	-3.8699620000
H	1.5402470000	4.3098710000	-3.0373860000

Ru-L-^{i}Pr-5-acetal

P	-2.3119410000	7.8338610000	2.9190130000
P	0.3504320000	6.1329750000	4.8506720000
O	-2.4133820000	7.8781280000	6.7960430000
N	0.7561210000	8.4016580000	2.6425310000
C	-1.5996330000	8.0154590000	1.2360090000
H	-0.9601910000	7.1369870000	1.1144030000
H	-2.4112040000	7.9413540000	0.5088970000
C	-0.7774550000	9.2341370000	0.9977100000
C	-1.1357510000	10.1758600000	0.0443250000
H	-2.0871160000	10.0712960000	-0.4667100000
C	-0.2757110000	11.2111300000	-0.2946700000
H	-0.5553170000	11.9304600000	-1.0521540000
C	0.9613070000	11.2900670000	0.3292200000
H	1.6608980000	12.0709970000	0.0457770000

C	1.3281930000	10.3889430000	1.3206270000
C	0.4487210000	9.3539470000	1.6831030000
C	2.6353910000	10.5121410000	2.0487880000
H	3.4100180000	10.9033380000	1.3835880000
H	2.5435730000	11.2713640000	2.8410950000
C	3.0755110000	9.2039180000	2.6408950000
C	4.4095360000	8.9570290000	2.9339160000
H	5.1390840000	9.7347590000	2.7349840000
C	4.8186490000	7.7457720000	3.4700640000
H	5.8604310000	7.5708780000	3.7015610000
C	3.8775830000	6.7473960000	3.6689790000
H	4.1907870000	5.7751280000	4.0342960000
C	2.5374810000	6.9490170000	3.3660580000
C	2.1048130000	8.2099850000	2.8913820000
C	1.5521760000	5.8323910000	3.4866160000
H	2.0684090000	4.8823460000	3.6266970000
H	0.9527430000	5.7662380000	2.5731870000
C	-3.2280320000	6.2321500000	2.7503280000
H	-4.0384060000	6.4538110000	2.0515150000
C	-3.8660990000	5.8215360000	4.0714810000
H	-3.1555640000	5.8188110000	4.8971500000
H	-4.2979820000	4.8221990000	3.9988290000
H	-4.6674750000	6.5039520000	4.3520380000
C	-2.3634260000	5.1544120000	2.0942900000
H	-2.3756470000	5.2462350000	1.0098990000
H	-2.7298380000	4.1570100000	2.3420650000
H	-1.3170780000	5.2026880000	2.3977720000
C	-3.6301510000	9.1410430000	2.9391130000
H	-3.0643040000	10.0036760000	2.5749030000

C	-4.1441010000	9.4930090000	4.3261350000
H	-4.6238220000	8.6483050000	4.8221830000
H	-4.8917450000	10.2834600000	4.2468160000
H	-3.3387110000	9.8611300000	4.9552130000
C	-4.7772730000	8.8614480000	1.9783970000
H	-4.4464650000	8.5755480000	0.9796590000
H	-5.3832440000	9.7612600000	1.8685210000
H	-5.4411370000	8.0789300000	2.3497710000
C	1.5092980000	6.1891140000	6.3097170000
H	2.2188510000	6.9472930000	5.9656190000
C	0.8897990000	6.7091360000	7.5985890000
H	0.3002150000	7.6076970000	7.4398760000
H	1.6776930000	6.9546340000	8.3127070000
H	0.2451070000	5.9741090000	8.0769070000
C	2.2953630000	4.9096190000	6.5612220000
H	1.6705700000	4.1190050000	6.9762720000
H	3.0854740000	5.1023230000	7.2888380000
H	2.7752450000	4.5213770000	5.6641270000
C	-0.6414830000	4.5649220000	5.0186260000
H	-1.4820560000	4.7473330000	4.3538460000
C	-1.2303550000	4.3987860000	6.4131490000
H	-0.4811280000	4.0882270000	7.1397450000
H	-1.9998680000	3.6253460000	6.3972770000
H	-1.6948040000	5.3134490000	6.7816540000
C	0.0396930000	3.2945280000	4.5302410000
H	0.3187140000	3.3561130000	3.4791680000
H	-0.6450800000	2.4508300000	4.6328650000
H	0.9360000000	3.0516720000	5.0983570000
C	-1.7298710000	8.0216640000	5.8676110000

Ru	-0.6299500000	8.2107230000	4.4208370000
O	1.1505410000	9.1013580000	5.5479980000
H	1.8348220000	9.3528340000	4.9053920000
C	1.0640570000	10.0959540000	6.5839420000
H	1.9321770000	9.9852490000	7.2430900000
O	-1.2232960000	10.1846340000	4.0048500000
C	-0.3513810000	11.2026070000	4.0515860000
H	0.6767490000	10.9266660000	3.7470360000
C	-0.8314580000	12.3350130000	3.1545440000
H	-1.6687700000	12.8330340000	3.6479580000
H	-1.1941160000	11.9028800000	2.2168160000
O	0.1727800000	13.3066680000	2.9229830000
H	0.6800070000	12.9934250000	2.1654330000
O	-0.2188490000	11.7742040000	5.3860280000
C	0.9729540000	11.5004010000	6.0425920000
H	1.0541880000	12.1954260000	6.8832700000
H	1.8447020000	11.6932430000	5.3925450000
H	0.1651830000	9.8490790000	7.1485560000

mer-Ru-LS-Ph-1

Ru	1.2049960000	10.7973030000	10.6146220000
H	1.4825270000	9.9512380000	11.9142160000
P	-0.6600210000	9.4135260000	10.5829690000
P	3.3363270000	11.6351110000	10.8537690000
O	0.0200520000	12.9450950000	12.3197250000
O	0.9301100000	11.9844760000	8.5591400000
N	2.2739240000	9.3334330000	9.2931530000
C	0.1062130000	7.7490500000	10.5445660000
H	0.8706460000	7.7684780000	11.3236130000

H	-0.6212650000	6.9791570000	10.8118790000
C	0.7046790000	7.4372750000	9.2128940000
C	0.1976880000	6.3355220000	8.5304870000
H	-0.6106270000	5.7797010000	8.9933050000
C	0.7156300000	5.9122150000	7.3210100000
H	0.3120680000	5.0445610000	6.8173030000
C	1.7819800000	6.6158450000	6.7921610000
H	2.2374080000	6.3001970000	5.8597450000
C	2.2924570000	7.7365140000	7.4263430000
C	1.7567250000	8.2047000000	8.6486460000
C	3.4338160000	8.4801140000	6.8141830000
H	4.0381160000	7.8177650000	6.1910330000
H	3.0496090000	9.2488490000	6.1256570000
C	4.2617030000	9.1396560000	7.8637390000
C	3.5779220000	9.6674790000	8.9891940000
C	5.6208740000	9.3271030000	7.7147650000
H	6.1106020000	8.8781530000	6.8568000000
C	6.3603650000	10.0995060000	8.6069810000
H	7.4266010000	10.2262340000	8.4806820000
C	5.6815400000	10.7553800000	9.6138050000
H	6.2108380000	11.4389920000	10.2682100000
C	4.3111300000	10.5772450000	9.7914500000
C	-1.8225580000	9.3469250000	9.1370970000
H	-1.1489760000	8.9921140000	8.3473670000
C	-2.3253920000	10.7224100000	8.7251840000
H	-3.0747730000	11.1117290000	9.4132390000
H	-2.7887450000	10.6805230000	7.7378540000
H	-1.5094940000	11.4428590000	8.6873610000
C	-2.9491140000	8.3362740000	9.2779090000

H	-2.5835190000	7.3449980000	9.5450890000
H	-3.4929820000	8.2368820000	8.3372760000
H	-3.6725350000	8.6417660000	10.0350550000
C	-1.7302930000	9.3255120000	12.0924150000
H	-2.3089080000	8.4022120000	11.9739330000
C	-2.6951360000	10.4974880000	12.1953560000
H	-2.1704470000	11.4524270000	12.1631340000
H	-3.2351620000	10.4572350000	13.1422750000
H	-3.4379510000	10.4952020000	11.3999230000
C	-0.8894720000	9.2033380000	13.3549030000
H	-0.1719440000	8.3852960000	13.3028350000
H	-1.5346360000	9.0242960000	14.2160460000
H	-0.3307200000	10.1200220000	13.5425940000
C	3.5632260000	13.3094480000	10.1644880000
C	3.2158820000	14.4205070000	10.9382160000
H	2.9194070000	14.2821110000	11.9719980000
C	3.2425140000	15.6948540000	10.3976850000
H	2.9736010000	16.5441970000	11.0126070000
C	3.6115800000	15.8823460000	9.0718870000
H	3.6355600000	16.8787250000	8.6498160000
C	3.9576900000	14.7870660000	8.2954150000
H	4.2554990000	14.9263940000	7.2633790000
C	3.9310380000	13.5085650000	8.8339710000
H	4.2053450000	12.6577060000	8.2207730000
C	4.1534980000	11.7591910000	12.4783070000
C	3.6736690000	11.0189990000	13.5568390000
H	2.7862160000	10.4122930000	13.4243940000
C	4.3158720000	11.0623490000	14.7841890000
H	3.9280230000	10.4842900000	15.6131360000

C	5.4451290000	11.8483180000	14.9523420000
H	5.9445850000	11.8845810000	15.9120350000
C	5.9284610000	12.5961970000	13.8886460000
H	6.8059880000	13.2173950000	14.0147240000
C	5.2865510000	12.5552070000	12.6620290000
H	5.6609390000	13.1571980000	11.8424150000
C	0.4592590000	12.0977490000	11.6522020000
C	0.4827850000	13.3476010000	8.4827980000
H	1.1050690000	13.9500560000	9.1457120000
H	-0.5501230000	13.3989100000	8.8476890000
C	0.5903970000	13.7191830000	7.0207580000
H	1.5989530000	14.0700680000	6.7971230000
H	-0.1072880000	14.5040710000	6.7366800000
C	0.3252140000	12.3877460000	6.3322150000
H	-0.7455210000	12.1782280000	6.3072080000
H	0.6960290000	12.3440550000	5.3103780000
C	1.0322330000	11.4120150000	7.2472210000
H	0.5922800000	10.4129100000	7.2736970000
H	2.0934050000	11.3044840000	6.9951880000

fac-Ru-LS-Ph-1

Ru	-0.2801670000	4.8011460000	3.0340170000
H	0.8061880000	6.0037530000	3.3355930000
P	-1.5321340000	2.7594560000	2.9412940000
P	-1.7084890000	6.2434410000	3.9117790000
O	-0.8103910000	5.8441820000	0.2796890000
N	0.0105370000	4.2002180000	5.1359800000
C	-0.3308120000	1.5557790000	3.6076580000
H	-0.7340620000	0.5442630000	3.5120060000

H	0.5004340000	1.6087960000	2.8973750000
C	0.1997180000	1.7332140000	5.0081800000
C	0.5441920000	0.5481350000	5.6573630000
H	0.3859500000	-0.3826840000	5.1235550000
C	1.0481940000	0.5148760000	6.9433200000
H	1.2907090000	-0.4260680000	7.4179270000
C	1.2293180000	1.7180960000	7.6026870000
H	1.6297610000	1.7386330000	8.6102560000
C	0.9225890000	2.9181610000	6.9881940000
C	0.3802950000	2.9692810000	5.6807740000
C	1.1908370000	4.2172140000	7.6767210000
H	1.3104620000	4.0774600000	8.7519600000
C	0.1160040000	5.1996520000	7.3613500000
C	-0.3562490000	5.1847530000	6.0310530000
C	-0.3370730000	6.1465240000	8.2569590000
H	0.0467990000	6.1266530000	9.2714340000
C	-1.2291130000	7.1455280000	7.8742640000
H	-1.5586290000	7.8919540000	8.5838590000
C	-1.6498330000	7.1899640000	6.5597160000
H	-2.3082550000	7.9852450000	6.2272990000
C	-1.2359350000	6.2234610000	5.6438150000
C	-1.9057290000	2.0181820000	1.2818660000
H	-2.1796410000	0.9731130000	1.4586430000
C	-0.6926360000	2.0465530000	0.3656570000
H	-0.3969250000	3.0728210000	0.1472950000
H	-0.9276040000	1.5676140000	-0.5858490000
H	0.1694180000	1.5311790000	0.7878410000
C	-3.0775680000	2.7417940000	0.6330620000
H	-3.9839440000	2.7047320000	1.2371580000

H	-3.3095060000	2.3011910000	-0.3373960000
H	-2.8358400000	3.7924590000	0.4630010000
C	-3.1050270000	2.4462790000	3.8850080000
H	-3.8102830000	3.1751300000	3.4715770000
C	-2.9414130000	2.7404880000	5.3697030000
H	-2.3241140000	1.9868110000	5.8591570000
H	-3.9179020000	2.7341450000	5.8564020000
H	-2.4838110000	3.7100050000	5.5547220000
C	-3.6800040000	1.0506120000	3.6857940000
H	-3.9615800000	0.8457650000	2.6544870000
H	-4.5775150000	0.9324930000	4.2947610000
H	-2.9779630000	0.2780490000	4.0045190000
C	-1.6680970000	7.9942610000	3.3814290000
C	-0.5187900000	8.5368710000	2.8100680000
H	0.3352550000	7.8930430000	2.6417700000
C	-0.4709650000	9.8762960000	2.4595600000
H	0.4274460000	10.2799920000	2.0103820000
C	-1.5682420000	10.6956350000	2.6762450000
H	-1.5301640000	11.7413920000	2.3990260000
C	-2.7180550000	10.1677870000	3.2428960000
H	-3.5810640000	10.7988700000	3.4125730000
C	-2.7701310000	8.8269230000	3.5884780000
H	-3.6790310000	8.4241310000	4.0189570000
C	-3.4976170000	5.8965810000	3.7906310000
C	-4.0533470000	5.7969400000	2.5126720000
H	-3.4305660000	5.9763640000	1.6424780000
C	-5.3869100000	5.4667280000	2.3471870000
H	-5.8021820000	5.3883490000	1.3502440000
C	-6.1871490000	5.2297700000	3.4583540000

H	-7.2285380000	4.9639400000	3.3303140000
C	-5.6487280000	5.3410310000	4.7305760000
H	-6.2694260000	5.1650040000	5.6000880000
C	-4.3116010000	5.6770250000	4.8975240000
H	-3.8924870000	5.7532420000	5.8938230000
C	-0.5855030000	5.4237340000	1.3423310000
H	2.1491220000	4.6235500000	7.3169610000
C	2.3027720000	4.1261160000	1.2522640000
H	2.2830350000	5.2212860000	1.2612980000
C	3.7091570000	3.5896160000	1.3138270000
H	4.3911120000	4.1171310000	0.6503170000
H	3.7244120000	2.5325540000	1.0427670000
C	4.0314850000	3.7802680000	2.7893150000
H	4.8286460000	3.1326570000	3.1473190000
H	4.3376350000	4.8106430000	2.9728160000
C	2.7071090000	3.4922930000	3.4751780000
O	1.6831450000	3.6305110000	2.4522520000
H	1.7062170000	3.7768220000	0.4110020000
H	2.6369610000	2.4726810000	3.8591050000
H	2.4760520000	4.1811370000	4.2882800000

Ru-LS-Ph-2-EG

Ru	-0.2008440000	4.6226830000	3.3176540000
H	0.9076840000	5.7771580000	3.6982960000
P	-1.5264020000	2.6381760000	3.1306640000
P	-1.6259010000	6.1627730000	4.0209140000
O	-0.3409820000	5.6345310000	0.5018330000
N	-0.1280420000	4.0692100000	5.4326750000
C	-0.5309830000	1.3597040000	3.9788710000

H	-1.1155760000	0.4363940000	4.0166550000
H	0.2842570000	1.1503520000	3.2816390000
C	0.0524370000	1.6010150000	5.3554910000
C	0.4306650000	0.4323730000	6.0203650000
H	0.3011120000	-0.5079970000	5.4954460000
C	0.9131620000	0.4243060000	7.3127590000
H	1.1805980000	-0.5048410000	7.7970960000
C	1.0241640000	1.6358180000	7.9756880000
H	1.3829240000	1.6714740000	8.9984140000
C	0.7006650000	2.8219510000	7.3473480000
C	0.2142820000	2.8494840000	6.0137760000
C	0.8869580000	4.1280760000	8.0520550000
H	0.9028440000	3.9884760000	9.1341100000
C	-0.1538610000	5.1121560000	7.6379120000
C	-0.5246950000	5.0837200000	6.2771240000
C	-0.6515590000	6.0885640000	8.4768030000
H	-0.3490030000	6.0816820000	9.5186130000
C	-1.4815330000	7.1027250000	8.0048480000
H	-1.8470240000	7.8705980000	8.6726910000
C	-1.7867420000	7.1383260000	6.6580990000
H	-2.3848060000	7.9502700000	6.2586980000
C	-1.3279350000	6.1442580000	5.7951060000
C	-1.7213360000	1.8835470000	1.4475390000
H	-2.0766080000	0.8580580000	1.5948470000
C	-0.3985900000	1.8330400000	0.6963510000
H	-0.0027010000	2.8350500000	0.5306350000
H	-0.5403370000	1.3729630000	-0.2823490000
H	0.3648110000	1.2585540000	1.2196010000
C	-2.7567800000	2.6655220000	0.6506210000

H	-3.7323680000	2.6909580000	1.1360070000
H	-2.8911800000	2.2266670000	-0.3386820000
H	-2.4327870000	3.6974920000	0.5072760000
C	-3.2161930000	2.4485410000	3.8814570000
H	-3.8124530000	3.2413910000	3.4190340000
C	-3.1585200000	2.7083810000	5.3803250000
H	-2.6114560000	1.9197300000	5.8997840000
H	-4.1674480000	2.7368140000	5.7943900000
H	-2.6738730000	3.6540300000	5.6171490000
C	-3.8882270000	1.1135740000	3.5947850000
H	-4.0666400000	0.9451280000	2.5342220000
H	-4.8568110000	1.0746310000	4.0956260000
H	-3.3031040000	0.2741450000	3.9741410000
C	-1.4425750000	7.9007220000	3.4821460000
C	-0.2144630000	8.3743540000	3.0247310000
H	0.6177670000	7.6859790000	2.9500270000
C	-0.0616530000	9.7038500000	2.6664600000
H	0.8973480000	10.0538920000	2.3065800000
C	-1.1312880000	10.5809850000	2.7609580000
H	-1.0109310000	11.6187640000	2.4773850000
C	-2.3587840000	10.1215780000	3.2128260000
H	-3.2005400000	10.7982730000	3.2860990000
C	-2.5153820000	8.7910520000	3.5666240000
H	-3.4830100000	8.4417810000	3.9063880000
C	-3.4100290000	5.9003220000	3.7326100000
C	-3.8452430000	5.8076740000	2.4080920000
H	-3.1352240000	5.9520690000	1.6004250000
C	-5.1683590000	5.5244200000	2.1188520000
H	-5.4900990000	5.4508640000	1.0876020000

C	-6.0783650000	5.3267800000	3.1506080000
H	-7.1118410000	5.0967190000	2.9258150000
C	-5.6593200000	5.4311140000	4.4675190000
H	-6.3656020000	5.2854770000	5.2751660000
C	-4.3326290000	5.7208220000	4.7585810000
H	-4.0071990000	5.7904850000	5.7897640000
C	-0.2712830000	5.2207630000	1.5871400000
H	1.8739410000	4.5400150000	7.7916520000
C	3.8506290000	2.6232220000	2.4614090000
H	3.4526440000	1.7447300000	1.9363530000
C	2.7906600000	3.6963690000	2.5089680000
O	1.6236960000	3.1995920000	3.1683520000
H	3.1589570000	4.6001630000	3.0000900000
H	2.4749540000	3.9748640000	1.5042640000
H	1.8202770000	3.0732830000	4.1098050000
H	4.0939240000	2.3041250000	3.4857410000
O	4.9686280000	3.1753500000	1.8054210000
H	5.6514780000	2.5032090000	1.7480350000

Ru-LS-Ph-3-EG-H_2

Ru	-0.2519770000	4.5534060000	2.9171260000
P	-1.4905410000	2.6608660000	2.8518250000
P	-1.7265140000	6.0539970000	3.8194090000
O	-0.9112930000	5.5457070000	0.1646500000
N	0.0831980000	4.0640390000	5.0066700000
C	-0.3559630000	1.4200030000	3.5486840000
H	-0.8518190000	0.4467940000	3.5062380000
H	0.4572790000	1.3997600000	2.8187760000
C	0.2458120000	1.6067540000	4.9162580000

C	0.5986670000	0.4302780000	5.5739240000
H	0.3985970000	-0.5099570000	5.0721180000
C	1.1717480000	0.4192570000	6.8318170000
H	1.4233210000	-0.5156910000	7.3137490000
C	1.4129320000	1.6304030000	7.4555450000
H	1.8653680000	1.6627170000	8.4404310000
C	1.0937610000	2.8221310000	6.8297780000
C	0.4904080000	2.8455090000	5.5531950000
C	1.4036300000	4.1373880000	7.4716020000
H	1.5737920000	4.0245720000	8.5431070000
C	0.3194440000	5.1225910000	7.1884080000
C	-0.2381320000	5.0716330000	5.8887670000
C	-0.0838980000	6.0926360000	8.0821860000
H	0.3559740000	6.0988850000	9.0738070000
C	-1.0013620000	7.0803720000	7.7269660000
H	-1.2878220000	7.8455720000	8.4351140000
C	-1.5049640000	7.0876950000	6.4416550000
H	-2.1874540000	7.8718190000	6.1328750000
C	-1.1468130000	6.0997180000	5.5230380000
C	-1.8735520000	1.9545010000	1.1881410000
H	-2.1970100000	0.9279370000	1.3915050000
C	-0.6502410000	1.8929950000	0.2858640000
H	-0.2531030000	2.8843220000	0.0760170000
H	-0.9211350000	1.4348240000	-0.6662360000
H	0.1601390000	1.3118640000	0.7195610000
C	-3.0194220000	2.7066220000	0.5243150000
H	-3.9229750000	2.7263710000	1.1338880000
H	-3.2752720000	2.2377190000	-0.4262460000
H	-2.7419500000	3.7377600000	0.3086870000

C	-3.0809720000	2.4586960000	3.7849510000
H	-3.7416250000	3.2345160000	3.3888640000
C	-2.8868140000	2.7040360000	5.2741740000
H	-2.2831840000	1.9188070000	5.7304850000
H	-3.8555600000	2.7119970000	5.7753540000
H	-2.3981270000	3.6536850000	5.4808480000
C	-3.7417370000	1.1038270000	3.5581730000
H	-4.0415430000	0.9404250000	2.5250840000
H	-4.6412720000	1.0333690000	4.1709680000
H	-3.0900560000	0.2811350000	3.8562830000
C	-1.6320120000	7.7827830000	3.2211220000
C	-0.5351780000	8.2125490000	2.4756450000
H	0.2456280000	7.5091680000	2.2175950000
C	-0.4382710000	9.5314900000	2.0597900000
H	0.4169830000	9.8459200000	1.4756030000
C	-1.4343380000	10.4402090000	2.3803950000
H	-1.3592430000	11.4686470000	2.0512870000
C	-2.5339690000	10.0235060000	3.1157710000
H	-3.3205240000	10.7247850000	3.3635440000
C	-2.6346280000	8.7055010000	3.5292230000
H	-3.5076640000	8.3876040000	4.0869230000
C	-3.5342730000	5.8155770000	3.8189190000
C	-4.1725550000	5.7628320000	2.5766520000
H	-3.5937310000	5.9097940000	1.6704040000
C	-5.5315980000	5.5171490000	2.4938840000
H	-6.0129700000	5.4750250000	1.5250780000
C	-6.2737580000	5.3199880000	3.6521460000
H	-7.3357550000	5.1213830000	3.5884260000
C	-5.6513640000	5.3846460000	4.8887880000

H	-6.2270080000	5.2402560000	5.7942850000
C	-4.2881780000	5.6343780000	4.9737350000
H	-3.8033150000	5.6738630000	5.9420270000
C	-0.6217730000	5.1262420000	1.2091470000
H	2.3479020000	4.5265550000	7.0591070000
C	2.7280600000	5.1236650000	3.2378750000
H	3.5793410000	5.7767410000	3.0287560000
C	2.4652840000	4.1064490000	2.1564110000
O	1.3851930000	3.3202300000	2.4797250000
H	2.3367050000	4.6492800000	1.1983000000
H	3.3703070000	3.4902530000	2.0396520000
H	2.8741380000	4.6167210000	4.1949420000
O	1.5308120000	5.9469340000	3.3195820000
H	1.4219810000	6.2418320000	4.2342140000

Ru-LS-Ph-4-CHO

Ru	-0.2567730000	4.9389260000	2.9929340000
H	0.7773920000	6.1796070000	3.2955710000
P	-1.4259620000	2.8480810000	2.8918350000
P	-1.7264740000	6.3129420000	3.9285560000
O	-0.8411330000	6.0159270000	0.2615640000
N	0.0903800000	4.3238090000	5.0747990000
C	-0.2134810000	1.6496460000	3.5594670000
H	-0.6861410000	0.6651310000	3.6168710000
H	0.5286410000	1.5602760000	2.7597950000
C	0.5086580000	1.9000330000	4.8644230000
C	1.0944890000	0.7583440000	5.4180710000
H	1.0011500000	-0.1719120000	4.8679830000
C	1.7428720000	0.7602730000	6.6385630000

H	2.1693370000	-0.1485940000	7.0398810000
C	1.8114590000	1.9569550000	7.3395570000
H	2.3031570000	1.9986930000	8.3048910000
C	1.2847450000	3.1203100000	6.8139080000
C	0.6199790000	3.1383100000	5.5566680000
C	1.4431310000	4.4202050000	7.5359370000
H	1.6203330000	4.2577410000	8.6000860000
C	0.2722460000	5.3135080000	7.2976390000
C	-0.2741590000	5.2813670000	5.9989080000
C	-0.1912040000	6.2238570000	8.2261570000
H	0.2470660000	6.2243740000	9.2184620000
C	-1.1620000000	7.1670650000	7.8984880000
H	-1.5012070000	7.8848270000	8.6326140000
C	-1.6438490000	7.2050010000	6.6040220000
H	-2.3550970000	7.9701820000	6.3125810000
C	-1.2183890000	6.2756280000	5.6563800000
C	-1.7741050000	2.1207170000	1.2221510000
H	-2.0350410000	1.0684880000	1.3765080000
C	-0.5585620000	2.1887670000	0.3098210000
H	-0.2318530000	3.2177050000	0.1613010000
H	-0.8045190000	1.7778160000	-0.6700490000
H	0.2915710000	1.6273750000	0.6937700000
C	-2.9561130000	2.8442520000	0.5901100000
H	-3.8585110000	2.7950060000	1.1997020000
H	-3.1913550000	2.4135720000	-0.3837390000
H	-2.7217630000	3.8980520000	0.4311700000
C	-2.9824070000	2.4801990000	3.8418980000
H	-3.7005150000	3.2259390000	3.4867960000
C	-2.7665420000	2.7011230000	5.3321860000

H	-2.0857250000	1.9573940000	5.7500260000
H	-3.7152010000	2.6123600000	5.8636090000
H	-2.3534540000	3.6842060000	5.5498270000
C	-3.5632520000	1.0967840000	3.5862290000
H	-3.8677710000	0.9469820000	2.5520530000
H	-4.4476480000	0.9494460000	4.2079470000
H	-2.8589650000	0.3048510000	3.8469920000
C	-1.7646470000	8.0765970000	3.4460080000
C	-0.6477840000	8.6761210000	2.8673370000
H	0.2268500000	8.0707920000	2.6657420000
C	-0.6579230000	10.0246620000	2.5500350000
H	0.2156010000	10.4733090000	2.0946890000
C	-1.7815870000	10.7951010000	2.8068620000
H	-1.7888670000	11.8479170000	2.5551090000
C	-2.8997880000	10.2097090000	3.3804610000
H	-3.7830860000	10.8025870000	3.5806980000
C	-2.8940060000	8.8600400000	3.6938530000
H	-3.7786190000	8.4118720000	4.1301710000
C	-3.5023190000	5.8966290000	3.8468070000
C	-4.0881850000	5.8027950000	2.5817430000
H	-3.4982120000	6.0314150000	1.7002250000
C	-5.4091070000	5.4142670000	2.4436470000
H	-5.8482660000	5.3416550000	1.4566190000
C	-6.1658390000	5.1111630000	3.5692660000
H	-7.1967860000	4.7994600000	3.4621140000
C	-5.5979560000	5.2159120000	4.8291540000
H	-6.1852750000	4.9890090000	5.7099890000
C	-4.2739530000	5.6111140000	4.9689270000
H	-3.8316800000	5.6822010000	5.9555470000

C	-0.5911990000	5.5794860000	1.3094320000
H	2.3460880000	4.9251990000	7.1582950000
C	3.5127720000	2.5450390000	3.4030120000
H	4.3345390000	2.8251020000	2.7350810000
C	2.4834270000	3.6329540000	3.3663600000
O	1.5916560000	3.6833170000	2.5387550000
H	2.5620780000	4.3898300000	4.1684660000
H	3.0740870000	1.6256520000	2.9930260000
O	4.0490050000	2.3864980000	4.6847700000
H	3.3578070000	2.0216460000	5.2582350000

Ru-LS-Ph-5-acetal

Ru	4.6841760000	6.6091160000	3.8861870000
P	3.4383150000	4.7182720000	3.9012450000
P	3.2637410000	8.0936910000	4.8855510000
O	3.8975680000	7.5899580000	1.1608260000
N	5.1795930000	6.1279600000	5.9274430000
C	4.5595240000	3.4448470000	4.5625340000
H	3.9803610000	2.5245110000	4.6815040000
H	5.2509390000	3.2720680000	3.7347030000
C	5.3661250000	3.6715240000	5.8174050000
C	5.8801750000	2.5090310000	6.3939300000
H	5.6658880000	1.5646840000	5.9057620000
C	6.6273560000	2.5164420000	7.5559980000
H	7.0022700000	1.5923630000	7.9741350000
C	6.8719630000	3.7310880000	8.1764320000
H	7.4453580000	3.7746570000	9.0954590000
C	6.4036500000	4.9090510000	7.6242740000
C	5.6476730000	4.9128270000	6.4298970000

C	6.7103110000	6.2361520000	8.2435790000
H	7.0116720000	6.1278420000	9.2859270000
C	5.5542900000	7.1692940000	8.0920450000
C	4.8890370000	7.1180690000	6.8490510000
C	5.1945370000	8.1134530000	9.0322080000
H	5.7169640000	8.1287230000	9.9826080000
C	4.2165660000	9.0685400000	8.7641390000
H	3.9599010000	9.8138110000	9.5042560000
C	3.6166300000	9.0811210000	7.5196450000
H	2.8944470000	9.8527340000	7.2792940000
C	3.9327850000	8.1193310000	6.5603590000
C	2.9616630000	4.0088690000	2.2635580000
H	2.6427130000	2.9848720000	2.4878280000
C	4.1287200000	3.9405830000	1.2898880000
H	4.5138450000	4.9291150000	1.0494490000
H	3.7991380000	3.4764830000	0.3595710000
H	4.9666640000	3.3632410000	1.6737890000
C	1.7853520000	4.7703370000	1.6659830000
H	0.9206960000	4.8073390000	2.3287430000
H	1.4667420000	4.2986280000	0.7361610000
H	2.0623850000	5.7961290000	1.4258940000
C	1.9006410000	4.5311570000	4.9221720000
H	1.2336570000	5.3289180000	4.5882080000
C	2.2094770000	4.7398910000	6.3976770000
H	2.8411190000	3.9403850000	6.7877790000
H	1.2837330000	4.7404340000	6.9743360000
H	2.7197430000	5.6819110000	6.5886040000
C	1.1871990000	3.2000200000	4.7170000000
H	0.8192620000	3.0625280000	3.7025640000

H	0.3249260000	3.1485240000	5.3830410000
H	1.8266400000	2.3510310000	4.9622710000
C	3.2970370000	9.8318780000	4.3012620000
C	4.2717970000	10.2729360000	3.4093880000
H	5.0004160000	9.5729940000	3.0290450000
C	4.3092110000	11.5952770000	2.9958750000
H	5.0704070000	11.9156130000	2.2960860000
C	3.3716990000	12.4997520000	3.4676990000
H	3.3996640000	13.5320180000	3.1434460000
C	2.3872540000	12.0725550000	4.3465130000
H	1.6424890000	12.7690160000	4.7097900000
C	2.3458610000	10.7498120000	4.7541560000
H	1.5550130000	10.4235470000	5.4193460000
C	1.4600600000	7.8487130000	4.9862420000
C	0.7514720000	7.8122580000	3.7820880000
H	1.2773130000	7.9714370000	2.8460580000
C	-0.6096490000	7.5641740000	3.7739260000
H	-1.1457380000	7.5335570000	2.8339160000
C	-1.2839670000	7.3506100000	4.9701830000
H	-2.3476500000	7.1510740000	4.9648140000
C	-0.5920140000	7.4009230000	6.1699770000
H	-1.1148830000	7.2446320000	7.1050090000
C	0.7737420000	7.6514950000	6.1801030000
H	1.3126660000	7.6783320000	7.1196720000
C	4.2208800000	7.1741920000	2.1950480000
H	7.5783730000	6.6765580000	7.7250040000
O	6.2712020000	5.3540850000	3.2375790000
C	7.4867590000	5.3502590000	3.8069560000
H	7.5000610000	5.7199890000	4.8599810000

C	8.1087500000	3.9547340000	3.7988480000
H	8.5359100000	3.7723300000	2.8120540000
H	7.3221870000	3.2123070000	3.9684130000
O	9.1557960000	3.8151810000	4.7372850000
H	8.7477060000	3.7933350000	5.6103610000
O	8.4529790000	6.1799310000	3.0960460000
C	8.6202060000	7.4687100000	3.5760820000
H	9.4770660000	7.8984410000	3.0504990000
H	8.8832870000	7.4642370000	4.6513530000
C	7.4398020000	8.3899650000	3.3852240000
H	7.0312590000	8.2877030000	2.3797370000
H	7.7395020000	9.4316420000	3.5414830000
O	6.3606230000	8.1011890000	4.2950620000
H	6.7275760000	7.7121790000	5.1065410000

L-iPr-TS2,3

Ru	-0.2203330000	-0.5924900000	-0.9440400000
P	1.8615680000	-0.9066070000	0.0929050000
P	-1.8034620000	-1.3380540000	0.5352840000
N	-0.3264820000	1.3310610000	0.1412140000
C	2.0945760000	0.5101360000	1.2500270000
H	1.2940130000	0.4191290000	1.9913700000
H	3.0512250000	0.4252860000	1.7654350000
C	1.9915920000	1.8134540000	0.5286310000
C	3.0844250000	2.6638430000	0.4061070000
H	4.0302540000	2.3755340000	0.8521240000
C	2.9680870000	3.8841980000	-0.2387270000
H	3.8210380000	4.5450430000	-0.3144320000
C	1.7411900000	4.2544680000	-0.7750270000

H	1.6397660000	5.2060350000	-1.2858740000
C	0.6407840000	3.4193300000	-0.6887800000
C	0.7541470000	2.1831420000	-0.0293890000
C	-0.6746340000	3.7473780000	-1.3283380000
H	-0.6689920000	3.3437260000	-2.3513540000
H	-0.8038210000	4.8264620000	-1.4356190000
C	-1.8182550000	3.1376880000	-0.5743730000
C	-3.0930300000	3.6817290000	-0.5637820000
H	-3.2691390000	4.6162320000	-1.0857820000
C	-4.1444310000	3.0409630000	0.0790790000
H	-5.1367560000	3.4713100000	0.0658530000
C	-3.9064720000	1.8521760000	0.7524150000
H	-4.7116610000	1.3569650000	1.2857100000
C	-2.6313460000	1.3036620000	0.7912390000
C	-1.5775550000	1.9288780000	0.0977310000
C	-2.3090250000	0.0850290000	1.5858170000
H	-3.1445350000	-0.2151130000	2.2217050000
H	-1.4451690000	0.2877640000	2.2243840000
C	2.3272260000	-2.4369200000	1.0430580000
H	1.3830440000	-2.7403210000	1.4938240000
C	2.7648300000	-3.5727080000	0.1287920000
H	3.7725590000	-3.4139040000	-0.2527950000
H	2.1010190000	-3.7050460000	-0.7255040000
H	2.7766380000	-4.5112280000	0.6847210000
C	3.3164000000	-2.2147320000	2.1787210000
H	3.5228940000	-3.1641470000	2.6752660000
H	2.9298500000	-1.5318810000	2.9337460000
H	4.2694300000	-1.8194420000	1.8293870000
C	3.1825840000	-0.5724000000	-1.1667780000

H	2.8518120000	0.4170340000	-1.5083650000
C	3.1864760000	-1.4975560000	-2.3768900000
H	3.7014500000	-1.0155550000	-3.2092660000
H	2.1877850000	-1.7512340000	-2.7204190000
H	3.7122350000	-2.4290880000	-2.1765380000
C	4.5878700000	-0.4388280000	-0.5969890000
H	4.9898070000	-1.4068920000	-0.2946460000
H	4.6422440000	0.2337230000	0.2565690000
H	5.2572180000	-0.0418910000	-1.3617730000
C	-3.4119820000	-1.7231690000	-0.3221840000
H	-3.7149290000	-0.7156120000	-0.6337040000
C	-4.4846030000	-2.2727980000	0.6082460000
H	-5.4536170000	-2.2486810000	0.1090360000
H	-4.5846000000	-1.7035150000	1.5319010000
H	-4.2908340000	-3.3132930000	0.8733430000
C	-3.2954220000	-2.5775730000	-1.5743690000
H	-2.8862910000	-3.5668490000	-1.3683820000
H	-2.6688440000	-2.1106010000	-2.3299620000
H	-4.2834590000	-2.7232770000	-2.0125010000
C	-1.5694630000	-2.6938540000	1.7746820000
H	-2.5659650000	-2.8946630000	2.1768420000
C	-1.0689090000	-3.9665430000	1.1067790000
H	-0.1422370000	-3.8060020000	0.5562050000
H	-1.7936830000	-4.3579510000	0.3934240000
H	-0.8855040000	-4.7451150000	1.8484990000
C	-0.6978050000	-2.2191360000	2.9351450000
H	-0.2827450000	-3.0678720000	3.4804720000
H	-1.2688750000	-1.6217310000	3.6431490000
H	0.1381880000	-1.6000730000	2.6064240000

C	-0.1266490000	-2.2076980000	-1.8217340000
O	-0.0646370000	-3.2331840000	-2.3616920000
H	-1.5920220000	0.0559020000	-1.9385850000
O	0.4951830000	0.8030620000	-2.5389740000
H	-0.8334390000	0.4742620000	-2.3195880000
C	0.8044330000	0.4840080000	-3.8639820000
H	1.0941790000	-0.5649780000	-4.0099000000
H	-0.0619150000	0.6492520000	-4.5299410000
C	1.9470150000	1.3772490000	-4.3092300000
H	2.8091210000	1.2195430000	-3.6579080000
H	1.6561170000	2.4287790000	-4.1893460000
O	2.3829750000	1.0883700000	-5.6238310000
H	1.6508170000	1.2689440000	-6.2207740000

L-iPr-TS3,4

Ru	-0.2216670000	-0.6490820000	-0.9929690000
P	1.9097810000	-0.9064000000	0.0607670000
P	-1.8439920000	-1.3475630000	0.4694160000
N	-0.3551050000	1.3674610000	0.0460060000
C	2.1074370000	0.5368330000	1.1782110000
H	1.3091470000	0.4439240000	1.9220500000
H	3.0649770000	0.4898330000	1.6979870000
C	1.9814890000	1.8290020000	0.4403050000
C	3.0874340000	2.6605730000	0.3091360000
H	4.0384850000	2.3334090000	0.7160750000
C	2.9815460000	3.9067630000	-0.2835030000
H	3.8466650000	4.5500660000	-0.3698510000

C	1.7381460000	4.3298930000	-0.7283490000
H	1.6266740000	5.3178860000	-1.1625490000
C	0.6203000000	3.5158280000	-0.6350360000
C	0.7303310000	2.2252310000	-0.0806570000
C	-0.7168010000	3.9904350000	-1.1210770000
H	-0.7805250000	3.8790300000	-2.2122980000
H	-0.8176670000	5.0661360000	-0.9531500000
C	-1.8478420000	3.2545370000	-0.4665900000
C	-3.1273980000	3.7881850000	-0.4191650000
H	-3.2973330000	4.7694170000	-0.8494720000
C	-4.1843380000	3.0890520000	0.1445620000
H	-5.1794570000	3.5125100000	0.1566650000
C	-3.9372770000	1.8550470000	0.7257710000
H	-4.7362330000	1.3189460000	1.2281990000
C	-2.6596320000	1.3125590000	0.7272960000
C	-1.6012200000	1.9778090000	0.0719360000
C	-2.3403550000	0.0817170000	1.5008780000
H	-3.1736940000	-0.2118520000	2.1430120000
H	-1.4729490000	0.2788750000	2.1367440000
C	2.3667770000	-2.4057650000	1.0657620000
H	1.4187890000	-2.6828420000	1.5267170000
C	2.7869420000	-3.5805100000	0.1930200000
H	3.7810460000	-3.4368270000	-0.2282180000
H	2.0971530000	-3.7567220000	-0.6319120000
H	2.8212680000	-4.4915160000	0.7922950000
C	3.3560050000	-2.1564150000	2.1956040000
H	3.5401140000	-3.0883060000	2.7326120000
H	2.9791820000	-1.4356180000	2.9197090000
H	4.3187410000	-1.7958810000	1.8366360000

C	3.2433800000	-0.6121840000	-1.1936080000
H	2.9857820000	0.4058860000	-1.5058080000
C	3.1582690000	-1.4917590000	-2.4330110000
H	3.8582260000	-1.1233620000	-3.1840720000
H	2.1660220000	-1.4649040000	-2.8759960000
H	3.4223250000	-2.5285780000	-2.2320690000
C	4.6542400000	-0.5889740000	-0.6225190000
H	5.0011520000	-1.5872870000	-0.3540360000
H	4.7448770000	0.0467110000	0.2574100000
H	5.3478330000	-0.2025050000	-1.3706130000
C	-3.4482290000	-1.7756640000	-0.3692630000
H	-3.7605010000	-0.7792000000	-0.7076820000
C	-4.5156280000	-2.3134790000	0.5729680000
H	-5.4846000000	-2.3147160000	0.0732210000
H	-4.6228400000	-1.7205380000	1.4809120000
H	-4.3095330000	-3.3438550000	0.8664490000
C	-3.3170200000	-2.6607950000	-1.5984970000
H	-2.9286270000	-3.6506450000	-1.3577820000
H	-2.6634050000	-2.2224670000	-2.3494410000
H	-4.2961630000	-2.8030220000	-2.0570740000
C	-1.5913250000	-2.6693410000	1.7454540000
H	-2.5862390000	-2.8717930000	2.1509870000
C	-1.0679030000	-3.9537510000	1.1209090000
H	-0.1164870000	-3.8021590000	0.6118220000
H	-1.7593680000	-4.3588490000	0.3825730000
H	-0.9175460000	-4.7184820000	1.8841490000
C	-0.7251130000	-2.1577750000	2.8939240000
H	-0.3012140000	-2.9897530000	3.4579930000
H	-1.2995090000	-1.5491990000	3.5894000000

H	0.1050400000	-1.5400200000	2.5474650000
C	-0.1011680000	-2.3207210000	-1.7298360000
O	-0.0237250000	-3.3831630000	-2.1917080000
O	0.7173250000	0.4807250000	-2.5935190000
C	-0.4582810000	0.2732680000	-3.0675360000
H	-0.6004520000	-0.6071710000	-3.7157380000
C	-1.2893210000	1.4887420000	-3.4329840000
H	-1.6713010000	1.9791870000	-2.5345070000
H	-1.5917120000	-0.1976800000	-1.8546470000
O	-0.4944710000	2.3569120000	-4.2133600000
H	0.3341020000	2.4630730000	-3.7256960000
H	-2.1449440000	1.1942820000	-4.0413950000

L-iPr-TS4,5

Ru	0.7389920000	-1.3177900000	-2.4724120000
P	1.9356580000	-1.1189120000	-0.4524290000
P	-0.9532900000	-2.8324080000	-2.1931380000
N	-0.7025790000	0.2832230000	-1.6762190000
C	0.9691110000	0.0515850000	0.5863440000
H	0.0109570000	-0.4450820000	0.7696230000
H	1.4630730000	0.2014700000	1.5469710000
C	0.7437490000	1.3658940000	-0.0863370000
C	1.3682070000	2.5092600000	0.3941450000
H	2.0524240000	2.4178380000	1.2309960000
C	1.1008850000	3.7570470000	-0.1454940000
H	1.5872870000	4.6408380000	0.2441850000
C	0.1649890000	3.8548840000	-1.1633940000
H	-0.0923200000	4.8287770000	-1.5688160000
C	-0.4638190000	2.7320000000	-1.6815350000

C	-0.1466560000	1.4515770000	-1.1811630000
C	-1.4700770000	2.8789600000	-2.7883800000
H	-0.9710600000	3.1904380000	-3.7191510000
H	-2.1499310000	3.7079250000	-2.5635070000
C	-2.2618310000	1.6275310000	-3.0280570000
C	-3.3909310000	1.6510380000	-3.8371990000
H	-3.6705590000	2.5863020000	-4.3117770000
C	-4.1655300000	0.5169060000	-4.0403020000
H	-5.0387050000	0.5515620000	-4.6775280000
C	-3.8329870000	-0.6469890000	-3.3575050000
H	-4.4654740000	-1.5248010000	-3.4402050000
C	-2.7195880000	-0.6976050000	-2.5326110000
C	-1.8595910000	0.4222780000	-2.4212020000
C	-2.4426810000	-1.8882690000	-1.6823190000
H	-3.3046480000	-2.5581230000	-1.6524650000
H	-2.2327010000	-1.5542850000	-0.6631580000
C	2.2704490000	-2.5897890000	0.6385100000
H	1.3954260000	-3.2153130000	0.4658060000
C	3.5300380000	-0.1985540000	-0.6757310000
H	3.1481540000	0.7483930000	-1.0714970000
C	-1.5455340000	-3.5660670000	-3.7987330000
H	-1.9890820000	-2.6814850000	-4.2713690000
C	-0.9585450000	-4.2399260000	-0.9864700000
H	-1.8474800000	-4.8291400000	-1.2261340000
C	1.8821080000	-2.5994940000	-3.0901710000
O	2.6124660000	-3.4204870000	-3.4648570000
H	-0.1777770000	-1.2168910000	-3.9446820000
O	1.9876740000	0.3299910000	-3.1432050000
C	1.4332550000	1.1638210000	-3.9559880000

H	0.4857860000	1.6311890000	-3.6419880000
C	2.3829340000	2.1957990000	-4.5320640000
H	3.0332600000	1.7283250000	-5.2711730000
H	3.0138200000	2.5385490000	-3.7038960000
O	1.7224590000	3.2708390000	-5.1702260000
H	1.3035030000	3.7991740000	-4.4828660000
O	0.8570610000	0.3063680000	-5.2165610000
C	-0.1247850000	0.9764640000	-6.0105120000
H	0.3928670000	1.6512900000	-6.6945540000
H	-0.7647050000	1.5910600000	-5.3630720000
C	-0.9660800000	-0.0129390000	-6.7740840000
H	-0.3263830000	-0.6761280000	-7.3591320000
H	-1.5875870000	0.5482590000	-7.4842460000
H	0.3310390000	-0.5672550000	-4.5095310000
O	-1.7442800000	-0.8504910000	-5.9486190000
H	-2.3476230000	-0.3024680000	-5.4235800000
C	-0.4504600000	-4.0566260000	-4.7319890000
H	0.1314190000	-4.8743260000	-4.3054900000
H	-0.8982830000	-4.4242060000	-5.6560450000
H	0.2384000000	-3.2598080000	-5.0013390000
C	-2.6312370000	-4.6198880000	-3.6355430000
H	-3.0945290000	-4.8237030000	-4.6013990000
H	-2.2255400000	-5.5658660000	-3.2729970000
H	-3.4268710000	-4.3152120000	-2.9555680000
C	0.2601530000	-5.1282110000	-1.1913780000
H	1.1931520000	-4.5714560000	-1.1073220000
H	0.2831000000	-5.9312740000	-0.4532680000
H	0.2589180000	-5.5896680000	-2.1786820000
C	-1.1207250000	-3.7315870000	0.4442980000

H	-2.1623400000	-3.5146800000	0.6738200000
H	-0.7744190000	-4.4754650000	1.1634010000
H	-0.5618510000	-2.8126680000	0.6289140000
C	4.4506370000	-0.7795300000	-1.7400710000
H	5.2704600000	-0.0834330000	-1.9252400000
H	4.8983360000	-1.7249280000	-1.4377620000
H	3.9300020000	-0.9239460000	-2.6824590000
C	4.2855530000	0.0924500000	0.6133520000
H	3.6546310000	0.5207940000	1.3914440000
H	4.7590020000	-0.8014580000	1.0205150000
H	5.0835620000	0.8100810000	0.4163270000
C	3.4771130000	-3.3905930000	0.1680030000
H	4.4141610000	-2.8864910000	0.4009910000
H	3.4985350000	-4.3578080000	0.6725850000
H	3.4593710000	-3.5805420000	-0.9050570000
C	2.3257910000	-2.3070510000	2.1333260000
H	1.3947530000	-1.8825970000	2.5063010000
H	2.4952030000	-3.2391060000	2.6752370000
H	3.1329690000	-1.6276620000	2.4024260000

L-iPr-TS5,1

Ru	-0.2127040000	-0.7525500000	-1.0297390000
P	1.8942120000	-0.9340170000	0.0047140000
P	-1.8145480000	-1.3841180000	0.4588440000
N	-0.3608180000	1.3705630000	-0.1059700000
C	2.1117960000	0.5615070000	1.0395490000
H	1.3281520000	0.5059450000	1.8023250000
H	3.0776680000	0.5323630000	1.5447420000
C	1.9824310000	1.8294840000	0.2632470000

C	3.0979370000	2.6401270000	0.0931610000
H	4.0555640000	2.2943270000	0.4680710000
C	2.9952710000	3.8891150000	-0.4938400000
H	3.8694220000	4.5126560000	-0.6230570000
C	1.7379250000	4.3447550000	-0.8556220000
H	1.6201820000	5.3449100000	-1.2600810000
C	0.6042880000	3.5603340000	-0.7044370000
C	0.7159050000	2.2394400000	-0.2165090000
C	-0.7396390000	4.1488570000	-1.0199920000
H	-0.7671620000	4.5110040000	-2.0523970000
H	-0.8716280000	5.0612410000	-0.4245720000
C	-1.8778510000	3.2204560000	-0.7252100000
C	-3.1894790000	3.6451070000	-0.8921170000
H	-3.3633710000	4.6200990000	-1.3369980000
C	-4.2659790000	2.8719620000	-0.4855200000
H	-5.2805390000	3.2132120000	-0.6400530000
C	-4.0122410000	1.6923560000	0.1970090000
H	-4.8293690000	1.1248220000	0.6311600000
C	-2.7111310000	1.2505320000	0.3885950000
C	-1.6222580000	1.9459860000	-0.1860930000
C	-2.4083480000	0.1181410000	1.3072460000
H	-3.2744740000	-0.1328840000	1.9231140000
H	-1.5908560000	0.4123060000	1.9703310000
C	2.3730630000	-2.3674680000	1.0955470000
H	1.4299020000	-2.6211380000	1.5796490000
C	2.8039480000	-3.5935990000	0.3024250000
H	3.7924950000	-3.4640630000	-0.1364210000
H	2.1109930000	-3.8403490000	-0.5005060000
H	2.8592920000	-4.4585950000	0.9651010000

C	3.3665170000	-2.0391910000	2.2019600000
H	3.5661920000	-2.9369350000	2.7892360000
H	2.9860910000	-1.2834510000	2.8872610000
H	4.3224490000	-1.6886080000	1.8155450000
C	3.2140250000	-0.7100800000	-1.2813070000
H	2.9474990000	0.2859080000	-1.6544000000
C	3.1234470000	-1.6696910000	-2.4601160000
H	3.8257960000	-1.3565350000	-3.2338910000
H	2.1317940000	-1.6702640000	-2.9051740000
H	3.3826440000	-2.6915480000	-2.1886530000
C	4.6312330000	-0.6428860000	-0.7291340000
H	4.9852470000	-1.6208690000	-0.4016280000
H	4.7282710000	0.0472710000	0.1078110000
H	5.3150530000	-0.3019190000	-1.5077910000
C	-3.3746100000	-1.9676190000	-0.3725430000
H	-3.7141150000	-1.0299070000	-0.8311690000
C	-4.4453550000	-2.4398640000	0.6018750000
H	-5.3998040000	-2.5325740000	0.0830650000
H	-4.5988530000	-1.7584620000	1.4383400000
H	-4.2105470000	-3.4244030000	1.0093190000
C	-3.1831030000	-2.9774990000	-1.4921750000
H	-2.7406110000	-3.9101910000	-1.1420410000
H	-2.5648990000	-2.5823350000	-2.2934170000
H	-4.1503010000	-3.2233470000	-1.9317480000
C	-1.5450230000	-2.5576220000	1.8676100000
H	-2.5380500000	-2.7199170000	2.2958470000
C	-1.0177990000	-3.9011730000	1.3862190000
H	-0.0891740000	-3.8022580000	0.8249090000
H	-1.7291060000	-4.4046440000	0.7325580000

H	-0.8246390000	-4.5628270000	2.2318440000
C	-0.6792670000	-1.9140170000	2.9478530000
H	-0.2467970000	-2.6732580000	3.6010780000
H	-1.2566860000	-1.2352180000	3.5723610000
H	0.1440480000	-1.3327580000	2.5296490000
C	-0.0607250000	-2.4710940000	-1.6109930000
O	0.0608640000	-3.5719740000	-1.9650360000
O	0.5409240000	0.1324980000	-2.9343570000
C	-0.6737330000	0.0689070000	-3.2952240000
H	-1.5465490000	-0.3172370000	-2.0422140000
C	-1.1876440000	-1.0318810000	-4.2143220000
H	-0.7127750000	-0.8354980000	-5.1881930000
H	-0.8565650000	-2.0109450000	-3.8772830000
O	-2.5854320000	-1.0526840000	-4.3091690000
H	-2.8626770000	-0.1363060000	-4.4327650000
O	-1.3345410000	1.2212460000	-3.6618300000
C	-0.5140110000	2.3782530000	-3.7972230000
H	-0.0400150000	2.6323640000	-2.8511590000
H	0.2957410000	2.1659630000	-4.5070580000
H	-2.1968540000	3.6861340000	-3.5913720000
C	-1.3986120000	3.4881470000	-4.3085070000
H	0.0270410000	4.5458480000	-5.0955210000
O	-0.6751130000	4.6932980000	-4.4542860000
H	-1.8774540000	3.1833220000	-5.2476470000

LS-Ph-TS2,3

Ru	-0.4612660000	4.3277430000	2.8856040000
H	1.0151000000	5.3813790000	3.1038900000
P	-1.8671680000	2.4832890000	2.8495980000

P	-1.8195880000	5.8846590000	3.7975250000
O	-1.0467740000	5.3358700000	0.1190230000
N	-0.1559450000	3.7612880000	4.9607580000
C	-0.6685450000	1.2230550000	3.3926430000
H	-1.0927630000	0.2299970000	3.2255890000
H	0.1572130000	1.3365630000	2.6820870000
C	-0.1170000000	1.3039320000	4.7892520000
C	0.1311220000	0.0856520000	5.4158150000
H	-0.1013520000	-0.8226100000	4.8707630000
C	0.6403770000	-0.0004640000	6.6981770000
H	0.8122110000	-0.9632750000	7.1594840000
C	0.9263670000	1.1746110000	7.3709560000
H	1.3360450000	1.1469210000	8.3745180000
C	0.7130570000	2.4051670000	6.7758210000
C	0.1672250000	2.5066670000	5.4773360000
C	1.0809500000	3.6747660000	7.4753160000
H	1.1915350000	3.5164270000	8.5489030000
C	0.0811530000	4.7410700000	7.1782360000
C	-0.4294330000	4.7636210000	5.8602380000
C	-0.2806320000	5.7190540000	8.0807490000
H	0.1257160000	5.6715710000	9.0854190000
C	-1.1093310000	6.7816700000	7.7214740000
H	-1.3591860000	7.5514440000	8.4384190000
C	-1.5719010000	6.8525530000	6.4237270000
H	-2.1871950000	7.6891370000	6.1102900000
C	-1.2561160000	5.8557590000	5.4988930000
C	-2.4269800000	1.8185160000	1.2132080000
H	-2.8034720000	0.8151900000	1.4341880000
C	-1.2906690000	1.6736420000	0.2148030000

H	-0.8432470000	2.6350500000	-0.0332260000
H	-1.6686780000	1.2450420000	-0.7140050000
H	-0.4995860000	1.0191140000	0.5760650000
C	-3.5617040000	2.6538770000	0.6394040000
H	-4.4165200000	2.7242190000	1.3118000000
H	-3.9141510000	2.2189220000	-0.2963200000
H	-3.2283180000	3.6679390000	0.4175040000
C	-3.3867390000	2.2881460000	3.8996100000
H	-4.0743610000	3.0466560000	3.5107750000
C	-3.1390990000	2.5872050000	5.3709770000
H	-2.5191600000	1.8211630000	5.8361250000
H	-4.0930860000	2.6077430000	5.8999810000
H	-2.6536330000	3.5463600000	5.5297550000
C	-4.0423200000	0.9197300000	3.7509800000
H	-4.4239950000	0.7311990000	2.7497340000
H	-4.8877980000	0.8482550000	4.4359880000
H	-3.3537680000	0.1143280000	4.0122260000
C	-1.6246400000	7.6084910000	3.2185600000
C	-0.4418040000	8.0164810000	2.6048090000
H	0.3420400000	7.2913250000	2.4258650000
C	-0.2642590000	9.3344650000	2.2180460000
H	0.6585540000	9.6316040000	1.7369150000
C	-1.2658680000	10.2671580000	2.4399780000
H	-1.1281030000	11.2962710000	2.1343080000
C	-2.4475170000	9.8747030000	3.0492480000
H	-3.2355600000	10.5958580000	3.2240080000
C	-2.6283690000	8.5554580000	3.4326770000
H	-3.5606750000	8.2595910000	3.8982410000
C	-3.6334920000	5.7127710000	3.7640290000

C	-4.2584110000	5.6774650000	2.5155220000
H	-3.6644440000	5.7988350000	1.6155840000
C	-5.6258240000	5.4890460000	2.4176100000
H	-6.0966440000	5.4593900000	1.4431260000
C	-6.3891940000	5.3340020000	3.5680270000
H	-7.4579270000	5.1807790000	3.4928670000
C	-5.7795490000	5.3833450000	4.8119560000
H	-6.3715720000	5.2725670000	5.7115040000
C	-4.4082900000	5.5751310000	4.9117420000
H	-3.9339630000	5.6055180000	5.8853410000
C	-0.7953370000	4.9206070000	1.1722140000
H	2.0656210000	4.0094340000	7.1168460000
C	3.4208830000	2.6198840000	1.3565250000
H	3.4441280000	1.6411060000	1.8368640000
C	1.9900810000	3.1195840000	1.2821620000
O	1.4508700000	3.2100180000	2.5670130000
H	1.9774630000	4.0935680000	0.7585920000
H	1.4101120000	2.4352060000	0.6461840000
H	1.3946170000	4.5433780000	2.9244980000
H	4.0149720000	3.2959760000	1.9848330000
O	3.9940250000	2.4497650000	0.0736820000
H	4.0025150000	3.3096950000	-0.3572840000

LS-Ph-TS3,4

Ru	-0.3139170000	4.6448340000	2.9317990000
P	-1.5999010000	2.6478980000	2.9198510000
P	-1.7141740000	6.1378950000	3.8761810000
O	-1.0789970000	5.6620320000	0.2189560000
N	0.0214530000	4.0788980000	5.0287610000

C	-0.3848240000	1.4382220000	3.5357430000
H	-0.8297070000	0.4407340000	3.4895800000
H	0.3959990000	1.4522240000	2.7715180000
C	0.2527590000	1.6235940000	4.8881140000
C	0.6631280000	0.4431660000	5.5067130000
H	0.4889190000	-0.4894560000	4.9815540000
C	1.2536520000	0.4201910000	6.7544850000
H	1.5510140000	-0.5156780000	7.2074300000
C	1.4490130000	1.6247690000	7.4075190000
H	1.9100550000	1.6495590000	8.3885080000
C	1.0792580000	2.8210170000	6.8212090000
C	0.4601910000	2.8597340000	5.5479930000
C	1.3495040000	4.1204650000	7.5094810000
H	1.4994280000	3.9733890000	8.5800020000
C	0.2537500000	5.0930790000	7.2368880000
C	-0.2889460000	5.0675500000	5.9314400000
C	-0.1572660000	6.0474820000	8.1432660000
H	0.2771740000	6.0367210000	9.1372310000
C	-1.0668160000	7.0478640000	7.7992250000
H	-1.3569080000	7.8000850000	8.5196770000
C	-1.5499930000	7.0863130000	6.5082670000
H	-2.2161280000	7.8842880000	6.1972440000
C	-1.1815170000	6.1085050000	5.5814460000
C	-2.0332330000	1.9199430000	1.2722660000
H	-2.3184440000	0.8819220000	1.4728850000
C	-0.8448490000	1.9101380000	0.3223180000
H	-0.4837270000	2.9181040000	0.1222420000
H	-1.1381920000	1.4715150000	-0.6320930000
H	-0.0050820000	1.3335430000	0.7042880000

C	-3.2186840000	2.6469760000	0.6537630000
H	-4.1013340000	2.6368540000	1.2933720000
H	-3.4939930000	2.1842410000	-0.2945110000
H	-2.9716170000	3.6883800000	0.4436320000
C	-3.1392020000	2.3910500000	3.9243930000
H	-3.8500600000	3.1190070000	3.5201940000
C	-2.9153230000	2.7154060000	5.3946350000
H	-2.2437940000	1.9962450000	5.8647740000
H	-3.8667740000	2.6769350000	5.9270770000
H	-2.4917270000	3.7060660000	5.5436940000
C	-3.7356490000	0.9980510000	3.7715350000
H	-4.0491240000	0.7765070000	2.7530400000
H	-4.6164150000	0.9058450000	4.4081770000
H	-3.0347430000	0.2234740000	4.0866230000
C	-1.5456120000	7.8819390000	3.3542010000
C	-0.3017770000	8.4922860000	3.5248830000
H	0.5141380000	7.9227340000	3.9564250000
C	-0.1060260000	9.8110690000	3.1601610000
H	0.8632870000	10.2701710000	3.3057750000
C	-1.1493060000	10.5433570000	2.6082030000
H	-0.9949180000	11.5742450000	2.3169180000
C	-2.3873840000	9.9495080000	2.4362620000
H	-3.2076010000	10.5139760000	2.0118200000
C	-2.5882160000	8.6277600000	2.8120580000
H	-3.5667320000	8.1842520000	2.6842520000
C	-3.5106090000	5.8477450000	3.7843860000
C	-4.0891460000	5.6569010000	2.5270060000
H	-3.4741390000	5.7314190000	1.6367890000
C	-5.4372030000	5.3652060000	2.4071450000

H	-5.8701400000	5.2185430000	1.4256320000
C	-6.2253910000	5.2483840000	3.5446310000
H	-7.2769540000	5.0089780000	3.4536550000
C	-5.6610100000	5.4385770000	4.7965120000
H	-6.2711080000	5.3483860000	5.6861680000
C	-4.3121830000	5.7418450000	4.9175010000
H	-3.8727830000	5.8740510000	5.8989050000
C	-0.7520580000	5.2422550000	1.2510760000
H	2.2878600000	4.5384680000	7.1200740000
C	3.1032150000	5.0407230000	3.3776310000
H	4.0173510000	4.8143900000	2.7996040000
C	1.9736380000	4.6800820000	2.4454110000
O	1.5087780000	3.4926680000	2.4825890000
H	0.8174540000	5.8487030000	3.1742630000
H	2.0001960000	5.2152040000	1.4835270000
H	3.1240560000	6.1133310000	3.5797430000
O	3.0803390000	4.3403460000	4.5802120000
H	2.5534780000	3.5410030000	4.4218320000

LS-Ph-TS4,5

Ru	1.2259360000	7.8785340000	1.5313630000
H	2.2618020000	9.2693780000	1.6644490000
P	0.1275200000	5.8141740000	1.6253700000
P	-0.2884650000	9.3003450000	2.3824350000
O	0.4459030000	8.6846780000	-1.2435540000
N	1.6384670000	7.4685400000	3.6764430000
C	1.4013650000	4.7233800000	2.3418960000
H	1.0208630000	3.6987830000	2.3452650000
H	2.1957300000	4.7507120000	1.5918040000

C	1.9867350000	5.0228030000	3.6983690000
C	2.4465770000	3.9057110000	4.3935040000
H	2.3405710000	2.9371690000	3.9173730000
C	2.9982680000	3.9824960000	5.6579890000
H	3.3321930000	3.0909290000	6.1707270000
C	3.0944800000	5.2279080000	6.2513630000
H	3.5119230000	5.3316350000	7.2468470000
C	2.6721410000	6.3675200000	5.5893740000
C	2.1051080000	6.3051800000	4.2934370000
C	2.8331330000	7.7066140000	6.2375300000
H	2.8757830000	7.6076160000	7.3237980000
C	1.7411980000	8.6360050000	5.8248240000
C	1.2345760000	8.4898110000	4.5116000000
C	1.2874290000	9.6536330000	6.6442170000
H	1.7021740000	9.7334110000	7.6435000000
C	0.3526960000	10.5871680000	6.2082790000
H	0.0297500000	11.3897060000	6.8564850000
C	-0.1293030000	10.4826570000	4.9180540000
H	-0.8344970000	11.2144720000	4.5387970000
C	0.2840450000	9.4496620000	4.0787840000
C	-0.2265770000	4.9575000000	0.0225660000
H	-0.4435440000	3.9164970000	0.2838220000
C	0.9745550000	4.9750950000	-0.9111900000
H	1.2782310000	5.9912010000	-1.1576370000
H	0.7235540000	4.4673790000	-1.8433300000
H	1.8438740000	4.4773530000	-0.4867300000
C	-1.4503160000	5.5629070000	-0.6503580000
H	-2.3383120000	5.5351580000	-0.0183630000
H	-1.6827910000	5.0232050000	-1.5689080000

H	-1.2691030000	6.6023470000	-0.9257280000
C	-1.4038560000	5.4948470000	2.6282680000
H	-2.1608130000	6.1478510000	2.1853060000
C	-1.2218000000	5.9038500000	4.0831610000
H	-0.5281530000	5.2404290000	4.6005650000
H	-2.1793230000	5.8503730000	4.6030500000
H	-0.8454130000	6.9194000000	4.1864600000
C	-1.9005680000	4.0575960000	2.5407840000
H	-2.1884430000	3.7656920000	1.5325640000
H	-2.7790260000	3.9355070000	3.1758070000
H	-1.1513410000	3.3488890000	2.8975470000
C	-0.3095800000	11.0154840000	1.7438850000
C	0.8691310000	11.7601700000	1.8308410000
H	1.7546950000	11.3231930000	2.2777410000
C	0.9135560000	13.0601120000	1.3597200000
H	1.8339150000	13.6240490000	1.4415570000
C	-0.2109340000	13.6370230000	0.7854270000
H	-0.1718460000	14.6523220000	0.4120020000
C	-1.3839690000	12.9074400000	0.6959340000
H	-2.2683550000	13.3490650000	0.2548090000
C	-1.4365100000	11.6062010000	1.1763070000
H	-2.3669540000	11.0588710000	1.1094280000
C	-2.0573990000	8.8605190000	2.3798670000
C	-2.6408550000	8.5234780000	1.1557190000
H	-2.0426140000	8.5599100000	0.2513030000
C	-3.9677660000	8.1346910000	1.0885920000
H	-4.4049960000	7.8772420000	0.1320840000
C	-4.7286060000	8.0630300000	2.2484680000
H	-5.7629740000	7.7481760000	2.2002350000

C	-4.1588530000	8.3955860000	3.4676260000
H	-4.7476400000	8.3407960000	4.3744240000
C	-2.8322940000	8.7990580000	3.5337700000
H	-2.3898170000	9.0464460000	4.4913600000
C	0.7784360000	8.3478230000	-0.1851100000
H	3.8057490000	8.1411920000	5.9564140000
C	4.0863540000	6.8818830000	1.5935400000
H	4.0595030000	6.5251400000	2.6465380000
C	5.3467080000	6.4196890000	0.8913560000
H	5.5215170000	7.0482730000	0.0196410000
H	5.1603900000	5.4002870000	0.5314470000
O	6.5037040000	6.4912630000	1.6991680000
H	6.4123320000	5.8378330000	2.3998240000
O	4.3489190000	8.4734020000	1.8760140000
C	4.8943390000	8.7446100000	3.1663140000
H	5.9331300000	8.4084820000	3.1624550000
H	4.3508810000	8.1539130000	3.9156760000
C	4.8232980000	10.2094060000	3.5044010000
H	5.3110600000	10.7988210000	2.7271200000
H	5.3885920000	10.3663140000	4.4329950000
H	3.1938740000	8.8677700000	1.7819850000
O	3.5095030000	10.7126970000	3.6063720000
H	3.0278190000	10.2066830000	4.2758320000
O	3.0142870000	6.7675180000	0.8892560000

LS-Ph-TS5,1

Ru	0.3792660000	4.2759890000	3.0096150000
P	-1.0947580000	2.4710930000	3.1329570000
P	-0.8817340000	5.9905180000	3.7184890000

O	-0.2354140000	5.0863120000	0.1967080000
N	0.5570300000	3.9063280000	5.1857900000
C	-0.0043570000	1.2183720000	3.8880550000
H	-0.5286990000	0.2587460000	3.8989640000
H	0.7967560000	1.1119290000	3.1519950000
C	0.6111590000	1.4499340000	5.2440970000
C	0.9685310000	0.2916860000	5.9309230000
H	0.7800960000	-0.6615420000	5.4495900000
C	1.5266860000	0.3164730000	7.1948920000
H	1.7872880000	-0.6017810000	7.7029780000
C	1.7117520000	1.5476010000	7.8011620000
H	2.1212780000	1.6093770000	8.8038430000
C	1.3887000000	2.7250610000	7.1476070000
C	0.8522760000	2.7140210000	5.8370010000
C	1.6435510000	4.0379930000	7.8210980000
H	1.5573170000	3.9330630000	8.9048340000
C	0.7309780000	5.1034910000	7.3134600000
C	0.3025410000	5.0113050000	5.9700300000
C	0.3824390000	6.1971900000	8.0808140000
H	0.7264060000	6.2344740000	9.1090400000
C	-0.3519120000	7.2606470000	7.5619100000
H	-0.5938010000	8.1162360000	8.1770450000
C	-0.7318650000	7.2148350000	6.2365290000
H	-1.2718120000	8.0456140000	5.7954330000
C	-0.4261920000	6.1066610000	5.4468000000
C	-1.5412080000	1.6440870000	1.5361310000
H	-1.9385510000	0.6632250000	1.8174100000
C	-0.3311840000	1.4208940000	0.6407970000
H	0.1485350000	2.3594890000	0.3670590000

H	-0.6420820000	0.9310730000	-0.2825790000
H	0.4258560000	0.7929270000	1.1058300000
C	-2.6272690000	2.4241980000	0.8082430000
H	-3.5257470000	2.5595050000	1.4103250000
H	-2.9185740000	1.9037480000	-0.1046000000
H	-2.2724710000	3.4127630000	0.5158900000
C	-2.6867060000	2.4406310000	4.0854290000
H	-3.3039320000	3.2026280000	3.5972760000
C	-2.4872160000	2.8514930000	5.5368300000
H	-1.9106730000	2.1067780000	6.0866910000
H	-3.4571350000	2.9473060000	6.0269740000
H	-1.9712560000	3.8037600000	5.6323200000
C	-3.4144630000	1.1046850000	4.0081840000
H	-3.7248460000	0.8430730000	2.9985370000
H	-4.3149040000	1.1446170000	4.6222760000
H	-2.8020820000	0.2894730000	4.3968800000
C	-0.5639550000	7.6282540000	2.9748550000
C	0.6578970000	7.8961810000	2.3598480000
H	1.4051210000	7.1156480000	2.2756830000
C	0.9246030000	9.1540150000	1.8443360000
H	1.8762870000	9.3388620000	1.3627680000
C	-0.0234100000	10.1616980000	1.9357140000
H	0.1843980000	11.1428810000	1.5284110000
C	-1.2438160000	9.9058650000	2.5423990000
H	-1.9912080000	10.6857370000	2.6127770000
C	-1.5150880000	8.6475310000	3.0550640000
H	-2.4770140000	8.4549970000	3.5153100000
C	-2.6992110000	5.8861380000	3.6030210000
C	-3.2636000000	5.7375020000	2.3336640000

H	-2.6247200000	5.7473590000	1.4569860000
C	-4.6300290000	5.5769820000	2.1847090000
H	-5.0528430000	5.4587590000	1.1950200000
C	-5.4542060000	5.5634660000	3.3031930000
H	-6.5223740000	5.4322570000	3.1882930000
C	-4.9050210000	5.7255410000	4.5653700000
H	-5.5435960000	5.7247960000	5.4396060000
C	-3.5344640000	5.8890500000	4.7161740000
H	-3.1086220000	6.0056420000	5.7055610000
C	0.0338980000	4.7399060000	1.2725510000
H	2.6875980000	4.3382050000	7.6506880000
C	3.2733520000	4.6937660000	1.3288220000
H	2.4644910000	4.6561560000	0.6042530000
C	2.8198410000	4.0754060000	2.6419350000
O	2.1526900000	2.9936330000	2.6688490000
H	1.8455810000	5.2098980000	3.0863330000
H	4.0874970000	4.0545120000	0.9588300000
O	3.6762400000	6.0314130000	1.4620510000
H	4.2824050000	6.0594280000	2.2120440000
O	3.8328800000	4.2986460000	3.5424300000
C	3.6904650000	3.7195610000	4.8347630000
H	3.1279850000	2.7868710000	4.7523260000
H	3.1179980000	4.4057500000	5.4636160000
C	5.0444890000	3.4544710000	5.4405720000
H	5.6266500000	4.3737640000	5.5202210000
H	5.6141910000	2.7633290000	4.8060290000
O	4.8907330000	2.9622620000	6.7553420000
H	4.2575260000	2.2330300000	6.7311170000

H_2

H	0.0331010000	1.4635960000	0.0000000000
H	-0.7109610000	1.4635960000	0.0000000000

EG

C	-2.2151100000	0.0242640000	0.0002210000
H	-1.8194920000	0.5405400000	0.8835660000
H	-1.8195440000	0.5406540000	-0.8830860000
C	-1.7292320000	-1.4019040000	0.0001040000
H	-2.1249780000	-1.9182080000	-0.8831710000
H	-2.1246710000	-1.9182670000	0.8834800000
O	-0.3164760000	-1.3621900000	-0.0001400000
H	0.0096950000	-2.2644970000	-0.0001890000
O	-3.6278660000	-0.0154500000	0.0002570000
H	-3.9540370000	0.8868570000	0.0008880000

HEG

H	-2.3747670000	-1.4615830000	3.6618100000
O	-1.6938580000	-2.0130150000	3.2476900000
C	-1.4374020000	-3.0344290000	4.1605710000
H	-0.3778380000	-3.0582600000	4.4419690000
H	-1.6633230000	-4.0198790000	3.7344560000
C	-2.2608270000	-2.8596220000	5.4058710000
O	-3.0462850000	-1.9531120000	5.5461610000
O	-2.0208670000	-3.8076020000	6.3096350000
C	-2.7926660000	-3.7166280000	7.5199260000
H	-3.8537320000	-3.7122380000	7.2666630000
H	-2.5635600000	-2.7756290000	8.0225140000
C	-2.4468430000	-4.8890680000	8.3836580000

H	-2.8879340000	-4.7199000000	9.3748500000
H	-1.3587850000	-4.9389790000	8.5124650000
O	-2.9551550000	-6.0562720000	7.7768060000
H	-2.6401420000	-6.8179270000	8.2691310000

THF

O	2.6749470000	0.8112630000	-1.6152830000
C	3.1054460000	1.9091030000	-0.8186140000
H	2.5856730000	2.8098120000	-1.1520200000
H	4.1803370000	2.0716010000	-0.9711020000
C	2.8074510000	1.5348940000	0.6203230000
H	3.4597510000	2.0342600000	1.3341370000
C	2.9810600000	0.0245210000	0.5811950000
H	4.0402440000	-0.2360390000	0.6208230000
H	2.4811830000	-0.4968620000	1.3952540000
C	2.4034640000	-0.3088160000	-0.7805000000
H	2.8424480000	-1.1994260000	-1.2350830000
H	1.3186950000	-0.4671530000	-0.7224140000
H	1.7777520000	1.7910100000	0.8761440000

Glycolaldehyde

O	0.9777470000	0.0922160000	-0.1873730000
C	2.1609800000	-0.0633870000	-0.0777820000
C	2.9847990000	-0.9622160000	-0.9695910000
O	4.3703800000	-0.8151120000	-0.7866210000
H	2.7597920000	0.4497910000	0.7147290000
H	2.6648050000	-0.8249190000	-2.0090440000
H	2.7391900000	-1.9955500000	-0.7105290000
H	4.6415060000	0.0253000000	-1.1659690000

10. References

1. Tang, S.; Rauch, M.; Montag, M.; Diskin-Posner, Y.; Ben-David, Y.; Milstein. D., Catalytic Oxidative Deamination by Water with H_2 Liberation. *J. Am. Chem. Soc.* **2020**, *142*, 20875-20882.
2. Wray, B. C.; Stambuli, J. P. Synthesis of *N*-Arylindazoles and Benzimidazoles from a Common Intermediate. *Org. Lett.* **2010**, *12*, 4576-4579.
3. Wang, T.-J.; Chen, W.-W.; Li, Y.; Xu, M.-H. Facile synthesis of acridines via Pd(0)-diphosphine complex-catalyzed tandem coupling/cyclization protocol, *Org. Biomol. Chem.* **2015**, *13*, 6580-6586.
4. Zou, S.; Yu, B.; Huang, H. Enantioselective ring-closing aminomethylamination of aminodienes enabled by modified Trost ligands. *Chem Catal.* **2022**, *2*, 2034-2048.
5. Vallée, M. R. J.; Majkut, P.; Wilkening, I.; Weise, C.; Müller, G.; Hackenberger, C. P. R. Staudinger-Phosphonite Reactions for the Chemoselective Transformation of Azido-Containing Peptides and Proteins. *Org. Lett.* **2011**, *13*, 5440-5443.
6. Provis-Evans, C. B.; Emanuelsson, E. A. C.; Webstera, R. L. Rapid Metal-Free Formation of Free Phosphines from Phosphine Oxides. *Adv. Synth. Catal*. **2018**, *360*, 3999-4004.
7. Evans, I. P.; Spencer, A.; Wilkinson, G. Dichlorotetrakis-(dimethy1 sulfoxide)ruthenium(II) and its Use as a Source Material for Some New Ruthenium(II) Complexes. *J. Chem. Soc., Dalton Trans.* **1973**, *204*, 204-209.
8. Alessio, E.; Milani, B.; Bolle, M.; Mestroni, G.; Faleschini, P.; Todone, F.; Geremia, S.; Calligaris, M. Carbonyl Derivatives of Chloride-Dimethyl Sulfoxide-Ruthenium (II) Complexes: Synthesis, Structural Characterization, and Reactivity of $Ru(CO)_x(DMSO)_{4-x}Cl_2$ Complexes (x = 1-3). *Inorg. Chem.* **1995**, *34*, 4722-4734.
9. Sheldrick, G. M. *SHELXT*-Integrated space-group and crystal-structure determination. *Acta Crystallogr. Sect. A* **2015**, *71*, 3-8.
10. Sheldrick, G. M. A short history of *SHELX. Acta Crystallogr. Sect. A* **2008,** *64*, 112-122.
11. Dolomanov, O. V.; Bourhis, L. J.; Gildea, R. J.; Howard, J. A. K.; Puschmann, H. *OLEX2*: a complete structure solution, refinement and analysis program. *J. Appl. Crystallogr*. **2009**, *42*, 339-341.

12. Frisch, M. J.; Trucks, G. W.; Schlegel, H. B.; Scuseria, G. E.; Robb, M. A.; Cheeseman, J. R.; Scalmani, G.; Barone, V.; Petersson, G. A.; Nakatsuji, H.; Li, X.; Caricato, M.; Marenich, A. V.; Bloino, J.; Janesko, B. G.; Gomperts, R.; Mennucci, B.; Hratchian, H. P.; Ortiz, J. V.; Izmaylov, A. F.; Sonnenberg, J. L.; Williams-Young, D.; Ding, F.; Lipparini, F.; Egidi, F.; Goings, J.; Peng, B.; Petrone, A.; Henderson, T.; Ranasinghe, D.; Zakrzewski, V. G.; Gao, J.; Rega, N.; Zheng, G.; Liang, W.; Hada, M.; Ehara, M.; Toyota, K.; Fukuda, R.; Hasegawa, J.; Ishida, M.; Nakajima, T.; Honda, Y.; Kitao, O.; Nakai, H.; Vreven, T.; Throssell, K.; Montgomery, J. A., Jr.; Peralta, J. E.; Ogliaro, F.; Bearpark, M. J.; Heyd, J. J.; Brothers, E. N.; Kudin, K. N.; Staroverov, V. N.; Keith, T. A.; Kobayashi, R.; Normand, J.; Raghavachari, K.; Rendell, A. P.; Burant, J. C.; Iyengar, S. S.; Tomasi, J.; Cossi, M.; Millam, J. M.; Klene, M.; Adamo, C.; Cammi, R.; Ochterski, J. W.; Martin, R. L.; Morokuma, K.; Farkas, O.; Foresman, J. B.; Fox, D. J. *Gaussian 16, Revision C.01;* Gaussian, Inc., Wallingford CT, 2016.

13. Zhao, Y.; Truhlar, D. G. A New Local Density Functional for Main-Group Thermochemistry, Transition Metal Bonding, Thermochemical Kinetics, and Noncovalent Interactions. *J. Chem. Phys.* **2006,** *125*, 194101.

14. Weigend, F.; Ahlrichs, R. Balanced Basis Sets of Split Valence, Triple Zeta Valence and Quadruple Zeta Valence Quality for H to Rn: Design and Assessment of Accuracy. *Phys. Chem. Chem. Phys.* **2005,** *7*, 3297-3305.

15. Weigend, F. Accurate Coulomb-Fitting Basis Sets for H to Rn. *Phys. Chem. Chem. Phys.* **2006,** *8*, 1057-1065.

16. Grimme, S.; Antony, J.; Ehrlich, S.; Krieg, H. A Consistent and Accurate *ab initio* Parametrization of Density Functional Dispersion Correction (DFT-D) for the 94 Elements H-Pu. *J. Chem. Phys.* **2010,** *132*, 154104.

17. Neese, F. Software Update: The ORCA Program System—Version 5.0. *WIREs Comput. Mol. Sci.* **2022,** *12*, e1606.

18. Mardirossian, N.; Head-Gordon, M. ωB97X-V: A 10-Parameter, Range-Separated Hybrid, Generalized Gradient Approximation Density Functional with Nonlocal Correlation, Designed by a Survival-of-the-Fittest Strategy. *Phys. Chem. Chem. Phys.* **2014,** *16*, 9904-9924.

19. Vydrov, O. A.; Van Voorhis, T. Nonlocal van der Waals Density Functional: The Simpler the

Better. *J. Chem. Phys.* **2010,** *133*, 244103.

20. Hujo, W.; Grimme, S. Performance of the van der Waals Density Functional VV10 and (hybrid) GGA Variants for Thermochemistry and Noncovalent Interactions. *J. Chem. Theory Comput.* **2011,** *7*, 3866-3871.
21. Hellweg, A.; Hattig, C.; Hofener, S.; Klopper, W. Optimized Accurate Auxiliary Basis Sets for RI-MP2 and RI-CC2 Calculations for the Atoms Rb to Rn. *Theor. Chem. Acc.* **2007,** *117*, 587-597.
22. Iron, M. A.; Janes, T. Evaluating Transition Metal Barrier Heights with the Latest Density Functional Theory Exchange-Correlation Functionals: The MOBH35 Benchmark Database. *J. Phys. Chem. A* **2019,** *123*, 3761-3781.
23. Cramer, C. J. *Essentials of Computational Chemistry: Theories and Models. 2nd Ed.;* John Wiley & Sons Ltd: West Sussex, England, 2004.
24. Sparta, M.; Riplinger, C.; Neese, F. Mechanism of Olefin Asymmetric Hydrogenation Catalyzed by Iridium Phosphino-Oxazoline: A Pair Natural Orbital Coupled Cluster Study. *J. Chem. Theory Comput.* **2014,** *10*, 1099-1108.
25. Hopmann, K. H. How Accurate Is Dft for Iridium-Mediated Chemistry? *Organometallics* **2016,** *35*, 3795-3807.
26. Gusev, D. G. Revised Mechanisms of the Catalytic Alcohol Dehydrogenation and Ester Reduction with the Milstein PNN Complex of Ruthenium. *Organometallics* **2020,** *39*, 258-270.
